# *LincIN*, a novel NF90-binding long non-coding RNA, is overexpressed in advanced breast tumors and involved in metastasis

**DOI:** 10.1186/s13058-017-0853-2

**Published:** 2017-05-30

**Authors:** Zhengyu Jiang, Carolyn M. Slater, Yan Zhou, Karthik Devarajan, Karen J. Ruth, Yueran Li, Kathy Q. Cai, Mary Daly, Xiaowei Chen

**Affiliations:** 1 Cancer Epigenetics Program, Fox Chase Cancer Center, Philadelphia, PA 19111 USA; 2 Department of Biostatistics and Bioinformatics, Fox Chase Cancer Center, Philadelphia, PA 19111 USA; 3 Cancer Biology Program, Fox Chase Cancer Center, Philadelphia, PA 19111 USA; 4 Department of Clinical Genetics, Fox Chase Cancer Center, Philadelphia, PA 19111 USA; 5 Present Address: Department of Medicine, Irving Cancer Research Center, Columbia University, New York, NY 10032 USA; 6 Present Address: The Third Xiangya Hospital of Central South University, Changsha, China

**Keywords:** lncRNA, LincIN, NF90, Metastasis, Breast cancer

## Abstract

**Background:**

Recent genome-wide profiling by sequencing and distinctive chromatin signatures has identified thousands of long non-coding RNA (lncRNA) species (>200 nt). LncRNAs have emerged as important regulators of gene expression, involving in both developmental and pathological processes. While altered expression of lncRNAs has been observed in breast cancer development, their roles in breast cancer progression and metastasis are still poorly understood.

**Methods:**

To identify novel breast cancer-associated lncRNA candidates, we employed a high-density SNP array-based approach to uncover intergenic lncRNA genes that are aberrantly expressed in breast cancer. We first evaluated the potential value as a breast cancer prognostic biomarker for one breast cancer-associated lncRNA, *LincIN*, using a breast cancer cohort retrieved from The Cancer Genome Atlas (TCGA) Data Portal. Then we characterized the role of *LincIN* in breast cancer progression and metastasis by in vitro invasion assay and a mouse tail vein injection metastasis model. To study the action of *LincIN*, we identified *LincIN*-interacting protein partner(s) by RNA pull-down experiments followed with protein identification by mass spectrometry.

**Results:**

High levels of *LincIN* expression are frequently observed in tumors compared to adjacent normal tissues, and are strongly associated with aggressive breast cancer. Importantly, analysis of TCGA data further suggest that high expression of *LincIN* is associated with poor overall survival in patients with breast cancer (*P* = 0.044 and *P* = 0.011 after adjustment for age). The functional experiments demonstrate that knockdown of *LincIN* inhibits tumor cell migration and invasion in vitro, which is supported by the results of transcriptome analysis in the *LincIN*-knockdown cells. Furthermore, knockdown of *LincIN* diminishes lung metastasis in a mouse tail vein injection model. We also identified a *LincIN*-binding protein, NF90, through which overexpression of *LincIN* may repress p21 protein expression by inhibiting its translation, and upregulation of p21 by *LincIN* knockdown may be associated with less aggressive metastasis phenotypes.

**Conclusions:**

Our studies provide clear evidence to support *LincIN* as a new regulator of tumor progression-metastasis at both transcriptional and translational levels and as a promising prognostic biomarker for breast cancer.

**Electronic supplementary material:**

The online version of this article (doi:10.1186/s13058-017-0853-2) contains supplementary material, which is available to authorized users.

## Background

Genome-wide profiling by deep sequencing and mapping distinctive chromatin signatures have identified thousands of long non-coding ribonucleic acid (lncRNA) species (>200 nt in length) [1–3]. A majority of these lncRNAs are derived from the intergenic genome and used to be considered as “transcriptional noise”. Now, some lncRNAs are recognized as novel regulators of gene expression, involving in both developmental and pathological processes [4–6]. Considerable attention has been garnered for the roles of lncRNAs in the development of cancer. Several well-characterized lncRNAs, such as *HOTAIR*, *FAL1*, *NKILA, LSINCT5*, and *BCAR4*, exhibit aberrant expression in cancer tissues and are associated with tumor progression and/or metastasis [7–11]. Hence, abnormal expression levels of these lncRNAs have potential clinical values as prognostic biomarkers, in addition to functional roles in promoting cancer [12]. In contrast to regulatory microRNAs, the functions of lncRNAs are not simply defined by a common mode of action, and each of them can act in a number of different ways, such as signals, decoys, guides or scaffolds [13]. In the nucleus, lncRNAs are primarily known for their interaction with chromatin modifiers, transcription factors or co-regulators, and DNA methylation enzymes, and, thus, epigenetically controlling gene transcription [14–16]. When present in the cytoplasm, lncRNAs are capable of regulating a number of post-transcriptional, translational and post-translational processes [17–19]. For example, the Half-staufen 1-binding site lncRNA (*1/2sbsRNAs*) is involved in staufen 1-mediated mRNA decay [18]. *NORAD* maintains genomic stability by sequestering PUMILIO proteins and regulates targeted mRNA stability and translation [19]. *LincRNA-p21* coordinates with RNA-binding protein HuR in the cytoplasm and modulates mRNA translation [20]. LncRNAs have also been found to directly regulate signal transduction at the post-translational level [9, 21]. For example, *Lnc-DC* expressed by dendritic cells promotes STAT6 phosphorylation and the activation of STAT6 signaling [21]. Although dysregulation of lncRNAs has been increasingly appreciated as a new “hallmark” of human cancer [22], the functional roles and regulatory mechanisms of many lncRNAs remain largely unknown, particularly for their co-actions with binding protein partners in these processes.

Nuclear factor 90 (NF90), a major spliced form of interleukin enhancer binding factor 3 (ILF3), was first identified on the basis of its ability to bind to the IL2 promoter in activated T cells [23], and it was subsequently found to bind double-stranded (ds) RNA structural elements [24]. Recent studies have shown that NF90 forms a complex with NF45 and plays multifunctional roles in the cells, including transcription, and microRNA biogenesis [25]. In addition to modulating transcription, NF90 is also capable of regulating gene expression at the post-transcriptional and translational levels [26–29]. However, the precise function of NF90 remains to be uncovered.

In the current investigation, we identified and characterized a novel breast cancer metastasis-associated lncRNA, a long intergenic non-coding RNA between ITGB1 and NRP1 (*LincIN*), by utilizing a high-density SNP array-based gene expression approach to evaluate the lncRNA transcriptome in paired normal versus tumor samples. *LincIN* is elevated in the majority of breast tumors and high levels of *LincIN* expression predict poor clinical outcomes. Our functional studies showed that *LincIN* plays a key role in breast cancer cell invasion and metastasis, interacts with NF90, and appears to regulate p21 expression at the translation level. Altogether, our studies provide evidence to support *LincIN* as a regulator in tumor cell invasion and a promising prognostic biomarker for breast cancer.

## Methods

### Biospecimens and a TCGA breast cancer cohort

Primary human mammary epithelial cells (HMECs) from breast tumors and matched adjacent non-tumor tissues were isolated and cultured as previously described [30]. For evaluating the expression of *LincIN* in clinical specimens by in situ analysis, a breast cancer tissue microarray (TMA) was prepared by the Biosample Core Facility of Fox Chase Cancer Center (FCCC). In addition, RNASeq reads per kilobase million (RPKM) values at the *LincIN* locus (reads falling into: chr10:3360887-3361048) as well as clinical and follow-up information were downloaded from The Cancer Genome Atlas (TCGA) Data Portal (https://tcga-data.nci.nih.gov) [31].

### Illumina HumanOmni5 quad BeadChip analysis

Genomic deoxyribonucleic acid (gDNA), RNAs and double-stranded cDNAs (ds-cDNA) from paired normal and tumor primary HEMCs were prepared as previously described [30]. gDNA (quantified by PicoGreen assay) and ds-cDNA samples were subjected to whole genome application and fragmentation prior to Illumina HumanOmni5-quad BeadChip hybridization (Additional file 1: Figure S1). gDNAs and ds-cDNAs from seven paired normal-tumor samples plus two technical replicates were analyzed in a total of 32 arrays. The data from five HMEC pairs were included for final analysis after two pairs were excluded by Illumina quality control. Data were analyzed using the Linear Models for Microarray (LIMMA) data package from R with modification (detailed in “Statistics”).

### Quantitative RT-PCR (RT-qPCR)

Quantitative PCR (qPCR) was performed using the ABI 7900HT system (Applied Biosystems, Foster City, CA, USA). TaqMan assays for *p21* ^*WAF1*^, *LincIN,* and *GAPDH* were designed and purchased from Applied Biosystems. In addition, the qPCR amplicon for each gene was cloned into the pCR4-TOPO vector (Invitrogen, Carlsbad, CA, USA). Linearized plasmids carrying respective gene amplicons were diluted and used for constructing standard curves for each gene. All cDNA samples calculated from 20 ng of total RNA per reaction were assayed in quadruplicate in 384 microwell plates.

### Microarray

Total RNA was isolated using TRIzol reagent (Invitrogen) and the quality of total RNA was assessed by an Agilent 2100 Bioanalyzer (Agilent Technologies, Santa Clara, CA, USA). A total of 250 ng of total RNA sample was labeled and hybridized to the Affymetrix Human Gene 2.0 ST Array according to the manufacturer’s instructions (Affymetrix, Santa Clara, CA, USA). Scanned microarray images were analyzed using the Affymetrix Gene Expression Console with the RMA (Robust Multi-array Average) normalization algorithm. Further statistical analyses were performed using BRB-ArrayTools [32].

### RNA ligase-mediated rapid amplification of cDNA ends (RLM-RACE)

5′ and 3′ RLM-RACE analysis was performed using the FirstChoice^®^ RLM-RACE Kit (Life Technologies, Carlsbad, CA, USA) as previously described [30]. For the 5′ end, first internal and nested primer sequences were as follows, ATAAAAAGGATAGATATTTATTTCTCTCACAC and AGAACTCCTGCCCCTCCCCTGT. For the 3′ end, internal and nested primer sequences were as follows, AGCAAAACCTGAAGCCCCAAAGAG and AATTCCCATGGAGGAAAGAG.

### RNA in situ hybridization (ISH)

RNA ISH was performed with the RNAscope® 2.0 HD formalin-fixed, paraffin-embedded (FFPE) Assay Kits [Advanced Cell Diagnostics (ACD), Newark, CA, USA] following the manufacturer’s user manual. *LincIN*- specific RNA probes were custom designed by ACD. RNA staining of individual cells was scored semi-quantitatively according to the manufacturer’s instructions with minor modifications. Briefly, the expression levels of *LincIN*, *dapB* (negative control) and *POLR2* (positive control) were scored manually by two independent observers using the slightly modified guidelines recommended by ACD: 0 (no staining or <1 dot/10 cell), 1 (1–10 dots/cell and few dot clusters) and 2 (>10 dots/cell and >10% of dots are in clusters).

### Cell lines

Non-tumorigenic mammary epithelial cell lines, MCF-10A and -10F, and human breast cancer cell lines, BT-20, HCC-1937, MCF-7, MDA-MB-231, SK-BR-3, T-47D, and ZR-75-1, were purchased from American Type Culture Collection (ATCC). Cell lines were maintained according to ATCC recommended medium at 37 °C in the presence of 5% CO_2_. MDA-MB-231luc was gifted by Dr. Jose Russo (FCCC). MCF10ADCIS and SUM225 cells were gifts from Dr. Fariba Behbod (University of Kansas Medical Center) and were maintained as previously described [33].

### Antibodies

The following primary antibodies were used: NF90 rabbit polyclonal antibody (1:10000, Cat# ab131004, Abcam, Cambridge, MA, USA), NF45 rabbit monoclonal antibody (1:1000, Cat# ab131004, Abcam), p21^WAF1^ mouse monoclonal antibody (1:200, Cat# OP64, Calbiochem, La Jolla, CA, USA), Keratin mouse monoclonal antibody (1:1000, Cat# ab8068, Abcam), vimentin mouse monoclonal antibody (1:1000, Cat# V5255, Sigma-Aldrich, St. Louis, MO, USA), and β-actin mouse monoclonal antibody (1:5000, Cat#A5316, Sigma-Aldrich). For secondary antibodies, ECL^™^ HRP-conjugated anti-mouse or anti-rabbit IgG (GE Healthcare, Chicago, IL, USA) were used. For pull-down western experiments, HRP-conjugated anti-rabbit heavy chain (Jackson ImmunoResearch Labs, West Grove, PA, USA) was used.

### Plasmid construction

For *LincIN* short hairpin RNAs (shRNAs), sense and antisense oligos were designed using the Whitehead Institute online tool (http://sirna.wi.mit.edu/) and synthesized by Integrated DNA Technologies (IDT) (Coralville, IA, USA). The oligos were then annealed prior to cloning into the Age *I* and EcoR *I* restriction enzyme sites of pMKO.1-GFP, which was purchased from Addgene (Cambridge, MA, USA). After screening for *LincIN* knockdown efficacy, two of the most efficient shRNA constructs were chosen from a total of seven shRNA designs (sequences listed in Additional file 1: Table S1). In addition, the full length of *LincIN* was synthesized by Genewiz, Inc. (Cambridge, MA, USA), and was cloned into the pRetroX-IRES-ZsGreen vector (Clontech, Mountain View, CA, USA) at the BamH *I* and Not *I* restriction enzyme sites.

### Dicer-substrate siRNA transfection

Dicer-substrate small interfering RNAs (siRNAs) for *LincIN* were designed and synthesized from IDT. Sequences for si*LincIN*.A and si*LincIN*.B were as follows: GGACAUUAUGCAAGGAGAUGGCATC (sense), GAUGCCAUCUCCUUGCAUAAUGUCCUU (antisense); and CACCCUGCCAGAUGUGUCUUGUUCC (sense) and GGAACAAGACACAUCUGGCAGGGUGUC (antisense). Predesigned NF90 siRNAs and scrambled controls (SC) were purchased from IDT. siRNAs were transfected into cells using Oligofectamine^™^ or Lipofectamine 3000 reagents (Life Technologies) according to the manufacturer’s instructions.

### Wound closure assay, invasion, and cell cycle analysis

After breast cancer cells were cultured in a monolayer and reached 90–95% confluence, a scratch wound was carried out by creating a linear cell-free region using sterilized pipette tips as described previously [34]. The progress of cell migration into the scratch was photographed every 24 hours using an inverted fluorescence microscope. The images are further analyzed quantitatively using National Institutes of Health ImageJ software. Invasion assay was performed using the BD BioCoat^™^ Matrigel^™^ Invasion Chamber (BD Biosciences, Franklin Lakes, NJ, USA) following the manufacturer’s instructions. The invaded cells were stained with 1% crystal violet and examined under a light microscope. Cell cycle assay was done using the Nuclear-ID^®^ Red Cell Cycle Kit (Enzo Life Sciences, Farmingdale, NY, USA).

### Mouse tail vein injection metastasis model and bioluminescent imaging analysis

All the animal protocols were approved by the Institutional Animal Care and Use Committee (IACUC) at FCCC. Stable lines expressing empty green fluorescent protein (GFP) vector or *LincIN* shRNAs were generated using MDA-MB-231-Luc-D3H1 cells. To establish a lung metastasis model, 2 × 10^6^ MDA-MB-231-Luc cells were injected intravenously (tail vein) into female SCID mice of 6–7 weeks old. Images were taken at multiple time points (days 1, 7, 14, 21, 28, and 43) after injections of tumor cells. Bioluminescence imaging of animals was performed using standard display methods (exposure time, 1-60s; binning 8; field of view 4; f/stop 1; open filter) on the IVIS® Spectrum system (PerkinElmer, Waltham, MA, USA). Total photon plux (photons/sec) was determined from the region-of-interest (ROI) using Living-Image (Xenogen, Hopkinton, MA, USA) analysis software.

### RNA-protein pull-down and mass spectrometry analysis

Full-length sense and antisense of *LincIN* or *LincIN* RNA fragments (1-320, 332-554, and 538-1031) were in vitro transcribed with the TranscriptAid T7 High Yield transcription Kit (Thermo Fisher Scientific, Waltham, MA, USA) and labeled with biotin using the Pierce™ RNA 3′End Desthiobiotinylation Kit (Thermo Fisher Scientific). The whole lysates from MDA-MB-231 cells were freshly prepared with the RNasin® Ribonuclease Inhibitor (Promega, Madison, WI, USA) and protease/phosphatase inhibitor cocktail (Roche, Basel, Switzerland). Pull-down experiments were performed using the Pierce™ Magnetic RNA-Protein Pull-Down Kit (Thermo Fisher Scientific) according to the manufacturer’s instructions. *LincIN*-associated proteins were eluted and resolved by gel electrophoresis followed by staining with the SilverQuest™ Silver Staining Kit (Life Technologies) or directly used for Western blotting. Protein bands of interest were excised, de-stained, and digested prior to analysis by LC-MS/MS using reverse phase capillary high-performance liquid chromatography (HPLC) with a Thermo Electron LTQ OrbiTrap XL mass spectrometer at The Wistar Institute.

### RNA immunoprecipitation (RIP) and Immunoprecipitation (IP)

RIP experiments were performed using the Magna RIP^™^ RNA-Binding Protein Immunoprecipitation Kit (EMD Millipore, Billerica, MA, USA) according to the manufacturer’s protocol. Briefly, fresh lysates from MDA-MB-231 cells were prepared using RIP lysis buffer containing a protease inhibitor cocktail and RNase inhibitor. Five μg of NF90 or control antibodies (anti-SNRNP70 and normal rabbit IgG) were captured by magnetic beads and incubated with 100 μL cell lysate for 8 h at 4 °C. The co-precipitated RNAs were extracted using protease K and phenol/chloroform precipitation. Precipitated RNAs and total RNAs (input controls) were treated with TURBO^™^ DNase (Life Technologies) prior to the reverse transcription with the iScript^™^ cDNA Synthesis Kit (Bio-Rad Laboratories, Hercules, CA, USA). Analysis of *LincIN* RIP signals were performed using a custom-designed TaqMan assay (Applied BioSystems) via real-time PCR. IP was performed using the Magnetic Dynabeads Kit (Thermo Fisher Scientific) according to the manufacturer’s manual.

### Statistical analysis

The LIMMA (Linear Models for Microarray Data) methodology [35, 36] was used to identify differentially expressed intergenic lncRNAs between paired tumor and normal samples. The Benjamini-Hochberg method was used to adjust for multiple testing and to calculate the false discovery rate (FDR) for each lncRNA probe [37]. Computations were performed using the R statistical language and environment [38]. The Wilcoxon signed-rank test was used to compare differences between paired tumor and normal samples for RNA-ISH and TCGA data; and Kaplan-Meier survival curves were compared using log-rank tests. Kruskal-Wallis and Fisher’s exact tests were used to test the association between clinical variables and *LincIN* expression levels. In vitro data was analyzed using one-way analysis of variance (ANOVA) and Dunnett’s test to account for multiple post hoc comparisons or the two-sample *t* test. All tests were two-sided and used a type I error of 5%. TCGA data was analyzed using SAS software, version 9.4 (SAS Institute Inc., Cary, NC, USA). If not mentioned specifically, all experiments were repeated in triplicate.

## Results

### Identification of a novel breast cancer-associated intergenic lncRNA, *LincIN*

**Fig. 1:**
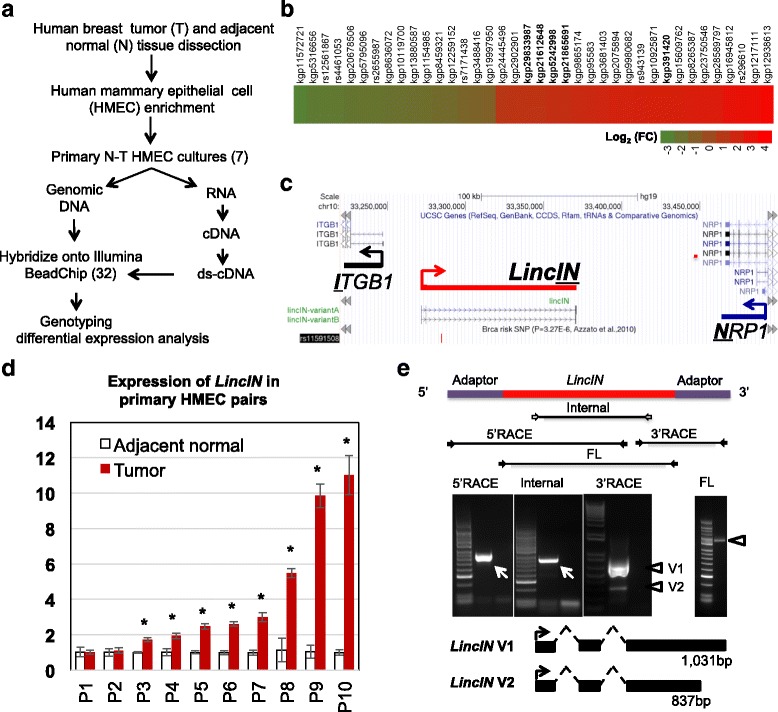
Identification and characterization of an intergenic lncRNA, *LincIN.* **a** Strategies for analyzing seven paired normal-tumor HEMC lines with high-density BeadChips (total 32 arrays) to identify differential expression in the intergenic lncRNA transcriptome. For each probe marker, the scanned raw signal intensities were processed by GenomeStudio software (Illumina, San Diego, CA, USA) to generate X and Y intensity values for allelic expression at each marker position. The total expression values for heterozygous markers were calculated using the sum of raw intensities from the X and Y channels. The total expression values for homozygous markers were assigned with either X or Y intensities based on the genotyping calls from corresponding gDNA signals. **b** The heatmap of top differentially expressed intergenic lncRNAs [FDR < 0.15, *P* < 0.005, |Log_2_ of fold change (FC)| >1]. Highlighted probes (in *bold*) were mapped to the *LincIN* locus*.* **c** The physical map and genomic organization of *LincIN* were illustrated using the annotation tracks created by the UCSC Genome Browser. **d** Validation of *LincIN* expression in ten HMEC normal-tumor pairs by RT-qPCR (^*^ *P* < 0.05, *t* test; technical replicates: n = 4). **e** Rapid amplification of cDNA ends (RACE) analysis of *LincIN* transcripts resulted in two RNA variants (V1:1031 bp and V2: 837 bp) and full length (FL) of V1 was obtained by RT-PCR

To identify novel breast cancer-associated lncRNA candidates at intergenic regions, we employed a high-density SNP array-based approach by specifically probing intergenic regions to uncover lncRNA genes, whose expression was altered in breast tumors (Fig. 1a). To minimize the confounding effect from admixed stromal cells, we enriched the epithelial cell population and investigated their differential expression patterns between invasive ductal carcinoma (IDC) tissues and adjacent normal breast tissues (Additional file 1: Figure S1A).

As a result, we identified 26 intergenic lncRNA transcripts that are dysregulated in tumors [*P* < 0.005, FDR < 0.15, |Log_2_ (fold change)| >1]. Among them, 14 lncRNAs (targeted by 22 SNP markers) were upregulated, and 12 lncRNAs (targeted by 16 SNP markers) were downregulated (Fig. 1b and Additional file 1: Table S2). One of the dysregulated intergenic lncRNAs, termed *LincIN* (GenBank access number: KX352723)*,* is located at Ch10p11-12 and between two coding genes, *I* *TGB1* and *N* *RP1* (Fig. 1c). Notably, data from five lncRNA exon-targeting probes (*kgp29833987, kgp21612648, kgp5242998, kgp21865691,* and *kgp391420*) showed that *LincIN* is significantly upregulated in breast tumors in comparison to the normal components (Log_2_FC = 1.3–2.3 and *P* < 0.005) (Fig. 1b and Additional file 1: Table S2). Further gene expression analysis by RT-qPCR demonstrated that *LincIN* is significantly upregulated in the majority of tumor HMEC lines in comparison to paired normal lines (8 out of 10, *P* < 0.05) (Fig. 1d).

To characterize the full-length transcript of *LincIN*, we performed 5′ and 3′ rapid amplification of cDNA ends (RACE) and identified two spliced *LincIN* RNA variants (Fig. 1e). Sequence analysis of *LincIN* transcripts revealed that *LincIN* RNA utilizes two poly(A) sites to generate a short transcript (837 bp) and a longer transcript (1031 bp) (Fig. 1e). The expression of the longer *LincIN* variant appears to be more abundant and thus is the focus of our functional analysis. In addition, cellular fractionation analysis revealed that *LincIN* is distributed in both the nucleus and the cytoplasm (Additional file 1: Figure S2). Lastly, the 3′ end of *LincIN* appears to be polyadenylated since *LincIN* is clearly detected in poly(A)- enriched RNA fractions (Additional file 1: Figure S2).

Based on a BLASTX analysis of all possible reading frames identified by the open reading frame (ORF) finder from the NCBI and ATGpr (http://atgpr.dbcls.jp/), *LincIN* lacks the potential to encode any recognizable protein domains. As described previously [19], we further examined the coding potential of *LincIN* using a bioinformatics tool, PhyloCSF, a comparative genomics method for distinguishing protein-coding and non-coding regions based on their evolutionary signatures characteristic to alignments of conserved coding regions [39]. This analysis confirmed the low coding potential of *LincIN*, which receives a maximum smoothed codon substitution frequency (CSF) value similar to other well-characterized lncRNAs (Additional file 1: Figure S3). These findings established *LincIN* as a new lncRNA, and we next investigated its role in breast cancer development.

### LincIN is overexpressed in advanced human breast tumors and is a promising breast cancer prognostic biomarker

**Fig. 2:**
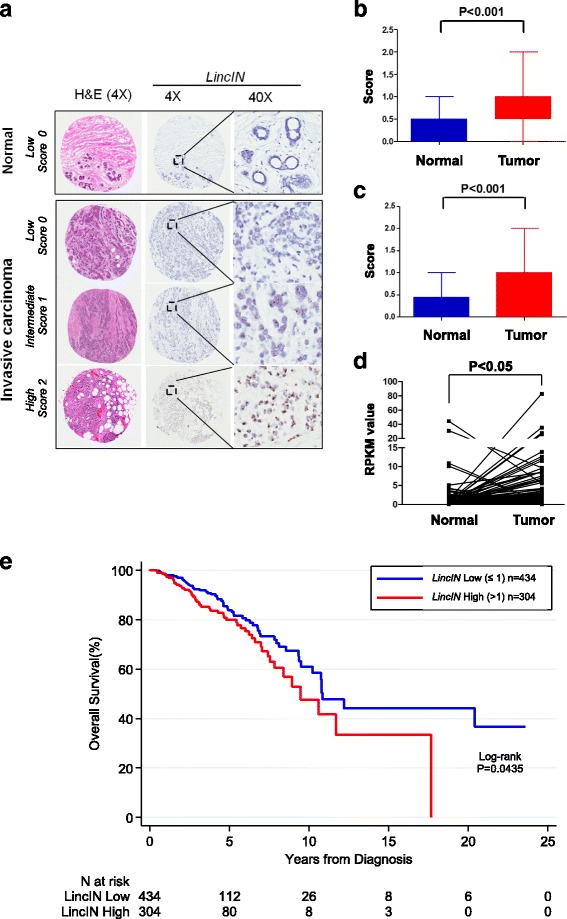
Expression of *LincIN* in clinical samples. **a** *LincIN* expression was evaluated by RNAcope^®^ ISH analysis on FFPE tissue microarrays (TMA) containing normal breast tissue and invasive breast carcinoma. To exclude low-quality tissue specimens due to potential RNA degradation, we simultaneously performed RNAScope^®^ ISH experiments in positive (*POLR2) and* negative *(dapB)* controls in serial TMA sections. **b** Analysis of RNA-ISH scores in 88 tumor and 31 normal samples using the two-sample Wilcoxon test. **c** Analysis of RNA ISH scores in 27 tumor and normal paired samples using the two-sample Wilcoxon test. **d** *LincIN* RNAseq expression data (RPKM value) for 88 normal-tumor pairs were retrieved from TCGA. The Wilcoxon signed-rank test was used for the comparison of matched pairs. **e** Overall survival (OS) from initial pathologic diagnosis was estimated using Kaplan-Meier methods, based on vital status and “days to death” or “days to last follow-up”. Individuals who were still alive at the time of last the follow-up were censored

Previously, one genome-wide association study (GWAS) identified a breast cancer survival variant, *rs11591508* (*P* = 3.27 × 10^-6^, hazard ratio = 2.41–3.29), which is located at the *LincIN* locus [40] (Fig. 1c). This finding suggested that *LincIN* may be a biomarker for breast cancer prognosis. Therefore, the expression of *LincIN* in breast tissues was evaluated initially by RNA *in situ* hybridization (RNAscope^®^) utilizing an in-house TMA, which contained a panel of breast normal (36) and tumor (103) specimens. After inspecting RNA quality with the *POLR2* and *dapB* staining, 20 samples with RNA degradation were excluded, which left the cohort with 88 tumors and 31 normal specimens including 27 tumor/normal pairs for final analysis. Figure 2a shows the representative RNAscope^®^ ISH images with different *LincIN* staining levels. Among all the specimens, *LincIN* exerts positive staining in approximately 72% of the breast tumor tissues (63 out of 88, score ranging from 1–2) while only approximately 29% of the normal tissues show positive *LincIN* expression (9 out of 31, score 1–2; *P* < 0.001) (Fig. 2b). Furthermore, *LincIN* levels are significantly higher in tumors comparing to those in matched adjacent normal tissues (*P* < 0.001) by Wilcoxon test (Fig. 2c). To further examine the potential value of *LincIN* as a breast cancer prognostic biomarker, we used a larger breast cohort retrieved from the TCGA Data Portal. After initial exclusions for replication and probe values of zero, there were expression data for 752 tumor specimens and 98 normal specimens. Among them, 88 normal samples were collected from matched breast tumor. Consistently, quantification of *LincIN* levels demonstrated significantly higher expression levels of *LincIN* in tumors versus matched adjacent normal tissues (*n* = 88) (4.28 vs.1.96, *P* = 0.018) (Fig. 2d). Notably, the levels of *LincIN* in breast tumors increase significantly with the pathologic stages defined by the American Joint Committee on Cancer (AJCC) or the tumor size (Table 1). For overall survival analysis, patients with follow-up days = 0 and pathologic tumor stage stated as “not available” were excluded, and 738 patients were included in the final survival analyses. Because of the skewed distribution of *LincIN*, we used categories based on *LincIN* levels rather than percentiles, with < =1, >1–5, >5–10, and >10–83 as the initial groups. Based on similarities in survival in the higher *LincIN* groups, the upper categories were combined into *LincIN* > 1. The survival analysis showed that patients expressing high levels of *LincIN* (>1.0) have worse survival outcomes (*P* = 0.044 and *P* = 0.011 after adjust for age) (Fig. 2e).

**Table 1:** TCGA, Comparison of *LincIN* expression values by pathologic stages

	N	Median (1st, 3rd quartiles)	Mean (Std Dev)	*P*-value*
Pathologic stage (AJCC)				*0.021*
I (incl stage I, IA, IB)	125	0.39 (0.22, 1.76)	2.06 (4.83)	
II (incl stage II, IIA, IIB)	432	0.54 (0.25, 3.75)	3.85 (8.34)	
III (incl stage III, IIIA, IIIB, IIIC)	165	0.68 (0.23, 5.09)	3.92 (6.32)	
IV (incl stage IV)	14	1.44 (0.28, 4.15)	4.33 (7.56)	
Pathologic stage (tumor size)				*0.0038*
T1, <2 cm (incl T1,T1a,T1b,T1c)	199	0.40 (0.22, 3.36)	2.95 (6.85)	
T2, 2-5 cm (incl T2, T2b)	443	0.54 (0.25, 4.04)	3.63 (7.10)	
T3, >5 cm (incl T3)	80	0.47 (0.21, 2.41)	3.76 (9.65)	
T4 (incl T4,T4b,T4d)	28	2.04 (0.63, 9.64)	6.02 (7.48)	

TCGA The Cancer Genome Atlas, *AJCC*: American Joint Committee on Cancer

^*^ *P* value is from Kruskal-Wallis test to compare *LincIN* distributions within pathologic stages defined by AJCC or tumor size. This nonparametric test was used instead of one-way ANOVA due to the skewed distribution of the *LincIN* expression values. The samples with pathologic information stated as ‘not available' were excluded from data analysis

### Role of LincIN in breast cancer cell invasion and metastasis

**Fig. 3:**
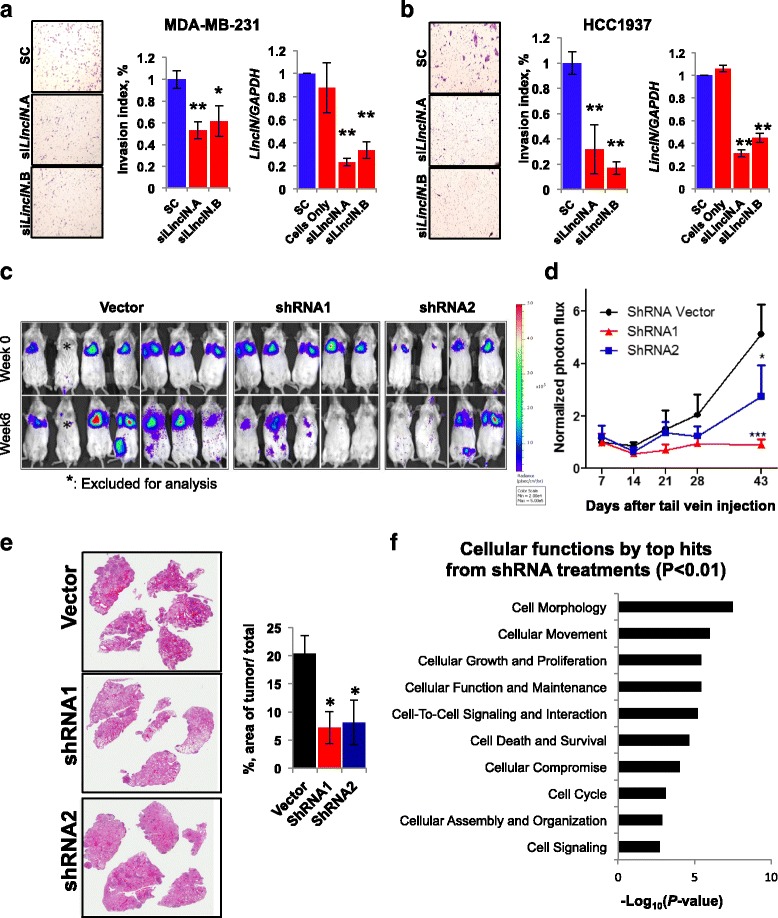
*LincIN* mediates breast cancer cell invasion and metastasis in vitro and in vivo. **a** Inhibitory effects of *LincIN* knockdown on MDA-MB-231 cell invasion. *Left* and *middle* panels: MDA-MB-231 cells were transfected with scrambled control (SC), *siLincIN*.A or *siLincIN*.B for 24 hours and seeded on Boyden chambers for in vitro invasion assays. Cell invasion capacity in si*LincIN*.A-, or si*LincIN*.B-treated MDA-MB-231 cells was compared to the SC group. *Right* panel: the RT-qPCR analysis of *LincIN* knockdown using dicer substrate si*LincIN*.A or si*LincIN*.B) versus SC or untreated cells. **b** Inhibitory effects of *LincIN* knockdown on HCC1937 cell invasion. **c** Bioluminescent imaging of mice harboring lung metastases after tail vein injection of MDA-MB-231luc cells stably expressing *LincIN* shRNAs or empty vector at week 0 and 6. **d** Bioluminescent quantification plot of lung metastasis by MDA-MB-231luc cells expressing shRNAs or control (empty vector) at day 7, 14, 21, 28, and 43. Data were combined from two independent experiments. **e** *Right* panel: representative whole slide imaging (×0.6) of dissected lung tissues from the tail vein injection mice carrying MDA-MB-231luc cells stably expressing *LincIN* shRNAs or empty vector [stained with hematoxylin and eosin (H&E), *left*]. *Left* panel: quantification of lung metastasis was performed using tumor area recorded per lung section against corresponding total lung section area (three mice per group). Data was analyzed using one-way ANOVA and the Dunnett’s test to account for multiple post hoc comparisons (^***^ *P* < 0.005, ^**^ *P* < 0.01, and ^*^ *P* < 0.05; images were taken at ×100). **f** Top cellular functions targeted by *LincIN* knockdown by shRNAs (*P* < 0.01)

As *LincIN* is frequently overexpressed in advanced breast tumors, we sought to explore the functional roles of *LincIN* in breast cancer progression-metastasis. Examining *LincIN* expression levels in a panel of 12 breast cell lines showed that *LincIN* is overexpressed up to 40-fold in highly metastatic MDA-MB-231 cells (ER-/PR-/HER2-), versus immortalized but non-transformed MCF10A cells (*P* < 0.001) (Additional file 1: Figure S4). We next evaluated the impact of *LincIN* loss on cell migration and invasion in MDA-MB-231 cells. Suppression of *LincIN* by RNA interference (RNAi) leads to an approximately 40–50% reduction of cell invasion compared to the control group (*P* < 0.05) (Fig. 3a). This inhibitory effect is even more profound in HCC1937 cells (high expression of *LincIN,* ER-/PR-/HER2-), where about 50–80% reduction is observed as the result of silencing *LincIN* (*P* < 0.01) (Fig. 3b). Knockdown of *LincIN* in MDA-MB-231 cells also significantly decreases cell migration about approximately 30% compared to those transfected with vector in the wound closure assay (Additional file 1: Figure S5A). In contrast, overexpression of *LincIN* in MCF10ADCIS cells (non-invasive breast cancer cells and low expression of *LincIN*) tended to accelerate cell migration (*P* < 0.05) (Additional file 1: Figure S5B). We also examined the role of *LincIN* in cell proliferation. As shown in Additional file 1: Figure S5C, overexpression of *LincIN* in MCF-10A and MCF10ADCIS cells increases cell proliferation moderately (*P* < 0.05). However, downregulation of *LincIN* in MDA-MB-231-luc cells has no significant effects on cell proliferation. Taken together, these data suggest that *LincIN* plays a role in breast tumor cell migration and invasion in vitro*,* while the effects of *LincIN* on cell proliferation may be cell type-specific*.*

Next, we injected MDA-MB-231luc cells via the tail vein into severely combined immunodeficiency (SCID) mice to test a possible role of *LincIN* in cancer cell metastasis. Strikingly, we found that knockdown of *LincIN* significantly decreases lung metastases compared to those in the vector control, as evaluated by bioluminescent signals (*P* < 0.005–0.05) (Fig. 3c and d). Furthermore, quantification of scanned images of whole lung tissues showed a significant decrease (approximately 60%) in the metastatic area in mice receiving shRNA-treated cells as compared to those of the vector control (*P* < 0.05) (Fig. 3e). In addition, we observed variations of the inhibitory effects on lung metastasis between shRNA1 and shRNA2 treatments. To exclude potential off-target effects, we performed transcriptome analysis in MDA-MB-231 cells treated with vector control, shRNA1 or shRNA2 in duplicate. As a result, we found 173 and 321 differentially expressed genes for shRNA1 and shRNA2 treatments, respectively, in comparison to the vector control (*P* < 0.001, Additional file 1: Table S3). Among them, 122 genes are overlapped (Additional file 1: Figure S6A), and the values of Log2 (FC) of overlapping “hits” are significantly correlated between the two shRNA groups (R2 = 0.9596, *P* < 0.0001 by Spearman’s rho test, Additional file 1: Figure S6B). Furthermore, biological process analysis of differentially expressed genes by Ingenuity Pathway Analysis (IPA) showed that over 97% of the identified pathways are overlapped (74 out of 75 or 76) for shRNA1 or shRNA2 treatments, respectively (*P* < 0.01, Additional file 1: Figure S6C and Additional file 1: Table S4). These results suggest that the variations from different shRNA effects are not accounted for by off-target effects. Notably, the very top cellular functions targeted by both shRNAs was cellular movement (P < 1.05 × 10^-6^ approximately 5.27 × 10^-3^), providing molecular insight for the role of *LincIN* in tumor cell invasion through the regulation of gene transcription (Fig. 3f). Collectively, our results suggest that *LincIN* is involved in breast cancer cell invasion in vivo and knockdown of *LincIN* in breast cancer cells may effectively inhibit the metastatic processes.

### LincIN interacts with the RNA-binding protein NF90

**Fig. 4:**
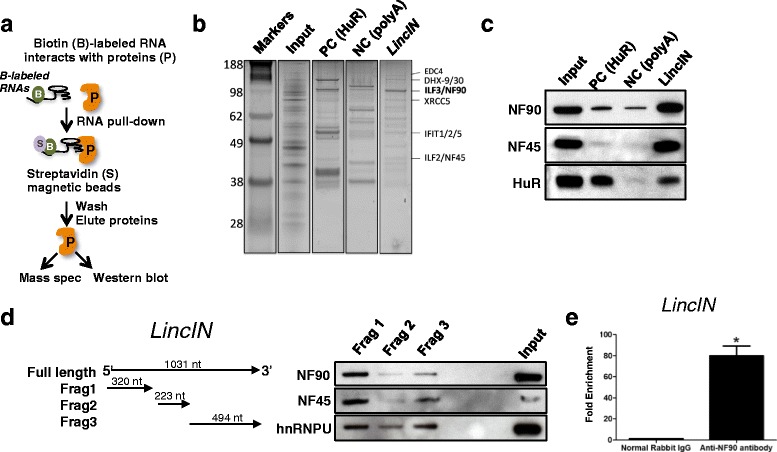
*LincIN* interacts with the NF90/NF45 complex. **a** Schematic flow of RNA pull-down experiments. **b** RNA pull-down was performed using the RNA-protein Pull Down Kit. Bands with arrows were submitted for mass spectrometric identification, and the most abundant band was identified as NF90/ILF3. Positive control (PC) and negative control (NC) were HuR and polyA as provided by the RNA pull-down kit, n = 4. **c** Western blot analysis was used to validate the specific association of NF90 with *LincIN* in pull-down lysates, repeated experiment. **d** *Left* panel: schematic diagram of *LincIN* fragments; *right* panel: Western blot analysis of NF90 in eluted protein samples pulled down by in vitro transcribed *LincIN* fragments. **e** RT-qPCR analysis of RNP samples (*right*) enriched by NF90 antibodies. RNP: RNA immunoprecipitation. Data was analyzed using *t* test (^**^ *P* < 0.01, and ^*^ *P* < 0.05)

To identify *LincIN*-interacting protein partner(s) that may contribute to its function in breast cancer development, we used in vitro transcribed biotin-labeled full-length *LincIN* for pull-down experiments followed with protein identification by mass spectrometry (MS) (Fig. 4a). The spectra counts were used to determine which proteins were unique/more abundant in a particular condition/gel band. Among the proteins identified by MS (Fig. 4b), NF90/ILF3 was the most abundant *LincIN*-binding partner. NF45/ILF2, which dimerizes with NF90 to form a functional complex, was also identified by MS as a *LincIN*-interacting protein. The *LincIN*-NF90 interaction was then validated by RNA pull-down Western blotting, and NF90 or NF45 were enriched in *LincIN*-pull-down cell lysates (Fig. 4c). To identify the critical regions of *LincIN* RNA required for NF90 binding, in vitro transcribed biotin-labeled truncated *LincIN* transcripts were also prepared for RNA pull-down experiments (Fig. 4d, left panel). Transcripts that contained the 5′ region of *LincIN* exhibited the strongest binding to NF90, while was weaker binding was observed in the 3′ and center regions (Fig. 4d, right panel). We further confirmed the interaction between *LincIN* and NF90 by performing RNA immunoprecipitation (RIP) with an antibody against NF90. As shown in Fig. 4e, *LincIN* RNA was enriched by approximately 40-fold in NF90 antibody precipitates. Altogether, RNA-pull-down and RIP experiments provided reciprocal evidence that *LincIN* directly interacts with NF90.

### LincIN regulates p21 protein expression at the translational level partially though the interactions with NF90

**Fig. 5:**
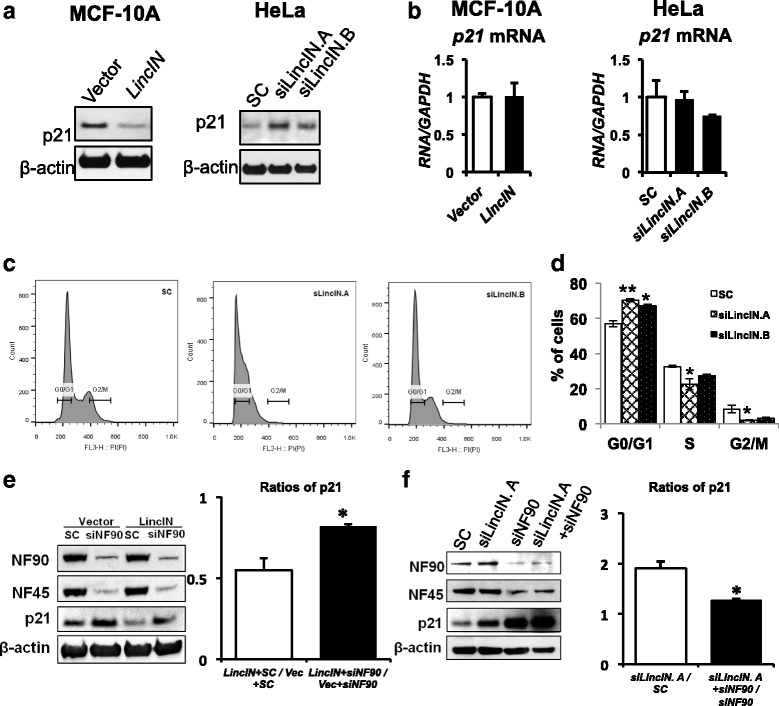
*LincIN* interacts with NF90 in the translation regulation of p21 expression. **a** *Left* panel: Western blot of p21 and beta-actin in vector and *LincIN* overexpressed MCF10A cells. *Right* panel: Western blot of p21, beta-actin or NF90, in SC and *LincIN* knockdown groups of HeLa cells. **b** RT-qPCR results of p21 in *LincIN* overexpression and vector control groups in MCF10A cells (*left*). RT-qPCR analysis of *LincIN* knockdown versus scrambled control in HeLa cells (*right*). (**c**) and (**d**) Cell cycle profiles and quantification of MDA-MB-231 cells treated with SC or *LincIN* siRNAs for 48 h (n = 3). Data was analyzed using one-way ANOVA and *t* test (^**^ *P* < 0.01, and ^*^ *P* < 0.05). **e** *Right* panel: MCF10A cells (vector or *LincIN*) were stably transfected with siRNA targeting NF90 or scrambled control. Cell lysates were collected at 48 h after transfection. *Left* panel: ratios of p21expression in *LincIN*-overexpression to vector control cells (*right* panel) (^*^ *P* < 0.05; *t* test). **f** *Right* panel: Western blot of NF90, NF45, and p21 in HeLa cells treated with SC, *LincIN* or NF90 siRNA alone or in combination. Cell lysates were collected at 48 h after transfection, repeated in triplicate. *Left* panel: ratios of p21expression in si*LincIN*-transfected to SC control cells (^*^ *P* < 0.05; *t* test)

Recent studies showed that NF90 is multifunctional in cells, including repressing p21 protein expression in cancer cells [28]. We next examined if *LincIN* plays a role in the NF90-mediated p21 pathway. Overexpression of *LincIN* in MCF10A cells diminishes p21 protein expression by approximately 40% (left panel, Fig. 5a,). Reciprocally, the level of p21 protein is consistently induced (approximately twofold) in *LincIN* knockdown groups compared to scrambled control (right panel, Fig. 5a). In contrast with altered protein expression, static *p21* mRNA levels remain unchanged in both *LincIN* overexpression and knockdown experiments (Fig. 5b), suggesting that *LincIN* regulates p21 at the translational level. Moreover, loss of *LincIN* results in increased G1/G0 arrest (*P* < 0.05) (Fig. 5c and d), which is a typical phenotype associated with the elevated expression of p21. In addition, we have evaluated the p21 level in the mouse lung metastasis colonies by immunohistochemistry (IHC), and the nuclear p21 levels are higher in the metastasis colonies from the *LincIN* shRNA knockdown group compared to those tissues from shRNA control group (*P* < 0.05, Additional file 1: Figure S7 and Additional file 1: Table S5). This finding suggests that upregulation of nuclear p21 by *LincIN* knockdown may be associated with less aggressive phenotype in our metastasis model.

Since recent studies showed that NF90 may regulate p21 protein expression at the translational level via binding its 3′ untranslated region (UTR) [29], we sought to determine if *LincIN* mediates p21 protein expression via NF90. We knocked down *LincIN*, NF90, or both using siRNAs and measured p21 expression by Western blotting. As expected, knockdown of NF90 upregulates p21 protein levels (approximately twofold) (right panel, Fig. 5e). Importantly, under the condition of NF90 knockdown, the effects of si*LincIN* on elevating p21 expression (evaluated by the ratio of si*LincIN* + siNF90/siNF90) in HeLa cells is decreased in comparison to the cells treated with si*LincIN* alone (the ratio of si*LincIN*/SC) (*P* < 0.05) (right panel, Fig. 5e). Furthermore, *LincIN* overexpression-induced p21 inhibition is also partially diminished by NF90 silencing in MCF-10A cells (Fig. 5f). Knockdown or overexpression of *LincIN* had no significant effects on the expression of NF90 (Fig. 5e and f). Taken together, these results suggest that *LincIN* may cooperate with NF90 to inhibit p21 translation.

## Discussion

**Fig. 6:**
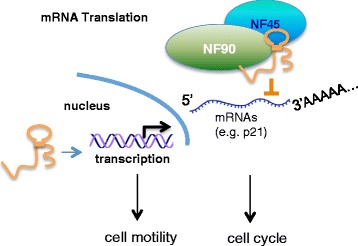
Schematic model for the role of *LincIN* in transcriptional and translational regulation

Functional characterization of individual lncRNAs has greatly extended our understanding of the complexity of the functional RNAs, which have been previously underappreciated, and it has also raised interest in determining underlying mechanisms. In the present study, we used high-density SNP arrays to explore the transcriptome of intergenic lncRNAs in breast cancer. We identified a new metastasis-associated lncRNA, *LincIN* (Fig. 1). High levels of *LincIN* expression are significantly associated with advanced breast cancer, and analysis of a large TCGA cohort suggested that *LincIN* is a promising prognostic biomarker for breast cancer (Fig. 2 and Table 1). Our in vitro and in vivo experiments demonstrated that *LincIN* may play an important role in tumor cell invasion and metastasis, and these findings are consistent with the results from transcriptome analysis in *LincIN*-knockdown cells (Fig. 3). We also identified a *LincIN*-binding protein, NF90, through which *LincIN* mediates p21 protein expression and cell cycle (Figs. 4 and 5). Our findings delineated a functional role of *LincIN* in breast tumor progression-metastasis, and mechanistically uncovered that it may regulate gene expression at both the transcriptional and translational levels (Fig. 6).

LncRNAs exhibit more tissue-, cell-type- and disease-specific patterns of expression [41], and this feature makes them a potentially precise biomarker for cancer diagnosis or prognosis. LncRNAs, thereby, are emerging as a new class of biomarkers for cancer, particularly for the later stage of cancer progression [12, 42, 43]. However, the potential for a majority of lncRNAs as biomarkers has not been fully explored in breast cancer. Here, we demonstrated that *LincIN* levels are consistently higher in tumors compared to those in adjacent normal tissues by evaluating two independent sample sets collected from the FCCC Biosample Repository and a breast cancer TCGA study (Fig. 2). Importantly, high levels of *LincIN* in breast tumors are correlated with advanced pathologic stages and a worse survival outcome, suggesting that *LincIN* is a promising prognostic biomarker for breast cancer and likely plays a functional role in breast tumor progression/metastasis.

A number of lncRNAs have been associated with breast cancer development. *HOTAIR*, the first lncRNA implicated in breast cancer, interacts with the PRC2 complex and LSD1 and drives transcriptional repression; overexpression of *HOTAIR* causes a global repression of tumor suppressors, which promotes breast cancer metastasis [7]. Oncogenic FAL1 binds and stabilizes epigenetic repressor BMI1, resulting in suppression of gene transcription, and then to malignant transformation and breast tumor growth [8]. NKILA inhibits breast cancer metastasis, specifically through binding and masking the IKB phosphorylation motif, and thus, preventing the activation of the NF-kB pathway [9]. Here, we demonstrate a critical role for *LincIN* in controlling breast cancer metastasis. Knockdown of *LincIN* in breast cancer cells diminished tumor cell invasion *in vitro* and it also reduced lung metastasis *in vivo* (Fig. 3). In this current study, we used a tail veil injection model to evaluate the role of *LincIN* in breast tumor metastasis. The tail vein injection metastasis model is generally considered as an experimental metastatic model - mimicking the late stage of metastasis when tumor cells are spreading and finding a metastatic niche. To further study the role of *LincIN* in tumor progression or early events of metastasis, orthotopic implantation to metastasis experiments should be considered in the future. Transcriptome analysis also showed that the very top cellular functions targeted by *LincIN*-knockdown are cellular movement, providing the molecular insights for the role of *LincIN* in tumor cell invasion (Fig. 3). Further investigations are needed to examine how *LincIN* mediates tumor progression and metastasis at the transcriptional level.

Mechanistic studies characterizing the lncRNA interactome have demonstrated that lncRNAs may serve as molecular scaffolds that connect or assemble multiple regulatory proteins and cooperatively control gene regulation [13]. Our results elucidate *LincIN* as a novel binding partner for NF90, which, together, inhibit p21 expression at the translational level and mediate cell cycle control (Figs. 4 and 5). NF90 was initially found to be essential for activating p21 expression in postnatal development since p21 was markedly reduced in NF90^-/-^ mice [29]. However, subsequent findings showed that p21 could also be upregulated in NF90-silenced viral-transfected HeLa cells [28], which were consistent with our data reported here. These findings suggest a bidirectional role of RNA-binding protein NF90 in regulating gene expression that is biological milieu-dependent. Such a bidirectional role has also been reported for other RNA-binding proteins, such as TARBP2 [44, 45]. These results suggest that NF90, previously believed to be a nonspecific double-stranded RNA-binding protein, may interact with specific partners, such as *LincIN,* and exert a specific functional role in certain biological milieu. Vumbaca et al. demonstrated that knockdown of NF90 decreased tumorigenesis and angiogenesis in an orthotropic breast tumor xenograft model [46]. As our results suggest, *LincIN* mediates protein translation (e.g., p21) through its interactions with NF90 (Fig. 6), and our IHC experiments show that the downregulation of *LincIN* knockdown significantly increases the level of nuclear p21 (*P < 0.05*, Additional file 1: Table S5 and Additional file 1: Figure S7B). Although the role of p21 in tumor invasion and metastasis is still not fully explored, nuclear p21 but not cytoplasmic p21 has favorable prognostic outcomes in breast and other cancers [47–49]. Our results suggest that the upregulation of nuclear p21 by *LincIN* knockdown may be associated with less aggressive metastasis phenotypes. Future studies examining how *LincIN* mediates tumor progression and metastasis through the NF90-mediated p21 pathway are warranted.

In our current study, we have demonstrated that *LincIN* is significantly upregulated in tumors versus normal samples. Importantly, knockdown of *LincIN* in breast cancer cells diminishes cancer cell migration and invasion in vitro, and reduced lung metastasis in a mouse tail vein injection model. These findings make *LincIN* a promising druggable target for preventing/treating breast cancer metastasis. The development of lncRNAs as a novel class of drug targets for breast cancer treatment is still at the very early stage, but these non-coding RNAs may hold the promise for new drug discovery [50]. Importantly, the identification of NF90, one of the functional *LincIN*-binding partners, provides an alternative strategy for drug development by targeting *LincIN*-NF90 interactions. As RNA-protein interactions (RPIs) are commonly present in functional RNAs, targeting RPIs using antisense oligos could provide a more efficient and specific approach than conventional RNAi technologies [51].

## Conclusions

Overall, we identified and characterized a novel breast cancer-associated lncRNA, *LincIN*, by evaluating the lncRNA transcriptome in paired normal versus tumor samples. Our demonstration of a correlation between breast patient survival outcomes and *LincIN* expression highlights its potential role as a prognostic biomarker. Our functional studies have established a potential role for *LincIN* in breast cancer progression/metastasis and revealed that its lncRNA-protein (e.g., NF90) interactions can be a novel approach for the regulation of protein expression. Since silencing of *LincIN* effectively reduced cancer cell metastasis, we propose that *LincIN* could potentially be a promising therapeutic target for the inhibition of progression of metastatic breast cancer.

## Additional file

Additional file 1: Supplementary figures and tables. (ZIP 4.69 mb)

## Abbreviations

AJCC: American Joint Committee on Cancer
ANOVA: Analysis of variance
ATCC: American Type Culture Collection
CSF: Codon substitution frequency
DNA: Deoxyribonucleic acid
FCCC: Fox Chase Cancer Center
FDA: Food and Drug Administration
FDR: False discovery rate
FFPE: Formalin-fixed, paraffin-embedded
GFP: Green fluorescent protein
GWAS: Genome-wide association studies
H&E: Hematoxylin and eosin stain
HMEC: Human mammary epithelial cells
HPLC: High-performance liquid chromatography
IDC: Invasive ductal carcinoma
IHC: Immunohistochemistry
IPA: Ingenuity^®^ Pathway Analysis
IRB: Institutional Review Board
LIMMA: Linear Models for Microarray
*LincIN*: Long intergenic non-coding RNA between ITGB1 and NRP1
lncRNA: Long non-coding RNA
MS: Mass spectrometry
NC: Negative control
NF90: Nuclear factor 90
PC: Positive control
qPCR: Quantitative polymerase chain reaction
RIP: RNA immunoprecipitation
RLM-RACE: RNA ligase-mediated rapid amplification of cDNA ends
RNA: Ribonucleic acid
RNA ISH: RNA in situ hybridization
RNAi: RNA interference
ROI: Region-of-interest
RPI: RNA-protein interactions
RPKM: Reads per kilobase million
RT-qPCR: Reverse transcription and quantitative PCR
SC: siRNA control
SCID: Severe combined immunodeficiency
shRNA: short hairpin RNA
siRNA: small interfering RNA
SNP: Single-nucleotide polymorphism
TCGA: The Cancer Genome Atlas
TMA: Tissue microarray
UTR: Untranslated region

## References

[1] Cabili MN, Trapnell C, Goff L, Koziol M, Tazon-Vega B, Regev A, Rinn JL (2011). Integrative annotation of human large intergenic noncoding RNAs reveals global properties and specific subclasses. Genes Dev..

[2] Derrien T, Johnson R, Bussotti G, Tanzer A, Djebali S, Tilgner H, Guernec G, Martin D, Merkel A, Knowles DG (2012). The GENCODE v7 catalog of human long noncoding RNAs: analysis of their gene structure, evolution, and expression. Genome Res.

[3] Guttman M, Amit I, Garber M, French C, Lin MF, Feldser D, Huarte M, Zuk O, Carey BW, Cassady JP (2009). Chromatin signature reveals over a thousand highly conserved large non-coding RNAs in mammals. Nature..

[4] Batista PJ, Chang HY (2013). Long noncoding RNAs: cellular address codes in development and disease. Cell..

[5] Fatica A, Bozzoni I (2014). Long non-coding RNAs: new players in cell differentiation and development. Nat Rev Genet.

[6] Li L, Chang HY (2014). Physiological roles of long noncoding RNAs: insight from knockout mice. Trends Cell Biol.

[7] Gupta RA, Shah N, Wang KC, Kim J, Horlings HM, Wong DJ, Tsai M-C, Hung T, Argani P, Rinn JL (2010). Long non-coding RNA HOTAIR reprograms chromatin state to promote cancer metastasis. Nature..

[8] Hu X, Feng Y, Zhang D, Zhao Sihai D, Hu Z, Greshock J, Zhang Y, Yang L, Zhong X, Wang L-P (2014). A Functional genomic approach identifies FAL1 as an oncogenic long noncoding RNA that associates with BMI1 and represses p21 expression in cancer. Cancer Cell..

[9] Liu B, Sun L, Liu Q, Gong C, Yao Y, Lv X, Lin L, Yao H, Su F, Li D (2015). A cytoplasmic NF-κB interacting long noncoding RNA blocks IκB phosphorylation and suppresses breast cancer metastasis. Cancer Cell..

[10] Silva JM, Boczek NJ, Berres MW, Ma X, Smith DI (2011). LSINCT5 is over expressed in breast and ovarian cancer and affects cellular proliferation. RNA Biol..

[11] Xing Z, Lin A, Li C, Liang K, Wang S, Liu Y, Park PK, Qin L, Wei Y, Hawke DH (2014). lncRNA directs cooperative epigenetic regulation downstream of chemokine signals. Cell.

[12] Yan X, Hu Z, Feng Y, Hu X, Yuan J, Zhao SD, Zhang Y, Yang L, Shan W, He Q (2015). Comprehensive genomic characterization of long non-coding RNAs across human cancers. Cancer Cell.

[13] Ulitsky I, Bartel DP (2013). lincRNAs: genomics, evolution, and mechanisms. Cell.

[14] Clemson CM, Hutchinson JN, Sara SA, Ensminger AW, Fox AH, Chess A, Lawrence JB (2009). An architectural role for a nuclear noncoding RNA: NEAT1 RNA is essential for the structure of paraspeckles. Mol Cell.

[15] Hacisuleyman E, Goff LA, Trapnell C, Williams A, Henao-Mejia J, Sun L, McClanahan P, Hendrickson DG, Sauvageau M, Kelley DR (2014). Topological organization of multichromosomal regions by the long intergenic noncoding RNA Firre. Nat Struct Mol Biol.

[16] Mercer TR, Mattick JS (2013). Structure and function of long noncoding RNAs in epigenetic regulation. Nat Struct Mol Biol..

[17] Faghihi MA, Modarresi F, Khalil AM, Wood DE, Sahagan BG, Morgan TE, Finch CE, St Laurent G, Kenny PJ, Wahlestedt C (2008). Expression of a noncoding RNA is elevated in Alzheimer’s disease and drives rapid feed-forward regulation of beta-secretase. Nat Med..

[18] Gong C, Maquat LE (2011). lncRNAs transactivate STAU1-mediated mRNA decay by duplexing with 3′ UTRs via Alu elements. Nature.

[19] Lee S, Kopp F, Chang TC, Sataluri A, Chen B, Sivakumar S, Yu H, Xie Y, Mendell JT (2016). Noncoding RNA NORAD regulates genomic stability by sequestering PUMILIO proteins. Cell.

[20] Yoon JH, Abdelmohsen K, Srikantan S, Yang X, Martindale JL, De S, Huarte M, Zhan M, Becker KG, Gorospe M (2012). LincRNA-p21 Suppresses Target mRNA Translation. Mol Cell..

[21] Wang P, Xue Y, Han Y, Lin L, Wu C, Xu S, Jiang Z, Xu J, Liu Q, Cao X (2014). The STAT3-binding long noncoding RNA lnc-DC controls human dendritic cell differentiation. Science.

[22] Gutschner T, Diederichs S (2012). The hallmarks of cancer: a long non-coding RNA point of view. RNA Biol..

[23] Kao PN, Chen L, Brock G, Ng J, Kenny J, Smith AJ, Corthésy B (1994). Cloning and expression of cyclosporin A- and FK506-sensitive nuclear factor of activated T-cells: NF45 and NF90. J Biol Chem..

[24] Castella S, Bernard R, Corno M, Fradin A, Larcher JC (2015). Ilf3 and NF90 functions in RNA biology. Wiley Interdiscip Rev RNA.

[25] Masuda K, Kuwano Y, Nishida K, Rokutan K, Imoto I (2013). NF90 in posttranscriptional gene regulation and microRNA biogenesis. Int J Mol Sci.

[26] Jiang W, Huang H, Ding L, Zhu P, Saiyin H, Ji G, Zuo J, Han D, Pan Y, Ding D (2015). Regulation of cell cycle of hepatocellular carcinoma by NF90 through modulation of cyclin E1 mRNA stability. Oncogene.

[27] Pullmann R, Kim HH, Abdelmohsen K, Lal A, Martindale JL, Yang X, Gorospe M (2007). Analysis of turnover and translation regulatory RNA-binding protein expression through binding to cognate mRNAs. Mol Cell Biol.

[28] Shamanna R, Hoque M, Pe’ery T, Mathews MB (2013). Induction of p53, p21 and apoptosis by silencing the NF90/NF45 complex in human papilloma virus-transformed cervical carcinoma cells. Oncogene..

[29] Shi L, Zhao G, Qiu D, Godfrey WR, Vogel H, Rando TA, Hu H, Kao PN (2005). NF90 regulates cell cycle exit and terminal myogenic differentiation by direct binding to the 3′-untranslated region of MyoD and p21 WAF1/CIP1 mRNAs. J Biol Chem..

[30] Gao C, Devarajan K, Zhou Y, Slater CM, Daly MB, Chen X (2012). Identifying breast cancer risk loci by global differential allele-specific expression (DASE) analysis in mammary epithelial transcriptome. BMC Genomics..

[31] Cancer Genome Atlas Network (2012). Comprehensive molecular portraits of human breast tumours. Nature.

[32] Simon R, Lam A, Li MC, Ngan M, Menenzes S, Zhao Y (2007). Analysis of gene expression data using BRB-ArrayTools. Cancer Inform..

[33] Behbod F, Kittrell FS, LaMarca H, Edwards D, Kerbawy S, Heestand JC, Young E, Mukhopadhyay P, Yeh HW, Allred DC (2009). An intraductal human-in-mouse transplantation model mimics the subtypes of ductal carcinoma in situ. Breast Cancer Res.

[34] Liang CC, Park AY, Guan JL (2007). In vitro scratch assay: a convenient and inexpensive method for analysis of cell migration in vitro. Nat Protoc.

[35] Gentleman RC, Carey VJ, Bates DM, Bolstad B, Dettling M, Dudoit S, Ellis B, Gautier L, Ge Y, Gentry J (2004). Bioconductor: open software development for computational biology and bioinformatics. Genome Biol.

[36] Smyth GK (2004). Linear models and empirical bayes methods for assessing differential expression in microarray experiments. Stat Appl Genet Mol Biol.

[37] Benjamini Y, Hochberg Y (1995). Controlling the False Discovery Rate: a practical and powerful approach to multiple testing. J R Stat Soc..

[38] R Development Core Team, R: a language and environment for statistical computing (2014). R: A language and environment for statistical computing.

[39] Lin MF, Jungreis I, Kellis M (2011). PhyloCSF: a comparative genomics method to distinguish protein coding and non-coding regions. Bioinformatics.

[40] Azzato EM, Pharoah PDP, Harrington P, Easton DF, Greenberg D, Caporaso NE, Chanock SJ, Hoover RN, Thomas G, Hunter DJ (2010). A genome-wide association study of prognosis in breast cancer. Cancer Epidemiol Biomark Prev..

[41] Brunner AL, Beck AH, Edris B, Sweeney RT, Zhu SX, Li R, Montgomery K, Varma S, Gilks T, Guo X (2012). Transcriptional profiling of long non-coding RNAs and novel transcribed regions across a diverse panel of archived human cancers. Genome Biol.

[42] Yang QQ, Deng YF (2014). Long non-coding RNAs as novel biomarkers and therapeutic targets in head and neck cancers. Int J Clin Exp Pathol.

[43] Yarmishyn AA, Kurochkin IV (2015). Long noncoding RNAs: a potential novel class of cancer biomarkers. Front Genet..

[44] Goodarzi H, Zhang S, Buss CG, Fish L, Tavazoie S, Tavazoie SF (2014). Metastasis-suppressor transcript destabilization through TARBP2 binding of mRNA hairpins. Nature.

[45] De Vito C, Riggi N, Cornaz S, Suvà ML, Baumer K, Provero P, Stamenkovic I (2012). A TARBP2-dDependent miRNA expression profile underlies cancer stem cell properties and provides candidate therapeutic reagents in Ewing sarcoma. Cancer Cell..

[46] Vumbaca F, Phoenix KN, Rodriguez-Pinto D, Han DK, Claffey KP (2008). Double-stranded RNA-binding protein regulates vascular endothelial growth factor mRNA stability, translation, and breast cancer angiogenesis. Mol Cell Biol.

[47] Cheng X, Xia W, Yang JY, Hsu JL, Chou CK, Sun HL, Wyszomierski SL, Mills GB, Muller WJ, Yu D (2010). Activation of p21(CIP1/WAF1) in mammary epithelium accelerates mammary tumorigenesis and promotes lung metastasis. Biochem Biophys Res Commun.

[48] Huang Y, Wang W, Chen Y, Huang Y, Zhang J, He S, Tan Y, Qiang F, Li A, Roe OD (2014). The opposite prognostic significance of nuclear and cytoplasmic p21 expression in resectable gastric cancer patients. J Gastroenterol.

[49] Winters ZE, Hunt NC, Bradburn MJ, Royds JA, Turley H, Harris AL, Norbury CJ (2001). Subcellular localisation of cyclin B, Cdc2 and p21(WAF1/CIP1) in breast cancer. association with prognosis. Eur J Cancer.

[50] Ling H, Fabbri M, Calin GA (2013). MicroRNAs and other non-coding RNAs as targets for anticancer drug development. Nat Rev Drug Discov.

[51] Bell NM, L'Hernault A, Murat P, Richards JE, Lever AM, Balasubramanian S (2013). Targeting RNA-protein interactions within the human immunodeficiency virus type 1 lifecycle. Biochemistry.

