# Supplementary material for: LincIN, a novel NF90-binding long non-coding RNA, is overexpressed in advanced breast tumors and involved in metastasis
Source: Breast Cancer Res. 2017 May 30;19:62. doi: 10.1186/s13058-017-0853-2 (PMC5450112; doi:10.1186/s13058-017-0853-2)
Supplement: Additional file 1: — Supplementary figures and tables. (ZIP 4.69 mb) [file 13058_2017_853_MOESM1_ESM.zip › Supplement Tables_0928.pdf]

Table S1, related to the Methods. Screening shRNA sequences for knockdown efficiency in transient transfection

No.	*LincIN* shRNA	Targeted sequence	% knockdown
1	sh401	GTGGACCTGGACCTGGATAAA	40.0
2	shsiA (shRNA2)	GACATTATGCAAGGAGATGGCA	68.6
3	sh324	AAAGGACATTATGCAAGGAGA	-0.4
4	sh420	AAATGGGTGTTTAACAAGTAT	17.1
5	sh681 (shRNA1)	CAGTTGGTCACTCTACTCAGT	75.8
6	sh614	GGATGTAGCCTCTCAGTGCTA	-0.9
7	sh767	GGGAGGAGAGTTAAATCTTCT	-0.5

Table S2. High dense array-based differential expression analysis of intergenic transcripts in breast tumors (T) vs. adjacent normal (N) (*: SNP probes targeting *LincIN* are highlighted in bold)

SNP Name	Chr	Coordinate	GeneSymbol	Ensembl ID	EnsemblExonID	ExonStart	ExonEnd	T vs. N (Log_2)	*P*-value	FDR
kgp10119700	8	101362845	RNF19A | ANKRD46	ENST00000519566	ENSE00002105796	101362511	101362935	-2.045248667	3.44E-05	0.041535179
rs296610	9	93763731	SYK | LOC100128909	ENST00000563268	ENSE00002600916	93763052	93764153	3.135225036	0.000901714	0.093018137
kgp12938613	11	12082124	DKK3 | MICAL2	ENST00000531559	ENSE00002186380	12080081	12083332	3.571358874	0.001003029	0.093018137
kgp28589797	8	49570001	LOC100289527 | EFCAB1	ENST00000518620	ENSE00002133004	49569820	49570248	2.53961768	0.001559584	0.093018137
kgp16945812	6	4494867	PECI | KU-MEL-3	ENST00000563079	ENSE00002597644	4494116	4496003	3.033355766	0.001740673	0.093018137
kgp15609762	1	20687903	VWA5B1 | CAMK2N1	ENST00000426428	ENSE00001636211	20687072	20688534	2.339099012	0.002304149	0.093018137
kgp23750546	7	112596586	C7orf60 | GPR85	ENST00000451962	ENSE00001676393	112596577	112596757	2.518496659	0.002521927	0.093018137
rs12561867	1	147801691	NBPF11 | LOC100132057	ENST00000452996	ENSE00001726117	147801651	147802209	-2.535435588	0.002631448	0.093018137
kgp20678506	4	174389186	SCRG1 | HAND2	ENST00000563631	ENSE00002606014	174388984	174391192	-2.243524967	0.003034708	0.093018137
rs2655987	14	77536732	C14orf4 | KIAA1737	ENST00000557526	ENSE00002498841	77535523	77537189	-2.193736021	0.003602533	0.093018137
kgp10925871	6	14282179	CD83 | JARID2	ENST00000427276	ENSE00001723124	14280358	14283419	2.306268253	0.003960234	0.093018137
kgp8636072	4	11477417	HS3ST1 | HSP90AB2P	ENST00000515343	ENSE00002070051	11476384	11479820	-2.129516795	0.004070573	0.093018137
kgp2075894	16	85319381	TMEM148 | KIAA0182	ENST00000366314	ENSE00001315394	85318604	85319569	2.160152458	0.004391908	0.093018137
kgp5316656	5	159912839	PTTG1 | ATP10B	ENST00000517927	ENSE00002103277	159912306	159914433	-2.591486451	0.00499737	0.093018137
rs1189827	14	57533464	LOC440180 | EXOC5	ENST00000554160	ENSE00002464555	57532978	57533886	2.337682103	0.005084419	0.093018137
rs7478802	11	12082861	DKK3 | MICAL2	ENST00000531559	ENSE00002186380	12080081	12083332	2.401696226	0.005107989	0.093018137
kgp28188898	14	57533821	LOC440180 | EXOC5	ENST00000554160	ENSE00002464555	57532978	57533886	2.102871791	0.00518167	0.093018137
rs7840059	8	29387821	LOC100132051 | C8orf75	ENST00000521101	ENSE00002099631	29387308	29387840	2.312145966	0.005363425	0.093018137
rs6655437		118469052	PGRMC1 | SLC25A43	ENST00000428222	ENSE00001764461	118467013	118469573	2.276622917	0.005581088	0.093018137
kgp10418412	14	21388431	LOC643332 | RNASE2	ENST00000555624	ENSE00002479155	21388166	21388458	-1.934018145	0.008168778	0.120686242
kgp2804462	22	27260683	MIAT | MN1	ENST00000434868	ENSE00001619874	27258220	27261749	-2.028311559	0.008465985	0.120686242
rs1878973	2	235131943	SPP2 | LOC100287159	ENST00000565664	ENSE00002619699	235131482	235132585	2.002505307	0.00847931	0.120686242
kgp18173532	3	20431041	SGOL1 | VENTXP7	ENST00000566804	ENSE00002605809	20429741	20432054	-1.911317449	0.008850324	0.120686242
rs1877351	11	112658438	LOC100132686 | NCAM1	ENST00000524772	ENSE00002183345	112658027	112658445	-2.200989498	0.009760572	0.12731181
kgp15201121	1	117008922	C1orf203 | CD58	ENST00000437308	ENSE00001641143	117008812	117009064	-1.821747475	0.010680388	0.133504846
rs4461053	15	101401592	LOC440313 | LOC145757	ENST00000431060	ENSE00001634368	101401151	101404501	-2.476627161	0.00026308	0.135793795
rs943139	1	153163377	SPRR2G | LELP1	ENST00000427145	ENSE00001706753	153162555	153164152	2.232827104	0.000364619	0.135793795
**kgp21865691***	**10**	**33370799**	**ITGB1 | NRP1**	**ENST00000450890**	**ENSE00001755916**	**33370270**	**33371030**	**1.555353877**	**0.000450021**	**0.135793795**
**kgp391420**	**10**	**33370289**	**ITGB1 | NRP1**	**ENST00000450890**	**ENSE00001755916**	**33370270**	**33371030**	**2.308419432**	**0.000760084**	**0.136655049**
kgp5795096	2	47567821	BCYRN1 | EPCAM	ENST00000418539	ENSE00001641977	47558199	47571656	-2.194324647	0.00151425	0.136655049
kgp1217111	11	12082595	DKK3 | MICAL2	ENST00000531559	ENSE00002186380	12080081	12083332	3.182924974	0.001646622	0.136655049
kgp13880587	17	70089562	LOC124685 | SOX9	ENST00000419257	ENSE00001783638	70089071	70089622	-1.9531175	0.00222606	0.136655049
kgp8265387	11	12082494	DKK3 | MICAL2	ENST00000531559	ENSE00002186380	12080081	12083332	2.444822027	0.002856578	0.136655049
kgp8459321	22	27255148	MIAT | MN1	ENST00000434868	ENSE00001656822	27254597	27256985	-1.739378916	0.002926665	0.136655049
kgp1154985	2	8702596	LOC339788 | ID2	ENST00000454224	ENSE00001713117	8699963	8708081	-1.89048152	0.003424679	0.136655049
kgp9980682	11	12082870	DKK3 | MICAL2	ENST00000531559	ENSE00002186380	12080081	12083332	2.217274552	0.003674317	0.136655049
kgp12259152	9	132045217	IER5L | C9orf106	ENST00000455981	ENSE00001596735	132045209	132045337	-1.710057217	0.003726956	0.136655049
**kgp5242998**	**10**	**33370878**	**ITGB1 | NRP1**	**ENST00000450890**	**ENSE00001755916**	**33370270**	**33371030**	**1.520726635**	**0.000971717**	**0.14589802**
kgp95583	1	153163029	SPRR2G | LELP1	ENST00000427145	ENSE00001706753	153162555	153164152	1.854468458	0.001055001	0.14589802
**kgp29833987**	**10**	**33370380**	**ITGB1 | NRP1**	**ENST00000450890**	**ENSE00001755916**	**33370270**	**33371030**	**1.291864391**	**0.001131027**	**0.14589802**
kgp9865174	12	60489522	SLC16A7 | LOC100289417	ENST00000552378	ENSE00002411934	60488910	60489632	1.791688154	0.001176635	0.14589802
**kgp21612648**	**10**	**33370735**	**ITGB1 | NRP1**	**ENST00000450890**	**ENSE00001755916**	**33370270**	**33371030**	**1.303201379**	**0.001417262**	**0.14589802**
kgp2902901	1	234765416	IRF2BP2 | PP2672	ENST00000429269	ENSE00001620425	234765057	234765493	1.238394919	0.001474133	0.14589802
kgp24445496	2	227045360	KIAA1486 | IRS1	ENST00000423838	ENSE00001659923	227044760	227045841	1.107691771	0.001492852	0.14589802
kgp3488416	15	101401408	LOC440313 | LOC145757	ENST00000431060	ENSE00001634368	101401151	101404501	-1.320725842	0.001514042	0.14589802
kgp3681403	12	60489264	SLC16A7 | LOC100289417	ENST00000552378	ENSE00002411934	60488910	60489632	1.999100055	0.00158928	0.14589802
kgp19997950	15	101401561	LOC440313 | LOC145757	ENST00000431060	ENSE00001634368	101401151	101404501	-1.189333273	0.001692272	0.14589802
rs3740219	10	9801095	GATA3 | SFTA1P	ENST00000419836	ENSE00001606593	9801042	9801200	1.795718899	0.012285084	0.146718472
rs1265242	6	4495016	PECI | KU-MEL-3	ENST00000563079	ENSE00002597644	4494116	4496003	1.944001855	0.012715601	0.146718472
rs7171438	15	101401700	LOC440313 | LOC145757	ENST00000431060	ENSE00001634368	101401151	101404501	-1.368349531	0.001915809	0.148780769
kgp11572721	15	63723317	CA12 | USP3	ENST00000559379	ENSE00002540116	63723256	63723541	-2.947660607	0.001972239	0.148780769
rs11781405	8	29702895	C8orf75 | LOC286135	ENST00000517356	ENSE00002111326	29702830	29702967	1.489784114	0.002155889	0.153068138
kgp15043032	22	27255167	MIAT | MN1	ENST00000434868	ENSE00001656822	27254597	27256985	-1.757054071	0.013901164	0.154457381
rs7273419	20	19789895	LOC100287166 | RIN2	ENST00000426012	ENSE00001700234	19789818	19790319	-1.222571683	0.002334499	0.156541098
kgp24677158	22	27259915	MIAT | MN1	ENST00000434868	ENSE00001619874	27258220	27261749	-1.718868502	0.014950181	0.160180512
kgp21808005	10	4698346	LOC100216001 | LOC33858	ENST00000417883	ENSE00001790831	4697619	4698512	1.220573801	0.002664681	0.163486218
kgp19918262	15	39481073	LOC100289563 | C15orf54	ENST00000560743	ENSE00002543139	39480639	39482367	1.808176476	0.002708968	0.163486218
rs2829750	21	26825565	NCRNA00158 | MIRHG2	ENST00000489205	ENSE00001894031	26825507	26825738	1.741044124	0.005510544	0.164802364
kgp5148979	2	9256821	MBOAT2 | ASAP2	ENST00000492300	ENSE00001828605	9256702	9257013	1.808931864	0.006299826	0.164802364
rs11022141	11	12082627	DKK3 | MICAL2	ENST00000531559	ENSE00002186380	12080081	12083332	2.283716744	0.006451007	0.164802364
rs10207811	2	208133613	KLF7 | CREB1	ENST00000438824	ENSE00001609347	208133425	208133617	1.607660109	0.006654897	0.164802364
kgp20709191	4	174390170	SCRG1 | HAND2	ENST00000563631	ENSE00002606014	174388984	174391192	-1.44955219	0.008874387	0.164802364
kgp6469327	8	29672701	C8orf75 | LOC286135	ENST00000517356	ENSE00002042927	29672520	29673808	1.438293148	0.00899267	0.164802364
kgp2030299	14	70924072	SYNJ2BP | ADAM21	ENST00000554551	ENSE00002473314	70924066	70924555	-1.793811051	0.009689783	0.164802364
rs10483130	22	27260639	MIAT | MN1	ENST00000434868	ENSE00001619874	27258220	27261749	-1.430847872	0.010794795	0.164802364
kgp15605984	1	87819491	LMO4 | PKN2	ENST00000452509	ENSE00001726170	87819207	87819550	-1.669727973	0.010922459	0.164802364
kgp30452030	5	87587207	TMEM161B | LOC645323	ENST00000501715	ENSE00001602037	87587201	87587293	-1.725966629	0.011374592	0.164802364
rs8017390	14	41444755	FBXO33 | LRFN5	ENST00000515218	ENSE00002067081	41444546	41445082	-1.766138004	0.011943855	0.164802364
rs292034	4	28478119	STIM2 | PCDH7	ENST00000509416	ENSE00002074495	28478072	28478131	1.73202552	0.012217986	0.164802364
rs4718532	7	66805000	STAG3L4 | AUTS2	ENST00000430244	ENSE00001624121	66804784	66805011	-1.373101968	0.012464608	0.164802364
rs927663	1	158102110	KIRREL | CD1D	ENST00000442358	ENSE00001705002	158101834	158102191	2.351140277	0.013498806	0.164802364
kgp5140573	20	25989429	FAM182B | LOC100134868	ENST00000448580	ENSE00001605646	25989427	25989691	-2.119401934	0.01360702	0.164802364
kgp3837811	12	89413399	KITLG | DUSP6	ENST00000500381	ENSE00001978090	89413330	89413456	1.279171936	0.014059995	0.164802364
rs10492195	12	16940720	LMO3 | LOC728622	ENST00000539266	ENSE00002241474	16940719	16940775	-1.840040124	0.014223379	0.164802364
kgp8532331	6	86096993	LOC100289423 | NT5E	ENST00000455071	ENSE00001534969	86096937	86099275	1.480706437	0.014252869	0.164802364
kgp9739214	13	21886630	LOC650794 | ZDHHC20	ENST00000434601	ENSE00001594917	21886590	21886694	-1.511871702	0.014467157	0.164802364
kgp30910771		115828094	CXorf61 | LOC653155	ENST00000446495	ENSE00001730486	115827969	115828156	-2.060135281	0.014482632	0.164802364
rs1369970	4	142246430	ZNF330 | LOC100286983	ENST00000509161	ENSE00002080359	142244576	142247161	-1.820010933	0.015940047	0.164897035
kgp22903170	11	112666519	LOC100132686 | NCAM1	ENST00000524772	ENSE00002165437	112666165	112666577	-2.027538334	0.003215481	0.1728902
kgp17327493	6	86097328	LOC100289423 | NT5E	ENST00000455071	ENSE00001534969	86096937	86099275	1.572931773	0.003272475	0.1728902
rs34667785	8	29679420	C8orf75 | LOC286135	ENST00000507496	ENSE00002071778	29678946	29679430	1.701222359	0.003294511	0.1728902

rs4848692	2	121940001	GLI2 | TFCP2L1	ENST00000432984	ENSE00001790363	121939674	121940156	-1.35762551	0.016850468	0.184886427
rs10734897	12	16848162	LMO3 | LOC728622	ENST00000418574	ENSE00001540498	16846734	16848407	-1.465219857	0.018440516	0.184886427
rs358994	3	8406919	GRM7 | LOC100288428	ENST00000435368	ENSE00001615003	8406393	8407062	-1.627164821	0.018504423	0.184886427
kgp22206609	5	148489210	SH3TC2 | ABLIM3	ENST00000509139	ENSE00002059570	148489026	148489350	-1.454790777	0.019194931	0.184886427
kgp10841095	3	59365858	C3orf67 | LOC339902	ENST00000497258	ENSE00001859002	59365750	59366309	1.213441614	0.019410112	0.184886427
rs3096021	5	159913603	PTTG1 | ATP10B	ENST00000517927	ENSE00002103277	159912306	159914433	-1.738512468	0.019609167	0.184886427
kgp15769169	1	209552880	PLXNA2 | LOC642587	ENST00000443636	ENSE00001637064	209552772	209553035	1.514093999	0.0037666	0.186772937
rs10106201	8	135809634	ZFAT | LOC286094	ENST00000568248	ENSE00002623781	135804263	135810515	-1.343456907	0.004049815	0.186772937
rs16981140	20	19789819	LOC100287166 | RIN2	ENST00000426012	ENSE00001700234	19789818	19790319	-1.04353681	0.004150106	0.186772937
kgp28240022	14	63590209	KCNH5 | RHOJ	ENST00000554921	ENSE00002440324	63589751	63590478	1.044799678	0.00419791	0.186772937
kgp1351230	12	1615702	ERC1 | FBXL14	ENST00000515614	ENSE00002050114	1615325	1616484	0.876052262	0.004351825	0.186772937
kgp17206651	6	86099238	LOC100289423 | NT5E	ENST00000455071	ENSE00001534969	86096937	86099275	1.629168927	0.004664995	0.186772937
kgp5192641	15	101402677	LOC145757 | ALDH1A3	ENST00000431060	ENSE00001634368	101401151	101404501	-1.011451735	0.004892666	0.186772937
kgp2218499	11	12094474	DKK3 | MICAL2	ENST00000476130	ENSE00002161996	12093738	12094561	1.930381197	0.004927925	0.186772937
kgp20001589	15	101401627	LOC440313 | LOC145757	ENST00000431060	ENSE00001634368	101401151	101404501	-3.004412445	0.004951727	0.186772937
kgp11188776	15	83380801	LOC338963 | SCARNA15	ENST00000559535	ENSE00002567720	83380428	83381059	-1.705947987	0.018768378	0.187304675
kgp5362706	6	4494738	PECI | KU-MEL-3	ENST00000563079	ENSE00002597644	4494116	4496003	1.807303408	0.019441691	0.187304675
P1_M_061510_2	22	37750028	CYTH4 | ELFN2	ENST00000445088	ENSE00001716394	37749166	37750879	1.652570676	0.019979165	0.187304675
kgp3193478	5	114541887	TRIM36 | PGGT1B	ENST00000507241	ENSE00002051389	114539713	114541943	-1.55858365	0.021353641	0.195741708
kgp28954431	8	135809943	ZFAT | LOC286094	ENST00000568248	ENSE00002623781	135804263	135810515	-1.231092058	0.022970677	0.196490735
kgp9980003	22	27260138	MIAT | MN1	ENST00000434868	ENSE00001619874	27258220	27261749	-1.144045484	0.02302917	0.196490735
kgp11614861	3	44162281	ABHD5 | LOC375337	ENST00000568686	ENSE00002586185	44158791	44163857	1.800346072	0.023221632	0.196490735
rs284826	21	19397062	NCRNA00157 | CHODL	ENST00000432412	ENSE00001631862	19396997	19397067	-1.519316596	0.023847311	0.196740311
kgp9414003	3	149796705	TMEM183B | TSC22D2	ENST00000464673	ENSE00001870543	149795281	149797168	1.799071306	0.005469968	0.200068227
kgp15472411	1	234765083	IRF2BP2 | PP2672	ENST00000429269	ENSE00001620425	234765057	234765493	1.141439982	0.025020374	0.2013835
kgp298117	1	23613063	HTR1D | HNRNPR	ENST00000566551	ENSE00002595853	23607802	23613245	-2.28071209	0.006000533	0.208514251
rs16866875	2	227045239	KIAA1486 | IRS1	ENST00000423838	ENSE00001659923	227044760	227045841	1.146355748	0.006054501	0.208514251
kgp10683194	5	33011467	C5orf23 | TARS	ENST00000511840	ENSE00002058225	33011428	33011666	1.10630531	0.006219149	0.208514251
kgp1836189	3	149796437	TMEM183B | TSC22D2	ENST00000464673	ENSE00001870543	149795281	149797168	1.179853952	0.027974203	0.213729189
kgp12988648	11	34595521	ELF5 | EHF	ENST00000527135	ENSE00002160386	34595094	34595529	-1.142567022	0.02821073	0.213729189
kgp10723230	22	27261049	MIAT | MN1	ENST00000434868	ENSE00001619874	27258220	27261749	-1.274675357	0.028497225	0.213729189
rs11638941	15	39481950	LOC100289563 | C15orf54	ENST00000560743	ENSE00002543139	39480639	39482367	1.942420925	0.006778515	0.217861163
kgp19744351	15	69760660	RPLP1 | LOC145837	ENST00000560882	ENSE00002551439	69760512	69760732	-1.385650177	0.006858926	0.217861163
kgp21951608	10	6628291	LOC439949 | SFMBT2	ENST00000561822	ENSE00002589425	6628186	6628882	0.79934684	0.007197427	0.222751135
kgp19867990	15	39481757	LOC100289563 | C15orf54	ENST00000560743	ENSE00002543139	39480639	39482367	1.609221665	0.007687699	0.226882838
kgp12383819	4	171197775	AADAT | LOC441052	ENST00000504509	ENSE00002074740	171197733	171197826	0.972684429	0.007706874	0.226882838
kgp7408566	2	221662520	SLC4A3 | EPHA4	ENST00000424395	ENSE00001644598	221662181	221662613	1.800674946	0.025354821	0.229534839
kgp27622817	12	65938237	MSRB3 | RPSAP52	ENST00000511935	ENSE00002027971	65938018	65938271	2.177091204	0.026013948	0.229534839
kgp1897448	15	72758104	LOC100130579 | ARIH1	ENST00000568345	ENSE00002623050	72757469	72758603	-0.959168161	0.008104192	0.232899046
kgp11437032	18	12775308	PSMG2 | PTPN2	ENST00000563722	ENSE00002613997	12774650	12775922	-1.453249776	0.032236655	0.236402137
rs7842777	8	10335032	MSRA | LOC346702	ENST00000520494	ENSE00002093248	10335001	10335291	-1.590276363	0.029314091	0.241212187
kgp18936391	12	65952039	MSRB3 | RPSAP52	ENST00000546198	ENSE00002266124	65951914	65952594	1.508028319	0.029765741	0.241212187
kgp27097887	3	20429867	SGOL1 | VENTXP7	ENST00000566804	ENSE00002605809	20429741	20432054	-1.597186214	0.030565684	0.241212187
kgp15238766	1	153163393	SPRR2G | LELP1	ENST00000427145	ENSE00001706753	153162655	153164152	1.543347586	0.031037983	0.241212187
kgp343459	2	231559416	SP100 | CAB39	ENST00000415174	ENSE00001532135	231559266	231559961	1.467519281	0.032446047	0.241212187
rs12216831	8	125847357	MTSS1 | LOC157381	ENST00000533496	ENSE00002167542	125847126	125847385	1.562720306	0.032604461	0.241212187
kgp9820410	4	142246106	ZNF330 | LOC100286983	ENST00000509161	ENSE00002080359	142244576	142247161	-1.6395808	0.032965666	0.241212187
kgp16460548	16	1029750	LMF1 | LOC100287129	ENST00000568394	ENSE00002581746	1029140	1031590	-1.891572124	0.034092394	0.244575871
kgp8109853	14	77534098	C14orf4 | KIAA1737	ENST00000500215	ENSE00001980448	77533452	77535846	-1.710594977	0.034987235	0.248405374
kgp7778226	22	27302631	MIAT | MN1	ENST00000447149	ENSE00001698602	27302541	27302730	-1.502248376	0.036410958	0.248405374
kgp8223382	5	103820340	NUDT12 | RAB9P1	ENST00000503650	ENSE00002041125	103819905	103820353	1.565691479	0.036432788	0.248405374
kgp19629373	14	77534214	C14orf4 | KIAA1737	ENST00000500215	ENSE00001980448	77533452	77535846	-1.109528705	0.008909636	0.250091417
kgp995924	1	185626194	LOC100288079 | HMCN1	ENST00000569292	ENSE00002629872	185625609	185626300	1.387281167	0.009569766	0.254735375
kgp15464389	1	234770330	IRF2BP2 | PP2672	ENST00000429269	ENSE00001771955	234768694	234770526	1.050965087	0.009856195	0.254735375
kgp10310964	13	73987741	KLF5 | LOC647281	ENST00000443621	ENSE00001781497	73987610	73987773	-1.510445726	0.009962044	0.254735375
rs11794563	9	132044938	IER5L | C9orf106	ENST00000455981	ENSE00001702211	132044737	132044971	-1.420813826	0.010043882	0.254735375
kgp207074	1	234770462	IRF2BP2 | PP2672	ENST00000429269	ENSE00001771955	234768694	234770526	1.08515317	0.010130322	0.254735375
kgp19923777	15	39482182	LOC100289563 | C15orf54	ENST00000560743	ENSE00002543139	39480639	39482367	1.896972305	0.011171173	0.265113513
rs1321991	1	185625971	LOC100288079 | HMCN1	ENST00000569292	ENSE00002629872	185625609	185626300	1.572725928	0.011215597	0.265113513
kgp906968	1	23612144	HTR1D | HNRNPR	ENST00000566551	ENSE00002595853	23607802	23613245	-1.46755886	0.011276754	0.265113513
kgp7930055	16	81993974	PLCG2 | SDR42E1	ENST00000564138	ENSE00002630315	81993524	81996298	-1.797639125	0.011551053	0.265113513
rs1421140	7	130123625	TSGA14 | MEST	ENST00000562524	ENSE00002587446	130121332	130124233	-1.509432008	0.011641273	0.265113513
kgp6353625	14	85991637	SEL1L | FLRT2	ENST00000380722	ENSE00001486019	85991477	85991851	0.918414076	0.012108415	0.265820453
kgp13933296	17	70068543	LOC124685 | SOX9	ENST00000419257	ENSE00001784948	70068474	70068983	-1.267974462	0.012484023	0.265820453
kgp1525997	12	1615724	ERC1 | FBXL14	ENST00000515614	ENSE00002050114	1615325	1616484	0.740470894	0.01261579	0.265820453
kgp7398633	11	69242990	MYEOV | CCND1	ENST00000545202	ENSE00002320990	69242131	69244389	1.008591614	0.013038541	0.265820453
kgp10784882	12	66006774	MSRB3 | RPSAP52	ENST00000541391	ENSE00002229891	66006472	66006777	1.038151964	0.013186082	0.265820453
rs10514804	2	47563573	BCYRN1 | EPCAM	ENST00000418539	ENSE00001641977	47558199	47571656	-0.930608732	0.013294953	0.265820453
rs2236000	11	32164835	RCN1 | WT1	ENST00000525133	ENSE00002145809	32164690	32165125	1.338063156	0.013486145	0.265820453
kgp20522819	8	135809850	ZFAT | LOC286094	ENST00000568248	ENSE00002623781	135804263	135810515	-1.56221932	0.013584142	0.265820453
kgp8325261	1	143186374	LOC100289550 | LOC10013	ENST00000437267	ENSE00001766550	143186154	143186607	-0.880216638	0.013687466	0.265820453
rs1055813	14	85886214	SEL1L | FLRT2	ENST00000557155	ENSE00002467826	85886028	85886396	1.085724548	0.014033428	0.265820453
kgp9850795	16	83833051	CDH13 | HSBP1	ENST00000567109	ENSE00002630405	83831266	83834245	1.217061754	0.014094871	0.265820453
rs2248625	12	66006488	MSRB3 | RPSAP52	ENST00000541391	ENSE00002229891	66006472	66006777	0.99982546	0.01447814	0.266555188
kgp6657500	17	70068663	LOC124685 | SOX9	ENST00000419257	ENSE00001784948	70068474	70068983	-1.508954755	0.014575512	0.266555188
rs2292480	1	212640367	LOC100287008 | LOC10028	ENST00000439570	ENSE00001732188	212640158	212640439	-1.090822061	0.041090402	0.274470457
kgp6403132	16	1029627	LMF1 | LOC100287129	ENST00000568394	ENSE00002581746	1029140	1031590	-1.901231606	0.04136721	0.274470457
kgp31115618		121006762	LOC643421 | GRIA3	ENST00000569460	ENSE00002575903	121005722	121010718	-1.001369909	0.041391184	0.274470457
rs4799131	18	77854379	C18orf22 | ADNP2	ENST00000569722	ENSE00002591961	77853168	77854968	-1.080850766	0.041586433	0.274470457
kgp18961336	12	1611716	ERC1 | FBXL14	ENST00000515614	ENSE00002028096	1611627	1612636	1.620547206	0.04158986	0.274684544
kgp3214838	20	2187943	STK35 | TGM3	ENST00000447956	ENSE00001798808	2187863	2188034	1.398504848	0.042118297	0.274684544
kgp30538070		121008174	LOC643421 | GRIA3	ENST00000569460	ENSE00002575903	121005722	121010718	-1.551087788	0.042939932	0.277846616
kgp24838613	1	234768772	IRF2BP2 | PP2672	ENST00000429269	ENSE00001771955	234768694	234770526	1.016289494	0.01638057	0.295094737
rs7822662	8	121064449	DEPDC6 | COL14A1	ENST00000520544	ENSE00002133133	121064419	121064634	-2.078337113	0.016972572	0.296514601
rs10742265	11	32158713	RCN1 | WT1	ENST00000525133	ENSE00002166436	32158667	32158788	1.52003201	0.017174746	0.296514601
kgp19774700	15	101404488	LOC145757 | ALDH1A3	ENST00000431060	ENSE00001634368	101401151	101404501	-1.471722341	0.017267	0.296514601

kgp9735261	4	106061668	LOC728847 | TET2	ENST00000504082	ENSE00002028435	106061667	106061776	-1.5716652	0.017888439	0.296514601
kgp11192799	16	63094271	CDH8 | CDH11	ENST00000563003	ENSE00002627024	63094198	63094314	0.670937727	0.018208293	0.296514601
kgp10789307	16	81993629	PLCG2 | SDR42E1	ENST00000564138	ENSE00002630315	81993524	81996298	-1.666530107	0.01847692	0.296514601
kgp18581058	9	96939087	PTPDC1 | ZNF169	ENST00000416309	ENSE00001793816	96938884	96940013	-0.890378497	0.018826877	0.296514601
kgp22826151	1	147816419	NBPF11 | LOC100132057	ENST00000452996	ENSE00001623768	147816357	147816764	-0.879241471	0.018898025	0.296514601
rs759497	17	70026560	LOC124685 | SOX9	ENST00000538693	ENSE00002240848	70026432	70026992	-1.669606778	0.019070334	0.296514601
kgp322149	2	227045550	KIAA1486 | IRS1	ENST00000423838	ENSE00001659923	227044760	227045841	0.955791412	0.019195931	0.296514601
kgp8201997	4	185540373	IRF2 | CASP3	ENST00000520280	ENSE00002120235	185540043	185540471	0.651050389	0.01953377	0.296514601
kgp31098924		7986173	PNPLA4 | LOC100288723	ENST00000422160	ENSE00001648336	7986097	7986201	0.670791037	0.020146321	0.296514601
kgp22649205	5	43015068	LOC100288522 | LOC64898	ENST00000503152	ENSE00002079428	43014838	43015252	0.68072667	0.020454308	0.296514601
kgp28517124	15	101404400	LOC145757 | ALDH1A3	ENST00000431060	ENSE00001634368	101401151	101404501	-1.161016243	0.020474477	0.296514601
kgp20028810	15	69760641	RPLP1 | LOC145837	ENST00000560882	ENSE00002551439	69760512	69760732	-0.650857746	0.020503987	0.296514601
rs12767790	10	70240115	DNA2 | SLC25A16	ENST00000439904	ENSE00001658409	70239548	70240521	-1.600488115	0.020851192	0.296514601
kgp29152561	4	158587962	LOC340017 | C4orf18	ENST00000507296	ENSE00002024944	158587823	158588196	-1.132772575	0.021152084	0.296514601
kgp378470	12	8449289	LOC653113 | LOC389634	ENST00000509919	ENSE00002028173	8449260	8450140	-0.760279707	0.021285954	0.296514601
kgp22766780	21	46414406	C21orf70 | NCRNA00162	ENST00000569966	ENSE00002621024	46414277	46415100	1.064977619	0.021333857	0.296514601
kgp4285429	9	3671575	RFX3 | GLIS3	ENST00000457566	ENSE00001687028	3671474	3671646	-0.810842659	0.021755146	0.296514601
kgp13310554	7	17503099	AHR | SNX13	ENST00000451792	ENSE00001773349	17503069	17503166	-0.869398498	0.021780671	0.296514601
kgp16359826	16	1026720	LMF1 | LOC100287129	ENST00000565467	ENSE00002593914	1025761	1027029	-2.499666773	0.02214865	0.296514601
kgp19017457	12	1615907	ERC1 | FBXL14	ENST00000515614	ENSE00002050114	1615325	1616484	0.732337569	0.022706071	0.296514601
kgp8477390	10	47096896	PPYR1 | LOC728643	ENST00000422732	ENSE00001719699	47096454	47098649	0.737108521	0.023066522	0.296514601
kgp2719408	4	40318894	RHOH | CHRNA9	ENST00000510551	ENSE00002087317	40318502	40318997	0.839616235	0.023167748	0.296514601
kgp1478336	11	67394604	LOC390213 | NUDT8	ENST00000533311	ENSE00002187408	67394518	67394775	1.254306432	0.023373302	0.296514601
kgp8107570	5	27477815	CDH9 | LOC729862	ENST00000514255	ENSE00002073910	27477746	27477908	-0.979804046	0.023565329	0.296514601
kgp9110312	2	156881093	KCNJ3 | NR4A2	ENST00000448255	ENSE00001719938	156880978	156881125	-2.240714513	0.023730576	0.296514601
kgp24656135	22	27261593	MIAT | MN1	ENST00000434868	ENSE00001619874	27258220	27261749	-1.592174113	0.023792945	0.296514601
kgp26338162	6	143277622	HIVEP2 | AIG1	ENST00000421237	ENSE00001750667	143277581	143277835	0.876215058	0.02382926	0.296514601
kgp28927962	8	123426668	HAS2AS | LOC100131552	ENST00000533992	ENSE00002158567	123426571	123426794	1.043804332	0.024945948	0.303709131
kgp3548794	4	11476533	HS3ST1 | HSP90AB2P	ENST00000515343	ENSE00002070051	11476384	11479820	-1.337289504	0.025936191	0.303709131
kgp12153866	14	53992760	DDHD1 | BMP4	ENST00000425648	ENSE00001776897	53992529	53992960	0.800257862	0.025946659	0.303709131
rs456699	16	83833689	CDH13 | HSBP1	ENST00000567109	ENSE00002630405	83831266	83834245	1.536320846	0.025962229	0.303709131
kgp12575988	5	27489430	CDH9 | LOC729862	ENST00000514844	ENSE00002045005	27489389	27489493	-1.22201381	0.026242582	0.303709131
kgp8993757	7	149699446	ATP6V0E2 | LOC100286961	ENST00000565102	ENSE00002598017	149697841	149700167	-0.654933813	0.026258011	0.303709131
kgp15759811	1	234770102	IRF2BP2 | PP2672	ENST00000429269	ENSE00001771955	234768694	234770526	0.833574404	0.026317409	0.303709131
kgp10943768	3	195677146	LOC727978 | SDHALP1	ENST00000570130	ENSE00002622318	195676059	195679566	-0.881715367	0.026459846	0.303709131
kgp319249	14	21513917	RNASE7 | RNASE8	ENST00000554568	ENSE00002516697	21511515	21514097	0.73274517	0.026921342	0.303709131
rs17780828	16	83833126	CDH13 | HSBP1	ENST00000567109	ENSE00002630405	83831266	83834245	1.007395313	0.027249172	0.303709131
kgp1106628	16	1026187	LMF1 | LOC100287129	ENST00000568394	ENSE00002595585	1025761	1026400	-1.048494821	0.027425757	0.303709131
kgp553755	9	94912677	LOC100128076 | LOC13865	ENST00000415471	ENSE00001624865	94912663	94912782	-0.821063124	0.027426922	0.303709131
kgp17356297	6	26686534	ZNF322A | GUSBL1	ENST00000562904	ENSE00002603318	26686469	26688192	-0.675581862	0.028224996	0.306948662
kgp10981302	2	52953908	LOC730100 | ASB3	ENST00000443237	ENSE00001712807	52953810	52953956	0.924652059	0.028285866	0.306948662
kgp4163244	8	102180742	YWHAZ | ZNF706	ENST00000565617	ENSE00002623035	102179033	102181857	-0.794351464	0.028482395	0.306948662
kgp1806364	4	185925755	ACSL1 | HELT	ENST00000505053	ENSE00002024490	185925654	185926340	-1.395462533	0.050525955	0.31032423
kgp2976383	8	10338456	MSRA | LOC346702	ENST00000518098	ENSE00002137308	10338056	10338496	-1.228113731	0.050965784	0.31032423
rs10831729	11	12110957	DKK3 | MICAL2	ENST00000527997	ENSE00002178930	12110669	12110988	1.089493621	0.051295071	0.31032423
kgp25283399	1	23612142	HTR1D | HNRNPR	ENST00000566551	ENSE00002595853	23607802	23613245	-1.27572205	0.052053241	0.31032423
kgp8268647	4	185910223	ACSL1 | HELT	ENST00000505053	ENSE00002041220	185910151	185910374	-1.152289007	0.054809043	0.31032423
rs1941075	18	12774848	PSMG2 | PTPN2	ENST00000563722	ENSE00002613997	12774650	12775922	-1.424332805	0.05596512	0.31032423
rs7593863	2	12855750	LOC729992 | LOC10028824	ENST00000569860	ENSE00002607395	12855541	12856353	-1.494854127	0.056581197	0.31032423
kgp380508	11	46260460	PHF21A | CREB3L1	ENST00000530049	ENSE00002168074	46260290	46260818	1.388443091	0.056993397	0.31032423
kgp8133391	9	33699329	PTENP1 | PRSS3	ENST00000566968	ENSE00002591924	33697457	33700984	1.652755904	0.05704755	0.31032423
kgp30444589	5	175603339	FAM153B | LOC643201	ENST00000515403	ENSE00002085036	175603285	175603453	0.965540061	0.057362964	0.31032423
rs533483	1	234765256	IRF2BP2 | PP2672	ENST00000429269	ENSE00001620425	234765057	234765493	1.045794654	0.029412295	0.314164956
kgp5762526	4	185910363	ACSL1 | HELT	ENST00000505053	ENSE00002041220	185910151	185910374	-1.276523734	0.060043953	0.318520505
kgp6987718	12	1612166	ERC1 | FBXL14	ENST00000515614	ENSE00002028096	1611627	1612636	0.968660122	0.061708054	0.318520505
kgp3482504	20	25845433	FAM182B | LOC100134868	ENST00000424021	ENSE00001770202	25844905	25845903	-1.292021561	0.061773674	0.318520505
kgp17875518	3	44162104	ABHD5 | LOC375337	ENST00000568686	ENSE00002586185	44158791	44163857	1.259563455	0.063596217	0.322757585
kgp22758386		134531624	ZNF449 | NCRNA00086	ENST00000439434	ENSE00001751572	134531318	134531689	-1.191285801	0.064642207	0.322757585
kgp4610052	6	134824656	SGK1 | LOC645175	ENST00000456749	ENSE00001676049	134823926	134825157	0.992311108	0.065851883	0.322757585
kgp20879775	4	185925693	ACSL1 | HELT	ENST00000505053	ENSE00002024490	185925654	185926340	-1.501082256	0.066507624	0.322757585
kgp578685	21	26887881	NCRNA00158 | MIRHG2	ENST00000419694	ENSE00001669365	26887785	26888767	1.314108235	0.050584007	0.322876638
kgp10926979	14	71107942	MED6 | TTC9	ENST00000500016	ENSE00001965431	71107725	71108015	-0.892019938	0.03076734	0.323574802
kgp3348927	4	125421750	LOC100289258 | ANKRD50	ENST00000563724	ENSE00002600455	125421097	125422149	0.740293095	0.030928367	0.323574802
kgp5162755	18	12775013	PSMG2 | PTPN2	ENST00000563722	ENSE00002613997	12774650	12775922	-1.565374115	0.031097496	0.323574802
kgp7907385	14	70950294	ADAM21 | ADAM20	ENST00000556964	ENSE00002478502	70950152	70950473	-0.918692902	0.069130602	0.325578236
rs1856197	9	33698114	PTENP1 | PRSS3	ENST00000566968	ENSE00002591924	33697457	33700984	1.34231282	0.069467071	0.325578236
rs1765372	9	33699373	PTENP1 | PRSS3	ENST00000566968	ENSE00002591924	33697457	33700984	1.428342253	0.070048651	0.325578236
kgp2664414	2	95538078	TEKT4 | LOC100289554	ENST00000568768	ENSE00002576720	95533231	95539050	-1.333412366	0.053713613	0.331160111
rs7224456	17	26592020	PPY2 | FLJ40504	ENST00000564762	ENSE00002585454	26590660	26593395	1.309342963	0.054089485	0.331160111
kgp1869919	3	177402761	TBL1XR1 | KCNMB2	ENST00000423466	ENSE00001744151	177402732	177402802	1.05569289	0.032180334	0.331980027
kgp12556318	18	12775827	PSMG2 | PTPN2	ENST00000563722	ENSE00002613997	12774650	12775922	-1.690062964	0.032643188	0.332178302
rs11227191	11	65209285	NCRNA00084 | MALAT1	ENST00000501122	ENSE00001961965	65190269	65213011	-1.162772774	0.033545064	0.332178302
rs4798937	18	77854537	C18orf22 | ADNP2	ENST00000569722	ENSE00002591961	77853168	77854968	-0.64486344	0.033753469	0.332178302
kgp17704603	3	156800634	LEKR1 | CCNL1	ENST00000471357	ENSE00001879700	156799456	156801064	0.675709145	0.034195859	0.332178302
kgp27848408	12	126845045	TMEM132B | LOC10028870	ENST00000536639	ENSE00002273467	126844647	126845611	-0.902779855	0.034320798	0.332178302
kgp7941860	2	47563683	BCYRN1 | EPCAM	ENST00000418539	ENSE00001641977	47558199	47571656	-0.813381274	0.03437503	0.332178302
rs2855405	16	67464617	ZDHHC1 | HSD11B2	ENST00000567261	ENSE00002578405	67464570	67464909	-0.965694379	0.034419855	0.332178302
kgp11471865	4	8358271	HTRA3 | ACOX3	ENST00000505448	ENSE00002069127	8357038	8359103	-0.76338616	0.034784632	0.332178302
kgp6113855	15	95400168	LOC440311 | LOC400456	ENST00000568835	ENSE00002603259	95399899	95400214	0.632511945	0.034972492	0.332178302
rs543651	15	63190950	TLN2 | TPM1	ENST00000557900	ENSE00002572006	63190850	63191742	0.939579172	0.035086243	0.332178302
kgp28457880	15	69760668	RPLP1 | LOC145837	ENST00000560882	ENSE00002551439	69760512	69760732	-0.694198387	0.035226862	0.332178302
kgp6816341	18	4295276	LOC284215 | LOC642597	ENST00000565811	ENSE00002615565	4293160	4295405	-2.884273279	0.006496692	0.332429421
kgp3850572	5	53072734	NDUFS4 | ARL15	ENST00000504552	ENSE00002029783	53072474	53073116	3.077473183	0.006786856	0.332429421
kgp4803222	16	1029276	LMF1 | LOC100287129	ENST00000568394	ENSE00002581746	1029140	1031590	-2.774907767	0.008639383	0.332429421
rs10483380	14	30674898	PRKD1 | G2E3	ENST00000508469	ENSE00002023847	30674740	30674946	-2.817285724	0.009175411	0.332429421
kgp6819366	1	20687755	VWA5B1 | CAMK2N1	ENST00000426428	ENSE00001636211	20687072	20688534	2.760057668	0.009340625	0.332429421

kgp15517179	1	222087825	DUSP10 | LOC100129950	ENST00000433576	ENSE00001758366	222087765	222087873	2.454234293	0.011041264	0.332429421
kgp8037842	14	62570323	SYT16 | FLJ43390	ENST00000554252	ENSE00002439671	62570096	62570499	2.401872053	0.01197438	0.332429421
rs523351	18	4295347	LOC284215 | LOC642597	ENST00000565811	ENSE00002615565	4293160	4295405	-2.421413408	0.012102758	0.332429421
rs17173563	10	48331883	LOC653110 | ZNF488	ENST00000454672	ENSE00001704522	48331809	48332197	-2.426293967	0.013214618	0.332429421
rs2046701	4	27211040	STIM2 | PCDH7	ENST00000382007	ENSE00001490576	27209127	27211372	2.316722346	0.013398857	0.332429421
kgp13544745	7	124791507	POT1 | GRM8	ENST00000454957	ENSE00001716537	124791380	124791830	-2.957555934	0.013970912	0.332429421
kgp4267232	4	188225262	FAT1 | ZFP42	ENST00000511385	ENSE00002073108	188225237	188225621	-2.539968002	0.014203553	0.332429421
kgp8885108	4	74580017	RASSF6 | IL8	ENST00000436089	ENSE00001754580	74579883	74580244	2.269638069	0.015180624	0.332429421
kgp19273385	20	51474899	ZFP64 | TSHZ2	ENST00000426963	ENSE00001648389	51474877	51475046	-2.255956888	0.015371494	0.332429421
kgp12164816	2	47567102	BCYRN1 | EPCAM	ENST00000418539	ENSE00001641977	47558199	47571656	-2.213801308	0.015435245	0.332429421
kgp391218	3	153658575	LOC152118 | SGEF	ENST00000463297	ENSE00001915943	153658322	153658625	2.525910583	0.01603349	0.332429421
kgp10065947	12	65938148	MSRB3 | RPSAP52	ENST00000511935	ENSE00002027971	65938018	65938271	2.148659218	0.016882349	0.332429421
kgp10872571	17	11465720	FLJ45455 | LOC100289291	ENST00000528745	ENSE00002184577	11464473	11467380	-2.318334393	0.017696976	0.332429421
kgp20757537	4	95676006	PDLIM5 | BMPR1B	ENST00000510795	ENSE00002043200	95675981	95676056	2.258937018	0.018251437	0.332429421
kgp5118555	6	14280961	CD83 | JARID2	ENST00000427276	ENSE00001723124	14280358	14283419	2.482989989	0.018459594	0.332429421
rs2278055	16	76141342	LOC100289629 | CNTNAP4	ENST00000564561	ENSE00002629788	76141181	76141401	3.476391486	0.019243387	0.332429421
kgp9786837	16	58467739	GINS3 | NDRG4	ENST00000567448	ENSE00002578209	58467714	58467852	2.285340895	0.020066814	0.332429421
kgp30787634		118469017	PGRMC1 | SLC25A43	ENST00000428222	ENSE00001764461	118467013	118469573	3.209321712	0.02103253	0.332429421
kgp7460813	10	7139472	LOC439949 | SFMBT2	ENST00000420049	ENSE00001641917	7139404	7139637	1.995097134	0.021437605	0.332429421
rs897067	2	3891792	LOC728597 | SOX11	ENST00000451101	ENSE00001607396	3891774	3892232	2.419863892	0.022114395	0.332429421
kgp8105254	12	89405221	KITLG | DUSP6	ENST00000500381	ENSE00001976792	89404458	89407094	2.319399262	0.022668933	0.332429421
rs2285935	11	2014646	LOC100133545 | H19	ENST00000418612	ENSE00001677397	2014485	2014699	-1.964742764	0.024288274	0.332429421
rs3740218	10	9801169	GATA3 | SFTA1P	ENST00000419836	ENSE00001606593	9801042	9801200	1.881764496	0.025317854	0.332429421
kgp30710920		1874722	ASMT |	ENST00000432272	ENSE00001667046	1874577	1874878	2.458898281	0.026388986	0.332429421
kgp4024529	2	3894919	LOC728597 | SOX11	ENST00000451101	ENSE00001772647	3894744	3895001	2.800012088	0.026684231	0.332429421
kgp8054489	4	188336850	FAT1 | ZFP42	ENST00000503677	ENSE00002020154	188336464	188336851	-1.829653462	0.027987593	0.332429421
kgp16968552	6	14280888	CD83 | JARID2	ENST00000427276	ENSE00001723124	14280358	14283419	1.968067395	0.028637266	0.332429421
kgp4869442	17	11465536	FLJ45455 | LOC100289291	ENST00000528745	ENSE00002184577	11464473	11467380	-1.777049739	0.030257602	0.332429421
kgp28500	14	51800136	TMX1 | FRMD6	ENST00000556479	ENSE00002430341	51800111	51800282	1.777312752	0.030563002	0.332429421
rs3828088	1	222087833	DUSP10 | LOC100129950	ENST00000433576	ENSE00001758366	222087765	222087873	1.782442401	0.030887294	0.332429421
rs17028166	2	103603960	TMEM182 | LOC644265	ENST00000435291	ENSE00001699112	103603952	103604071	-1.771859799	0.031387776	0.332429421
kgp6338384	1	94798858	ARHGAP29 | ABCD3	ENST00000418242	ENSE00001609430	94798823	94798965	1.782546243	0.032152443	0.332429421
kgp5212323	12	68323030	DYRK2 | IFNG	ENST00000545520	ENSE00002285526	68323015	68323167	1.750208775	0.032679939	0.332429421
kgp22152625	5	1546912	LPCAT1 | SDHAP3	ENST00000503113	ENSE00002023116	1546874	1547080	-1.723768383	0.032805739	0.332429421
kgp6798649	2	213661416	ERBB4 | LOC646249	ENST00000434559	ENSE00001604224	213660735	213661444	-1.726892975	0.032806687	0.332429421
kgp22773306	4	174389179	SCRG1 | HAND2	ENST00000563631	ENSE00002606014	174388984	174391192	-1.782052832	0.032872632	0.332429421
kgp1745820	6	14282023	CD83 | JARID2	ENST00000427276	ENSE00001723124	14280358	14283419	2.105361354	0.033821232	0.332429421
kgp20996292	4	27278734	STIM2 | PCDH7	ENST00000512873	ENSE00002054503	27278487	27278915	-2.235574686	0.034006301	0.332429421
kgp13165363	21	46410200	C21orf70 | NCRNA00162	ENST00000439088	ENSE00001802548	46409779	46411747	1.788333102	0.037303476	0.332429421
kgp8059973	6	168082315	LOC401286 | LOC441178	ENST00000400831	ENSE00001612490	168080306	168082617	1.635071309	0.038176663	0.332429421
kgp12051842	8	130229164	PVT1 | LOC100287906	ENST00000509893	ENSE00002036854	130228728	130229652	1.693722095	0.039108997	0.332429421
kgp15851967	1	246845799	C1orf71 | SCCPDH	ENST00000570141	ENSE00002627157	246845410	246848377	-1.610980391	0.040111895	0.332429421
kgp19798877	15	45740904	C15orf48 | SLC30A4	ENST00000559869	ENSE00002543658	45740625	45740999	-1.626720873	0.040837856	0.332429421
rs6922929	6	19044000	RNF144B | ID4	ENST00000447237	ENSE00001721861	19043980	19044144	-2.011696465	0.042317781	0.332429421
rs855434	9	33700901	PTENP1 | PRSS3	ENST00000566968	ENSE00002591924	33697457	33700984	1.572764045	0.042665455	0.332429421
kgp20927850	4	188228281	FAT1 | ZFP42	ENST00000511385	ENSE00002054563	188228260	188228423	-2.343096989	0.042676203	0.332429421
rs4806397	19	28909671	LOC148189 | LOC148145	ENST00000567877	ENSE00002576754	28909390	28910818	2.886917958	0.042941753	0.332429421
rs3286	7	93693901	BET1 | COL1A2	ENST00000415536	ENSE00001681093	93693738	93694035	1.750532567	0.043333979	0.332429421
kgp24074524	2	52949839	LOC730100 | ASB3	ENST00000443237	ENSE00001746572	52949815	52950001	1.585755989	0.044961277	0.332429421
kgp1195553	5	33245067	C5orf23 | TARS	ENST00000503577	ENSE00002066006	33244958	33245162	1.541311629	0.045070292	0.332429421
kgp2520325	4	166613474	CPE | TLL1	ENST00000507838	ENSE00002079374	166613328	166613497	1.55585601	0.045494599	0.332429421
kgp13579960	7	20345477	MACC1 | ITGB8	ENST00000455373	ENSE00001787242	20345334	20345646	1.534677942	0.046197557	0.332429421
kgp22542490	5	139121182	CXXC5 | PSD2	ENST00000515306	ENSE00002047047	139120934	139121225	1.773456365	0.046368094	0.332429421
kgp10346659	7	51682035	LOC100131871 | DKFZp564	ENST00000414409	ENSE00001688459	51681947	51682342	1.518880986	0.046919241	0.332429421
kgp9704923	6	30756596	IER3 | DDR1	ENST00000439406	ENSE00001653297	30756468	30756649	2.204625396	0.046938828	0.332429421
kgp11585865	1	209541248	PLXNA2 | LOC642587	ENST00000443636	ENSE00001715807	209541234	209541357	1.64248105	0.047268519	0.332429421
kgp17759655	3	177652615	TBL1XR1 | KCNMB2	ENST00000450500	ENSE00001709656	177652611	177652751	-1.544767584	0.048488127	0.334514817
rs5997130	22	27258653	MIAT | MN1	ENST00000434868	ENSE00001619874	27258220	27261749	-1.497737738	0.050874544	0.334514817
kgp4997265	14	51426659	PYGL | TRIM9	ENST00000553648	ENSE00002451667	51422977	51427333	-1.624342383	0.05161452	0.334514817
rs7323288	13	45657195	KIAA1704 | GTF2F2	ENST00000437748	ENSE00001761242	45657173	45657260	1.558123214	0.052512919	0.334514817
kgp602848	17	16918025	TNFRSF13B | MPRIP	ENST00000562897	ENSE00002580335	16915228	16918304	-1.458805561	0.052575604	0.334514817
kgp4960635	7	93695755	BET1 | COL1A2	ENST00000438538	ENSE00001771519	93695679	93695973	1.461620046	0.053330809	0.334514817
kgp2825390	22	27259889	MIAT | MN1	ENST00000434868	ENSE00001619874	27258220	27261749	-1.454980344	0.05461474	0.334514817
kgp10458664	7	26594478	KIAA0087 | LOC285941	ENST00000457000	ENSE00001792454	26594428	26594538	1.603874222	0.055264144	0.334514817
kgp26644528	3	64072949	LOC100287879 | PRICKLE2	ENST00000485805	ENSE00002071040	64072553	64073039	1.527612851	0.056133184	0.334514817
kgp671519	11	57812244	OR6Q1 | OR9I1	ENST00000526131	ENSE00002143143	57811582	57812814	1.416396469	0.056763671	0.334514817
kgp25790336	16	50673665	NKD1 | SNX20	ENST00000565077	ENSE00002621561	50671504	50674771	1.48134543	0.05678227	0.334514817
kgp7857501	6	107144167	QRSL1 | LOC553137	ENST00000424162	ENSE00001693072	107144150	107144275	1.457090098	0.056922102	0.334514817
kgp11795975	10	5637535	CALML3 | ASB13	ENST00000478294	ENSE00001928580	5636954	5637542	0.999239351	0.035810012	0.335059572
kgp10715219	20	23170450	LOC200261 | NXT1	ENST00000411595	ENSE00001684449	23170420	23170944	-1.281589913	0.056318365	0.335476705
kgp22376337	5	10197505	LOC285692 | FAM173B	ENST00000566945	ENSE00002600148	10195233	10197740	-1.38548203	0.05703104	0.335476705
kgp26681046	3	177040959	TBL1XR1 | KCNMB2	ENST00000425388	ENSE00001591376	177039217	177041206	1.521343169	0.058004572	0.336269751
kgp28835642	8	102181652	YWHAZ | ZNF706	ENST00000565617	ENSE00002623035	102179033	102181857	-1.506928416	0.036716544	0.340319833
kgp7134298	1	235097240	PP2672 | TOMM20	ENST00000458044	ENSE00001789725	235097123	235097313	0.646596326	0.037119716	0.340319833
kgp1200480	16	1027069	LMF1 | LOC100287129	ENST00000565069	ENSE00002607939	1026521	1027093	-1.70148615	0.037642319	0.340319833
kgp7576805	15	101403655	LOC145757 | ALDH1A3	ENST00000431060	ENSE00001634368	101401151	101404501	-1.435200169	0.037944336	0.340319833
rs405478	16	83832304	CDH13 | HSBP1	ENST00000567109	ENSE00002630405	83831266	83834245	0.92278686	0.038197458	0.340319833
kgp28922076	8	123426603	HAS2AS | LOC100131552	ENST00000533992	ENSE00002158567	123426571	123426794	0.965366728	0.038685961	0.340319833
kgp7481381	16	81993671	PLCG2 | SDR42E1	ENST00000564138	ENSE00002630315	81993524	81996298	-1.248658184	0.038830236	0.340319833
kgp27446049	9	109350663	TMEM38B | LOC644620	ENST00000435485	ENSE00001685919	109350658	109350745	0.755914591	0.038973349	0.340319833
kgp99793	15	101401421	LOC440313 | LOC145757	ENST00000431060	ENSE00001634368	101401151	101404501	-0.507371677	0.039118192	0.340319833
kgp15135146	1	158102005	KIRREL | CD1D	ENST00000442358	ENSE00001705002	158101834	158102191	1.898959267	0.039368164	0.340319833
kgp15481286	1	159948851	SLAMF9 | PIGM	ENST00000423943	ENSE00001610015	159948390	159948851	-1.045197653	0.039829385	0.340319833
kgp9002385	10	5637114	CALML3 | ASB13	ENST00000478294	ENSE00001928580	5636954	5637542	0.886840884	0.040473474	0.340319833
kgp3909776	11	67657230	LOC645332 | LOC10013226	ENST00000511677	ENSE00002078750	67655624	67658816	-0.782089765	0.040671893	0.340319833
rs2246373	4	177796385	VEGFC | NEIL3	ENST00000510203	ENSE00002058465	177796330	177796408	1.096628319	0.04084326	0.340319833

rs10874868	1	95087398	F3 | SLC44A3	ENST00000435559	ENSE00001601116	95086707	95087512	-0.894482618	0.041276646	0.340319833
kgp19796156	15	45804615	SLC30A4 | C15orf21	ENST00000560647	ENSE00002620391	45804571	45804647	-0.86928886	0.041465396	0.340319833
kgp18101736	3	156799531	LEKR1 | CCNL1	ENST00000471357	ENSE00001879700	156799456	156801064	0.685503625	0.04168787	0.340319833
kgp22810706	13	48506435	HTR2A | SUCLA2	ENST00000566385	ENSE00002595049	48504290	48506757	0.620691363	0.041715837	0.340319833
kgp11424248	11	73639711	PAAF1 | DNAJB13	ENST00000535936	ENSE00002217938	73639578	73639855	-1.513597261	0.041997234	0.340319833
kgp12161256	17	70085422	LOC124685 | SOX9	ENST00000419257	ENSE00001766642	70085228	70085816	-1.026775577	0.042011313	0.340319833
kgp893021	3	20431317	SGOL1 | VENTXP7	ENST00000566804	ENSE00002605809	20429741	20432054	-1.38684484	0.059965705	0.342422567
kgp11539052	4	11770411	HS3ST1 | HSP90AB2P	ENST00000515286	ENSE00002065709	11770352	11770434	-2.296213573	0.06108266	0.342422567
kgp6435531	14	71077857	MED6 | TTC9	ENST00000500016	ENSE00001980392	71075515	71078067	-1.41282088	0.061460461	0.342422567
kgp11174771	7	56561056	LOC100132050 | DKFZp434	ENST00000566570	ENSE00002614277	56560817	56564978	1.317358445	0.059492166	0.343224037
kgp30296223	5	175612262	LOC643201 | C5orf25	ENST00000508056	ENSE00002083677	175612104	175612345	1.404437764	0.064376478	0.345948908
kgp6622479	1	200447186	ZNF281 | KIF14	ENST00000427825	ENSE00001737494	200447096	200447421	1.402606087	0.065279612	0.345948908
kgp19201897	20	23170707	LOC200261 | NXT1	ENST00000411595	ENSE00001684449	23170420	23170944	-1.447407468	0.065892676	0.345948908
rs1492171	3	162919329	OTOL1 | LOC730129	ENST00000494897	ENSE00001816915	162919326	162919405	1.391949654	0.06596649	0.345948908
kgp18788039	12	126845541	TMEM132B | LOC10028870	ENST00000536639	ENSE00002273467	126844647	126845611	-1.845047314	0.066125432	0.345948908
rs17055549	4	171204193	AADAT | LOC441052	ENST00000504509	ENSE00002064375	171203902	171204230	0.96765404	0.07555886	0.34631144
kgp9791115	1	158102020	KIRREL | CD1D	ENST00000442358	ENSE00001705002	158101834	158102191	2.084141496	0.043279139	0.347800931
kgp7633219	14	21175150	RNASE4 | FAM12A	ENST00000554286	ENSE00002486764	21174906	21175279	-1.090456641	0.043511136	0.347800931
kgp18764117	12	89408584	KITLG | DUSP6	ENST00000500381	ENSE00001990176	89408469	89408645	0.710216488	0.044253159	0.350519904
kgp6065494	3	30566257	RBMS3 | TGFBR2	ENST00000450746	ENSE00001692853	30566237	30566318	0.729787749	0.045329608	0.350519904
rs7727632	5	90609009	LOC729040 | ARRDC3	ENST00000513626	ENSE00002034273	90606838	90609046	-1.234019768	0.045348362	0.350519904
rs16990157	20	44076324	LOC100130157 | WFDC2	ENST00000424705	ENSE00001676186	44076042	44076456	-0.576933462	0.045730569	0.350519904
kgp3592063	10	5637080	CALML3 | ASB13	ENST00000478294	ENSE00001928580	5636954	5637542	0.685851001	0.045827569	0.350519904
kgp9675747	7	157075201	UBE3C | DNAJB6	ENST00000442017	ENSE00001628506	157074838	157075380	-0.83124959	0.045960643	0.350519904
kgp158702	4	40318903	RHOH | CHRNA9	ENST00000510551	ENSE00002087317	40318502	40318997	0.673981091	0.046051936	0.350519904
kgp23841664	17	80251940	CSNK1D | CD7	ENST00000566986	ENSE00002591651	80251592	80252786	-0.955734518	0.046399755	0.350519904
rs6748811	2	156877774	KCNJ3 | NR4A2	ENST00000428651	ENSE00001782847	156877047	156878411	-2.000955842	0.046464942	0.350519904
kgp8378041	7	149699676	ATP6V0E2 | LOC100286961	ENST00000565102	ENSE00002598017	149697841	149700167	-1.251929911	0.062254538	0.35238418
kgp6968619	8	127341403	LOC650095 | FAM84B	ENST00000500180	ENSE00001990119	127340553	127341780	1.313443055	0.069374346	0.353980523
kgp22831217	7	43011894	MRPL32 | LOC100288217	ENST00000456114	ENSE00001756348	43011866	43012442	1.328667479	0.069426006	0.353980523
kgp14517369	2	187219719	FLJ44048 | ZC3H15	ENST00000564407	ENSE00002576512	187219299	187221500	-1.678364653	0.070136001	0.353980523
kgp6540047	3	30566773	RBMS3 | TGFBR2	ENST00000431057	ENSE00001758672	30566482	30566939	1.660082078	0.071755183	0.35481252
kgp10885924	21	26832720	NCRNA00158 | MIRHG2	ENST00000489205	ENSE00001933675	26832668	26832730	1.298702548	0.071954987	0.35481252
kgp15490341	1	234769913	IRF2BP2 | PP2672	ENST00000429269	ENSE00001771955	234768694	234770526	0.851328198	0.047838033	0.355794822
kgp4545267	18	12775417	PSMG2 | PTPN2	ENST00000563722	ENSE00002613990	12774650	12775922	-1.791031388	0.048371038	0.355794822
kgp11297526	16	32469511	LOC390705 | TP53TG3	ENST00000565549	ENSE00002609095	32465933	32470079	-0.680063746	0.048429795	0.355794822
kgp17212390	6	134824281	SGK1 | LOC645175	ENST00000456749	ENSE00001676049	134823926	134825157	0.706453025	0.049157118	0.355794822
kgp1913725	16	86754576	FOXL1 | LOC100288525	ENST00000563331	ENSE00002586134	86754294	86754609	-2.380399852	0.049654945	0.355794822
kgp17907087	3	177040671	TBL1XR1 | KCNMB2	ENST00000425388	ENSE00001591376	177039217	177041206	0.989062656	0.049724649	0.355794822
kgp18578461	9	109366134	TMEM38B | LOC644620	ENST00000435485	ENSE00001653720	109366037	109367076	0.785892329	0.049869401	0.355794822
kgp16225896	16	81993728	PLCG2 | SDR42E1	ENST00000564138	ENSE00002630315	81993524	81996298	-1.092638676	0.049873316	0.355794822
kgp23051235	11	65190595	FRMD8 | NCRNA00084	ENST00000501122	ENSE00001961965	65190269	65213011	-0.700404674	0.049881713	0.355794822
kgp5000754	1	1365570	TMEM88B | LOC100288271	ENST00000428781	ENSE00001762472	1365355	1365635	-1.521808309	0.050346436	0.355794822
kgp22826774	8	135805982	ZFAT | LOC286094	ENST00000568248	ENSE00002623781	135804263	135810515	-0.545095603	0.05043719	0.355794822
rs4422207	2	47564034	BCYRN1 | EPCAM	ENST00000418539	ENSE00001641977	47558199	47571656	-0.921257005	0.051066392	0.355794822
rs2017650	8	23351559	ENTPD4 | SLC25A37	ENST00000518590	ENSE00002103749	23351317	23351711	-0.627722753	0.051284438	0.355794822
kgp17381880	6	160697065	SLC22A2 | SLC22A3	ENST00000419196	ENSE00001749585	160696793	160697162	0.803022812	0.051291051	0.355794822
kgp4894937	17	70026609	LOC124685 | SOX9	ENST00000538693	ENSE00002240848	70026432	70026992	-1.655108861	0.052002302	0.356633081
kgp1056253	22	27261205	MIAT | MN1	ENST00000434868	ENSE00001619874	27258220	27261749	-1.072441476	0.052007259	0.356633081
rs11996854	8	102180058	YWHAZ | ZNF706	ENST00000565617	ENSE00002623035	102179033	102181857	-0.474495931	0.052889237	0.356633081
kgp24934090	1	234768953	IRF2BP2 | PP2672	ENST00000429269	ENSE00001771955	234768694	234770526	0.950682096	0.053138638	0.356633081
kgp9963829	17	12538520	MAP2K4 | MYOCD	ENST00000313495	ENSE00001251429	12538318	12540504	0.544544963	0.05323401	0.356633081
kgp8960906	4	11471259	HS3ST1 | HSP90AB2P	ENST00000515343	ENSE00002047387	11470874	11471455	-1.211933066	0.053307181	0.356633081
kgp15729197	1	234769113	IRF2BP2 | PP2672	ENST00000429269	ENSE00001771955	234768694	234770526	0.817471321	0.053480189	0.356633081
kgp25137749	1	158102131	KIRREL | CD1D	ENST00000442358	ENSE00001705002	158101834	158102191	1.825918115	0.054445625	0.358805571
kgp22172935	5	87565642	TMEM161B | LOC645323	ENST00000504636	ENSE00002071912	87565285	87566247	-0.583369805	0.0548908	0.358805571
kgp9958448	2	192108553	STAT4 | MYO1B	ENST00000419640	ENSE00001719703	192108346	192108799	0.628566624	0.055007583	0.358805571
kgp16244546	16	81112257	C16orf46 | GCSH	ENST00000501068	ENSE00001976225	81111937	81112466	-0.484282788	0.05507173	0.358805571
kgp6568995	6	129849833	LAMA2 | ARHGAP18	ENST00000430296	ENSE00001679894	129849810	129849883	-0.530718082	0.055292325	0.358805571
kgp19966503	15	69760593	RPLP1 | LOC145837	ENST00000560882	ENSE00002551439	69760512	69760732	-0.831062437	0.08004118	0.361829992
kgp11077394	12	1612239	ERC1 | FBXL14	ENST00000515614	ENSE00002028096	1611627	1612636	1.339751229	0.056059496	0.361838563
kgp5945220	8	81447844	ZBTB10 | ZNF704	ENST00000523871	ENSE00002102242	81447241	81450812	-0.963164855	0.081216765	0.362182873
kgp5793648	14	85995131	SEL1L | FLRT2	ENST00000380722	ENSE00001486021	85994961	85996332	0.732992869	0.056622485	0.363528401
kgp24006502	2	38466554	C2orf58 | ATL2	ENST00000450854	ENSE00001733174	38466200	38466732	-1.372830416	0.082784451	0.364251585
rs7828323	8	102181249	YWHAZ | ZNF706	ENST00000565617	ENSE00002623035	102179033	102181857	-1.499778118	0.057329435	0.364583426
kgp15831311	1	16788219	NECAP2 | CROCCL2	ENST00000457898	ENSE00001761634	16787443	16789782	-0.661829988	0.05768881	0.364583426
kgp4707227	16	81418132	GAN | CMIP	ENST00000568107	ENSE00002626991	81416874	81424489	-0.963235426	0.057692986	0.364583426
rs7788668	7	22980638	SNORD93 | FAM126A	ENST00000421730	ENSE00001741676	22980591	22980702	0.781361626	0.058513367	0.366347048
kgp17368545	6	82555244	FAM46A | IBTK	ENST00000418567	ENSE00001802829	82554902	82555626	-0.616415967	0.058579105	0.366347048
rs11801300	1	28974734	TAF12 | RNU11	ENST00000427804	ENSE00001796797	28974705	28975093	0.457300285	0.059412113	0.369641339
kgp388921	1	143394413	LOC100132733 | LOC10013	ENST00000421640	ENSE00001628783	143393991	143394444	-0.904517624	0.05999812	0.371327727
kgp12005625	2	114588799	SLC35F5 | ACTR3	ENST00000446401	ENSE00001709078	114588765	114589005	-0.575017122	0.060973259	0.371327727
kgp18852302	12	1612036	ERC1 | FBXL14	ENST00000515614	ENSE00002028096	1611627	1612636	0.907723003	0.061074653	0.371327727
kgp18709749	12	1612266	ERC1 | FBXL14	ENST00000515614	ENSE00002028096	1611627	1612636	1.114406576	0.061167138	0.371327727
kgp648284	8	29647917	C8orf75 | LOC286135	ENST00000523123	ENSE00002100667	29647848	29647963	0.926043371	0.06122139	0.371327727
kgp28878331	8	123429113	HAS2AS | LOC100131552	ENST00000533992	ENSE00002177014	123429064	123429261	0.84145007	0.061549262	0.371449794
kgp5520275	9	5588988	PDCD1LG2 | KIAA1432	ENST00000443792	ENSE00001619824	5588593	5588994	-1.280570558	0.066934732	0.37185962
kgp4255269	9	3691706	RFX3 | GLIS3	ENST00000427722	ENSE00001728381	3691504	3691814	-1.02480045	0.087191167	0.374523017
rs1061568	18	65149160	CDH19 | DSEL	ENST00000562669	ENSE00002612652	65149028	65152203	0.819181568	0.08748039	0.374523017
kgp6395801	2	221662583	SLC4A3 | EPHA4	ENST00000424395	ENSE00001644598	221662181	221662613	1.006921542	0.089289951	0.374523017
rs12660871	6	143277652	HIVEP2 | AIG1	ENST00000421237	ENSE00001750667	143277581	143277835	0.859535466	0.08965854	0.374523017
kgp23793121	17	11465891	FLJ45455 | LOC100289291	ENST00000528745	ENSE00002184577	11464473	11467380	-1.863369933	0.078337041	0.374990193
kgp6915132	6	147183108	C6orf103 | STXBP5	ENST00000436295	ENSE00001748830	147183097	147183177	1.347435354	0.078564891	0.374990193
kgp1526057	6	86099813	LOC100289423 | NT5E	ENST00000455071	ENSE00001752264	86099731	86099904	1.527397572	0.078669271	0.374990193
kgp11196053	6	3906054	LOC100289591 | PRPF4B	ENST00000566733	ENSE00002617674	3905144	3912213	0.754451502	0.0633071	0.376752468
kgp2671926	16	70610223	SF3B3 | IL34	ENST00000562874	ENSE00002576989	70610204	70610251	-1.619729759	0.063404888	0.376752468

kgp8219122	10	5637993	CALML3 | ASB13	ENST00000478294	ENSE00001823487	5637974	5638081	0.757962671	0.063457059	0.376752468
kgp7323492	12	133490234	CHFR | LOC100289635	ENST00000503695	ENSE00002082163	133489682	133491318	-0.552688648	0.063875499	0.376752468
kgp28616538	8	102151915	YWHAZ | ZNF706	ENST00000518090	ENSE00002093548	102151893	102151979	0.731060959	0.063988613	0.376752468
kgp11888975	2	221315486	SLC4A3 | EPHA4	ENST00000432993	ENSE00001620071	221315323	221315499	1.40052714	0.069204406	0.376815866
kgp10165058	4	174907906	MORF4 | FBXO8	ENST00000503325	ENSE00002067875	174907844	174907950	-1.243995912	0.070338962	0.376815866
kgp6507046	15	45752975	C15orf48 | SLC30A4	ENST00000559960	ENSE00002569243	45752644	45753154	-1.302384668	0.080367609	0.37887587
kgp2765098	11	82803315	RAB30 | PCF11	ENST00000533528	ENSE00002157702	82803075	82803380	-0.999532756	0.064972145	0.379829802
kgp19435987	14	106067375	TMEM121 | LOC100288531	ENST00000549427	ENSE00002587194	106067228	106067548	0.462737486	0.065140654	0.379829802
kgp13133286	21	18012732	C21orf34 | CXADR	ENST00000438762	ENSE00001712871	18012549	18013002	-1.296499212	0.072924354	0.381088587
kgp8668768	1	153163300	SPRR2G | LELP1	ENST00000427145	ENSE00001706753	153162555	153164152	1.23045311	0.073677127	0.381088587
kgp5147558	11	46260304	PHF21A | CREB3L1	ENST00000530049	ENSE00002168074	46260290	46260818	1.26715973	0.081949451	0.382133851
kgp2640905	21	33633516	NCRNA00159 | C21orf45	ENST00000565959	ENSE00002613058	33632115	33633896	1.311239274	0.085535043	0.389688074
kgp17106157	6	169434925	SMOC2 | THBS2	ENST00000419800	ENSE00001774129	169434292	169435155	1.616684572	0.085831649	0.389688074
kgp17993828	3	197171972	DLG1 | BDH1	ENST00000438408	ENSE00001728557	197171932	197172194	1.287053373	0.086294562	0.389688074
kgp5394371	14	75763124	FOS | JDP2	ENST00000558575	ENSE00002540211	75762823	75763341	-1.192492259	0.067935058	0.394219301
kgp25699814	16	19365822	LOC728276 | TMC5	ENST00000567369	ENSE00002590029	19365784	19366123	1.20475471	0.088226156	0.394260633
kgp25280684	1	143401987	LOC100131965 | LOC10013	ENST00000433980	ENSE00001631037	143401688	143402134	-1.162762917	0.078623253	0.399779251
kgp11153540	1	28974840	TAF12 | RNU11	ENST00000427804	ENSE00001796797	28974705	28975093	0.629500772	0.069234958	0.399840163
rs11884580	2	75156603	HK2 | POLE4	ENST00000377469	ENSE00001474023	75155366	75158663	1.307146531	0.091907581	0.401680607
rs502819	1	48532672	LOC388630 | SKINTL	ENST00000439795	ENSE00001721913	48532270	48532873	1.247390671	0.092565812	0.401680607
kgp27393600	9	93927608	LOC100128909 | AUH	ENST00000423719	ENSE00001710054	93925153	93927858	1.337773951	0.093324775	0.401680607
kgp30821269		1853054	ASMT |	ENST00000432272	ENSE00001752639	1853010	1853089	1.650713459	0.093631843	0.401680607
kgp5949693	15	96592837	LOC145820 | NR2F2	ENST00000558860	ENSE00002558494	96592504	96592924	-0.81964762	0.070112672	0.402268962
kgp2300525	10	5650458	CALML3 | ASB13	ENST00000425246	ENSE00001704738	5650438	5650694	0.771426133	0.07032208	0.402268962
kgp21809393	10	92262627	LOC119358 | HTR7	ENST00000414903	ENSE00001602435	92262543	92262725	1.185861912	0.095776641	0.403899634
kgp1774932	16	19365911	LOC728276 | TMC5	ENST00000567369	ENSE00002590029	19365784	19366123	-1.297353789	0.096032081	0.403899634
kgp24252794	2	10180331	UNQ5830 | KLF11	ENST00000567540	ENSE00002582289	10179219	10180790	-0.931802396	0.071317381	0.406038109
kgp34018	7	17496359	AHR | SNX13	ENST00000454003	ENSE00001770962	17496306	17496386	-0.451122444	0.072176878	0.406368248
kgp16400031	16	86119166	IRF8 | LOC732275	ENST00000563931	ENSE00002583061	86115015	86122279	0.696206918	0.072784442	0.406368248
kgp12327474	5	90607925	LOC729040 | ARRDC3	ENST00000513626	ENSE00002034273	90606838	90609046	-1.118473012	0.07284452	0.406368248
GA032252	17	27878898	TAOK1 | ABHD15	ENST00000562535	ENSE00002584590	27873905	27878921	-0.422504619	0.072882616	0.406368248
kgp763330	2	9898350	YWHAQ | TAF1B	ENST00000474667	ENSE00001869213	9897625	9899428	-0.473143944	0.073561809	0.406368248
kgp17399355	6	82555053	FAM46A | IBTK	ENST00000418567	ENSE00001802829	82554902	82555626	-0.660438504	0.07362084	0.406368248
kgp1534401	2	85291526	KCMF1 | LOC647302	ENST00000567718	ENSE00002604161	85291231	85291802	0.606028903	0.073732101	0.406368248
kgp4037313	16	1026640	LMF1 | LOC100287129	ENST00000565467	ENSE00002593914	1025761	1027029	-1.466445637	0.07424251	0.407321409
rs3902783	9	137475130	RXRA | COL5A1	ENST00000423455	ENSE00001806378	137475038	137477036	0.411281413	0.07464871	0.407696801
rs11695569	2	156877890	KCNJ3 | NR4A2	ENST00000428651	ENSE00001782847	156877047	156878411	-1.369910539	0.075140541	0.408197135
kgp11797315	6	24749242	C6orf62 | GMNN	ENST00000453179	ENSE00001719383	24749224	24749376	1.17585422	0.075876549	0.408197135
kgp9688833	3	188665111	LPP | LOC100132319	ENST00000444488	ENSE00001791567	188665068	188665428	0.423758806	0.076167156	0.408197135
kgp11526605	2	19226545	NT5C1B | OSR1	ENST00000424895	ENSE00001670652	19226396	19226726	-0.877722213	0.076254483	0.408197135
kgp28980815	8	102088389	YWHAZ | ZNF706	ENST00000514926	ENSE00002063679	102087397	102088479	0.505669998	0.076689043	0.408197135
kgp3913913	16	81995937	PLCG2 | SDR42E1	ENST00000564138	ENSE00002630315	81993524	81996298	-0.655257109	0.076782317	0.408197135
kgp9652931	10	128106335	ADAM12 | C10orf90	ENST00000456514	ENSE00001799229	128106285	128106377	-0.890474756	0.077331864	0.408197135
kgp22328002	5	148443214	SH3TC2 | ABLIM3	ENST00000515519	ENSE00002033933	148442880	148443237	0.550479174	0.077445852	0.408197135
kgp13203052	21	41099685	B3GALT5 | IGSF5	ENST00000457325	ENSE00001726936	41099682	41099919	-1.269614605	0.10019697	0.408749327
rs17291653	10	64063345	RTKN2 | ZNF365	ENST00000442753	ENSE00001705493	64063259	64063792	1.043742234	0.101198588	0.408749327
kgp11792777	4	185910278	ACSL1 | HELT	ENST00000505053	ENSE00002041220	185910151	185910374	-0.83702638	0.101568015	0.408749327
kgp9651302	8	123440771	HAS2AS | LOC100131552	ENST00000533992	ENSE00002155400	123440660	123440790	1.144614644	0.081906621	0.409533105
kgp5662856	12	16847071	LMO3 | LOC728622	ENST00000418574	ENSE00001540498	16846734	16848407	-1.214661739	0.103652285	0.412111494
kgp8682510	17	70026464	LOC124685 | SOX9	ENST00000538693	ENSE00002240848	70026432	70026992	-1.411517585	0.079402408	0.415430769
kgp6214704	9	33605019	LOC100288689 | ANXA2P2	ENST00000433357	ENSE00001611427	33605009	33605291	1.653405399	0.079777965	0.415430769
kgp4850930	14	21513239	RNASE7 | RNASE8	ENST00000554568	ENSE00002516697	21511515	21514097	0.389804708	0.079850819	0.415430769
kgp24958414	1	16787799	NECAP2 | CROCCL2	ENST00000457898	ENSE00001761634	16787443	16789782	-0.865314742	0.080363658	0.416304443
rs552631	18	77839092	C18orf22 | ADNP2	ENST00000566810	ENSE00002581238	77838683	77839140	-0.928711594	0.106693262	0.417493979
kgp23235364	21	46409962	C21orf70 | NCRNA00162	ENST00000439088	ENSE00001802548	46409779	46411747	0.965736764	0.107536328	0.417493979
kgp8900297	4	40332388	RHOH | CHRNA9	ENST00000510551	ENSE00002025439	40332177	40332436	1.596956855	0.101411411	0.422383451
kgp20691828	4	118280937	TRAM1L1 | LOC100288955	ENST00000416680	ENSE00001655896	118280884	118280965	0.699323275	0.082144785	0.422579268
kgp3729239	6	134824211	SGK1 | LOC645175	ENST00000456749	ENSE00001676049	134823926	134825157	0.583001265	0.082705339	0.422579268
kgp10080081	22	17156280	psiTPTE22 | XKR3	ENST00000457060	ENSE00001611736	17156013	17156342	-0.844192515	0.082967891	0.422579268
kgp4068529	5	1967041	IRX4 | IRX2	ENST00000511698	ENSE00002049647	1966914	1967077	0.601278969	0.082975382	0.422579268
kgp22761853		2532771	DHRSX | CD99	ENST00000445785	ENSE00001676508	2532758	2533388	-1.125243206	0.085971032	0.422808353
rs10524	1	41726473	SCMH1 | FOXO6	ENST00000425554	ENSE00001766482	41726415	41726545	-1.187503383	0.087531136	0.423537756
kgp11175295	14	50413317	ARF6 | C14orf182	ENST00000556130	ENSE00002433545	50412872	50413432	-1.611297355	0.104556231	0.424255275
kgp6944680	5	82215380	ATP6AP1L | TMEM167A	ENST00000504916	ENSE00002074263	82215195	82215781	1.712043454	0.105432606	0.424255275
rs12593159	15	63188295	TLN2 | TPM1	ENST00000557900	ENSE00002560171	63188011	63188342	1.096793403	0.106754798	0.424255275
kgp9483884	15	65011176	OAZ2 | RBPMS2	ENST00000560387	ENSE00002574073	65010265	65011801	-1.829191621	0.107227628	0.424255275
rs403829	9	106087037	CYLC2 | SMC2	ENST00000455051	ENSE00001794494	106086939	106087060	1.093856766	0.107711281	0.424255275
kgp16923525	6	19730747	RNF144B | ID4	ENST00000445568	ENSE00001687059	19730658	19731010	-1.617508922	0.107794464	0.424255275
kgp6793572	5	4515695	IRX1 | LOC340094	ENST00000503188	ENSE00002026322	4515436	4516889	-1.064230742	0.111124384	0.426407521
rs10195870	2	213784460	LOC646249 | IKZF2	ENST00000437261	ENSE00001626455	213784156	213784533	-1.198108578	0.089775177	0.427500841
kgp8744540	18	12775625	PSMG2 | PTPN2	ENST00000563722	ENSE00002613997	12774650	12775922	-1.073388396	0.084322198	0.427634004
rs7116965	11	46260696	PHF21A | CREB3L1	ENST00000530049	ENSE00002168074	46260290	46260818	1.144034823	0.110332	0.430294801
kgp21438550	19	35303421	ZNF599 | LOC401913	ENST00000561778	ENSE00002616079	35302738	35305249	0.728651101	0.085605589	0.431005426
kgp4292255	12	1612295	ERC1 | FBXL14	ENST00000515614	ENSE00002028096	1611627	1612636	1.045692296	0.08591345	0.431005426
kgp11078470	14	101424675	SNORD114-6 | SNORD114-1	ENST00000556637	ENSE00001803798	101424599	101424680	0.800190091	0.08605825	0.431005426
rs10993081	9	96939718	PTPDC1 | ZNF169	ENST00000416309	ENSE00001793816	96938884	96940013	-0.648701898	0.08701457	0.431119047
kgp15618123	1	234769004	IRF2BP2 | PP2672	ENST00000429269	ENSE00001771955	234768694	234770526	0.720721565	0.087317789	0.431119047
kgp9498825	20	29638329	FRG1B | DEFB115	ENST00000446917	ENSE00001782794	29638246	29638813	-0.820822644	0.087541178	0.431119047
kgp11211753	16	761180	LOC100287301 | METRN	ENST00000567096	ENSE00002610724	760938	761277	-0.500260277	0.08762789	0.431119047
kgp16296741	16	81419333	GAN | CMIP	ENST00000568107	ENSE00002626991	81416874	81424489	-1.625547616	0.088577263	0.431119047
kgp5248848	1	234770150	IRF2BP2 | PP2672	ENST00000429269	ENSE00001771955	234768694	234770526	0.738075792	0.088795879	0.431119047
kgp9447823	21	41099724	B3GALT5 | IGSF5	ENST00000457325	ENSE00001726936	41099682	41099919	-1.055083366	0.089098966	0.431119047
rs11712937	3	156800384	LEKR1 | CCNL1	ENST00000471357	ENSE00001879700	156799456	156801064	1.606811729	0.089111378	0.431119047
rs11132270	4	185925922	ACSL1 | HELT	ENST00000505053	ENSE00002024490	185925654	185926340	-1.138802396	0.089295577	0.431119047
kgp29100983	4	11771044	HS3ST1 | HSP90AB2P	ENST00000506195	ENSE00002077426	11770927	11771092	-1.14069824	0.092443836	0.433330482
rs12585178	13	74120735	KLF5 | LOC647281	ENST00000452852	ENSE00001591643	74120525	74120978	-0.940656388	0.114634984	0.434822353

kgp6459772	12	1612090	ERC1 | FBXL14	ENST00000515614	ENSE00002028096	1611627	1612636	0.651626847	0.09094162	0.435341254
kgp1689967	3	149102219	LOC100289491 | TM4SF4	ENST00000462931	ENSE00001946706	149101966	149102790	-0.764391148	0.091162466	0.435341254
kgp9916948	16	1026094	LMF1 | LOC100287129	ENST00000568394	ENSE00002595585	1025761	1026400	-1.355703751	0.091252144	0.435341254
kgp10515776	14	50410528	ARF6 | C14orf182	ENST00000556913	ENSE00002499815	50408654	50410610	-0.716738369	0.116154593	0.435579724
kgp8569278	11	65211979	NCRNA00084 | MALAT1	ENST00000501122	ENSE00001961965	65190269	65213011	-0.779774799	0.092004583	0.437202878
rs17645573	3	44160183	ABHD5 | LOC375337	ENST00000568686	ENSE00002586185	44158791	44163857	0.893507077	0.120532855	0.43808375
kgp6651504	4	150076761	NR3C2 | LOC100287246	ENST00000503100	ENSE00002056626	150076604	150076877	-1.732494738	0.122593043	0.43808375
kgp4137720	1	173832447	DARS2 | GAS5	ENST00000416578	ENSE00001660333	173832364	173833079	-0.972477871	0.12281099	0.43808375
kgp16826896	13	44711363	LOC121838 | LOC10028773	ENST00000432331	ENSE00001755361	44711239	44711372	0.692983894	0.123208716	0.43808375
kgp4614732	14	21513028	RNASE7 | RNASE8	ENST00000554568	ENSE00002516697	21511515	21514097	0.732519189	0.124447672	0.43808375
rs12447206	16	85319399	TMEM148 | KIAA0182	ENST00000366314	ENSE00001315394	85318604	85319569	1.049870986	0.12553318	0.43808375
kgp63849	16	63402942	CDH8 | CDH11	ENST00000561595	ENSE00002622759	63402748	63403113	0.708531874	0.12772862	0.43808375
kgp10722569	3	193924054	HES1 | LOC100131551	ENST00000432908	ENSE00001803637	193924037	193924096	-0.781717508	0.128629357	0.43808375
kgp10146854	2	38466599	C2orf58 | ATL2	ENST00000450854	ENSE00001733174	38466200	38466732	-0.959760025	0.128939212	0.43808375
kgp3719269	18	77837728	C18orf22 | ADNP2	ENST00000564012	ENSE00002624040	77837589	77838211	-0.817308891	0.130130349	0.43808375
kgp464033	14	38781016	CLEC14A | LOC283547	ENST00000555636	ENSE00002455148	38780991	38781134	-1.265893627	0.131425125	0.43808375
rs17663749	11	107185075	GUCY1A2 | CWF19L2	ENST00000561746	ENSE00002621788	107182858	107186997	-0.731367592	0.09307901	0.438170352
kgp5282430	11	82314959	LOC100288816 | FAM181B	ENST00000527364	ENSE00002181359	82314785	82315143	0.674901472	0.093135564	0.438170352
kgp6123103	14	91294397	TTC7B | LOC283588	ENST00000555975	ENSE00002479564	91294333	91294472	0.427140481	0.093768572	0.438170352
rs7537211	1	182058556	ZNF648 | LOC100130996	ENST00000428646	ENSE00001688250	182058500	182059247	0.648078353	0.093890078	0.438170352
kgp12334337	4	8358437	HTRA3 | ACOX3	ENST00000505448	ENSE00002069127	8357038	8359103	-0.579279089	0.094248241	0.438170352
rs2282579	11	130735482	C11orf44 | SNX19	ENST00000525716	ENSE00002194756	130735365	130737889	-0.763140493	0.094613665	0.438170352
kgp11432932	17	70020440	LOC124685 | SOX9	ENST00000430908	ENSE00001702183	70020294	70020445	-1.575401307	0.095169681	0.438170352
kgp19471340	14	39572929	SEC23A | SIP1	ENST00000557350	ENSE00002472340	39572749	39573016	-0.504353889	0.095217865	0.438170352
kgp14668068	2	806175	LOC100128185 | LOC39134	ENST00000415700	ENSE00001684242	806037	806257	-1.185472676	0.096058583	0.438170352
rs936523	5	111064296	STARD4 | C5orf13	ENST00000500779	ENSE00001972809	111064234	111064457	0.623686957	0.096129709	0.438170352
kgp22255081	5	116790994	LOC100287135 | DTWD2	ENST00000504107	ENSE00002069187	116790959	116791150	-1.660707088	0.09662148	0.438170352
kgp9892084	5	52410814	LOC257396 | FST	ENST00000502171	ENSE00001985169	52409689	52410953	-0.489289781	0.096666319	0.438170352
kgp22106863	5	1958881	IRX4 | IRX2	ENST00000513419	ENSE00002028240	1958696	1959015	0.417719003	0.097235779	0.438170352
GA009930	11	65212447	NCRNA00084 | MALAT1	ENST00000501122	ENSE00001961965	65190269	65213011	-1.746773821	0.097651949	0.438170352
rs11159706	14	85995687	SEL1L | FLRT2	ENST00000380722	ENSE00001486021	85994961	85996332	0.530663797	0.098155139	0.438170352
kgp3005683	3	188659783	LPP | LOC100132319	ENST00000444488	ENSE00001595578	188659504	188659839	0.405965796	0.098615938	0.438170352
kgp12501620	15	63191602	TLN2 | TPM1	ENST00000557900	ENSE00002572006	63190850	63191742	0.823161562	0.098700405	0.438170352
kgp24545282	2	806176	LOC100128185 | LOC39134	ENST00000415700	ENSE00001684242	806037	806257	-1.223584223	0.098742615	0.438170352
kgp26385317	6	30759596	IER3 | DDR1	ENST00000439406	ENSE00001648072	30759203	30760027	0.922416111	0.099179124	0.438495246
kgp3986189	22	27261517	MIAT | MN1	ENST00000434868	ENSE00001619874	27258220	27261749	-0.984860278	0.134785617	0.440031128
rs3740427	10	130713288	MKI67 | MGMT	ENST00000446589	ENSE00001801032	130713265	130713429	-0.728124623	0.13651623	0.440031128
kgp22535709	5	175612232	LOC643201 | C5orf25	ENST00000508056	ENSE00002083677	175612104	175612345	0.948937205	0.137786937	0.440031128
kgp7068255	14	21388397	LOC643332 | RNASE2	ENST00000555624	ENSE00002479155	21388166	21388458	-0.682423132	0.138646516	0.440031128
kgp1481251	11	34595436	ELF5 | EHF	ENST00000527135	ENSE00002160386	34595094	34595529	-0.727374105	0.138676477	0.440031128
kgp2479558	3	186195998	DGKG | CRYGS	ENST00000456535	ENSE00001761039	186195874	186196159	1.14539623	0.114451718	0.441645213
rs394327	4	104473722	CENPE | TACR3	ENST00000509399	ENSE00002051208	104473665	104473743	1.285955988	0.115662278	0.441645213
rs6711574	2	99378801	MGAT4A | C2orf55	ENST00000419865	ENSE00001740570	99378401	99379041	-1.218751975	0.116727158	0.441645213
kgp9982130	1	76484827	ASB17 | ST6GALNAC3	ENST00000436121	ENSE00001700878	76484819	76485041	1.075642112	0.117360266	0.441645213
rs680413	11	65190379	FRMD8 | NCRNA00084	ENST00000501122	ENSE00001961965	65190269	65213011	-0.499758107	0.101097421	0.443091007
kgp12382430	8	19546254	CSGALNACT1 | INTS10	ENST00000519803	ENSE00002130675	19546235	19546445	-0.786396711	0.101152104	0.443091007
kgp19580540	14	85995127	SEL1L | FLRT2	ENST00000380722	ENSE00001486021	85994961	85996332	0.49952154	0.101575852	0.443091007
rs2546890	5	158759900	IL12B | LOC285627	ENST00000521472	ENSE00002061392	158759790	158760011	0.654007478	0.101687	0.443091007
kgp26343947	6	109090626	FOXO3 | ARMC2	ENST00000448744	ENSE00001698309	109089594	109090773	-0.739192241	0.141393993	0.443493602
kgp12117403	2	43329664	HAAO | ZFP36L2	ENST00000434020	ENSE00001640903	43329173	43329829	0.67092574	0.144419685	0.443493602
kgp23485017	7	130122526	TSGA14 | MEST	ENST00000562524	ENSE00002587446	130121332	130124233	-0.747675017	0.14620322	0.443493602
kgp29822029	10	116539415	ABLIM1 | FAM160B1	ENST00000436932	ENSE00001725826	116537710	116539662	-1.142316959	0.147991586	0.443493602
kgp8499190	2	139357374	SPOPL | NXPH2	ENST00000562796	ENSE00002608781	139357233	139359996	-0.767732557	0.1490724	0.443493602
kgp9634455	21	25333463	NCAM2 | NCRNA00158	ENST00000447405	ENSE00001601833	25333220	25333903	-1.040047284	0.14928121	0.443493602
kgp8047261	22	27316435	MIAT | MN1	ENST00000438113	ENSE00001597568	27316340	27316574	-0.956900121	0.150470202	0.443493602
kgp11092387	6	109089649	FOXO3 | ARMC2	ENST00000448744	ENSE00001698309	109089594	109090773	-0.645640665	0.152391452	0.443493602
kgp13171636	21	32932789	LOC150051 | SOD1	ENST00000433071	ENSE00001760430	32932330	32932800	0.775868466	0.152908795	0.443493602
kgp807262	5	10137878	LOC285692 | FAM173B	ENST00000506299	ENSE00002031624	10137664	10138477	-0.900660885	0.15390766	0.443493602
rs11124635	2	38466625	C2orf58 | ATL2	ENST00000450854	ENSE00001733174	38466200	38466732	-0.832174312	0.155458015	0.443493602
kgp414257	7	157075319	UBE3C | DNAJB6	ENST00000442017	ENSE00001628506	157074838	157075380	-0.800692976	0.155894721	0.443493602
kgp16337578	16	81417975	GAN | CMIP	ENST00000568107	ENSE00002626991	81416874	81424489	-1.113229559	0.102221682	0.443818597
kgp1486210	5	43015122	LOC100288522 | LOC64898	ENST00000503152	ENSE00002079428	43014838	43015252	0.42993257	0.102683997	0.444227903
kgp18248936	9	37079950	LOC100287249 | ZCCHC7	ENST00000430809	ENSE00001660879	37079932	37080035	-0.745906832	0.103382624	0.445013293
kgp1188341	4	158587989	LOC340017 | C4orf18	ENST00000507296	ENSE00002024944	158587823	158588196	-1.361839775	0.103900278	0.445013293
rs6084040	20	25989665	FAM182B | LOC100134868	ENST00000448580	ENSE00001605646	25989427	25989691	-0.702949997	0.103971623	0.445013293
rs745526	1	38675803	POU3F1 | LOC400750	ENST00000431311	ENSE00001646865	38674706	38676494	1.097834691	0.096548407	0.445608032
rs10018622	4	39135564	KLHL5 | WDR19	ENST00000507579	ENSE00002042591	39135533	39135665	0.414533747	0.105597386	0.44732888
rs11105136	12	89406493	KITLG | DUSP6	ENST00000500381	ENSE00001976792	89404458	89407094	0.738668312	0.105733226	0.44732888
kgp12911902	11	107185504	GUCY1A2 | CWF19L2	ENST00000561746	ENSE00002621788	107182858	107186997	-0.955025951	0.106223875	0.44732888
kgp6796426	11	78140792	GAB2 | NARS2	ENST00000513207	ENSE00002020056	78140746	78140882	-0.552054072	0.10622451	0.44732888
rs3749376	3	24538267	THRB | LOC644990	ENST00000438096	ENSE00001661734	24538155	24538672	-0.48792584	0.106365691	0.44732888
kgp14773055	2	95539242	LOC100289554 | TEKT4	ENST00000568768	ENSE00002592719	95539134	95539294	-0.715787768	0.164059864	0.448296603
rs10831728	11	12110677	DKK3 | MICAL2	ENST00000527997	ENSE00002178930	12110669	12110988	0.744469351	0.164246066	0.448296603
rs8040523	15	30297758	LOC727808 | LOC10012843	ENST00000561392	ENSE00002564316	30297646	30297992	0.649030844	0.164750077	0.448296603
rs4930714	12	124067197	RILPL1 | TMED2	ENST00000498967	ENSE00002259901	124066767	124067714	-1.146146982	0.165529165	0.448296603
kgp15305294	1	87820606	LMO4 | PKN2	ENST00000452509	ENSE00001793610	87820527	87820881	-0.692081369	0.166346126	0.448296603
rs10902661	1	25908241	LDLRAP1 | MAN1C1	ENST00000412797	ENSE00001796972	25907969	25908263	-0.695447249	0.166478743	0.448296603
rs10137819	14	101394941	SNORD113-2 | SNORD113-	ENST00000553465	ENSE00002494508	101394436	101394952	0.772401445	0.16772169	0.448296603
kgp27368706	9	129281012	FAM125B | LMX1B	ENST00000454034	ENSE00001806230	129280634	129281087	0.683270484	0.168450845	0.448296603
rs9597282	13	33929518	STARD13 | RFC3	ENST00000454681	ENSE00001707731	33929343	33929803	-0.700915161	0.171222331	0.449204966
rs271740	1	234664631	TARBP1 | IRF2BP2	ENST00000435574	ENSE00001674386	234663637	234666088	1.061891941	0.173610251	0.449204966
kgp11649707	14	95801653	CLMN | C14orf139	ENST00000554522	ENSE00002482888	95800366	95801841	0.757439998	0.175158433	0.449204966
kgp10449840	2	9247424	MBOAT2 | ASAP2	ENST00000565044	ENSE00002602192	9246722	9249994	-0.895643105	0.176036339	0.449204966
kgp25563987	16	83831552	CDH13 | HSBP1	ENST00000567109	ENSE00002630405	83831266	83834245	0.797375749	0.176334051	0.449204966
kgp4238370	16	83831735	CDH13 | HSBP1	ENST00000567109	ENSE00002630405	83831266	83834245	0.79644853	0.177488326	0.449204966
kgp22375190	5	10137910	LOC285692 | FAM173B	ENST00000506299	ENSE00002031624	10137664	10138477	-0.774708441	0.178320759	0.449204966

kgp1955953	15	39481940	LOC100289563 | C15orf54	ENST00000560743	ENSE00002543139	39480639	39482367	1.147438304	0.09887341	0.449424592
kgp27279882	9	132044872	IER5L | C9orf106	ENST00000455981	ENSE00001702211	132044737	132044971	-1.103140769	0.100955426	0.449855667
kgp1321487	5	82216798	ATP6AP1L | TMEM167A	ENST00000504916	ENSE00002026199	82216781	82216938	1.076383555	0.102916742	0.449855667
kgp30193552	5	6826800	POLS | LOC442132	ENST00000508881	ENSE00002063489	6826756	6826841	1.224511965	0.106579998	0.449855667
kgp11937175	17	13836393	HS3ST3A1 | CDRT15P	ENST00000457609	ENSE00001754385	13836393	13836502	1.137603095	0.107554924	0.449855667
kgp19787448	15	96939402	LOC728800 | SPATA8	ENST00000560242	ENSE00002545323	96939285	96939415	-1.105272117	0.107773673	0.449855667
rs11134055	5	4516670	IRX1 | LOC340094	ENST00000503188	ENSE00002026322	4515436	4516889	-1.155987946	0.10796536	0.449855667
kgp7352382	16	81420058	GAN | CMIP	ENST00000568107	ENSE00002626991	81416874	81424489	-0.771690862	0.107489873	0.45003384
kgp14792508	2	780091	LOC100128185 | LOC39134	ENST00000414556	ENSE00001725567	779837	780588	-1.157099494	0.10792303	0.45003384
kgp26736927	3	156800001	LEKR1 | CCNL1	ENST00000471357	ENSE00001879700	156799456	156801064	0.451352131	0.108919512	0.45003384
rs12280900	11	71412414	KRTAP5-11 | FAM86C	ENST00000532530	ENSE00002171966	71412314	71412531	0.515310645	0.109161757	0.45003384
rs1017923	5	1967265	IRX4 | IRX2	ENST00000511698	ENSE00002081037	1967191	1967268	0.740756049	0.109597752	0.45003384
kgp5795390	5	52410868	LOC257396 | FST	ENST00000502171	ENSE00001985169	52409689	52410953	-0.789047838	0.109766082	0.45003384
rs603834	7	42942126	GLI3 | C7orf25	ENST00000569883	ENSE00002599349	42940871	42942238	-0.479311411	0.10982372	0.45003384
rs7779460	7	116213679	LOC100287239 | MET	ENST00000458082	ENSE00001754833	116213539	116213798	0.958941472	0.109991701	0.45003384
kgp17943226	3	149957922	TMEM183B | TSC22D2	ENST00000498005	ENSE00001866947	149957667	149957996	0.944708599	0.111142978	0.453208021
kgp18892342	12	52206402	FLJ33996 | FIGNL2	ENST00000562518	ENSE00002618150	52203789	52206648	0.759692915	0.183325958	0.458314895
rs1047955	17	70080082	LOC124685 | SOX9	ENST00000419257	ENSE00001718229	70079973	70080102	-0.781226325	0.113421451	0.459442918
kgp25144997	1	234769843	IRF2BP2 | PP2672	ENST00000429269	ENSE00001771955	234768694	234770526	0.685336453	0.113592258	0.459442918
kgp4009529	12	114184172	LHX5 | RBM19	ENST00000550223	ENSE00002363302	114183509	114184400	-0.776205911	0.114014273	0.459442918
kgp16275563	16	81995974	PLCG2 | SDR42E1	ENST00000564138	ENSE00002630315	81993524	81996298	-0.63969298	0.114194594	0.459442918
rs2339989	5	173146043	BOD1 | CPEB4	ENST00000521128	ENSE00002122678	173145844	173146695	1.245099082	0.11186979	0.459738863
kgp25674002	16	1026300	LMF1 | LOC100287129	ENST00000568394	ENSE00002595585	1025761	1026400	-1.311008228	0.114741463	0.460109453
rs12598147	16	81995177	PLCG2 | SDR42E1	ENST00000564138	ENSE00002630315	81993524	81996298	-1.055721639	0.115422252	0.461306816
kgp8363402	1	232314708	DISC1 | SIPA1L2	ENST00000441459	ENSE00001701526	232314543	232315859	0.598993347	0.185943842	0.46136442
kgp17446460	6	19749908	RNF144B | ID4	ENST00000450310	ENSE00001785182	19749879	19749989	-2.061175678	0.124523135	0.461891094
kgp10740526	6	4495520	PECI | KU-MEL-3	ENST00000563079	ENSE00002597644	4494116	4496003	1.454433486	0.125634112	0.461891094
kgp9996903	9	93763628	SYK | LOC100128909	ENST00000563268	ENSE00002600916	93763052	93764153	1.045754147	0.126279192	0.461891094
kgp14596811	2	174746372	LOC643997 | SP3	ENST00000418620	ENSE00001593303	174746253	174746418	1.01730217	0.127550235	0.461891094
rs3947590	4	27211078	STIM2 | PCDH7	ENST00000382007	ENSE00001490576	27209127	27211372	1.508070786	0.130428483	0.461891094
kgp4326980	11	74954168	LOC441617 | ARRB1	ENST00000562197	ENSE00002609763	74953064	74954742	1.052750176	0.130547105	0.461891094
rs11583460	1	17521930	LOC400743 | PADI1	ENST00000539219	ENSE00002261175	17520556	17524112	-1.017101644	0.130571884	0.461891094
kgp22736401	2	95533605	ANKRD20B | LOC10028955	ENST00000568768	ENSE00002576720	95533231	95539050	1.006783307	0.132033479	0.461891094
rs1556002	10	21657474	LOC100128511 | C10orf114	ENST00000433460	ENSE00001607392	21657426	21657484	1.048432721	0.132430314	0.461891094
kgp11146746	15	93323470	LOC643797 | CHD2	ENST00000562894	ENSE00002583293	93322987	93324722	0.525115143	0.116010869	0.462129105
kgp7470516	11	69867047	LOC100127946 | ANO1	ENST00000528507	ENSE00002193800	69866989	69867165	1.24020489	0.18810113	0.463234126
rs4927807	3	195677895	LOC727978 | SDHALP1	ENST00000570130	ENSE00002622318	195676059	195679566	-0.762171714	0.116825705	0.463844164
kgp20251725	8	54429009	OPRK1 | ATP6V1H	ENST00000426023	ENSE00002169965	54427731	54429514	-0.56112245	0.117561366	0.46523465
kgp23290950	21	46414549	C21orf70 | NCRNA00162	ENST00000569966	ENSE00002621024	46414277	46415100	0.539759457	0.118955089	0.467562071
kgp33193	1	189005998	PLA2G4A | FAM5C	ENST00000445072	ENSE00001699193	189005849	189006393	0.441972496	0.119650835	0.467562071
rs3072	2	20878406	GDF7 | C2orf43	ENST00000565841	ENSE00002602242	20877569	20879005	0.425622734	0.1198558	0.467562071
kgp11161988	2	43329803	HAAO | ZFP36L2	ENST00000434020	ENSE00001640903	43329173	43329829	0.459546119	0.119912394	0.467562071
kgp10176500	2	208095430	KLF7 | CREB1	ENST00000448786	ENSE00001762022	208095248	208095445	0.831383258	0.120086365	0.467562071
kgp6278098	4	178612208	AGA | LOC285501	ENST00000503093	ENSE00002030481	178612159	178612222	0.473173138	0.121364908	0.468985561
rs827631	10	9015230	GATA3 | SFTA1P	ENST00000456526	ENSE00001747623	9014903	9015431	-0.929471604	0.121545534	0.468985561
kgp22745857	2	71117052	CD207 | VAX2	ENST00000449073	ENSE00002473498	71116951	71117089	-0.771991091	0.122072039	0.468985561
kgp10513811	12	126845352	TMEM132B | LOC10028870	ENST00000536639	ENSE00002273467	126844647	126845611	-0.881011686	0.122358856	0.468985561
kgp3441924	17	17093532	MPRIP | PLD6	ENST00000567268	ENSE00002611064	17091982	17095962	-0.971370451	0.122394741	0.468985561
kgp4309426	1	92865697	RPAP2 | GFI1	ENST00000564442	ENSE00002592577	92864531	92867613	-1.333574012	0.123260879	0.470809749
kgp4521769	2	140235206	LOC647012 | LRP1B	ENST00000421326	ENSE00001637803	140235062	140235317	0.98810919	0.138060902	0.472640722
kgp27353649	9	129281087	FAM125B | LMX1B	ENST00000454034	ENSE00001806230	129280634	129281087	1.001653967	0.138511883	0.472640722
kgp6764638	5	6796418	POLS | LOC442132	ENST00000508881	ENSE00002021213	6795993	6796458	0.973489921	0.139747946	0.472640722
rs2808699	9	100505830	XPA | FOXE1	ENST00000562653	ENSE00002577170	100505490	100507217	0.972362828	0.140826741	0.472640722
kgp6082721	10	47639917	LOC100133189 | LOC10028	ENST00000543148	ENSE00002314176	47639863	47639922	-1.279566888	0.141927974	0.472640722
kgp24491057	2	75157029	HK2 | POLE4	ENST00000377469	ENSE00001474023	75155366	75158663	1.046487831	0.142919006	0.472640722
kgp14412334	2	75156998	HK2 | POLE4	ENST00000377469	ENSE00001474023	75155366	75158663	0.95829232	0.143905513	0.472640722
rs4959299	6	4492079	PECI | KU-MEL-3	ENST00000563079	ENSE00002584191	4492041	4492424	0.953500863	0.144326188	0.472640722
kgp2131784	2	806080	LOC100128185 | LOC39134	ENST00000415700	ENSE00001684242	806037	806257	-0.952756408	0.125521816	0.472877439
kgp15359536	1	219395779	LYPLAL1 | LOC728510	ENST00000420237	ENSE00001793926	219395590	219396529	-0.576254452	0.125522088	0.472877439
kgp9850306	15	36003975	ATPBD4 | LOC100288892	ENST00000559210	ENSE00002539117	36003908	36003992	0.50366474	0.125905419	0.472877439
kgp17913216	3	195678537	LOC727978 | SDHALP1	ENST00000570130	ENSE00002622318	195676059	195679566	-0.927223642	0.126019266	0.472877439
kgp729334	4	8358995	HTRA3 | ACOX3	ENST00000505448	ENSE00002069127	8357038	8359103	-1.289295542	0.126255707	0.472877439
kgp3002758	16	21806465	OTOA | LOC730092	ENST00000567370	ENSE00002577410	21805416	21807080	0.514748446	0.126416379	0.472877439
kgp11906527	2	8861745	ID2 | KIDINS220	ENST00000569008	ENSE00002625681	8861264	8862816	-0.507194534	0.126785214	0.472877439
kgp20430629	8	19546424	CSGALNACT1 | INTS10	ENST00000519803	ENSE00002130675	19546235	19546445	-1.250088258	0.127176884	0.472877439
rs9803801	1	234769793	IRF2BP2 | PP2672	ENST00000429269	ENSE00001771955	234768694	234770526	0.641688875	0.127328225	0.472877439
kgp1261714	3	30566565	RBMS3 | TGFBR2	ENST00000431057	ENSE00001758672	30566482	30566939	2.302316695	0.145728001	0.473616003
kgp12069110	10	73638474	PSAP | CHST3	ENST00000441348	ENSE00001621547	73638471	73638865	0.526554512	0.128462697	0.47418085
kgp1506344	8	128032277	FAM84B | POU5F1B	ENST00000561978	ENSE00002619091	128031889	128033259	-0.76169558	0.129202972	0.47418085
kgp24622402	2	10180168	UNQ5830 | KLF11	ENST00000567540	ENSE00002582289	10179219	10180790	-0.621155225	0.129243867	0.47418085
GA028063	11	65207975	NCRNA00084 | MALAT1	ENST00000501122	ENSE00001961965	65190269	65213011	-0.426719574	0.129595495	0.47418085
kgp16429166	16	23681963	DCTN5 | PLK1	ENST00000566996	ENSE00002576769	23681332	23683518	-0.664926242	0.129970432	0.47418085
kgp1596693	5	106346707	RAB9P1 | LOC100287833	ENST00000513273	ENSE00002038419	106346641	106346715	-0.986415416	0.130036339	0.47418085
rs740539	7	156398403	C7orf4 | C7orf13	ENST00000439364	ENSE00001693490	156398352	156398461	-0.613958642	0.194292464	0.474937134
kgp9771059	8	54436244	OPRK1 | ATP6V1H	ENST00000426023	ENSE00001712348	54435889	54436491	-0.417025799	0.130825206	0.475620551
kgp5491579	18	12774782	PSMG2 | PTPN2	ENST00000563722	ENSE00002613997	12774650	12775922	-1.210849454	0.117872712	0.477862346
kgp16797251	13	34185598	STARD13 | RFC3	ENST00000442716	ENSE00001655744	34185328	34185659	-0.949160914	0.134770156	0.479157849
kgp569784	10	38536882	LOC100129055 | LOC10013	ENST00000450980	ENSE00001732551	38536850	38536993	0.401558884	0.135400736	0.479157849
kgp25612195	16	70611559	SF3B3 | IL34	ENST00000562874	ENSE00002580772	70611221	70611571	-0.383809738	0.13540423	0.479157849
rs16884265	5	10196616	LOC285692 | FAM173B	ENST00000566945	ENSE00002600148	10195233	10197740	-0.901230497	0.135606397	0.479157849
kgp3854969	1	21742816	ECE1 | LOC100289081	ENST00000423921	ENSE00001597153	21742815	21743112	-0.684708596	0.135830359	0.479157849
kgp21624166	10	47096983	PPYR1 | LOC728643	ENST00000422732	ENSE00001719699	47096454	47098649	0.395482267	0.136113201	0.479157849
rs11227296	11	65538931	LOC100288054 | DKFZp761	ENST00000534505	ENSE00002188507	65538663	65539056	-1.02490642	0.136277384	0.479157849
kgp8371846	9	3691687	RFX3 | GLIS3	ENST00000427722	ENSE00001728381	3691504	3691814	-0.664796378	0.137624634	0.479157849
kgp8409130	13	40918250	COG6 | LOC646982	ENST00000400432	ENSE00001550801	40918094	40918342	-0.784742574	0.137727332	0.479157849
kgp11748560	21	36511081	RUNX1 | SETD4	ENST00000455028	ENSE00001764112	36511034	36511519	0.381022469	0.137866525	0.479157849

GA016281	19	36822469	ZNF146 | ZFP14	ENST00000438368	ENSE00001799972	36822349	36822602	-1.350440672	0.138240796	0.479157849
kgp455711	21	46414470	C21orf70 | NCRNA00162	ENST00000569966	ENSE00002621024	46414277	46415100	0.471773193	0.138290158	0.479157849
kgp1763108	7	156397966	C7orf4 | C7orf13	ENST00000439364	ENSE00002600631	156397882	156398074	-0.497751472	0.138421452	0.479157849
kgp19583155	14	62031923	PRKCH | HIF1A	ENST00000553288	ENSE00002522136	62031837	62031959	0.494793811	0.13851569	0.479157849
kgp10586676	2	89110282	FLJ40330 | LOC100132330	ENST00000452230	ENSE00001624389	89109984	89111209	-1.516932241	0.13860185	0.479157849
kgp5157008	12	31521702	FAM60A | DENND5B	ENST00000541749	ENSE00002274720	31521683	31522235	0.392271688	0.138684368	0.479157849
rs971150	12	126845050	TMEM132B | LOC10028870	ENST00000536639	ENSE00002273467	126844647	126845611	-0.857133116	0.138789025	0.479157849
rs17281076		152691123	ZFP92 | TREX2	ENST00000569962	ENSE00002593900	152689210	152691934	0.342538845	0.138995802	0.479157849
rs3812145	6	82555016	FAM46A | IBTK	ENST00000418567	ENSE00001802829	82554902	82555626	-0.490023889	0.139340849	0.479157849
kgp2998578	17	48292128	COL1A1 | TMEM92	ENST00000514468	ENSE00002068391	48292055	48292136	1.005666961	0.122777869	0.481941884
kgp13091355	21	41102475	B3GALT5 | IGSF5	ENST00000457325	ENSE00001686404	41101431	41102607	-1.09314388	0.123287277	0.481941884
kgp9622938	14	41444636	FBXO33 | LRFN5	ENST00000515218	ENSE00002067081	41444546	41445082	-1.028654147	0.123698417	0.481941884
rs12906432	15	101414167	LOC145757 | ALDH1A3	ENST00000558641	ENSE00002572542	101414028	101414195	-1.128389492	0.140624703	0.482198911
kgp19912353	15	93323386	LOC643797 | CHD2	ENST00000562894	ENSE00002583293	93322987	93324722	0.402746512	0.141088164	0.482417603
kgp4181515	8	102087549	YWHAZ | ZNF706	ENST00000514926	ENSE00002063679	102087397	102088479	0.887735494	0.198970108	0.482795115
kgp7351457	16	86755470	FOXL1 | LOC100288525	ENST00000563331	ENSE00002578897	86755371	86755560	-0.982101815	0.141898305	0.483657247
kgp3912614	20	37034694	LBP | LOC388796	ENST00000422519	ENSE00001803279	37034658	37034969	0.486262252	0.142389054	0.483657247
rs10031209	4	11773427	HS3ST1 | HSP90AB2P	ENST00000510639	ENSE00002053018	11773329	11773508	-1.519247305	0.142652842	0.483657247
kgp22329244	5	108655701	FER | PJA2	ENST00000512693	ENSE00002023745	108655460	108656336	1.277533716	0.150310655	0.484231003
kgp4480159	18	77827192	C18orf22 | ADNP2	ENST00000568911	ENSE00002622433	77822752	77827308	-0.948867049	0.151251642	0.484231003
rs11246068	11	327334	IFITM3 | B4GALNT4	ENST00000534483	ENSE00002157743	327171	327736	0.954524379	0.152691015	0.485218115
kgp4298286	12	3405902	TSPAN9 | LOC100128253	ENST00000505276	ENSE00002256861	3405368	3407426	1.100835947	0.12685361	0.487898498
kgp6631667	5	114541352	TRIM36 | PGGT1B	ENST00000507241	ENSE00002051389	114539713	114541943	-0.416459559	0.145077463	0.490076455
kgp5559317	12	3434009	LOC100128253 | PRMT8	ENST00000432994	ENSE00002259013	3432515	3434509	0.367934165	0.145358219	0.490076455
kgp16354985	16	66517023	BEAN | FLJ27243	ENST00000544589	ENSE00002208571	66516775	66519747	-0.649371719	0.204077034	0.490139032
kgp9585560	8	130960915	FAM49B | ASAP1	ENST00000520146	ENSE00002129601	130960795	130960944	-0.656849499	0.204967232	0.490139032
kgp13956477	17	69592588	LOC100133226 | LOC12468	ENST00000442627	ENSE00001722368	69592479	69592707	0.997124597	0.135303287	0.490900166
kgp6394330	9	93764114	SYK | LOC100128909	ENST00000563268	ENSE00002600916	93763052	93764153	1.41683651	0.13570334	0.490900166
rs6770717	3	20431544	SGOL1 | VENTXP7	ENST00000566804	ENSE00002605809	20429741	20432054	-0.965428941	0.135870649	0.490900166
kgp18751657	12	68323021	DYRK2 | IFNG	ENST00000545520	ENSE00002285526	68323015	68323167	1.231308083	0.138074448	0.490900166
kgp8612898	18	12775295	PSMG2 | PTPN2	ENST00000563722	ENSE00002613997	12774650	12775922	-1.119910999	0.139191012	0.490900166
kgp933302	14	71100550	MED6 | TTC9	ENST00000500016	ENSE00002608293	71100479	71100575	-1.119740416	0.139987678	0.490900166
kgp4614866	8	54308503	OPRK1 | ATP6V1H	ENST00000521558	ENSE00002131352	54308459	54308506	-1.34604945	0.140415948	0.490900166
kgp703908	18	3350498	MYL12B | TGIF1	ENST00000558690	ENSE00002571551	3350485	3350557	0.947460666	0.142022606	0.490900166
kgp12200116	2	235370647	LOC100287159 | ARL4C	ENST00000418025	ENSE00001670233	235370511	235370771	0.967036444	0.143569031	0.490900166
kgp4288146	9	26801837	TUSC1 | C9orf82	ENST00000521572	ENSE00002109868	26801731	26802058	-0.968514172	0.143997382	0.490900166
kgp1120751	9	111014459	LOC100128657 | ACTL7B	ENST00000453455	ENSE00001750142	111014434	111014548	0.495929446	0.146282808	0.491819913
rs1232138		8832017	FAM9A | FAM9B	ENST00000440430	ENSE00001673495	8831902	8832133	0.925202449	0.156310081	0.493066358
kgp4025455	1	23608567	HTR1D | HNRNPR	ENST00000566551	ENSE00002595853	23607802	23613245	-0.344998829	0.147173438	0.493439831
kgp12484286	6	10452237	C6orf218 | GCNT2	ENST00000449333	ENSE00001788112	10452136	10452480	0.562537952	0.210881667	0.494375948
kgp5561714	4	76007375	DKFZP564O0823 | RCHY1	ENST00000567197	ENSE00002629844	76007135	76007705	1.227430227	0.213165686	0.494375948
kgp2504943	4	95664988	PDLIM5 | BMPR1B	ENST00000510795	ENSE00002074054	95664951	95665245	0.949267937	0.213806397	0.494375948
rs2244398	1	235097172	PP2672 | TOMM20	ENST00000458044	ENSE00001789725	235097123	235097313	0.560202759	0.214068014	0.494375948
kgp11646827	5	159204748	LOC285627 | ADRA1B	ENST00000522627	ENSE00002140147	159203779	159205694	0.71553034	0.215531309	0.494375948
kgp18624272	9	137475140	RXRA | COL5A1	ENST00000423455	ENSE00001806378	137475038	137477036	0.661862145	0.215727687	0.494375948
kgp3163864	8	127341177	LOC650095 | FAM84B	ENST00000500180	ENSE00001990119	127340553	127341780	0.623024275	0.147982943	0.494779534
kgp1815990	8	19546319	CSGALNACT1 | INTS10	ENST00000519803	ENSE00002130675	19546235	19546445	-1.120688247	0.148583444	0.495414963
rs28361590	9	132121725	C9orf106 | LOC100128077	ENST00000423122	ENSE00001601157	132121615	132121817	-0.625388289	0.149777374	0.496408818
kgp4153970	12	66002444	MSRB3 | RPSAP52	ENST00000355869	ENSE00001733461	66002251	66003073	0.741879529	0.150086902	0.496408818
kgp2579933	20	57932766	EDN3 | LOC645605	ENST00000424662	ENSE00001647182	57932364	57932829	0.395527101	0.150115343	0.496408818
rs2952134	8	16989568	EFHA2 | ZDHHC2	ENST00000513892	ENSE00002077855	16988690	16990578	-0.638777537	0.218521769	0.496892713
rs1078819	2	3129088	MYT1L | LOC729897	ENST00000457478	ENSE00001720874	3129067	3129210	-0.768956365	0.219929508	0.496892713
rs9928133	16	83831763	CDH13 | HSBP1	ENST00000567109	ENSE00002630405	83831266	83834245	0.763076004	0.221343118	0.496892713
rs9295914	6	30758707	IER3 | DDR1	ENST00000439406	ENSE00001608692	30758666	30758733	1.463137894	0.147571576	0.49743228
kgp13002976	11	65191044	FRMD8 | NCRNA00084	ENST00000501122	ENSE00001961965	65190269	65213011	-0.854862524	0.150908229	0.497667302
kgp8980484	4	11772074	HS3ST1 | HSP90AB2P	ENST00000515286	ENSE00002073273	11771933	11772099	-0.753811689	0.224112272	0.498380269
kgp11814991	10	5659916	CALML3 | ASB13	ENST00000427341	ENSE00001632682	5659894	5660124	0.788889334	0.225892284	0.498380269
kgp22406932	5	142140072	FGF1 | ARHGAP26	ENST00000432677	ENSE00001747662	142138651	142140563	-0.612263762	0.226536486	0.498380269
kgp27529161	12	132671705	NOC4L | GALNT9	ENST00000538731	ENSE00002290912	132671287	132672246	-0.649353158	0.152617486	0.501932714
rs9721232	8	29654363	C8orf75 | LOC286135	ENST00000523123	ENSE00002091034	29654351	29656006	1.106004681	0.160643961	0.502775231
kgp1852861	14	65689005	MAX | LOC645431	ENST00000531609	ENSE00002179905	65688937	65689065	0.963410359	0.16251047	0.502775231
kgp2872894	7	31454298	LOC100289675 | CCDC129	ENST00000433356	ENSE00001631090	31454259	31454496	1.010090583	0.163285319	0.502775231
kgp13128229	21	20307904	LOC100288117 | C21orf131	ENST00000440372	ENSE00001751294	20307678	20308177	1.10057266	0.164075833	0.502775231
rs5756643	22	37748394	CYTH4 | ELFN2	ENST00000445088	ENSE00001596234	37748230	37748480	0.580321227	0.230642699	0.504053581
rs2642428	1	221005557	MOSC1 | LOC100129376	ENST00000431347	ENSE00001806503	221005455	221005771	0.888658748	0.16625526	0.505840471
kgp4514927	7	26591585	KIAA0087 | LOC285941	ENST00000457000	ENSE00001596777	26591458	26591829	0.916871966	0.15281654	0.506022733
kgp1069026	14	105311439	MGC23270 | KIAA0284	ENST00000556430	ENSE00002477842	105309000	105312253	-0.922226255	0.153557172	0.506022733
kgp9508887	14	77534266	C14orf4 | KIAA1737	ENST00000500215	ENSE00001980448	77533452	77535846	-0.937040887	0.155180305	0.506022733
kgp22067479	5	87566536	TMEM161B | LOC645323	ENST00000513011	ENSE00002074829	87566343	87566950	-0.331543496	0.15466528	0.506602
kgp5518345	2	780358	LOC100128185 | LOC39134	ENST00000415700	ENSE00001761035	780348	780588	-0.675557823	0.154876668	0.506602
rs7722073	5	108661938	FER | PJA2	ENST00000512693	ENSE00002038146	108658697	108662070	-0.843312862	0.155718737	0.507943497
kgp21592724	10	116538837	ABLIM1 | FAM160B1	ENST00000436932	ENSE00001725826	116537710	116539662	-1.145923147	0.156128449	0.507943497
kgp10691965	16	89113353	CBFA2T3 | ACSF3	ENST00000537498	ENSE00002244645	89112580	89114228	0.517506014	0.156881249	0.508195355
kgp15172307	1	234665672	TARBP1 | IRF2BP2	ENST00000435574	ENSE00001674386	234663637	234666088	-0.861932431	0.157047943	0.508195355
rs7573518	2	132166387	LOC389043 | LOC401010	ENST00000437751	ENSE00001792133	132165418	132166622	0.929252329	0.15818992	0.510290064
kgp11332971	11	86425072	ME3 | PRSS23	ENST00000526206	ENSE00002173689	86424732	86425134	-1.004194225	0.169012292	0.510607558
kgp270728	16	56643750	MT2A | MT1L	ENST00000569778	ENSE00002603822	56643413	56643796	0.987960668	0.158335471	0.510991748
kgp7004714	5	175572044	FAM153B | LOC643201	ENST00000515403	ENSE00002084966	175572028	175572198	0.953437923	0.161528143	0.511439066
kgp5059463	2	174746347	LOC643997 | SP3	ENST00000418620	ENSE00001593303	174746253	174746418	0.908384786	0.161955704	0.511439066
kgp14942318	2	780100	LOC100128185 | LOC39134	ENST00000414556	ENSE00001725567	779837	780588	-1.032802775	0.160095062	0.515292638
kgp7666775	7	22930730	SNORD93 | FAM126A	ENST00000421730	ENSE00001671326	22930554	22930768	0.901879733	0.171978976	0.515936929
kgp11752435	16	30759716	SRCAP | PHKG2	ENST00000483578	ENSE00001943631	30759527	30759811	-0.579561249	0.238003683	0.516718523
kgp13952400	17	11465305	FLJ45455 | LOC100289291	ENST00000528745	ENSE00002184577	11464473	11467380	-1.331085548	0.174120548	0.51793146
kgp15702263	1	90421449	LRRC8D | LOC492303	ENST00000445845	ENSE00001696260	90421225	90421760	-1.357879166	0.17825975	0.51793146
kgp926523	4	24672350	DHX15 | SOD3	ENST00000569621	ENSE00002600133	24671185	24673191	-1.139024813	0.178436499	0.51793146
kgp10214297	5	19035626	LOC391769 | CDH18	ENST00000513955	ENSE00002033565	19035306	19036142	0.867439215	0.178591926	0.51793146

kgp3943461	2	38054547	LOC344382 | FAM82A1	ENST00000413792	ENSE00001797876	38053390	38055021	-1.922609851	0.178680317	0.51793146
rs26935	3	64069310	PSMD6 | LOC100287879	ENST00000485805	ENSE00001871475	64068767	64069360	0.87019398	0.180045334	0.518385559
rs4841356	8	10334694	MSRA | LOC346702	ENST00000524047	ENSE00002099410	10334633	10334804	0.3459996	0.161590119	0.518721473
kgp19338198	20	62666038	PRPF6 | PRR17	ENST00000444463	ENSE00001787207	62665697	62667400	0.629641522	0.241254575	0.519515301
kgp6461809	3	30566310	RBMS3 | TGFBR2	ENST00000450746	ENSE00001692853	30566237	30566318	0.612938229	0.243236944	0.519515301
kgp62030	5	4515797	IRX1 | LOC340094	ENST00000503188	ENSE00002026322	4515436	4516889	-0.910653483	0.244014763	0.519515301
kgp11089236	1	179805154	LOC100288340 | TOR1AIP2	ENST00000442108	ENSE00001803508	179805130	179805259	-0.838126151	0.162383086	0.519595492
rs310020	16	81421075	GAN | CMIP	ENST00000568107	ENSE00002626991	81416874	81424489	-1.431094548	0.163467129	0.519595492
kgp3979256	8	9060077	PPP1R3B | TNKS	ENST00000523747	ENSE00002113722	9060009	9060347	-0.548406642	0.163568646	0.519595492
kgp22150258	5	148444507	SH3TC2 | ABLIM3	ENST00000507318	ENSE00002020377	148443630	148446548	0.413715458	0.164750165	0.519595492
kgp12548126	5	147647990	SPINK6 | SPINK5L3	ENST00000501695	ENSE00001960784	147647870	147648567	-0.442762078	0.164761381	0.519595492
kgp4449823	5	117881665	LOC100287135 | DTWD2	ENST00000515704	ENSE00002051714	117881553	117881844	-1.217670793	0.165194231	0.519595492
kgp2211771	11	69867031	LOC100127946 | ANO1	ENST00000528507	ENSE00002193800	69866989	69867165	1.333892848	0.165957097	0.519595492
kgp10808727	16	23683082	DCTN5 | PLK1	ENST00000566996	ENSE00002576769	23681332	23683518	-0.89467598	0.166775364	0.519595492
kgp1570712	8	54428066	OPRK1 | ATP6V1H	ENST00000521930	ENSE00002114547	54427730	54428166	-0.695359425	0.166821448	0.519595492
kgp17581821	3	156800229	LEKR1 | CCNL1	ENST00000471357	ENSE00001879700	156799456	156801064	0.600027575	0.167424995	0.519595492
kgp16497174	16	50674031	NKD1 | SNX20	ENST00000565077	ENSE00002621561	50671504	50674771	0.481827917	0.167624848	0.519595492
kgp28319028	14	85995349	SEL1L | FLRT2	ENST00000380722	ENSE00001486021	85994961	85996332	0.500542248	0.167659065	0.519595492
rs3196549	1	147763232	NBPF11 | LOC100132057	ENST00000434245	ENSE00001734617	147762995	147763407	-0.460064118	0.16777904	0.519595492
rs938906	2	231555684	SP100 | CAB39	ENST00000415174	ENSE00001768231	231555636	231556948	0.597092329	0.168156506	0.519595492
kgp2763924	16	89979959	TCF25 | MC1R	ENST00000539976	ENSE00002202946	89979640	89981576	0.362056992	0.168319666	0.519595492
kgp21417462	19	54368958	NLRP12 | MYADM	ENST00000422045	ENSE00001672208	54368764	54368982	-0.887841273	0.167651331	0.520131289
kgp8613383	12	54539892	LOC400043 | SMUG1	ENST00000564380	ENSE00002606100	54538853	54541009	-0.879829383	0.169880785	0.520131289
kgp19498751	14	39644282	TRAPPC6B | PNN	ENST00000556537	ENSE00002477748	39644089	39644516	0.985258106	0.171019782	0.520131289
kgp16264332	16	1029110	LMF1 | LOC100287129	ENST00000565069	ENSE00002606805	1029025	1029110	-1.069967238	0.175631562	0.520131289
kgp22742691		5571581	LOC729162 | NLGN4X	ENST00000456559	ENSE00001596540	5571464	5571744	1.531735692	0.176436838	0.520131289
kgp11086996	11	56644575	OR9G4 | OR5AK2	ENST00000532274	ENSE00002149580	56643073	56644687	0.907483824	0.177363253	0.520131289
rs726330	12	40536061	SLC2A13 | LRRK2	ENST00000563933	ENSE00002589573	40534728	40536678	0.917551314	0.178127398	0.520131289
kgp6316455	8	12623463	LONRF1 | LOC340357	ENST00000534827	ENSE00002185079	12623358	12624104	0.851915422	0.179629032	0.520131289
rs1041914	6	106060602	PREP | PRDM1	ENST00000423489	ENSE00001617276	106060542	106060667	1.043951673	0.18111643	0.520131289
kgp13151543	21	46410367	C21orf70 | NCRNA00162	ENST00000439088	ENSE00001802548	46409779	46411747	0.973680154	0.182045951	0.520131289
kgp2512842	20	17868104	BANF2 | SNX5	ENST00000425405	ENSE00001740096	17868007	17868448	0.456209997	0.169634717	0.520868616
kgp3969847	15	90788908	C15orf58 | TTLL13	ENST00000565730	ENSE00002596043	90788645	90789043	-0.571104216	0.170583386	0.520868616
kgp22578963	5	87705997	TMEM161B | LOC645323	ENST00000510087	ENSE00001963557	87705890	87706011	-0.819879015	0.170603797	0.520868616
kgp6984142	3	134068071	RYK | AMOTL2	ENST00000568384	ENSE00002597828	134066130	134068075	-0.677799802	0.171196073	0.520868616
kgp12522057	12	132671796	NOC4L | GALNT9	ENST00000538731	ENSE00002290912	132671287	132672246	-0.454585329	0.171669714	0.520868616
kgp6472267	6	169687446	THBS2 | LOC100289536	ENST00000457833	ENSE00001785351	169687140	169687571	0.346946004	0.172391736	0.520868616
kgp4607813	2	9898712	YWHAQ | TAF1B	ENST00000474667	ENSE00001869213	9897625	9899428	-0.433647317	0.172794954	0.520868616
rs11686263	2	89110855	FLJ40330 | LOC100132330	ENST00000452230	ENSE00001624389	89109984	89111209	-0.336295029	0.172817228	0.520868616
kgp17293870	6	141770017	CITED2 | LOC729076	ENST00000565399	ENSE00002622426	141768148	141772143	0.772046517	0.173313498	0.520868616
kgp21996609	10	5652373	CALML3 | ASB13	ENST00000425246	ENSE00001777052	5652277	5652399	0.62426795	0.173771158	0.520868616
kgp21868483	10	4285245	LOC727894 | LOC10012835	ENST00000418372	ENSE00001533958	4285142	4285301	0.704655077	0.174117299	0.520868616
kgp8852246	14	62148116	PRKCH | HIF1A	ENST00000557544	ENSE00002446428	62147759	62148293	0.650007257	0.17497342	0.520868616
rs7255	2	20878820	GDF7 | C2orf43	ENST00000565841	ENSE00002602242	20877569	20879005	0.353977339	0.175793096	0.520868616
rs2251244	14	62023264	PRKCH | HIF1A	ENST00000553288	ENSE00002467335	62023031	62023270	0.511742139	0.17608018	0.520868616
kgp7060630	1	94784068	ARHGAP29 | ABCD3	ENST00000414039	ENSE00001703043	94784035	94784463	0.758783575	0.176621703	0.520868616
kgp19032220	12	4809776	NDUFA9 | GALNT8	ENST00000527518	ENSE00002151206	4809583	4810345	0.689106541	0.176631445	0.520868616
kgp1812712	6	134824022	SGK1 | LOC645175	ENST00000456749	ENSE00001676049	134823926	134825157	0.445634337	0.177105217	0.520868616
kgp29783257	10	47099688	PPYR1 | LOC728643	ENST00000422732	ENSE00001763272	47099588	47099716	0.395731391	0.177122074	0.520868616
kgp1933956	1	22350834	CELA3A | HSPC157	ENST00000455966	ENSE00001645056	22350487	22350938	0.324906479	0.177232397	0.520868616
rs6981122	8	128094460	FAM84B | POU5F1B	ENST00000523510	ENSE00002124052	128094366	128094466	-0.441846356	0.177362884	0.520868616
kgp5473634	16	81421977	GAN | CMIP	ENST00000568107	ENSE00002626991	81416874	81424489	-1.128699316	0.178708544	0.521116947
kgp17099527	6	81178521	BCKDHB | FAM46A	ENST00000569267	ENSE00002590559	81176675	81178797	-0.508210965	0.179064699	0.521116947
kgp10062178	15	25280802	PAR5 | SNORD109A	ENST00000552334	ENSE00002349489	25277020	25281637	0.31978719	0.179156813	0.521116947
kgp5352748	14	39644171	TRAPPC6B | PNN	ENST00000556537	ENSE00002477748	39644089	39644516	1.111973214	0.179852079	0.521116947
rs1001304	1	212732686	LOC100287039 | ATF3	ENST00000564287	ENSE00002618928	212731175	212733073	-0.68057587	0.180301743	0.521116947
kgp7606402	4	8358279	HTRA3 | ACOX3	ENST00000505448	ENSE00002069127	8357038	8359103	-0.39718754	0.180428775	0.521116947
kgp14344508	2	9897684	YWHAQ | TAF1B	ENST00000474667	ENSE00001869213	9897625	9899428	-0.54754537	0.180469664	0.521116947
kgp11979014	2	8762912	LOC339788 | ID2	ENST00000425678	ENSE00001638956	8762900	8763072	1.392964811	0.18283261	0.521449541
kgp24285002	2	38054707	LOC344382 | FAM82A1	ENST00000413792	ENSE00001797876	38053390	38055021	-1.891065265	0.18436649	0.521449541
kgp1025566	4	62293253	IGFBP7 | LPHN3	ENST00000381217	ENSE00001385838	62292442	62293939	1.222288157	0.184756015	0.521449541
kgp16350329	16	1026124	LMF1 | LOC100287129	ENST00000568394	ENSE00002595585	1025761	1026400	-1.100712237	0.181266833	0.522169611
kgp19902018	15	81285046	MESDC2 | MESDC1	ENST00000563737	ENSE00002624578	81283145	81285599	-0.557472303	0.246903856	0.522296619
kgp6348834	10	116539304	ABLIM1 | FAM160B1	ENST00000436932	ENSE00001725826	116537710	116539662	-0.760948864	0.248493785	0.522311778
rs17123266	14	51428551	PYGL | TRIM9	ENST00000553648	ENSE00002524429	51428364	51428720	-0.893202231	0.188859071	0.523957752
kgp12005440	2	1625705	TPO | PXDN	ENST00000366424	ENSE00001647238	1624282	1625751	0.864394121	0.190482682	0.523957752
kgp15956366	18	4295267	LOC284215 | LOC642597	ENST00000565811	ENSE00002615665	4293160	4295405	-0.948907565	0.191551873	0.523957752
rs1408954	1	59486190	LOC100131060 | LOC72946	ENST00000447329	ENSE00001702204	59486059	59486295	0.876637279	0.191847021	0.523957752
kgp5975933	9	138477032	PAEP | GLT6D1	ENST00000447907	ENSE00001767898	138476895	138477210	0.832443914	0.191880547	0.523957752
kgp24251078	2	75156405	HK2 | POLE4	ENST00000377469	ENSE00001474023	75155366	75158663	1.031698157	0.193271616	0.523957752
kgp24398992	2	59241519	LOC644456 | LOC730134	ENST00000452840	ENSE00001659117	59241465	59241533	0.98291761	0.194741636	0.523957752
rs2972050	2	28671048	FOSL2 | PLB1	ENST00000439700	ENSE00001803576	28671034	28671737	0.835668276	0.195415479	0.523957752
kgp10506064	2	217475599	RPL37A | IGFBP2	ENST00000441803	ENSE00001659572	217475467	217475925	0.2923851	0.18294199	0.524948499
kgp11177536	20	17868221	BANF2 | SNX5	ENST00000425405	ENSE00001740096	17868007	17868448	0.413990189	0.183414829	0.524948499
kgp16402183	16	86754592	FOXL1 | LOC100288525	ENST00000563331	ENSE00002586134	86754294	86754609	-1.161069025	0.183828291	0.524948499
kgp18885027	12	66002270	MSRB3 | RPSAP52	ENST00000355869	ENSE00001733641	66002251	66003073	0.805102472	0.184335557	0.524948499
kgp11705341	8	54428977	OPRK1 | ATP6V1H	ENST00000426023	ENSE00002169965	54427731	54429514	-0.589954628	0.184523236	0.524948499
kgp1686179	18	77807137	C18orf22 | ADNP2	ENST00000562391	ENSE00002576217	77806900	77807425	-0.618682773	0.184841021	0.524948499
kgp3389725	15	101709952	LRRK1 | CHSY1	ENST00000558515	ENSE00002553658	101709774	101710146	0.894902906	0.198882366	0.526157431
kgp603614	16	89119156	CBFA2T3 | ACSF3	ENST00000537498	ENSE00002221112	89118869	89119373	-0.803234033	0.20121616	0.526157431
kgp11628107	13	58807131	PCDH17 | LOC341689	ENST00000454780	ENSE00001701736	58806969	58807251	0.863067622	0.202407745	0.526157431
kgp10504114	12	115151623	TBX3 | MED13L	ENST00000551875	ENSE00002365231	115151616	115151804	0.867990176	0.203080457	0.526157431
rs12929407	16	63092995	CDH8 | CDH11	ENST00000568741	ENSE00002616828	63091122	63093812	-1.104077277	0.203990928	0.526157431
kgp1860715	2	42117501	SLC8A1 | LOC400950	ENST00000398796	ENSE00001677131	42117491	42117558	0.796017749	0.204183214	0.526157431
kgp14962002	2	75156451	HK2 | POLE4	ENST00000377469	ENSE00001474023	75155366	75158663	0.797109535	0.204821191	0.526157431
rs5915590		5587613	LOC729162 | NLGN4X	ENST00000444185	ENSE00001609078	5587447	5587647	1.232897548	0.186816961	0.528727248

kgp14464684	2	118597277	LOC100287740 | CCDC93	ENST00000420330	ENSE00001639718	118596758	118599234	-0.413763233	0.186956046	0.529708798
kgp4199777	13	30679604	UBL3 | KATNAL1	ENST00000413591	ENSE00001796013	30677315	30679832	-0.879076821	0.18908979	0.53015829
kgp5869405	2	75159388	HK2 | POLE4	ENST00000418204	ENSE00001757721	75159326	75159416	0.726324099	0.253856889	0.530207426
rs7732591	5	142125177	FGF1 | ARHGAP26	ENST00000432677	ENSE00001803349	142125165	142125283	-1.04726159	0.19214497	0.531513916
kgp2633573	21	29818763	C21orf94 | NCRNA00161	ENST00000433310	ENSE00001711357	29818611	29818793	0.859833579	0.193116723	0.531513916
kgp5453080	16	1026398	LMF1 | LOC100287129	ENST00000568394	ENSE00002595585	1025761	1026400	-1.024115019	0.188071691	0.531621852
kgp17139560	6	141770335	CITED2 | LOC729076	ENST00000565399	ENSE00002622426	141768148	141772143	1.093460795	0.256157775	0.531648212
kgp5718408	15	40664647	DISP2 | C15orf23	ENST00000561261	ENSE00002575109	40664575	40664677	-0.445984014	0.189952534	0.532671817
kgp9580216	6	42060827	TAF8 | C6orf132	ENST00000562471	ENSE00002625332	42059976	42061997	1.136752006	0.189984567	0.532671817
kgp6544381	6	141769692	CITED2 | LOC729076	ENST00000565399	ENSE00002622426	141768148	141772143	0.661394795	0.190160499	0.532671817
kgp953292	1	98433839	DPYD | FLJ35409	ENST00000561881	ENSE00002583625	98432561	98434370	0.465886188	0.190483005	0.532671817
kgp7182756	20	29638332	FRG1B | DEFB115	ENST00000446917	ENSE00001782794	29638246	29638813	-0.307867675	0.191012532	0.532671817
kgp5157122	17	70030172	LOC124685 | SOX9	ENST00000543512	ENSE00002217013	70030040	70030233	-0.973548253	0.191091049	0.532671817
kgp3444717	8	128085044	FAM84B | POU5F1B	ENST00000523510	ENSE00002108688	128084939	128085163	-0.422757667	0.192049366	0.534109642
kgp6552231	9	22768155	DMRTA1 | LOC402360	ENST00000448570	ENSE00001671272	22767961	22768315	-0.387840332	0.192572072	0.534332164
kgp7657204	16	71465028	CALB2 | ZNF23	ENST00000561754	ENSE00002586894	71465024	71465177	1.025018921	0.19652066	0.534827081
kgp19566013	14	39644195	TRAPPC6B | PNN	ENST00000556537	ENSE00002477748	39644089	39644516	0.88796723	0.19788602	0.534827081
kgp535773	14	46181143	LOC644589 | LOC10028958	ENST00000555442	ENSE00002481274	46181102	46181156	-1.228197033	0.209834253	0.535826752
kgp19747032	15	40604999	PLCB2 | C15orf52	ENST00000559030	ENSE00002547681	40604816	40608835	-1.265642825	0.193787127	0.536470328
kgp22114822	5	148443888	SH3TC2 | ABLIM3	ENST00000515519	ENSE00002061149	148443813	148443921	0.308732899	0.194656076	0.53669596
kgp22760639	16	1026009	LMF1 | LOC100287129	ENST00000568394	ENSE00002595585	1025761	1026400	-0.907163993	0.195110932	0.53669596
rs17013477	4	129489592	PGRMC2 | PHF17	ENST00000514265	ENSE00002048854	129489127	129490442	0.508748132	0.19520259	0.53669596
rs2282351	10	45676748	LOC100133308 | OR13A1	ENST00000422807	ENSE00001735051	45676530	45676875	0.667147753	0.2612622	0.538853287
kgp7441346	16	81112136	C16orf46 | GCSH	ENST00000501068	ENSE00001976225	81111937	81112466	-0.497905245	0.26387811	0.540868176
rs2312968	13	20676866	ZMYM2 | GJA3	ENST00000455848	ENSE00001620926	20676840	20676965	0.910083238	0.202542461	0.542524449
kgp17918866	3	156800088	LEKR1 | CCNL1	ENST00000471357	ENSE00001879700	156799456	156801064	0.581193015	0.197886418	0.542838423
rs3763243	6	82555289	FAM46A | IBTK	ENST00000418567	ENSE00001802829	82554902	82555626	-0.454012635	0.200171365	0.547861309
kgp28568935	8	102087704	YWHAZ | ZNF706	ENST00000514926	ENSE00002063679	102087397	102088479	0.569500483	0.201380801	0.549535027
kgp7986207	6	141769220	CITED2 | LOC729076	ENST00000565399	ENSE00002622426	141768148	141772143	0.505448782	0.201775918	0.549535027
kgp6947923	14	75763091	FOS | JDP2	ENST00000558575	ENSE00002540211	75762823	75763341	-0.527163545	0.202612998	0.549535027
GA030572	15	67811407	IQCH | C15orf61	ENST00000561232	ENSE00002566141	67811338	67811440	0.360504504	0.203053968	0.549535027
kgp18408469	9	37087641	LOC100287249 | ZCCHC7	ENST00000429493	ENSE00001770690	37086665	37090398	-0.330224614	0.203418318	0.549535027
kgp18619988	9	37088682	LOC100287249 | ZCCHC7	ENST00000429493	ENSE00001770690	37086665	37090398	-1.506246878	0.203514629	0.549535027
rs10408331	19	28221474	LOC100101266 | LOC14818	ENST00000561521	ENSE00002619204	28221181	28223355	1.537400179	0.204607543	0.549861305
rs10504154	8	54428996	OPRK1 | ATP6V1H	ENST00000426023	ENSE00002169965	54427731	54429514	-0.517516486	0.204810861	0.549861305
rs1422577	5	148486371	SH3TC2 | ABLIM3	ENST00000507373	ENSE00002019457	148486331	148486701	-0.49505346	0.205247937	0.549861305
kgp10594557	2	114588534	SLC35F5 | ACTR3	ENST00000435407	ENSE00001660656	114588429	114588696	-0.391121422	0.205544361	0.549861305
kgp19489012	14	38781060	CLEC14A | LOC283547	ENST00000555636	ENSE00002455148	38780991	38781134	-0.810382916	0.206771698	0.549861305
kgp1244276	6	109089783	FOXO3 | ARMC2	ENST00000448744	ENSE00001698309	109089594	109090773	-0.509673391	0.206940322	0.549861305
kgp27344987	9	137476689	RXRA | COL5A1	ENST00000423455	ENSE00001806378	137475038	137477036	-0.653289606	0.207468423	0.549861305
kgp19918532	15	72766057	LOC100130579 | ARIH1	ENST00000565181	ENSE00002621761	72765185	72767509	0.273062301	0.207625758	0.549861305
kgp11054009	7	6695998	ZNF316 | LOC100133111	ENST00000564837	ENSE00002606444	6694495	6696063	-0.34945129	0.20823247	0.549861305
kgp4354372	22	42671066	LOC388906 | NFAM1	ENST00000415205	ENSE00001699487	42670994	42671202	-1.028870121	0.20841349	0.549861305
kgp19626814	14	85995662	SEL1L | FLRT2	ENST00000380722	ENSE00001486021	85994961	85996332	0.313910532	0.208646626	0.549861305
kgp28527830	15	77337454	PSTPIP1 | TSPAN3	ENST00000560446	ENSE00002562292	77337397	77337502	-0.479891607	0.21022059	0.552194788
kgp7216336	17	80251977	CSNK1D | CD7	ENST00000566986	ENSE00002591651	80251592	80252786	-0.454622041	0.210447061	0.552194788
kgp3445447	8	16990078	EFHA2 | ZDHHC2	ENST00000513892	ENSE00002077855	16988690	16990578	-0.839334907	0.21820257	0.553898833
rs3803522	15	68126675	LBXCOR1 | RNU6-1	ENST00000502156	ENSE00001979265	68126648	68128119	-0.634737715	0.211561293	0.553914274
rs13025811	2	216582873	FN1 | LOC646324	ENST00000415479	ENSE00001677355	216582766	216582985	0.76612169	0.220169152	0.55560333
rs3761180	20	48894872	CEBPB | PTPN1	ENST00000445003	ENSE00001632288	48894714	48894964	-0.391561521	0.21271707	0.555734857
rs4767119	12	114183479	LHX5 | RBM19	ENST00000547963	ENSE00002355822	114182382	114184400	-0.550221694	0.274283669	0.558725992
rs10108910	8	8419612	PRAGMIN | CLDN23	ENST00000524073	ENSE00002092903	8419343	8419634	0.75454175	0.223088787	0.559678888
kgp28263734	14	97411417	VRK1 | C14orf64	ENST00000495064	ENSE00001942533	97411386	97411487	0.428844418	0.215055492	0.559770362
kgp5594649	4	47842372	CORIN | NFXL1	ENST00000563286	ENSE00002580194	47842139	47846356	0.326307274	0.215634917	0.559770362
rs16970331	16	70611562	SF3B3 | IL34	ENST00000562874	ENSE00002580772	70611221	70611571	-0.37334695	0.216136476	0.559770362
rs9262199	6	30759283	IER3 | DDR1	ENST00000439406	ENSE00001648072	30759203	30760027	0.993363321	0.216341833	0.559770362
rs754171	1	17522821	LOC400743 | PADI1	ENST00000539219	ENSE00002261175	17520556	17524112	-0.761321464	0.216744285	0.559770362
kgp1598537	16	80632143	DYNLRB2 | CDYL2	ENST00000570137	ENSE00002617613	80631803	80636416	0.542988898	0.217393514	0.559770362
rs907652	5	9549178	SNORD123 | TAS2R1	ENST00000509788	ENSE00002040542	9549102	9549702	-0.623303026	0.217699929	0.559770362
kgp13086588	21	36511336	RUNX1 | SETD4	ENST00000455028	ENSE00001764112	36511034	36511519	0.298655233	0.21824191	0.559770362
rs1007832	2	8684493	LOC339788 | ID2	ENST00000418358	ENSE00001592879	8683722	8684532	0.289345218	0.218435659	0.559770362
kgp11533093	6	168082209	LOC401286 | LOC441178	ENST00000400831	ENSE00001612490	168080306	168082617	0.807700217	0.214836052	0.561553534
kgp10132499	9	134696326	RAPGEF1 | MED27	ENST00000444708	ENSE00001741256	134696029	134696375	0.822355497	0.21484799	0.561553534
kgp30926385		2533309	DHRSX | CD99	ENST00000445785	ENSE00001676508	2532758	2533388	-0.834948411	0.215262188	0.561553534
kgp9189309	5	142083969	FGF1 | ARHGAP26	ENST00000566527	ENSE00002614243	142083347	142084986	-0.897698643	0.22032052	0.564588299
kgp8197522	22	39320602	CBX6 | APOBEC3A	ENST00000450216	ENSE00001738334	39320122	39320713	-0.883061894	0.223350509	0.564588299
rs7524120	1	92865160	RPAP2 | GFI1	ENST00000564442	ENSE00002592577	92864531	92867613	-0.832697881	0.223715891	0.564588299
rs10177020	2	38054589	LOC344382 | FAM82A1	ENST00000413792	ENSE00001797876	38053390	38055021	-1.0868101	0.223953359	0.564588299
kgp22797838	5	106154155	RAB9P1 | LOC100287833	ENST00000513273	ENSE00002042089	106154100	106154178	-0.8265929	0.223197672	0.570761844
kgp7525168	7	153110723	LOC100288889 | LOC10013	ENST00000453187	ENSE00001642767	153110698	153111021	-0.470761303	0.285197643	0.575486891
kgp4273849	2	9789403	YWHAQ | TAF1B	ENST00000478468	ENSE00001888943	9788981	9789568	-0.571901459	0.285999546	0.575486891
kgp28094661	14	101376589	MEG8 | SNORD113-1	ENST00000556475	ENSE00002479305	101376570	101376641	0.789362084	0.22553824	0.575527812
kgp11199017	16	19356094	LOC728276 | TMC5	ENST00000565398	ENSE00002596933	19355692	19356145	0.46760494	0.288284726	0.576569452
kgp12005264	3	64068857	PSMD6 | LOC100287879	ENST00000485805	ENSE00001871475	64068767	64069360	0.812148606	0.231355483	0.578388707
kgp8631773	3	177040528	TBL1XR1 | KCNMB2	ENST00000425388	ENSE00001591376	177039217	177041206	0.756300929	0.227215677	0.578585069
kgp17442075	6	81153171	BCKDHB | FAM46A	ENST00000452402	ENSE00001672190	81153061	81153255	-0.33021039	0.22916125	0.57894758
kgp11118502	4	1576105	NKX1-1 | LOC100289589	ENST00000466692	ENSE00001822323	1575789	1576271	-0.814188542	0.229403786	0.578947158
kgp18608891	9	37086686	LOC100287249 | ZCCHC7	ENST00000430809	ENSE00001609838	37086665	37086871	-0.292521729	0.229737636	0.578947158
kgp11188446	2	10180004	UNQ5830 | KLF11	ENST00000567540	ENSE00002582289	10179219	10180790	-0.30270883	0.231010129	0.578947158
kgp27647687	12	1612422	ERC1 | FBXL14	ENST00000515614	ENSE00002028096	1611627	1612636	0.862244498	0.231071842	0.578947158
kgp1646718	16	56643594	MT2A | MT1L	ENST00000569778	ENSE00002603822	56643413	56643796	0.845172983	0.2312066	0.578947158
kgp2953720	14	50413230	ARF6 | C14orf182	ENST00000556130	ENSE00002433545	50412872	50413432	-0.344931678	0.231614673	0.578947158
rs8033336	15	43035131	CDAN1 | TTBK2	ENST00000500850	ENSE00002588053	43034200	43035400	-0.463773856	0.231669268	0.578947158
kgp6196089	1	234664944	TARBP1 | IRF2BP2	ENST00000435574	ENSE00001674386	234663637	234666088	-0.600277409	0.231674795	0.578947158
rs12427929	13	48504709	HTR2A | SUCLA2	ENST00000566385	ENSE00002595049	48504290	48506757	0.349376447	0.232155746	0.578950384
kgp11805279	6	109089170	FOXO3 | ARMC2	ENST00000416104	ENSE00001702473	109088199	109089659	-0.752519733	0.233909132	0.579939997

rs353212	10	6779443	LOC439949 | SFMBT2	ENST00000417112	ENSE00001657227	6779344	6779590	0.52176381	0.233523476	0.580161066
kgp6249333	12	65369745	TBC1D30 | WIF1	ENST00000535058	ENSE00002259827	65368351	65371302	0.308904951	0.23360255	0.580161066
rs7699689	4	127452	LOC100287931 | ZNF718	ENST00000513304	ENSE00002046767	126732	127532	-0.393449611	0.234236765	0.58017238
GA002720	2	217475515	RPL37A | IGFBP2	ENST00000441803	ENSE00001659572	217475467	217475925	0.292298363	0.234568452	0.58017238
kgp18085194	3	134067011	RYK | AMOTL2	ENST00000568384	ENSE00002597828	134066130	134068075	-0.36416081	0.235494157	0.581270854
kgp27738117	12	1612576	ERC1 | FBXL14	ENST00000515614	ENSE00002028096	1611627	1612636	0.82269651	0.237959971	0.582551969
rs16869156	6	71109059	COL9A1 | FAM135A	ENST00000418403	ENSE00001643230	71108918	71109120	0.425911702	0.237995626	0.582551969
rs512715	11	65191208	FRMD8 | NCRNA00084	ENST00000501122	ENSE00001961965	65190269	65213011	-0.419079404	0.239760298	0.582551969
kgp12700902	11	65191950	FRMD8 | NCRNA00084	ENST00000501122	ENSE00001961965	65190269	65213011	0.271586478	0.240017767	0.582551969
kgp11845895	1	17523426	LOC400743 | PADI1	ENST00000539219	ENSE00002261175	17520556	17524112	-1.079046197	0.240365039	0.582551969
rs6892417	5	1939938	IRX4 | IRX2	ENST00000513419	ENSE00002070223	1939886	1940055	-1.398621029	0.240366539	0.582551969
kgp25049541	1	234769769	IRF2BP2 | PP2672	ENST00000429269	ENSE00001771955	234768694	234770526	0.55262563	0.240548794	0.582551969
kgp10457959	8	130229151	PVT1 | LOC100287906	ENST00000509893	ENSE00002036854	130228728	130229652	0.300332223	0.240587459	0.582551969
kgp12466281	3	177613524	TBL1XR1 | KCNMB2	ENST00000436078	ENSE00001753247	177613401	177613564	-0.840423997	0.241904744	0.582551969
kgp26971342	3	125605535	LOC651856 | LOC10012555	ENST00000468859	ENSE00001925582	125604723	125606178	-0.550027682	0.241909481	0.582551969
rs7930061	11	107184123	GUCY1A2 | CWF19L2	ENST00000561746	ENSE00002621788	107182858	107186997	-0.860378724	0.242309235	0.582551969
rs7741924	6	158733310	GTF2H5 | TULP4	ENST00000432358	ENSE00001664047	158733083	158733390	-0.313162803	0.24246352	0.582551969
kgp2030580	4	47845299	CORIN | NFXL1	ENST00000563286	ENSE00002580194	47842139	47846356	-0.625814711	0.243380904	0.582551969
kgp19962036	15	95025175	MCTP2 | LOC440311	ENST00000565106	ENSE00002296294	95024040	95027181	-0.25505864	0.243747443	0.582551969
kgp6564877	16	89113712	CBFA2T3 | ACSF3	ENST00000537498	ENSE00002244645	89112580	89114228	0.599427821	0.244216136	0.582551969
kgp1355833	15	67813210	IQCH | C15orf61	ENST00000559285	ENSE00002550018	67813117	67813287	0.327738755	0.244216956	0.582551969
kgp7830411	12	1612579	ERC1 | FBXL14	ENST00000515614	ENSE00002028096	1611627	1612636	0.851824445	0.244441409	0.582551969
kgp5078811	8	54429080	OPRK1 | ATP6V1H	ENST00000426023	ENSE00002169965	54427731	54429514	-0.301023856	0.244771775	0.582551969
kgp19790636	15	72766458	LOC100130579 | ARIH1	ENST00000565181	ENSE00002621761	72765185	72767509	-0.400481338	0.24518343	0.582551969
rs11820310	11	68384108	SAPS3 | GAL	ENST00000564469	ENSE00002582835	68383943	68384179	-0.76024994	0.241457615	0.583081878
kgp18783201	12	124067102	RILPL1 | TMED2	ENST00000498967	ENSE00002259901	124066767	124067714	-0.842094766	0.241591175	0.583081878
kgp13085974	21	46410660	C21orf70 | NCRNA00162	ENST00000439088	ENSE00001802548	46409779	46411747	0.899087817	0.241877025	0.583081878
kgp19693086	14	77536948	C14orf4 | KIAA1737	ENST00000557526	ENSE00002498841	77535523	77537189	-0.746633855	0.242950783	0.583081878
kgp8319129	16	21806168	OTOA | LOC730092	ENST00000567370	ENSE00002577410	21805416	21807080	0.380586597	0.246265469	0.583227501
rs279025	1	16788515	NECAP2 | CROCCL2	ENST00000457898	ENSE00001761634	16787443	16789782	-0.309593132	0.246434155	0.583227501
rs2288341	15	44819581	CTDSPL2 | LOC645212	ENST00000560750	ENSE00002572553	44819455	44819737	-0.315211405	0.246923568	0.583242165
kgp4853661	16	2014979	RPS2 | SNHG9	ENST00000531523	ENSE00002165414	2014960	2015130	0.551319997	0.29374808	0.583957027
rs2382948	16	1025983	LMF1 | LOC100287129	ENST00000568394	ENSE00002595585	1025761	1026400	-1.085274835	0.248137913	0.584965744
kgp10894596	12	93397419	EEA1 | LOC100127919	ENST00000549914	ENSE00002381227	93397191	93397749	0.738089126	0.250383253	0.585041161
kgp5657678	21	46410609	C21orf70 | NCRNA00162	ENST00000439088	ENSE00001802548	46409779	46411747	0.833241004	0.25111357	0.585041161
kgp272231	12	104565703	NFYB | TXNRD1	ENST00000547554	ENSE00002363303	104565551	104565814	-0.732972166	0.251161358	0.585041161
rs17762734	2	231561178	SP100 | CAB39	ENST00000415174	ENSE00001612168	231561018	231561410	0.929027553	0.253373631	0.585041161
rs13213674	6	3907174	LOC100289591 | PRPF4B	ENST00000566733	ENSE00002617674	3905144	3912213	0.788455793	0.254322837	0.585041161
kgp3839885	14	50409121	ARF6 | C14orf182	ENST00000556913	ENSE00002499815	50408654	50410610	-0.821496752	0.255931485	0.585041161
kgp22812512		21916054	MBTPS2 | SMS	ENST00000449605	ENSE00001619106	21913079	21920026	-0.789192629	0.257418111	0.585041161
kgp16228142	16	79804376	MAF | DYNLRB2	ENST00000568751	ENSE00002607451	79804368	79804424	-0.353911268	0.250013937	0.586454077
kgp742881	6	109090349	FOXO3 | ARMC2	ENST00000448744	ENSE00001698309	109089594	109090773	-0.352883852	0.250798844	0.586454077
rs2236536	1	182058661	ZNF648 | LOC100130996	ENST00000428646	ENSE00001688250	182058500	182059247	0.375633367	0.250885754	0.586454077
kgp19420106	14	85995199	SEL1L | FLRT2	ENST00000380722	ENSE00001486021	85994961	85996332	0.266307863	0.251698742	0.586454077
kgp1638031	16	81423102	GAN | CMIP	ENST00000568107	ENSE00002626991	81416874	81424489	-1.132489736	0.252701662	0.586454077
kgp9315747	9	81751480	PSAT1 | TLE4	ENST00000566873	ENSE00002621179	81750337	81752092	0.28755959	0.252709556	0.586454077
kgp2484178	11	58903016	FAM111B | FAM111A	ENST00000531708	ENSE00002199481	58902763	58903061	-0.296605589	0.252767218	0.586454077
kgp3704817	2	52949860	LOC730100 | ASB3	ENST00000443237	ENSE00001746572	52949815	52950001	0.469909753	0.253532228	0.586454077
kgp19637853	14	70936332	ADAM21 | ADAM20	ENST00000556964	ENSE00002518821	70935598	70938309	0.43945775	0.253592288	0.586454077
kgp24690963	22	26920942	TFIP11 | TPST2	ENST00000564772	ENSE00002607234	26917949	26921002	-0.629825895	0.253903516	0.586454077
rs9262222	6	30807735	IER3 | DDR1	ENST00000442852	ENSE00001790053	30807303	30807796	-0.585758412	0.254335231	0.586454077
kgp2278872	9	37087064	LOC100287249 | ZCCHC7	ENST00000429493	ENSE00001770690	37086665	37090398	-0.643232769	0.256063805	0.586454077
kgp3978081	8	130692301	LOC100287906 | GSDMC	ENST00000446592	ENSE00002063024	130692174	130692485	-0.510856432	0.256359173	0.586454077
kgp9154027	16	32469035	LOC390705 | TP53TG3	ENST00000565549	ENSE00002609095	32465933	32470079	-0.354395061	0.257199423	0.586454077
kgp18570472	9	37089135	LOC100287249 | ZCCHC7	ENST00000429493	ENSE00001770690	37086665	37090398	-1.330026718	0.257276192	0.586454077
kgp12715221	11	74954285	LOC441617 | ARRB1	ENST00000562197	ENSE00002609763	74953064	74954742	0.482589472	0.258069595	0.586454077
kgp4281417	11	65191447	FRMD8 | NCRNA00084	ENST00000501122	ENSE00001961965	65190269	65213011	-1.168343254	0.258385485	0.586454077
kgp219544	6	3910607	LOC100289591 | PRPF4B	ENST00000566733	ENSE00002617674	3905144	3912213	0.341830341	0.258682773	0.586454077
kgp8080973	4	47842346	CORIN | NFXL1	ENST00000563286	ENSE00002580194	47842139	47846356	0.246991182	0.259171615	0.586454077
kgp1054326	15	44827257	CTDSPL2 | LOC645212	ENST00000313807	ENSE00001427514	44825691	44827672	-1.074408829	0.259218208	0.586454077
GA002157	2	75169401	HK2 | POLE4	ENST00000453951	ENSE00001643565	75169014	75169797	0.357123992	0.25970131	0.586454077
kgp11604000	3	114034574	TIGIT | ZBTB20	ENST00000570269	ENSE00002576360	114033348	114035026	-0.317037575	0.259750179	0.586454077
kgp527360	5	127338508	LOC728586 | FLJ33630	ENST00000512185	ENSE00002085536	127338387	127338514	-0.274003134	0.260103939	0.586454077
kgp25277067	1	234665844	TARBP1 | IRF2BP2	ENST00000435574	ENSE00001674386	234663637	234666088	-0.52833117	0.26059897	0.586454077
kgp11679236	2	231556040	SP100 | CAB39	ENST00000415174	ENSE00001768231	231555636	231556948	0.514523391	0.260916188	0.586454077
rs2915806	5	148450915	SH3TC2 | ABLIM3	ENST00000515519	ENSE00002081992	148450910	148451090	0.246174558	0.261450431	0.586562583
kgp7499122	18	10413506	VAPA | APCDD1	ENST00000567609	ENSE00002594117	10412778	10414367	0.849306014	0.260158185	0.586822974
kgp6240162	11	74954342	LOC441617 | ARRB1	ENST00000562197	ENSE00002609763	74953064	74954742	0.723818019	0.262972154	0.588260828
kgp11073252	21	44786015	FLJ41733 | SIK1	ENST00000435702	ENSE00001619112	44785987	44786446	0.894200682	0.265205438	0.588260828
kgp14828936	2	780391	LOC100128185 | LOC39134	ENST00000415700	ENSE00001761035	780348	780588	-0.728055773	0.266678242	0.588260828
kgp6371819	16	23681432	DCTN5 | PLK1	ENST00000566996	ENSE00002576769	23681332	23683518	-0.536957141	0.298708753	0.589219389
kgp3311005	1	87820741	LMO4 | PKN2	ENST00000452509	ENSE00001793610	87820527	87820881	-0.564321473	0.299966234	0.589219389
rs40191	16	66517326	BEAN | FLJ27243	ENST00000544589	ENSE00002208571	66516775	66519747	-0.479517415	0.263507141	0.590079998
rs11855930	15	90788884	C15orf58 | TTLL13	ENST00000565730	ENSE00002596043	90788645	90789043	-0.483921075	0.264340551	0.590242488
kgp5462768	3	188665108	LPP | LOC100132319	ENST00000444488	ENSE00001791567	188665068	188665428	0.379061619	0.264557735	0.590242488
kgp20419204	8	102087605	YWHAZ | ZNF706	ENST00000514926	ENSE00002063679	102087397	102088479	0.672249606	0.305094468	0.590438026
kgp17958347	3	177040947	TBL1XR1 | KCNMB2	ENST00000425388	ENSE00001591376	177039217	177041206	0.595058375	0.309503437	0.590438026
kgp6826295	20	17868447	BANF2 | SNX5	ENST00000425405	ENSE00001740096	17868007	17868448	0.476800492	0.310662983	0.590438026
kgp4995234	8	10339479	MSRA | LOC346702	ENST00000524047	ENSE00002116612	10339371	10339484	-0.434016035	0.312143712	0.590438026
rs533048	1	234808018	IRF2BP2 | PP2672	ENST00000442382	ENSE00001695948	234805269	234808736	-0.852284786	0.312667402	0.590438026
kgp10983201	2	47466221	CALM2 | BCYRN1	ENST00000444361	ENSE00001668926	47465872	47466346	-0.514705665	0.313955792	0.590438026
kgp18915434	12	43110560	PRICKLE1 | ADAMTS20	ENST00000550177	ENSE00002385666	43110293	43110653	0.464240065	0.314321665	0.590438026
rs225563	12	126447887	TMEM132B | LOC10028870	ENST00000502479	ENSE00002049964	126447557	126451454	0.54820912	0.315644773	0.590438026
kgp2079070	2	112369618	LOC541471 | LOC10028721	ENST00000426124	ENSE00001708549	112369479	112370095	0.440052547	0.317825796	0.590438026
kgp2531272	2	8704057	LOC339788 | ID2	ENST00000454224	ENSE00001713117	8699963	8708081	-0.51741512	0.318905264	0.590438026
kgp5054598	3	134067028	RYK | AMOTL2	ENST00000568384	ENSE00002597828	134066130	134068075	-0.476429193	0.320429898	0.590438026

kgp19236228	20	61668160	BHLHE23 | HAR1B	ENST00000370341	ENSE00001346988	61667400	61668380	0.482496047	0.322057105	0.590438026
rs170688	14	45234246	FSCB | C14orf28	ENST00000556405	ENSE00002460394	45232360	45234829	-1.045284259	0.265603105	0.591086795
kgp15368406	1	112142584	ADORA3 | RAP1A	ENST00000450155	ENSE00001793340	112142277	112142717	0.24971281	0.2659156	0.591086795
kgp3429237	16	22622722	LOC653786 | HS3ST2	ENST00000567401	ENSE00002582095	22621852	22623517	0.462068071	0.267231992	0.591816645
rs6074396	20	12224896	BTBD3 | LOC100289040	ENST00000455132	ENSE00001774029	12224817	12224944	-0.447265448	0.267554264	0.591816645
rs250671	5	148446523	SH3TC2 | ABLIM3	ENST00000507318	ENSE00002020377	148443630	148446548	0.27952478	0.267714903	0.591816645
kgp18120952	3	176373457	NAALADL2 | TBL1XR1	ENST00000428516	ENSE00001656471	176373343	176373457	-0.782116258	0.238094615	0.593021813
kgp6288157	6	160010135	FNDC1 | SOD2	ENST00000430078	ENSE00001713414	160009201	160010201	-0.724572965	0.239143995	0.593021813
rs4670222	2	38054623	LOC344382 | FAM82A1	ENST00000413792	ENSE00001797876	38053390	38055021	-1.32791075	0.24053211	0.593036065
kgp1625090	1	156660311	NES | CRABP2	ENST00000441272	ENSE00001669342	156657489	156660673	0.83680863	0.242124756	0.593551545
rs17506603	8	79750813	IL7 | STMN2	ENST00000565297	ENSE00002583800	79749764	79752757	-1.165196282	0.271058888	0.593559608
P1_M_061510_2	2	201691443	BZW1 | CLK1	ENST00000568571	ENSE00002589934	201689396	201692061	0.251244294	0.270081883	0.594895724
kgp10514626	16	81995543	PLCG2 | SDR42E1	ENST00000564138	ENSE00002630315	81993524	81996298	-0.635924713	0.270093502	0.594895724
kgp11945833	2	28671123	FOSL2 | PLB1	ENST00000439700	ENSE00001803576	28671034	28671737	0.447745317	0.327546505	0.595326714
rs6551627	4	62292785	IGFBP7 | LPHN3	ENST00000381217	ENSE00001385838	62292442	62293939	-0.522250697	0.328331703	0.595326714
kgp12345613	14	71107939	MED6 | TTC9	ENST00000500016	ENSE00001965431	71107725	71108015	-0.384012403	0.271097909	0.595692746
rs16867150	2	9899127	YWHAQ | TAF1B	ENST00000474667	ENSE00001869213	9897625	9899428	0.385975319	0.271442428	0.595692746
kgp7182161	21	16439965	NRIP1 | USP25	ENST00000449746	ENSE00001752929	16439856	16440158	0.374987984	0.272312601	0.595710526
kgp19953544	15	95026126	MCTP2 | LOC440311	ENST00000565106	ENSE00002296294	95024040	95027181	-0.260349328	0.272437622	0.595710526
kgp3657309	16	89113583	CBFA2T3 | ACSF3	ENST00000537498	ENSE00002244645	89112580	89114228	0.55316931	0.273420594	0.596778765
rs7248731	19	36822594	ZNF146 | ZFP14	ENST00000438368	ENSE00001799972	36822349	36822602	-0.774346746	0.275310748	0.597961168
kgp7458189	2	47559113	CALM2 | BCYRN1	ENST00000418539	ENSE00001641977	47558199	47571656	-0.409163611	0.275695051	0.597961168
kgp477052	5	149855119	RPS14 | NDST1	ENST00000519040	ENSE00002114422	149855094	149855322	-0.448868296	0.275881715	0.597961168
rs13242	16	2204835	RAB26 | SNORD60	ENST00000563192	ENSE00002629934	2204798	2204923	-0.531970539	0.276982017	0.597961168
kgp16397588	16	89112625	CBFA2T3 | ACSF3	ENST00000537498	ENSE00002244645	89112580	89114228	-0.913878171	0.277488527	0.597961168
rs185596	8	52935478	LOC100287313 | ST18	ENST00000521188	ENSE00002103365	52934976	52935534	0.299589981	0.278136586	0.597961168
kgp10153581	6	109089663	FOXO3 | ARMC2	ENST00000448744	ENSE00001698309	109089594	109090773	-0.397429488	0.278212772	0.597961168
kgp10081358	3	152557997	P2RY1 | LOC100287133	ENST00000460407	ENSE00001822402	152557179	152559228	-0.303679452	0.278219997	0.597961168
kgp7020023	7	17475792	AHR | SNX13	ENST00000454003	ENSE00001755535	17475654	17475857	-0.309200318	0.278421024	0.597961168
kgp16916784	6	141769878	CITED2 | LOC729076	ENST00000565399	ENSE00002622426	141768148	141772143	0.552911502	0.279499106	0.598638591
kgp545060	7	114765556	MDFIC | TFEC	ENST00000467677	ENSE00001823639	114765497	114765997	0.331529735	0.279884499	0.598638591
kgp22465327	5	10196102	LOC285692 | FAM173B	ENST00000566945	ENSE00002600148	10195233	10197740	-0.326778393	0.280224361	0.598638591
kgp28355849	15	95025291	MCTP2 | LOC440311	ENST00000565106	ENSE00002296294	95024040	95027181	-0.268608925	0.280733918	0.59866756
rs13148112	4	138118905	LOC646316 | PCDH18	ENST00000509798	ENSE00002058756	138118360	138119521	-1.237602847	0.246913614	0.601851934
kgp6726493	8	102179539	YWHAZ | ZNF706	ENST00000565617	ENSE00002623035	102179033	102181857	-1.086528268	0.283130889	0.602088561
kgp3624248	2	9898039	YWHAQ | TAF1B	ENST00000474667	ENSE00001869213	9897625	9899428	-0.448387463	0.283335793	0.602088561
kgp2765214	1	84266820	LPHN2 | TTLL7	ENST00000439186	ENSE00001799963	84266722	84267148	-0.814721573	0.277004867	0.602184494
kgp24159504	2	166651479	GALNT3 | TTC21B	ENST00000428888	ENSE00001662010	166651367	166651599	-0.259961836	0.284092263	0.602551178
rs7896078	10	5652277	CALML3 | ASB13	ENST00000425246	ENSE00001777052	5652277	5652399	0.463042916	0.284985835	0.602551178
kgp7602091	1	116971043	C1orf203 | CD58	ENST00000423907	ENSE00001716889	116971004	116971116	-0.684205183	0.285051137	0.602551178
kgp7665436	16	66923588	PDP2 | CDH16	ENST00000563086	ENSE00002616204	66923094	66924996	-0.368888969	0.286304824	0.603647091
kgp4944991	1	223318105	TLR5 | SUSD4	ENST00000435108	ENSE00001645603	223317879	223318296	-0.36245703	0.286569829	0.603647091
kgp8510787	15	38332294	TMCO5A | LOC728288	ENST00000558081	ENSE00002554814	38332197	38333188	0.718036138	0.249061002	0.603656326
kgp13161871	21	41101700	B3GALT5 | IGSF5	ENST00000457325	ENSE00001686404	41101431	41102607	-0.567773911	0.337715341	0.604814361
kgp17284619	6	24751883	C6orf62 | GMNN	ENST00000453179	ENSE00001787704	24751793	24752149	0.481725933	0.339034569	0.604814361
rs11862324	16	72462749	PMFBP1 | ZFHX3	ENST00000564508	ENSE00002602918	72459847	72463072	-0.455044153	0.339062596	0.604814361
kgp7016080	4	142245708	ZNF330 | LOC100286983	ENST00000509161	ENSE00002080359	142244576	142247161	-0.668760294	0.341556052	0.605986544
rs12071518	1	234805957	IRF2BP2 | PP2672	ENST00000442382	ENSE00001695948	234805269	234808736	0.579704213	0.290414836	0.609684894
rs6881826	5	4515447	IRX1 | LOC340094	ENST00000503188	ENSE00002026322	4515436	4516889	-0.658526765	0.290580021	0.609684894
kgp4664965	12	110325994	GLTP | TCHP	ENST00000446473	ENSE00002242321	110325598	110326272	0.357833088	0.291205008	0.609684894
kgp25773634	16	49329018	CBLN1 | C16orf78	ENST00000569877	ENSE00002612536	49328797	49329877	0.247705782	0.291456656	0.609684894
kgp17722458	3	176571516	NAALADL2 | TBL1XR1	ENST00000428516	ENSE00001594167	176571497	176571556	-0.484114262	0.292042126	0.609852675
rs9532150	13	38628041	TRPC4 | UFM1	ENST00000454060	ENSE00001783231	38627835	38628306	0.268133609	0.293516139	0.611294994
kgp13135901	21	35335785	FLJ46020 | MRPS6	ENST00000381181	ENSE00001487739	35334367	35336260	0.424624547	0.29374573	0.611294994
rs9348512	6	10456706	C6orf218 | GCNT2	ENST00000366312	ENSE00001441402	10456669	10457014	0.28039655	0.294352304	0.611502979
kgp23411958	7	80239	LOC100132858 | LOC10028	ENST00000478759	ENSE00001902048	80130	80418	-0.605959867	0.347855536	0.613862711
rs11868320	17	27875213	TAOK1 | ABHD15	ENST00000562535	ENSE00002584590	27873905	27878921	-0.31084734	0.296922121	0.614586958
kgp4457737	17	70030083	LOC124685 | SOX9	ENST00000543512	ENSE00002217013	70030040	70030233	-0.919952216	0.297243692	0.614586958
kgp12308355	6	10453056	C6orf218 | GCNT2	ENST00000366312	ENSE00001744731	10452981	10453060	0.236019554	0.297364361	0.614586958
kgp8908843	16	80632501	DYNLRB2 | CDYL2	ENST00000570137	ENSE00002617613	80631803	80636416	0.278078268	0.297896535	0.614634389
kgp17329383	6	109090659	FOXO3 | ARMC2	ENST00000448744	ENSE00001698309	109089594	109090773	-0.427968328	0.350731264	0.615645303
rs17275065	21	16742975	NRIP1 | USP25	ENST00000449602	ENSE00001624296	16742841	16743230	-1.737748269	0.256001084	0.616991375
kgp2910387	16	27183176	C16orf82 |	ENST00000564381	ENSE00002612841	27183151	27183300	0.752728796	0.257966392	0.618254649
kgp19347071	20	37034770	LBP | LOC388796	ENST00000422519	ENSE00001803279	37034658	37034969	0.362612424	0.300922475	0.61877992
kgp2887778	7	153108673	LOC100288889 | LOC10013	ENST00000416982	ENSE00001637005	153108667	153111048	-0.309567562	0.30093108	0.61877992
kgp5342909	20	6508010	FERMT1 | BMP2	ENST00000415932	ENSE00001785606	6507841	6509106	0.430235462	0.301954017	0.61950468
kgp8357764	2	139370451	SPOPL | NXPH2	ENST00000453636	ENSE00001804638	139370108	139370737	0.402420849	0.302310072	0.61950468
rs7439909	4	106058718	LOC728847 | TET2	ENST00000504082	ENSE00002021304	106058437	106058875	-0.80544266	0.303395548	0.619960075
rs4702392	5	6796405	POLS | LOC442132	ENST00000508881	ENSE00002021213	6795993	6796458	0.629503509	0.303787369	0.619960075
kgp11705444	7	65993766	LOC346329 | LOC493754	ENST00000449307	ENSE00001674867	65993425	65996858	-0.623800168	0.304292879	0.619960075
rs515347	6	81177227	BCKDHB | FAM46A	ENST00000569267	ENSE00002590559	81176675	81178797	-0.33130529	0.304692248	0.619960075
kgp6708936	17	27875403	TAOK1 | ABHD15	ENST00000562535	ENSE00002584590	27873905	27878921	-0.346494144	0.305103743	0.619960075
rs6729885	2	231556273	SP100 | CAB39	ENST00000415174	ENSE00001768231	231555636	231556948	0.647111272	0.305898715	0.619960075
rs3815694	5	175612177	LOC643201 | C5orf25	ENST00000512260	ENSE00002068798	175612104	175612183	0.50532588	0.306127759	0.619960075
kgp3937676	11	67655784	LOC645332 | LOC10013226	ENST00000533670	ENSE00002175549	67655624	67655795	-0.338134938	0.307062182	0.620002618
kgp10470462	8	99383729	NIPAL2 | KCNS2	ENST00000518704	ENSE00002131207	99383583	99383906	-0.30245495	0.307833466	0.620002618
kgp10372493	20	31445147	MAPRE1 | EFCAB8	ENST00000569087	ENSE00002597460	31444848	31446557	-0.583346948	0.308200263	0.620002618
rs10099070	8	135805476	ZFAT | LOC286094	ENST00000568248	ENSE00002623781	135804263	135810515	-0.224811021	0.308203456	0.620002618
kgp24126268	2	221568761	SLC4A3 | EPHA4	ENST00000414512	ENSE00001798750	221568123	221569286	1.294157833	0.260369633	0.620547625
kgp3199114	17	17095310	MPRIP | PLD6	ENST00000567268	ENSE00002611064	17091982	17095962	0.223973071	0.31004209	0.621820509
rs3809472	15	45694318	GATM | SPATA5L1	ENST00000458245	ENSE00001988574	45694097	45694525	0.275905005	0.311049409	0.621820509
kgp11361601	15	25277609	PAR5 | SNORD109A	ENST00000552334	ENSE00002349489	25277020	25281637	0.861508935	0.311650878	0.621820509
kgp18435640	9	37090324	LOC100287249 | ZCCHC7	ENST00000429493	ENSE00001770690	37086665	37090398	-0.26757156	0.3116957	0.621820509
kgp19946040	15	44827663	CTDSPL2 | LOC645212	ENST00000560750	ENSE00002536899	44827461	44827672	-0.655659609	0.311819325	0.621820509
kgp25818454	16	21806228	OTOA | LOC730092	ENST00000567370	ENSE00002577410	21805416	21807080	0.391616276	0.312198201	0.621820509
rs10515946	2	208038537	KLF7 | CREB1	ENST00000435643	ENSE00001613406	208038358	208038538	0.377771931	0.313022662	0.622247458

kgp3337094	9	33605081	LOC100288689 | ANXA2P2	ENST00000433357	ENSE00001611427	33605009	33605291	0.269654979	0.313523676	0.622247458
kgp20379703	8	102087458	YWHAZ | ZNF706	ENST00000514926	ENSE00002063679	102087397	102088479	0.663574514	0.314134739	0.622247458
kgp2355868	6	81178549	BCKDHB | FAM46A	ENST00000569267	ENSE00002590559	81176675	81178797	-0.354765401	0.315334637	0.622247458
kgp2192533	1	96242713	LOC729977 | LOC10028691	ENST00000456933	ENSE00001738832	96242703	96242934	-0.614922813	0.31540289	0.622247458
kgp12936726	11	69187125	MYEOV | CCND1	ENST00000561588	ENSE00002612930	69186994	69187279	-0.635414232	0.315505753	0.622247458
kgp14812196	2	96331868	LOC100287711 | LOC64321	ENST00000425887	ENSE00001615342	96331832	96332151	-0.50135619	0.317089716	0.624351203
kgp3032029	9	93763993	SYK | LOC100128909	ENST00000563268	ENSE00002600916	93763052	93764153	0.803969379	0.317727019	0.624587154
kgp18713822	12	126451215	TMEM132B | LOC10028870	ENST00000545784	ENSE00002228181	126450746	126451888	1.140983754	0.264296317	0.626426076
rs10494494	1	175874139	TNR | RFWD2	ENST00000426575	ENSE00001678929	175873898	175874272	0.269784448	0.320389459	0.628609774
rs2326224	8	120258996	MAL2 | NOV	ENST00000524129	ENSE00002115818	120258970	120259083	-0.34783793	0.320993777	0.628609774
kgp1027081	8	102087625	YWHAZ | ZNF706	ENST00000514926	ENSE00002063679	102087397	102088479	0.72152002	0.321734402	0.628609774
kgp5311720	2	95873379	ZNF2 | LOC344065	ENST00000425953	ENSE00001732426	95873283	95873582	-0.394658222	0.321856537	0.628609774
kgp7227595	5	57412297	ACTBL2 | PLK2	ENST00000505861	ENSE00002022169	57412289	57412478	0.608383887	0.361719391	0.628812835
rs1266264	14	46184595	LOC644589 | LOC10028958	ENST00000555442	ENSE00002532610	46184553	46185155	-0.918684416	0.364421101	0.628812835
rs10147143	14	71076428	MED6 | TTC9	ENST00000500016	ENSE00001980392	71075515	71078067	-0.695681134	0.365774286	0.628812835
kgp7452615	16	49205189	N4BP1 | CBLN1	ENST00000564222	ENSE00002605351	49204463	49205697	0.474985343	0.36585474	0.628812835
kgp24437796	2	221662424	SLC4A3 | EPHA4	ENST00000424395	ENSE00001644598	221662181	221662613	0.734194444	0.291555519	0.629256515
kgp7791273	13	48505679	HTR2A | SUCLA2	ENST00000566385	ENSE00002595049	48504290	48506757	-0.341738552	0.322846126	0.629523868
kgp24152783	2	227669041	IRS1 | RHBDD1	ENST00000567305	ENSE00002618058	227668752	227669777	-0.582546024	0.324415288	0.63077139
kgp2571569	9	132121671	C9orf106 | LOC100128077	ENST00000423122	ENSE00001601157	132121615	132121817	-0.355025586	0.324531096	0.63077139
kgp16325156	16	50673537	NKD1 | SNX20	ENST00000565077	ENSE00002621561	50671504	50674771	0.543632317	0.370245677	0.632001225
kgp8687931	4	62292761	IGFBP7 | LPHN3	ENST00000381217	ENSE00001385838	62292442	62293939	-0.456210515	0.371540114	0.632001225
kgp428663	14	85995614	SEL1L | FLRT2	ENST00000380722	ENSE00001486021	85994961	85996332	0.220037605	0.326779338	0.634120034
kgp4355400	17	80252227	CSNK1D | CD7	ENST00000566986	ENSE00002591651	80251592	80252786	-0.231478473	0.327674313	0.634836108
kgp9709280	12	2039690	LOC100288635 | DCP1B	ENST00000418006	ENSE00001804023	2038368	2040596	-0.686778858	0.270937692	0.635995906
kgp5680503	8	83542196	SNX16 | RALYL	ENST00000522776	ENSE00002129465	83542158	83542270	1.10003603	0.272004036	0.635995906
kgp3984971	12	102349653	DRAM | CCDC53	ENST00000550307	ENSE00002337214	102348776	102349668	-0.737531736	0.274188526	0.635995906
kgp7833046	14	101537913	SNORD114-31 | LOC10013	ENST00000444846	ENSE00001687158	101537124	101539271	1.021620734	0.274263969	0.635995906
kgp21512157	19	43330858	LOC100289650 | PSG10	ENST00000425668	ENSE00001777489	43330585	43331030	0.369159512	0.329582258	0.636741245
kgp11661290	10	131865429	EBF3 | LOC387723	ENST00000456581	ENSE00001788710	131864638	131866872	-0.326307352	0.33032706	0.636741245
kgp438096	14	101539090	SNORD114-31 | LOC10013	ENST00000554016	ENSE00002513162	101538696	101539274	0.559155363	0.331330676	0.636741245
kgp2355775	16	66445923	CDH5 | BEAN	ENST00000499966	ENSE00001976529	66444183	66446038	-0.301120907	0.331396984	0.636741245
kgp10254238	12	133488171	CHFR | LOC100289635	ENST00000503695	ENSE00002081172	133488056	133489173	-0.236321953	0.331664414	0.636741245
kgp22784493		39262981	LOC100289469 | BCOR	ENST00000438867	ENSE00001687518	39262855	39263139	0.254785113	0.331867754	0.636741245
rs4868306	5	173138522	BOD1 | CPEB4	ENST00000521128	ENSE00002126658	173137443	173138951	0.34435594	0.332427543	0.636741245
kgp29704051	10	92259368	LOC119358 | HTR7	ENST00000422206	ENSE00001666560	92259030	92261197	0.805022777	0.332877983	0.636741245
kgp10610742	2	3129079	MYT1L | LOC729897	ENST00000457478	ENSE00001720874	3129067	3129210	-0.703974462	0.297827908	0.63685177
kgp733804	9	93145839	LOC100129066 | LOC34051	ENST00000425666	ENSE00001605021	93145802	93146003	-0.692337841	0.301111124	0.63685177
kgp30236381	5	57047079	ACTBL2 | PLK2	ENST00000502647	ENSE00002046459	57047019	57047544	0.662350145	0.301443171	0.63685177
rs12889775	14	77426077	C14orf166B | C14orf4	ENST00000553613	ENSE00002443575	77425981	77426096	0.491611447	0.335442427	0.640631344
GA028577	12	3151456	TEAD4 | TSPAN9	ENST00000513358	ENSE00002302855	3150603	3152614	-0.930217995	0.378592426	0.640694875
kgp11890922	16	79755348	MAF | DYNLRB2	ENST00000567993	ENSE00002590366	79755209	79755518	-0.70323967	0.308741276	0.640981096
kgp14688393	2	1624918	TPO | PXDN	ENST00000366424	ENSE00001647238	1624282	1625751	0.628226161	0.315132175	0.640981096
rs2587155	8	79750564	IL7 | STMN2	ENST00000565297	ENSE00002583800	79749764	79752757	-0.764796494	0.316027367	0.640981096
kgp5287900	16	33957004	LOC100287647 | TOP	ENST00000567668	ENSE00002612584	33956915	33957115	-0.630567947	0.317325881	0.640981096
kgp18452451	9	129280717	FAM125B | LMX1B	ENST00000454034	ENSE00001806230	129280634	129281087	0.622601544	0.318758051	0.640981096
rs6998516	8	20146804	LZTS1 | GFRA2	ENST00000523103	ENSE00002116374	20146720	20146819	0.834738922	0.32070069	0.640981096
kgp2743522	6	3905809	LOC100289591 | PRPF4B	ENST00000566733	ENSE00002617674	3905144	3912213	0.706791132	0.323835633	0.640981096
rs10761343	9	93145905	LOC100129066 | LOC34051	ENST00000436671	ENSE00001632139	93145892	93146003	-0.676252749	0.32633788	0.640981096
rs11649081	16	21314612	CRYM | NCRNA00169	ENST00000444326	ENSE00001737705	21314568	21314778	-0.683644006	0.328647932	0.640981096
rs2017328	13	33909346	STARD13 | RFC3	ENST00000443576	ENSE00001780719	33909142	33909403	-0.752818432	0.329101604	0.640981096
kgp20017323	15	81283841	MESDC2 | MESDC1	ENST00000563737	ENSE00002624578	81283145	81285599	-0.623668972	0.330099528	0.640981096
kgp914817	16	83832493	CDH13 | HSBP1	ENST00000567109	ENSE00002630405	83831266	83834245	0.606727771	0.330148656	0.640981096
rs11692804	2	221662586	SLC4A3 | EPHA4	ENST00000424395	ENSE00001644598	221662181	221662613	0.686562024	0.331173566	0.640981096
kgp12823839	11	65191922	FRMD8 | NCRNA00084	ENST00000501122	ENSE00001961965	65190269	65213011	-0.211546724	0.336205957	0.641075182
kgp4508243	1	17522822	LOC400743 | PADI1	ENST00000539219	ENSE00002261175	17520556	17524112	-0.473401351	0.338659454	0.644734954
kgp28694963	8	135845249	ZFAT | LOC286094	ENST00000521444	ENSE00002111866	135844990	135845608	-0.608220031	0.335307434	0.644821988
kgp18314958	9	44261311	LOC728832 | LOC10028954	ENST00000439824	ENSE00001686690	44260422	44263319	-0.633395716	0.339335263	0.645155824
rs921392	5	4515553	IRX1 | LOC340094	ENST00000503188	ENSE00002026322	4515436	4516889	-0.751546406	0.34122303	0.645155824
kgp19128476	12	10551026	KLRK1 | KLRC4	ENST00000500682	ENSE00001969973	10548778	10551105	-0.663846746	0.342203621	0.645155824
kgp19527737	14	50428543	ARF6 | C14orf182	ENST00000556913	ENSE00002460979	50428421	50428678	-0.606823967	0.344083106	0.645155824
kgp5576434	16	81420621	GAN | CMIP	ENST00000568107	ENSE00002626991	81416874	81424489	-0.575215927	0.340394698	0.645884453
kgp12928916	11	58265822	OR5B12 | OR5B21	ENST00000531715	ENSE00002174597	58265646	58265863	0.33741976	0.340537543	0.645884453
kgp25171799	1	234664957	TARBP1 | IRF2BP2	ENST00000435574	ENSE00001674386	234663637	234666088	-0.468470226	0.341734618	0.645884453
rs9679195	2	20878719	GDF7 | C2orf43	ENST00000565841	ENSE00002602242	20877569	20879005	0.227672815	0.341854384	0.645884453
kgp20276433	8	127340735	LOC650095 | FAM84B	ENST00000500180	ENSE00001990119	127340553	127341780	0.560401948	0.341938828	0.645884453
kgp6496571	12	106445852	C12orf75 | NUAK1	ENST00000546699	ENSE00002371121	106445747	106445953	-0.683215609	0.280082565	0.645996884
rs7906617	10	131984960	GLRX3 | TCERG1L	ENST00000440388	ENSE00001697992	131984687	131985130	-1.038962371	0.282114903	0.647204777
rs1047799	17	17095487	MPRIP | PLD6	ENST00000567268	ENSE00002611064	17091982	17095962	-0.247399368	0.343644098	0.648091291
rs680757		90563860	LOC100288789 | PABPC5	ENST00000418369	ENSE00001679583	90563289	90563877	0.585725449	0.348227234	0.648870623
rs10505278	8	117271922	TRPS1 | EIF3H	ENST00000505156	ENSE00002027873	117271725	117272264	0.213099346	0.344874123	0.64939636
kgp24138444	2	75169534	HK2 | POLE4	ENST00000453951	ENSE00001643565	75169014	75169797	0.372932833	0.345789919	0.650106591
kgp17495903	6	81172867	BCKDHB | FAM46A	ENST00000443221	ENSE00001751052	81172589	81172924	-0.329378085	0.347220479	0.651780899
kgp1769523	22	25608776	CRYBB3 | CRYBB2	ENST00000454253	ENSE00001665821	25608709	25608807	0.207701899	0.348994977	0.653126875
kgp6639677	8	130695894	LOC100287906 | GSDMC	ENST00000522667	ENSE00002129228	130695818	130695925	-0.375368197	0.349019747	0.653126875
kgp7443541	12	93501170	LOC100287580 | NUDT4	ENST00000551928	ENSE00002372876	93501084	93501376	0.650635811	0.288212836	0.653161451
kgp12314649	7	33833205	LOC100288021 | BMPER	ENST00000420185	ENSE00001771988	33832780	33834159	0.685916469	0.288748427	0.653161451
kgp4867215	8	20832136	LZTS1 | GFRA2	ENST00000522604	ENSE00002112320	20831497	20832164	0.683135587	0.290854787	0.653161451
kgp12332612	10	87209779	LOC100131699 | GRID1	ENST00000441457	ENSE00001594363	87209349	87209933	0.636278091	0.291256487	0.653161451
kgp9296682	2	67516447	LOC100289292 | ETAA1	ENST00000452716	ENSE00001675571	67516215	67516556	0.624985199	0.297941733	0.653161451
kgp8285617	9	26801887	TUSC1 | C9orf82	ENST00000521572	ENSE00002109868	26801731	26802058	-1.05308421	0.299464089	0.653161451
rs2112090	2	207686727	FASTKD2 | CPO	ENST00000561915	ENSE00002617758	207685914	207687018	-0.629988384	0.30009025	0.653161451
kgp7757938	6	168396971	HGC6.3 | KIF25	ENST00000456585	ENSE00001778623	168396886	168397757	0.619899226	0.300804562	0.653161451
kgp4497385	1	221004092	MOSC1 | LOC100129376	ENST00000431347	ENSE00001673900	221004035	221004249	0.641762938	0.301267752	0.653161451
rs13015944	2	213681716	ERBB4 | LOC646249	ENST00000453965	ENSE00001784368	213681647	213681731	-0.693921084	0.301754963	0.653161451
kgp12276934	2	65074479	SERTAD2 | SLC1A4	ENST00000445865	ENSE00001755691	65073264	65074746	0.832195858	0.302204022	0.653161451

rs7620839	3	149956598	TMEM183B | TSC22D2	ENST00000498005	ENSE00001857016	149956487	149956617	0.640366312	0.304653526	0.653161451
rs564779	21	44785422	FLJ41733 | SIK1	ENST00000435702	ENSE00001758406	44783212	44785567	0.615381656	0.305189318	0.653161451
kgp379054	6	33857482	MLN | GRM4	ENST00000506222	ENSE00002057806	33857288	33860228	0.637246319	0.306026694	0.653161451
kgp5782917	18	77838977	C18orf22 | ADNP2	ENST00000566810	ENSE00002581238	77838683	77839140	-0.306093275	0.349747813	0.653476176
kgp19660340	14	39644368	TRAPPC6B | PNN	ENST00000556537	ENSE00002477748	39644089	39644516	0.595003218	0.353155447	0.653991569
kgp28171514	14	77252935	VASH1 | ANGEL1	ENST00000556072	ENSE00002525376	77252428	77253067	-0.43616693	0.351667661	0.654952068
kgp6753706	12	69069079	RAP1B | NUP107	ENST00000500695	ENSE00001974668	69068151	69069601	0.342832195	0.352025241	0.654952068
kgp10754770	17	17095644	MPRIP | PLD6	ENST00000567268	ENSE00002611064	17091982	17095962	0.285822039	0.352515633	0.654952068
kgp2056619	9	137475635	RXRA | COL5A1	ENST00000423455	ENSE00001806378	137475038	137477036	0.196264442	0.352708239	0.654952068
kgp2400459	20	56532263	PMEPA1 | LOC100129869	ENST00000371169	ENSE00001454531	56532182	56532545	0.229411141	0.354009341	0.655648714
kgp2645534	14	101538792	SNORD114-31 | LOC10013(	ENST00000554016	ENSE00002513162	101538696	101539274	0.331638061	0.354169811	0.655648714
kgp24260716	2	8861392	ID2 | KIDINS220	ENST00000569008	ENSE00002625681	8861264	8862816	-0.40880854	0.354987809	0.655728358
rs9669611	12	6503786	LTBR | SRP14P1	ENST00000541888	ENSE00002315622	6503673	6504235	0.223980751	0.355299375	0.655728358
kgp1720402	14	21513410	RNASE7 | RNASE8	ENST00000554568	ENSE00002516697	21511515	21514097	0.246718482	0.35674798	0.65717898
kgp8636503	15	95024591	MCTP2 | LOC440311	ENST00000565106	ENSE00002296294	95024040	95027181	0.19590514	0.357174326	0.65717898
kgp10316069	1	163229838	RGS5 | NUF2	ENST00000416401	ENSE00001628182	163229640	163229998	-0.641588124	0.3591914	0.657325834
kgp20738315	4	142245811	ZNF330 | LOC100286983	ENST00000509161	ENSE00002080359	142244576	142247161	-0.677957545	0.359671977	0.657325834
kgp325656	7	26591573	KIAA0087 | LOC285941	ENST00000457000	ENSE00001596777	26591458	26591829	0.573790211	0.361529209	0.657325834
kgp10635077	10	128102639	ADAM12 | C10orf90	ENST00000456514	ENSE00001685542	128102438	128103307	-0.51020551	0.390427956	0.657353191
rs2289804	10	69993032	ATOH7 | PBLD	ENST00000444086	ENSE00001694139	69993008	69993330	-0.78987047	0.365122459	0.657582384
kgp2989086	3	177039320	TBL1XR1 | KCNMB2	ENST00000425388	ENSE00001591376	177039217	177041206	0.626775621	0.366054194	0.657582384
rs2272450	6	148338672	SAMD5 | SASH1	ENST00000422023	ENSE00001797597	148338558	148338701	0.612674863	0.311156787	0.658908275
rs9306023	21	23109608	NCAM2 | NCRNA00158	ENST00000419069	ENSE00001715952	23109572	23109639	0.656876908	0.311791095	0.658908275
kgp5168523	16	66786276	DYNC1LI2 | CCDC79	ENST00000501143	ENSE00001985625	66785655	66786803	-0.346074145	0.359191392	0.659884339
kgp12194836	1	180535383	ACBD6 | XPR1	ENST00000442621	ENSE00001599647	180534387	180535654	-0.811584978	0.359751335	0.659908604
rs7150866	14	71076334	MED6 | TTC9	ENST00000500016	ENSE00001980392	71075515	71078067	-0.500341457	0.361670386	0.660489722
rs1985288	16	80632586	DYNLRB2 | CDYL2	ENST00000570137	ENSE00002617613	80631803	80636416	0.34061866	0.361813262	0.660489722
kgp5763752	1	159948847	SLAMF9 | PIGM	ENST00000423943	ENSE00001610015	159948390	159948851	-0.267659697	0.362048383	0.660489722
kgp3843094	9	37088104	LOC100287249 | ZCCHC7	ENST00000429493	ENSE00001770690	37086665	37090398	-0.223445163	0.362415507	0.660489722
kgp7722986	13	48504568	HTR2A | SUCLA2	ENST00000566385	ENSE00002595049	48504290	48506757	0.328691077	0.362804214	0.660489722
rs10749896	11	107184828	GUCY1A2 | CWF19L2	ENST00000561746	ENSE00002621788	107182858	107186997	-0.243203147	0.363678511	0.661084281
rs4411509	16	79784626	MAF | DYNLRB2	ENST00000566729	ENSE00002626243	79784589	79785173	-0.313108208	0.364421296	0.661438352
kgp22279620	5	95550829	ELL2 | PCSK1	ENST00000507997	ENSE00002057926	95550679	95551223	0.622461284	0.315424165	0.663318465
kgp20735787	4	142245012	ZNF330 | LOC100286983	ENST00000509161	ENSE00002080359	142244576	142247161	-0.627137602	0.396999392	0.664869442
rs12679969	8	102087549	YWHAZ | ZNF706	ENST00000514926	ENSE00002063679	102087397	102088479	0.629316526	0.401282225	0.664869442
kgp18759266	12	3434277	LOC100128253 | PRMT8	ENST00000543036	ENSE00002292692	3434256	3434506	0.375957991	0.401443291	0.664869442
kgp11981208	7	65994162	LOC346329 | LOC493754	ENST00000449307	ENSE00001674867	65993425	65996858	-0.55228923	0.403421137	0.664869442
kgp19824907	15	93323660	LOC643797 | CHD2	ENST00000562894	ENSE00002583293	93322987	93324722	0.367345658	0.404965933	0.664869442
kgp5771754	3	114034138	TIGIT | ZBTB20	ENST00000570269	ENSE00002576360	114033348	114035026	-0.255026807	0.367041695	0.664964837
kgp2418733	16	81422315	GAN | CMIP	ENST00000568107	ENSE00002626991	81416874	81424489	-0.512204131	0.36746607	0.664964837
kgp3887478	1	48226825	LOC100287946 | LOC10028	ENST00000438589	ENSE00001628376	48226804	48226952	-0.311747802	0.368088485	0.665094013
kgp19079470	12	34209167	ALG10 | LOC100130830	ENST00000537655	ENSE00002322254	34208992	34209394	0.262961756	0.369804221	0.667195359
kgp103500	1	95086815	F3 | SLC44A3	ENST00000435559	ENSE00001601116	95086707	95087512	-0.34530313	0.370525651	0.667499196
kgp5465458	11	65210748	NCRNA00084 | MALAT1	ENST00000501122	ENSE00001961965	65190269	65213011	-0.288701918	0.3712183	0.667750355
kgp20470213	8	128085043	FAM84B | POU5F1B	ENST00000523510	ENSE00002108688	128084939	128085163	-0.208438618	0.372478228	0.668077798
kgp27457669	9	37088261	LOC100287249 | ZCCHC7	ENST00000429493	ENSE00001770690	37086665	37090398	-0.203839595	0.373198426	0.668077798
kgp2394494	2	231556882	SP100 | CAB39	ENST00000415174	ENSE00001768231	231555636	231556948	0.22485269	0.373808006	0.668077798
kgp5127219	2	139228879	LOC100129375 | SPOPL	ENST00000412593	ENSE00001802345	139228610	139228901	-0.267433271	0.374106692	0.668077798
rs1958032	14	21513536	RNASE7 | RNASE8	ENST00000554568	ENSE00002516697	21511515	21514097	-0.97664704	0.374167847	0.668077798
kgp10593256	4	128391624	FAT4 | LOC729424	ENST00000509671	ENSE00002064193	128391590	128391724	0.673177382	0.374855282	0.669341834
kgp30649106		135924637	ARHGEF6 | LOC100128416	ENST00000435597	ENSE00001714609	135923090	135925008	-0.5706968	0.378115054	0.669341834
rs11203833	8	16989032	EFHA2 | ZDHHC2	ENST00000513892	ENSE00002077855	16988690	16990578	-0.586938773	0.379293706	0.669341834
rs4531246	1	2362020	PEX10 | PLCH2	ENST00000420406	ENSE00001621165	2361777	2362089	-0.689456635	0.376039883	0.669443662
kgp20675749	4	47843071	CORIN | NFXL1	ENST00000563286	ENSE00002580194	47842139	47846356	0.182682855	0.37604209	0.669443662
kgp2961877	1	17523049	LOC400743 | PADI1	ENST00000539219	ENSE00002261175	17520556	17524112	-0.367581075	0.377083344	0.670308684
kgp9861059	16	81424042	GAN | CMIP	ENST00000568107	ENSE00002626991	81416874	81424489	0.181355284	0.3789634	0.672660035
rs2289150	16	66442499	CDH5 | BEAN	ENST00000499966	ENSE00001972605	66442427	66442717	0.433969084	0.379840025	0.673009284
rs10940291	5	52409259	LOC257396 | FST	ENST00000499459	ENSE00001982677	52408070	52410956	-0.270530633	0.380826924	0.673009284
rs13224057	7	65993792	LOC346329 | LOC493754	ENST00000449307	ENSE00001674867	65993425	65996858	-0.593483663	0.381371996	0.673009284
rs10431347	12	3153694	TEAD4 | TSPAN9	ENST00000513358	ENSE00002234190	3152644	3154116	-0.318525166	0.381390514	0.673009284
kgp13284555	7	130121403	TSGA14 | MEST	ENST00000562524	ENSE00002587446	130121332	130124233	-0.644415576	0.386042714	0.673171413
kgp14664966	2	75159901	HK2 | POLE4	ENST00000435984	ENSE00001619573	75159788	75159921	0.594841331	0.386236629	0.673171413
kgp8970629	14	50409096	ARF6 | C14orf182	ENST00000556913	ENSE00002499815	50408654	50410610	-0.573530848	0.388195515	0.673171413
rs2839586	21	44251223	PDE9A | WDR4	ENST00000431150	ENSE00001661955	44251150	44251315	-0.65319813	0.321782903	0.673389587
kgp24965505	1	14548574	PRDM2 | KIAA1026	ENST00000449215	ENSE00001604030	14548386	14548602	-0.688753565	0.324376083	0.673664591
kgp3829092	7	22930683	SNORD93 | FAM126A	ENST00000421730	ENSE00001671326	22930554	22930768	0.587426926	0.325562006	0.673664591
kgp22133922	5	175570290	FAM153B | LOC643201	ENST00000515403	ENSE00002052084	175570088	175570629	0.582400806	0.326625256	0.673664591
kgp10374467	12	4809841	NDUFA9 | GALNT8	ENST00000527518	ENSE00002151206	4809583	4810345	0.435157254	0.38359844	0.674218965
kgp916870	3	176322288	NAALADL2 | TBL1XR1	ENST00000434969	ENSE00001760449	176321931	176322474	-0.461082196	0.384004351	0.674218965
kgp25746828	16	63093740	CDH8 | CDH11	ENST00000568741	ENSE00002616828	63091122	63093812	0.376194306	0.384085291	0.674218965
kgp19970574	15	95025601	MCTP2 | LOC440311	ENST00000565106	ENSE00002296294	95024040	95027181	-0.245872651	0.384310396	0.674218965
kgp15737156	1	234663881	TARBP1 | IRF2BP2	ENST00000435574	ENSE00001674386	234663637	234666088	-0.715844004	0.38526724	0.674916631
kgp564075	7	89941884	C7orf63 | GTPBP10	ENST00000445784	ENSE00001665847	89941810	89942114	-0.546379217	0.39399867	0.675460879
kgp13189616	21	39695599	KCNJ15 | ERG	ENST00000414189	ENSE00001639894	39695557	39695820	0.528694409	0.394018846	0.675460879
kgp23203635	11	65192125	FRMD8 | NCRNA00084	ENST00000501122	ENSE00001961965	65190269	65213011	-0.182730445	0.389254747	0.67851677
kgp2719542	11	68640990	CPT1A | MRPL21	ENST00000512200	ENSE00002055373	68639011	68642010	0.295528362	0.389668995	0.67851677
rs4837360	9	132109628	C9orf106 | LOC100128077	ENST00000444125	ENSE00001697151	132109505	132109743	-0.766269032	0.389938623	0.67851677
rs10395	16	80632290	DYNLRB2 | CDYL2	ENST00000570137	ENSE00002617613	80631803	80636416	0.311567461	0.390708444	0.67851677
kgp17613194	3	152557560	P2RY1 | LOC100287133	ENST00000460407	ENSE00001822402	152557179	152559228	-0.293956532	0.391344768	0.67851677
kgp11724505	4	11775236	HS3ST1 | HSP90AB2P	ENST00000515286	ENSE00002038589	11775217	11775288	-0.379060651	0.391944379	0.67851677
rs11720805	3	156800360	LEKR1 | CCNL1	ENST00000471357	ENSE00001879700	156799456	156801064	0.323544953	0.393163678	0.67851677
kgp2485265	5	148445390	SH3TC2 | ABLIM3	ENST00000507318	ENSE00002020377	148443630	148446548	0.17366358	0.393289695	0.67851677
kgp4943335	1	159946796	SLAMF9 | PIGM	ENST00000443364	ENSE00001751381	159946434	159946902	-0.25155904	0.393492843	0.67851677
rs6799987	3	73919008	LOC100129282 | CNTN3	ENST00000498832	ENSE00001816260	73918927	73919112	0.198844316	0.394013085	0.67851677
kgp10924700	12	3153587	TEAD4 | TSPAN9	ENST00000513358	ENSE00002234190	3152644	3154116	-0.439074331	0.394504961	0.67851677
kgp4567448	9	93944753	LOC100128909 | AUH	ENST00000439438	ENSE00001743018	93944751	93945044	0.222042062	0.394526769	0.67851677

kgp6084741	5	106151169	RAB9P1 | LOC100287833	ENST00000513273	ENSE00002030686	106150898	106151247	-0.591674826	0.394908596	0.67851677
rs28704789	9	98178056	FANCC | PTCH1	ENST00000433644	ENSE00001650490	98177781	98178446	0.221233535	0.395192452	0.67851677
kgp847387	1	17522551	LOC400743 | PADI1	ENST00000539219	ENSE00002261175	17520556	17524112	-0.575466015	0.332364647	0.679588088
kgp8081869	8	73861028	LOC100288310 | TERF1	ENST00000564832	ENSE00002604837	73859385	73862680	0.742488559	0.332665498	0.679588088
rs2295655	14	101539131	SNORD114-31 | LOC100130	ENST00000554016	ENSE00002513162	101538696	101539274	0.388879005	0.398878327	0.68046482
rs10499225	6	141769324	CITED2 | LOC729076	ENST00000565399	ENSE00002622426	141768148	141772143	0.3943087	0.39905934	0.68046482
kgp24942332	1	17523367	LOC400743 | PADI1	ENST00000539219	ENSE00002261175	17520556	17524112	-0.352227001	0.399137679	0.68046482
kgp6754133	12	44116330	ADAMTS20 | PUS7L	ENST00000553202	ENSE00002345647	44112796	44117803	0.176008597	0.399721435	0.68046482
kgp3549466	11	78135519	GAB2 | NARS2	ENST00000513207	ENSE00002083399	78135028	78136241	0.185246207	0.400360261	0.68046482
kgp17157260	6	134749978	SGK1 | LOC645175	ENST00000417483	ENSE00001603855	134749378	134750353	0.32163572	0.401021462	0.68046482
kgp3649817	14	50408906	ARF6 | C14orf182	ENST00000556913	ENSE00002499815	50408654	50410610	0.205048526	0.401048487	0.68046482
kgp21617381	10	6779561	LOC439949 | SFMBT2	ENST00000417112	ENSE00001657227	6779344	6779590	0.237945492	0.401730404	0.68046482
kgp7280769	16	50674103	NKD1 | SNX20	ENST00000565077	ENSE00002621561	50671504	50674771	0.333077882	0.402643919	0.68046482
kgp6238318	2	121076629	RALB | INHBB	ENST00000437837	ENSE00001710373	121076583	121076714	-0.532456677	0.403427918	0.68046482
kgp28388391	15	80557812	FAH | ARNT2	ENST00000558913	ENSE00002560671	80555410	80558035	0.298621384	0.403456095	0.68046482
kgp28266264	14	70936789	ADAM21 | ADAM20	ENST00000556964	ENSE00002518821	70935598	70938309	0.214193322	0.404334346	0.68046482
rs6437415	3	194500357	FAM43A | C3orf21	ENST00000455796	ENSE00001647637	194500191	194500387	-0.419582747	0.404724228	0.68046482
rs310025	16	81422980	GAN | CMIP	ENST00000568107	ENSE00002626991	81416874	81424489	0.180826181	0.405108378	0.68046482
kgp11918076	3	177614971	TBL1XR1 | KCNMB2	ENST00000436078	ENSE00001606478	177614352	177617012	-0.388425521	0.40633354	0.68046482
kgp7308433	12	130734520	FZD10 | PIWIL1	ENST00000563922	ENSE00002623704	130734224	130734563	0.308050606	0.407855419	0.68046482
kgp766657	11	58902412	FAM111B | FAM111A	ENST00000501817	ENSE00001976137	58901697	58903061	-0.216959003	0.408463542	0.68046482
kgp8364192	8	130253443	PVT1 | LOC100287906	ENST00000509893	ENSE00002072925	130253423	130253496	0.557075592	0.409122038	0.68046482
kgp7939945	6	110149960	FIG4 | GPR6	ENST00000458693	ENSE00001647751	110149699	110149988	0.247509471	0.409592124	0.68046482
rs617445	11	111649040	PPP2R1B | ALG9	ENST00000534218	ENSE00002168229	111648900	111649074	0.224137417	0.411352303	0.68046482
kgp7611563	15	95026435	MCTP2 | LOC440311	ENST00000565106	ENSE00002296294	95024040	95027181	-0.175182889	0.411964438	0.68046482
kgp5980540	12	44116028	ADAMTS20 | PUS7L	ENST00000553202	ENSE00002345647	44112796	44117803	0.212174535	0.413554178	0.68046482
kgp7194671	5	43015214	LOC100288522 | LOC64898	ENST00000503152	ENSE00002079428	43014838	43015252	0.344591104	0.413958659	0.68046482
rs4972969	2	231556663	SP100 | CAB39	ENST00000415174	ENSE00001768231	231555636	231556948	0.541313205	0.414311162	0.68046482
kgp6337748	9	100506937	XPA | FOXE1	ENST00000562653	ENSE00002577170	100505490	100507217	0.176249023	0.414531897	0.68046482
rs3814018	1	95086778	F3 | SLC44A3	ENST00000435559	ENSE00001601116	95086707	95087512	-0.349568723	0.415456227	0.68046482
rs3003470	1	17523263	LOC400743 | PADI1	ENST00000539219	ENSE00002261175	17520556	17524112	-0.394341241	0.41546392	0.68046482
rs8062805	16	81422951	GAN | CMIP	ENST00000568107	ENSE00002626991	81416874	81424489	-0.177003693	0.415844861	0.68046482
kgp18561815	9	37088703	LOC100287249 | ZCCHC7	ENST00000429493	ENSE00001770690	37086665	37090398	0.17335123	0.415909418	0.68046482
kgp4507086	10	43247120	LOC728064 | LOC283028	ENST00000568976	ENSE00002596639	43246626	43247593	0.312864083	0.415977926	0.68046482
kgp9594162	22	42673003	LOC388906 | NFAM1	ENST00000420096	ENSE00001679176	42672702	42673057	0.173660227	0.416125601	0.68046482
rs607407	1	234769499	IRF2BP2 | PP2672	ENST00000429269	ENSE00001771955	234768694	234770526	0.377954085	0.416760383	0.68046482
rs2935941	1	17523194	LOC400743 | PADI1	ENST00000539219	ENSE00002261175	17520556	17524112	-0.544697564	0.417432828	0.68046482
kgp21034429	4	11771963	HS3ST1 | HSP90AB2P	ENST00000515286	ENSE00002073273	11771933	11772099	-0.395521523	0.417992771	0.68046482
kgp950341	8	52935344	LOC100287313 | ST18	ENST00000521188	ENSE00002103365	52934976	52935534	0.19831496	0.418788858	0.68046482
kgp18212320	3	156800300	LEKR1 | CCNL1	ENST00000471357	ENSE00001879700	156799456	156801064	0.412939409	0.418860535	0.68046482
kgp10384923	16	49501794	C16orf78 | ZNF423	ENST00000561523	ENSE00002623579	49500017	49502392	0.210965216	0.419649077	0.68046482
kgp4981719	12	3151024	TEAD4 | TSPAN9	ENST00000513358	ENSE00002302855	3150603	3152614	-0.5385393	0.419765252	0.68046482
kgp5589760	2	217083754	XRCC5 | MARCH4	ENST00000562038	ENSE00002618694	217081768	217084915	0.176256729	0.419792039	0.68046482
kgp11665069	15	25279909	PAR5 | SNORD109A	ENST00000552334	ENSE00002349489	25277020	25281637	-0.19736194	0.420182201	0.68046482
kgp3039572	21	41099821	B3GALT5 | IGSF5	ENST00000457325	ENSE00001726936	41099682	41099919	-0.299326551	0.420777928	0.68046482
rs16951299	16	79791603	MAF | DYNLRB2	ENST00000568389	ENSE00002607025	79791548	79791612	0.192091015	0.420845681	0.68046482
kgp19771779	15	44827080	CTDSPL2 | LOC645212	ENST00000313807	ENSE00001427514	44825691	44827672	-0.179735525	0.420928758	0.68046482
kgp19439306	14	50793314	ATP5S | CDKL1	ENST00000555403	ENSE00002482016	50793244	50793491	-0.190066261	0.421132743	0.68046482
rs2524148	6	31262770	HLA-C | HLA-B	ENST00000539514	ENSE00002312291	31262721	31262790	0.313845853	0.422431228	0.68165039
rs17137253	6	3910784	LOC100289591 | PRPF4B	ENST00000566733	ENSE00002617674	3905144	3912213	0.520277626	0.400607977	0.682854506
rs1492753	4	125421325	LOC100289258 | ANKRD50	ENST00000563724	ENSE00002600455	125421097	125422149	0.679346453	0.336586725	0.684339835
rs12995928	2	201689742	BZW1 | CLK1	ENST00000568571	ENSE00002589934	201689396	201692061	0.359968548	0.425319505	0.68450081
kgp5448838	15	95024363	MCTP2 | LOC440311	ENST00000565106	ENSE00002296294	95024040	95027181	-0.242699759	0.425358948	0.68450081
rs1440221	4	47845920	CORIN | NFXL1	ENST00000563286	ENSE00002580194	47842139	47846356	-0.202617751	0.425899013	0.68450081
rs6959306	7	73402458	WBSCR28 | ELN	ENST00000567919	ENSE00002628099	73400322	73403097	0.589925204	0.407294815	0.68557191
kgp22817976	11	68640586	CPT1A | MRPL21	ENST00000512200	ENSE00002055373	68639011	68642010	0.545259955	0.408896467	0.68557191
kgp306018	10	36378240	FZD8 | ANKRD30A	ENST00000412439	ENSE00001680347	36378207	36378317	-0.691804177	0.411462467	0.68557191
kgp7405854	8	79750756	IL7 | STMN2	ENST00000565297	ENSE00002583800	79749764	79752757	-0.578131462	0.414258933	0.68557191
kgp2883781	2	166689636	GALNT3 | TTC21B	ENST00000457108	ENSE00001618535	166689558	166689725	-0.509643387	0.415096523	0.68557191
kgp28953981	8	137827619	KHDRBS3 | FLJ45872	ENST00000520068	ENSE00002130161	137827374	137827931	-0.865035618	0.415938227	0.68557191
rs12432178	14	77534069	C14orf4 | KIAA1737	ENST00000500215	ENSE00001980448	77533452	77535846	-0.502277625	0.418198865	0.68557191
rs10847284	12	127631071	LOC121296 | LOC10028841	ENST00000541419	ENSE00002259712	127630881	127631077	-0.533286981	0.420639602	0.685825437
kgp842752	3	177615133	TBL1XR1 | KCNMB2	ENST00000436078	ENSE00001606478	177614352	177617012	-0.671678916	0.4280584	0.687056501
rs930076	5	9550642	SNORD123 | TAS2R1	ENST00000508179	ENSE00002021903	9549414	9550721	0.470756378	0.421156721	0.688028307
rs914219	21	46414888	C21orf70 | NCRNA00162	ENST00000569966	ENSE00002621024	46414277	46415100	0.445517928	0.430442887	0.689768595
rs717993	16	80632970	DYNLRB2 | CDYL2	ENST00000570137	ENSE00002617613	80631803	80636416	0.267465578	0.43089107	0.689768595
kgp18909752	12	111381139	LOC100131138 | CUX2	ENST00000548368	ENSE00002354416	111381128	111381499	0.358436684	0.424469346	0.689923268
rs10500471	16	63092934	CDH8 | CDH11	ENST00000568741	ENSE00002616828	63091122	63093812	0.386448068	0.427259305	0.689923268
kgp18046090	3	194014438	HES1 | LOC100131551	ENST00000456816	ENSE00001766474	194014254	194014636	-0.404245565	0.428588697	0.689923268
kgp1075922	1	29197206	OPRD1 | EPB41	ENST00000443593	ENSE00001675297	29197025	29197779	-0.372304787	0.431109594	0.690612456
rs10141627	14	101538613	SNORD114-31 | LOC100130	ENST00000444846	ENSE00001687158	101537124	101539271	0.321042675	0.432562716	0.690792406
kgp10737811	16	81424024	GAN | CMIP	ENST00000568107	ENSE00002626991	81416874	81424489	-0.205190421	0.432675277	0.690792406
kgp14624616	2	187220154	FLJ44048 | ZC3H15	ENST00000564407	ENSE00002576512	187219299	187221500	-0.263187044	0.43347782	0.691159483
kgp17220738	6	26687991	ZNF322A | GUSBL1	ENST00000562904	ENSE00002603318	26686469	26688192	0.354389232	0.434499725	0.691874891
kgp8842607	16	89980158	TCF25 | MC1R	ENST00000539976	ENSE00002202946	89979640	89981576	-0.369713902	0.436069987	0.69346044
kgp2017849	20	48895479	CEBPB | PTPN1	ENST00000425497	ENSE00001595587	48894714	48896332	-0.702842809	0.437374184	0.694619264
kgp11809220	3	151525976	LOC201651 | AADAC	ENST00000485020	ENSE00001820869	151525932	151526037	-0.56001707	0.428518373	0.694894658
kgp4197929	16	68607749	ZFP90 | CDH3	ENST00000569654	ENSE00002581868	68607685	68608126	-0.21845448	0.438274277	0.695134102
kgp18459769	9	92783799	LOC100129066 | LOC34051	ENST00000426593	ENSE00001656859	92782936	92783827	0.490699666	0.439054824	0.695458232
kgp16866690	13	51095386	FAM10A4 | DLEU7	ENST00000428276	ENSE00001601718	51095069	51095572	0.559202824	0.343968476	0.696049417
kgp3201195	5	117881816	LOC100287135 | DTWD2	ENST00000515704	ENSE00002051714	117881553	117881844	-0.548399807	0.431688966	0.696272526
kgp19572413	14	39954452	FBXO33 | LRFN5	ENST00000556620	ENSE00002506169	39954221	39954461	0.361320144	0.438173817	0.697279422
kgp7017943	14	71076645	MED6 | TTC9	ENST00000500016	ENSE00001980392	71075515	71078067	-0.398180474	0.439497333	0.697279422
rs2977256	1	17523137	LOC400743 | PADI1	ENST00000539219	ENSE00002261175	17520556	17524112	-0.376609722	0.441656299	0.697753413
kgp938085	2	47006413	SOCS5 | LOC388948	ENST00000568862	ENSE00002622745	47004922	47007384	0.185993618	0.442012537	0.697753413
kgp12046794	14	70937424	ADAM21 | ADAM20	ENST00000556964	ENSE00002518821	70935598	70938309	-0.288999199	0.442238079	0.697753413

kgp19844705	15	95024079	MCTP2 | LOC440311	ENST00000565106	ENSE00002296294	95024040	95027181	-0.157908571	0.443726192	0.698130263
rs4973343	2	231556188	SP100 | CAB39	ENST00000415174	ENSE00001768231	231555636	231556948	0.460056855	0.444037952	0.698130263
kgp30623521		46185411	LOC392452 | ZNF673	ENST00000446884	ENSE00001797372	46185359	46185889	-0.4700782	0.444642517	0.698130263
kgp4300418	12	4135584	PARP11 | CCND2	ENST00000543206	ENSE00002239925	4135388	4135824	-0.257839042	0.445424465	0.698130263
kgp4112258	8	128084965	FAM84B | POU5F1B	ENST00000523510	ENSE00002108688	128084939	128085163	-0.196622125	0.446281393	0.698130263
kgp19888289	15	72114745	NR2E3 | MYO9A	ENST00000563041	ENSE00002601016	72114376	72114796	0.210703226	0.446511876	0.698130263
kgp12843812	11	58902679	FAM111B | FAM111A	ENST00000501817	ENSE00001976137	58901697	58903061	0.166926377	0.44659732	0.698130263
kgp23602270	7	148605	LOC100132858 | LOC10028	ENST00000497320	ENSE00001836600	148328	148618	-0.248592319	0.447373907	0.698130263
kgp9522742	7	6694776	ZNF316 | LOC100133111	ENST00000564837	ENSE00002606444	6694495	6696063	-0.498563526	0.448392744	0.698130263
rs2454181	1	17522940	LOC400743 | PADI1	ENST00000539219	ENSE00002261175	17520556	17524112	-0.361931427	0.448503811	0.698130263
kgp3587176	7	156737504	LMBR1 | NOM1	ENST00000427073	ENSE00001697959	156737415	156737665	-0.302235235	0.450185207	0.698130263
kgp27356505	9	37087539	LOC100287249 | ZCCHC7	ENST00000429493	ENSE00001770690	37086665	37090398	0.230628976	0.450609948	0.698130263
rs2464877	7	6694718	ZNF316 | LOC100133111	ENST00000564837	ENSE00002606444	6694495	6696063	-0.577413301	0.451324972	0.698130263
kgp6177002	15	40664583	DISP2 | C15orf23	ENST00000561261	ENSE00002575109	40664575	40664677	-0.388649726	0.451717806	0.698130263
kgp343748	1	56297981	USP24 | LOC100288320	ENST00000424357	ENSE00001708423	56297937	56298098	-0.373575017	0.452029482	0.698130263
kgp6446390	11	65211227	NCRNA00084 | MALAT1	ENST00000501122	ENSE00001961965	65190269	65213011	-0.273480913	0.452153009	0.698130263
kgp20594119	8	54429314	OPRK1 | ATP6V1H	ENST00000426023	ENSE00002169965	54427731	54429514	-0.188460036	0.452887643	0.698130263
kgp11196746	1	247351259	ZNF124 | LOC729806	ENST00000566446	ENSE00002602464	247350583	247351828	0.163011044	0.452888149	0.698130263
rs7324465	13	45238038	LOC100287772 | NUFIP1	ENST00000426509	ENSE00001757959	45238031	45238171	0.611095435	0.34675211	0.698388052
kgp5117715	10	54733328	MBL2 | PCDH15	ENST00000444155	ENSE00001799433	54733248	54733450	0.155809881	0.454168914	0.69921158
kgp1813353	12	58287630	LOC100289282 | LOC10028	ENST00000548955	ENSE00002418778	58287523	58287735	0.484530478	0.437195852	0.701383719
kgp20507175	8	135224135	LOC100129104 | ZFAT	ENST00000523317	ENSE00002137494	135224108	135224449	-0.704715882	0.351824672	0.702255992
rs6045326	20	18304525	ZNF133 | MGC44328	ENST00000457009	ENSE00001749592	18304288	18305878	-0.70682581	0.353193607	0.702255992
kgp25147348	1	168382072	TBX19 | XCL2	ENST00000441851	ENSE00001627105	168382031	168382174	0.546691027	0.353583436	0.702255992
rs2236537	1	182058955	ZNF648 | LOC100130996	ENST00000428646	ENSE00001688250	182058500	182059247	0.347318149	0.445198614	0.70294518
kgp7339167	2	118597434	LOC100287740 | CCDC93	ENST00000420330	ENSE00001639718	118596758	118599234	-0.307118148	0.457281814	0.703107197
kgp20510875	8	79750704	IL7 | STMN2	ENST00000565297	ENSE00002583800	79749764	79752757	-0.515929566	0.440862375	0.703503789
kgp21775799	10	69993241	ATOH7 | PBLD	ENST00000444086	ENSE00001694139	69993008	69993330	-0.545277251	0.458180073	0.703592045
kgp13155602	21	35336062	FLJ46020 | MRPS6	ENST00000381181	ENSE00001487739	35334367	35336260	0.401649898	0.452552958	0.704645471
kgp23726948	7	80222	LOC100132858 | LOC10028	ENST00000478759	ENSE00001902048	80130	80418	-0.546828736	0.456224425	0.704645471
rs2015398	15	30297737	LOC727808 | LOC10012843	ENST00000561392	ENSE00002564316	30297646	30297992	-0.585036242	0.457311247	0.704645471
kgp6635361	1	56714590	USP24 | LOC100288320	ENST00000569425	ENSE00002618882	56713966	56714872	-0.388277112	0.457409339	0.704645471
kgp13091143	21	40249313	ETS2 | FLJ45139	ENST00000544859	ENSE00002214012	40249215	40252276	0.315665648	0.458205145	0.704645471
kgp6923718	2	46619601	EPAS1 | LOC388946	ENST00000418415	ENSE00001603980	46619436	46619622	-0.418487989	0.459087201	0.704645471
kgp28211892	14	62031540	PRKCH | HIF1A	ENST00000508827	ENSE00002070975	62027461	62031959	-0.511237641	0.44489612	0.706184318
rs959932	8	9060044	PPP1R3B | TNKS	ENST00000523747	ENSE00002113722	9060009	9060347	-0.26212617	0.460563808	0.706353895
kgp21963658	10	47099588	PPYR1 | LOC728643	ENST00000422732	ENSE00001763272	47099588	47099716	0.226793652	0.461500535	0.706457272
kgp483346	10	131865321	EBF3 | LOC387723	ENST00000456581	ENSE00001788710	131864638	131866872	-0.172938159	0.462615552	0.706457272
kgp26483832	6	26686666	ZNF322A | GUSBL1	ENST00000562904	ENSE00002603318	26686469	26688192	-0.189174385	0.462933624	0.706457272
kgp23728108	7	6695293	ZNF316 | LOC100133111	ENST00000564837	ENSE00002606444	6694495	6696063	-0.548183199	0.462972413	0.706457272
kgp7073327	8	130691533	LOC100287906 | GSDMC	ENST00000522667	ENSE00002128400	130691381	130691566	-0.59194867	0.358149643	0.706577484
rs2270973	1	48520777	LOC388630 | SKINTL	ENST00000456803	ENSE00001733807	48520742	48520868	0.541321621	0.35905336	0.706577484
kgp1954720	2	105321806	LOC150568 | LOC10028707	ENST00000453322	ENSE00001732703	105321309	105321967	0.577321639	0.464388164	0.707722872
kgp22411335	5	127302070	LOC728586 | FLJ33630	ENST00000501652	ENSE00001970319	127301580	127302620	0.146595388	0.466103003	0.708653442
kgp13806428	7	46040165	LOC100287349 | TNS3	ENST00000436056	ENSE00001608996	46040139	46040497	-0.217266957	0.466173018	0.708653442
rs3088220	20	48894756	CEBPB | PTPN1	ENST00000445003	ENSE00001632288	48894714	48894964	-0.234774384	0.467078345	0.709136556
kgp9888546	12	3151949	TEAD4 | TSPAN9	ENST00000513358	ENSE00002302855	3150603	3152614	-0.606902868	0.46800907	0.709653666
kgp28400602	15	25279335	PAR5 | SNORD109A	ENST00000552334	ENSE00002349489	25277020	25281637	-0.212627033	0.46859484	0.709653666
kgp4730824	6	81177838	BCKDHB | FAM46A	ENST00000569267	ENSE00002590559	81176675	81178797	-0.223523098	0.469679026	0.71040424
rs2294106	4	4323754	ZNF509 | D4S234E	ENST00000509015	ENSE00002051780	4323689	4324272	-1.200488598	0.363060624	0.711200949
kgp14542849	2	207731643	FASTKD2 | CPO	ENST00000438070	ENSE00001761767	207731519	207731701	-0.330127734	0.470800364	0.71120906
kgp21797895	10	3026634	LOC727878 | PFKP	ENST00000446337	ENSE00001649984	3026556	3026736	-0.25367024	0.471455664	0.711308733
kgp4085468	11	130736831	C11orf44 | SNX19	ENST00000525716	ENSE00002194156	130735365	130737889	0.148741354	0.473137258	0.712523194
kgp11903749	16	34213369	TOP | MGC34800	ENST00000568121	ENSE00002592667	34213115	34213562	-0.155503814	0.473815015	0.712523194
kgp17848130	3	128579199	ERV3 | LOC653712	ENST00000567253	ENSE00002589601	128578559	128579369	0.177791875	0.474031586	0.712523194
rs17781620	8	135804596	ZFAT | LOC286094	ENST00000568248	ENSE00002623781	135804263	135810515	-0.185876178	0.475627549	0.7140329
kgp3975923	16	89113394	CBFA2T3 | ACSF3	ENST00000537498	ENSE00002244645	89112580	89114228	0.214236536	0.476353859	0.714234916
kgp4935524	20	31445335	MAPRE1 | EFCAB8	ENST00000569087	ENSE00002597460	31444848	31446557	-0.410862778	0.477214789	0.714638027
kgp11700460	1	92865513	RPAP2 | GFI1	ENST00000564442	ENSE00002592577	92864531	92867613	-0.41793943	0.468555688	0.715848967
kgp16029593	18	77806932	C18orf22 | ADNP2	ENST00000562391	ENSE00002576217	77806900	77807425	-0.280731934	0.478830347	0.71616881
kgp11984745	2	1625064	TPO | PXDN	ENST00000366424	ENSE00001647238	1624282	1625751	0.541474448	0.36851699	0.716798006
rs731284	4	25871101	KIAA0746 | LOC389203	ENST00000503085	ENSE00002057179	25870937	25871172	0.654681244	0.369259579	0.716798006
rs11249215	1	25297184	RUNX3 | SYF2	ENST00000568143	ENSE00002580573	25294914	25297356	-0.569662447	0.371074592	0.717076576
kgp794834	5	72584546	TMEM174 | FOXD1	ENST00000515556	ENSE00002058064	72584394	72584666	0.503989232	0.456902588	0.719529009
kgp11575712	8	129418091	PVT1 | LOC100287906	ENST00000520206	ENSE00002117776	129417515	129418879	0.557998625	0.458100136	0.719529009
kgp7113129	13	48505286	HTR2A | SUCLA2	ENST00000566385	ENSE00002595049	48504290	48506757	0.195666219	0.481782926	0.719693059
kgp2883184	17	80252707	CSNK1D | CD7	ENST00000566986	ENSE00002591651	80251592	80252786	-0.187723298	0.482921983	0.720502885
kgp4138732	3	27901738	EOMES | CMC1	ENST00000425195	ENSE00001781688	27901578	27901816	0.568394555	0.375699947	0.72275909
kgp10693243	7	17490654	AHR | SNX13	ENST00000419463	ENSE00001618421	17490619	17490685	-0.481814058	0.477658545	0.723118068
kgp12428200	6	109090119	FOXO3 | ARMC2	ENST00000448744	ENSE00001698309	109089594	109090773	-0.343480395	0.478125026	0.723118068
kgp11593949	1	98432735	DPYD | FLJ35409	ENST00000561881	ENSE00002583625	98432561	98434370	0.376615138	0.480359845	0.723118068
kgp10791401	15	98564516	ARRDC4 | FAM169B	ENST00000554798	ENSE00002533240	98564417	98565007	0.366865516	0.482078712	0.723118068
rs8033826	15	101368511	LOC440313 | LOC145757	ENST00000559755	ENSE00002543926	101368331	101368702	-1.143740691	0.377791424	0.723538039
kgp5469448	4	8357923	HTRA3 | ACOX3	ENST00000505448	ENSE00002069127	8357038	8359103	-0.159078495	0.487927152	0.727071695
kgp12122477	2	896089	LOC100128185 | LOC39134	ENST00000445279	ENSE00001727388	895902	896138	-0.546143375	0.46532798	0.727074968
kgp27718128	12	3152971	TEAD4 | TSPAN9	ENST00000513358	ENSE00002234190	3152644	3154116	0.24253723	0.489629901	0.727099173
kgp8420392	7	6695994	ZNF316 | LOC100133111	ENST00000564837	ENSE00002606444	6694495	6696063	-0.161781156	0.489671618	0.727099173
kgp25486034	18	77806938	C18orf22 | ADNP2	ENST00000562391	ENSE00002576217	77806900	77807425	-0.236936818	0.490317259	0.727099173
kgp6863112	11	75922211	WNT11 | PRKRIR	ENST00000531538	ENSE00002157470	75922000	75922293	-0.393080959	0.4903552	0.727099173
kgp14544614	2	201690515	BZW1 | CLK1	ENST00000568571	ENSE00002589934	201689396	201692061	0.193177213	0.492626617	0.729247484
kgp19546234	14	45233273	FSCB | C14orf28	ENST00000556405	ENSE00002460394	45232360	45234829	-0.551172989	0.493012383	0.729247484
kgp28960254	8	137743607	KHDRBS3 | FLJ45872	ENST00000520060	ENSE00002138718	137743562	137743862	-0.97570356	0.382567206	0.72942814
kgp284828	12	3153157	TEAD4 | TSPAN9	ENST00000513358	ENSE00002234190	3152644	3154116	-0.474939383	0.494756827	0.730143699
kgp5683896	4	8357687	HTRA3 | ACOX3	ENST00000505448	ENSE00002069127	8357038	8359103	-0.152015176	0.494885865	0.730143699
kgp19868050	15	32907145	LOC728080 | ARHGAP11A	ENST00000500941	ENSE00001975629	32906941	32907312	0.16009951	0.496977389	0.730143699
kgp20786650	4	157704	ZNF718 | ZNF732	ENST00000510175	ENSE00002048735	154702	157779	0.233665919	0.497315784	0.730143699

kgp16329115	16	25111991	LOC554206 | LCMT1	ENST00000563176	ENSE00002620737	25111885	25112006	-0.311894762	0.497554772	0.730143699
kgp6741788	12	3153343	TEAD4 | TSPAN9	ENST00000513358	ENSE00002234190	3152644	3154116	-0.387323117	0.497661368	0.730143699
kgp22722214	5	9550529	SNORD123 | TAS2R1	ENST00000508179	ENSE00002021903	9549414	9550721	0.302306064	0.498515781	0.730143699
rs4682534	3	114034098	TIGIT | ZBTB20	ENST00000570269	ENSE00002576360	114033348	114035026	-0.152674718	0.498825602	0.730143699
kgp30141015	5	139486429	NRG2 | PURA	ENST00000499203	ENSE00001984051	139485881	139487228	-0.166812478	0.499062595	0.730143699
kgp4846379	8	83777734	SNX16 | RALYL	ENST00000522123	ENSE00002129829	83777702	83777885	-0.618036839	0.385417474	0.730768203
kgp18815692	12	126451214	TMEM132B | LOC10028870	ENST00000545784	ENSE00002228181	126450746	126451888	0.763775824	0.387731941	0.730768203
kgp29047966	4	129490213	PGRMC2 | PHF17	ENST00000514265	ENSE00002048854	129489127	129490442	0.513537905	0.389198517	0.730768203
kgp3427530	3	120555165	GTF2E1 | STXBP5L	ENST00000490647	ENSE00001923330	120555116	120555207	0.507231813	0.390083726	0.730768203
rs2174893	18	74403875	FLJ44881 | ZNF236	ENST00000415242	ENSE00001659524	74401986	74403929	0.537045385	0.392340746	0.73077737
rs2291204	11	38676579	LOC100129825 | LOC10028	ENST00000534756	ENSE00002166839	38676510	38676802	0.499610114	0.393495507	0.73077737
kgp5670917	2	166657989	GALNT3 | TTC21B	ENST00000425688	ENSE00001735410	166657691	166658092	-0.293431874	0.500397482	0.731210365
kgp22496473	5	106151104	RAB9P1 | LOC100287833	ENST00000513273	ENSE00002030686	106150898	106151247	-0.456415337	0.501659179	0.732096237
kgp12445572	15	77337455	PSTPIP1 | TSPAN3	ENST00000560446	ENSE00002562292	77337397	77337502	-0.20977752	0.502216805	0.732096237
kgp8194184	12	3428691	LOC100128253 | PRMT8	ENST00000543036	ENSE00002256323	3428607	3428892	0.299059929	0.503048833	0.732293671
kgp9838011	15	89340726	ISG20 | ACAN	ENST00000561358	ENSE00002559856	89340644	89341135	-0.466972845	0.503565656	0.732293671
kgp17829394	3	30568556	RBMS3 | TGFBR2	ENST00000450746	ENSE00001781896	30568324	30568677	0.536902043	0.396713332	0.73357767
GA014049	17	17094919	MPRIP | PLD6	ENST00000567268	ENSE00002611064	17091982	17095962	-0.153854962	0.505078726	0.733610135
kgp24919298	1	166304630	LOC284685 | FMO9P	ENST00000425271	ENSE00001641265	166304565	166304702	-0.387060695	0.496801316	0.734541
rs2332431	14	71075851	MED6 | TTC9	ENST00000500016	ENSE00001980392	71075515	71078067	-0.524454281	0.498282828	0.734541
rs12708878	16	66786367	DYNC1LI2 | CCDC79	ENST00000501143	ENSE00001985625	66785655	66786803	-0.327105884	0.501313108	0.734541
rs1986346	8	8426753	PRAGMIN | CLDN23	ENST00000522661	ENSE00002101795	8426697	8427198	0.302958878	0.502480289	0.734541
rs921389	5	4515851	IRX1 | LOC340094	ENST00000503188	ENSE00002026322	4515436	4516889	-0.319389825	0.50610131	0.734541
kgp373969	14	101430324	SNORD114-7 | SNORD114-8	ENST00000554693	ENSE00001626199	101430302	101430337	0.476000461	0.509208905	0.734541
kgp19122858	12	3150874	TEAD4 | TSPAN9	ENST00000513358	ENSE00002302855	3150603	3152614	-0.504892944	0.511448661	0.734541
kgp11767148	12	3151567	TEAD4 | TSPAN9	ENST00000513358	ENSE00002302855	3150603	3152614	-0.633623745	0.512233413	0.734541
kgp13148197	21	41101808	B3GALT5 | IGSF5	ENST00000457325	ENSE00001686404	41101431	41102607	-0.465310065	0.512660368	0.734541
kgp8649578	11	107182950	GUCY1A2 | CWF19L2	ENST00000561746	ENSE00002621788	107182858	107186997	0.475135636	0.512661218	0.734541
kgp12228940	22	32672792	SLC5A4 | RFPL3	ENST00000452181	ENSE00001763735	32672672	32673173	-0.43788671	0.5141787	0.734541
rs579501	10	43246795	LOC728064 | LOC283028	ENST00000568976	ENSE00002596639	43246626	43247593	0.154634542	0.509640431	0.738980408
kgp22833416		46185835	LOC392452 | ZNF673	ENST00000446884	ENSE00001797372	46185359	46185889	0.188357562	0.510513071	0.738980408
kgp9729567	18	77807211	C18orf22 | ADNP2	ENST00000562391	ENSE00002576217	77806900	77807425	-0.238976966	0.510612809	0.738980408
kgp3534819	21	40378632	FLJ45139 | LOC100289305	ENST00000419664	ENSE00001795712	40378574	40378710	0.200843782	0.511277455	0.739056154
kgp5185837	6	168662879	FRMD1 | DACT2	ENST00000446811	ENSE00001679224	168662592	168663261	0.82649316	0.401444139	0.739139638
rs9402608	6	134786163	SGK1 | LOC645175	ENST00000440090	ENSE00001693619	134786115	134786196	0.86993667	0.404550159	0.741675291
rs7034845	9	120413192	ASTN2 | TLR4	ENST00000450938	ENSE00001614425	120413130	120413255	-0.296987823	0.515420933	0.743690835
kgp2418376	16	80635560	DYNLRB2 | CDYL2	ENST00000570137	ENSE00002617613	80631803	80636416	-0.280197139	0.515937234	0.743690835
kgp24027849	2	9247081	MBOAT2 | ASAP2	ENST00000565044	ENSE00002602192	9246722	9249994	-0.281797612	0.516332162	0.743690835
kgp7563214	2	16385176	MYCN | FAM49A	ENST00000444804	ENSE00001692237	16384969	16385494	0.186460677	0.51759536	0.744621692
kgp3490181	14	54316625	DDHD1 | BMP4	ENST00000418927	ENSE00001646248	54316440	54316688	-0.574304521	0.48099854	0.747666124
kgp9143094	2	2840716	MYT1L | LOC729897	ENST00000452701	ENSE00001717958	2840691	2840778	-0.500794516	0.485148495	0.748055701
rs10735019	12	3405864	TSPAN9 | LOC100128253	ENST00000505276	ENSE00002256861	3405368	3407426	0.48639704	0.486236205	0.748055701
rs4842007	9	138474785	PAEP | GLT6D1	ENST00000447907	ENSE00001628101	138474782	138474855	0.430055344	0.489428353	0.74912503
kgp19646416	14	45234499	FSCB | C14orf28	ENST00000556405	ENSE00002460394	45232360	45234829	-0.544353597	0.521460292	0.749238738
rs3732812	3	112863027	C3orf17 | BOC	ENST00000490139	ENSE00001823459	112861197	112863182	-0.341237057	0.522046213	0.749238738
kgp25670024	16	81423393	GAN | CMIP	ENST00000568107	ENSE00002626991	81416874	81424489	0.145032076	0.524970397	0.749834722
kgp24050627	2	34902889	MYADML | LOC100288911	ENST00000423663	ENSE00001665890	34902646	34902994	-0.187061269	0.525761897	0.749834722
rs7178173	15	26640219	LOC100128714 | GABRB3	ENST00000451579	ENSE00001718076	26640219	26640391	0.296716105	0.526104045	0.749834722
kgp10253331	1	92867450	RPAP2 | GFI1	ENST00000564442	ENSE00002592577	92864531	92867613	-0.392000853	0.526823877	0.749834722
kgp21446122	19	28248413	LOC100101266 | LOC14818	ENST00000562493	ENSE00002598618	28248092	28251757	-0.136467606	0.527251442	0.749834722
kgp6562889	12	3153712	TEAD4 | TSPAN9	ENST00000513358	ENSE00002234190	3152644	3154116	-0.345390909	0.527682558	0.749834722
kgp10065154	16	72460467	PMFBP1 | ZFHX3	ENST00000564508	ENSE00002602918	72459847	72463072	0.215692527	0.527782534	0.749834722
GA014048	17	17092263	MPRIP | PLD6	ENST00000567268	ENSE00002611064	17091982	17095962	-0.197980889	0.527876993	0.749834722
kgp11003347	1	234664728	TARBP1 | IRF2BP2	ENST00000435574	ENSE00001674386	234663637	234666088	-0.219610812	0.528052621	0.749834722
kgp10212369	13	100232527	TM9SF2 | CLYBL	ENST00000437113	ENSE00001719585	100232423	100232622	0.272169675	0.530251791	0.75175113
kgp8217795	14	101538546	SNORD114-31 | LOC10013	ENST00000444846	ENSE00001687158	101537124	101539271	0.361054777	0.530781858	0.75175113
kgp8855952	11	38676665	LOC100129825 | LOC10028	ENST00000534756	ENSE00002166839	38676510	38676802	0.496996771	0.411932794	0.751996462
kgp2207553	8	79750764	IL7 | STMN2	ENST00000565297	ENSE00002583800	79749764	79752757	0.387826499	0.535281608	0.752080819
kgp2588812	12	9408262	PZP | LOC642846	ENST00000538219	ENSE00002299624	9408155	9408288	-0.513941967	0.538305263	0.752080819
kgp6211811	14	62027791	PRKCH | HIF1A	ENST00000508827	ENSE00002070975	62027461	62031959	-0.279939926	0.538798411	0.752080819
kgp8022242	10	45676724	LOC100133308 | OR13A1	ENST00000422807	ENSE00001735051	45676530	45676875	0.390829421	0.54013077	0.752080819
rs7749023	6	42061733	TAF8 | C6orf132	ENST00000562471	ENSE00002625332	42059976	42061997	-0.131397449	0.532968738	0.755926283
kgp348326	8	80683826	HEY1 | MRPS28	ENST00000502766	ENSE00002069117	80683802	80683857	-0.550158498	0.417412884	0.758771726
kgp17128721	6	109090008	FOXO3 | ARMC2	ENST00000448744	ENSE00001698309	109089594	109090773	-0.145497321	0.535978891	0.759303429
kgp3484740	3	9298564	SRGAP3 | LOC100288572	ENST00000449023	ENSE00001601324	9298443	9298684	-0.169121835	0.536751169	0.759506051
kgp10223430	12	54144274	CALCOCO1 | HOXC13	ENST00000570015	ENSE00002617645	54144231	54144749	0.510138471	0.420057323	0.759734294
kgp25190694	1	61125914	C1orf87 | NFIA	ENST00000423403	ENSE00001777761	61125303	61127142	0.497546358	0.421556302	0.759734294
kgp4374198	6	125995549	HDDC2 | HEY2	ENST00000423208	ENSE00001608620	125995499	125995621	0.494210143	0.425464442	0.759734294
kgp8252659	12	104565585	NFYB | TXNRD1	ENST00000547554	ENSE00002363303	104565551	104565814	-0.573196794	0.426391283	0.759734294
kgp27231123	9	19926539	SLC24A2 | MLLT3	ENST00000568063	ENSE00002587912	19926092	19929935	-0.873268184	0.426797121	0.759734294
kgp1464464	3	20432033	SGOL1 | VENTXP7	ENST00000566804	ENSE00002605809	20429741	20432054	-0.306931594	0.550026306	0.761092799
kgp11668663	19	51289007	GPR32 | ACPT	ENST00000563228	ENSE00002613480	51289000	51289080	0.269517104	0.551215694	0.761092799
kgp19145373	12	44116922	ADAMTS20 | PUS7L	ENST00000553202	ENSE00002345647	44112796	44117803	0.128916323	0.539432379	0.761922302
kgp14854593	2	75169151	HK2 | POLE4	ENST00000453951	ENSE00001643565	75169014	75169797	0.191052119	0.539721266	0.761922302
kgp22734248	9	37087230	LOC100287249 | ZCCHC7	ENST00000429493	ENSE00001770690	37086665	37090398	0.13033994	0.541642585	0.762157317
rs12594765	15	43034790	CDAN1 | TTBK2	ENST00000500850	ENSE00002588053	43034200	43035400	-0.183436568	0.542310369	0.762157317
kgp6463979	14	101424766	SNORD114-6 | SNORD114-7	ENST00000414488	ENSE00001602356	101424599	101424887	0.345138084	0.542560814	0.762157317
kgp5697409	10	116539401	ABLIM1 | FAM160B1	ENST00000436932	ENSE00001725826	116537710	116539662	-0.373080636	0.5426832	0.762157317
kgp7534664	16	80636220	DYNLRB2 | CDYL2	ENST00000570137	ENSE00002617613	80631803	80636416	-0.27409431	0.543887753	0.762157317
rs1041070	13	48505493	HTR2A | SUCLA2	ENST00000566385	ENSE00002595049	48504290	48506757	0.142875538	0.545623552	0.762157317
kgp7301641	17	17095198	MPRIP | PLD6	ENST00000567268	ENSE00002611064	17091982	17095962	-0.126614559	0.546307648	0.762157317
kgp16773831	13	114629983	FLJ44054 | RASA3	ENST00000562710	ENSE00002578109	114629487	114631817	0.195728365	0.546423974	0.762157317
kgp18480658	9	37088941	LOC100287249 | ZCCHC7	ENST00000429493	ENSE00001770690	37086665	37090398	0.146841893	0.546436812	0.762157317
kgp5218401	17	13680481	HS3ST3A1 | CDRT15P	ENST00000423323	ENSE00001689622	13679951	13680666	-0.151192454	0.54677818	0.762157317
kgp9659044	1	17523144	LOC400743 | PADI1	ENST00000539219	ENSE00002261175	17520556	17524112	-0.256210187	0.546970083	0.762157317
kgp1443025	21	18003265	C21orf34 | CXADR	ENST00000413645	ENSE00001665029	18003146	18003291	-0.235198952	0.547465115	0.762157317

rs1109400	20	61006405	C20orf151 | GATA5	ENST00000433121	ENSE00001685083	61006316	61006568	-0.197163891	0.548266888	0.762394163
rs685487	15	80136129	KIAA1024 | MTHFS	ENST00000567415	ENSE00002606026	80135889	80136646	-0.229336906	0.550061972	0.76278531
kgp15854040	1	17523136	LOC400743 | PADI1	ENST00000539219	ENSE00002261175	17520556	17524112	-0.290569346	0.550663259	0.76278531
kgp13181394	21	46411015	C21orf70 | NCRNA00162	ENST00000439088	ENSE00001802548	46409779	46411747	0.183864002	0.550696292	0.76278531
kgp11994252	17	27877857	TAOK1 | ABHD15	ENST00000562535	ENSE00002584590	27873905	27878921	0.130554043	0.55168597	0.76278531
kgp24784421	1	28975034	TAF12 | RNU11	ENST00000427804	ENSE00001796797	28974705	28975093	0.170486585	0.551973035	0.76278531
kgp20021044	15	95024854	MCTP2 | LOC440311	ENST00000565106	ENSE00002296294	95024040	95027181	0.11785966	0.553168682	0.76278531
kgp27227769	9	37088065	LOC100287249 | ZCCHC7	ENST00000429493	ENSE00001770690	37086665	37090398	-0.183843028	0.553518235	0.76278531
kgp974235	3	176352183	NAALADL2 | TBL1XR1	ENST00000434969	ENSE00001632374	176352133	176352323	0.28834423	0.553904604	0.76278531
kgp28375230	15	95024436	MCTP2 | LOC440311	ENST00000565106	ENSE00002296294	95024040	95027181	0.119820544	0.554521817	0.76278531
kgp3433875	1	121322275	LOC647121 | LOC10028939	ENST00000450546	ENSE00001787847	121322230	121322322	0.180652219	0.554867856	0.76278531
kgp5080707	6	3906139	LOC100289591 | PRPF4B	ENST00000566733	ENSE00002617674	3905144	3912213	0.250197423	0.556622927	0.763860951
kgp228780	1	98432917	DPYD | FLJ35409	ENST00000561881	ENSE00002583625	98432561	98434370	0.243539356	0.55691602	0.763860951
kgp292253	21	46410121	C21orf70 | NCRNA00162	ENST00000439088	ENSE00001802548	46409779	46411747	0.445783374	0.501845369	0.764231527
kgp20437924	8	1248775	LOC401442 | DLGAP2	ENST00000521186	ENSE00002090396	1248184	1250829	-0.566381223	0.432969146	0.765246612
kgp1818602	14	93372553	GOLGA5 | CHGA	ENST00000555299	ENSE00002497085	93372042	93373011	0.439787285	0.445381764	0.765246612
kgp9155537	17	69095332	LOC100133226 | LOC12468	ENST00000569074	ENSE00002589961	69093916	69095759	0.438863421	0.44749599	0.765246612
kgp10692126	9	93925941	LOC100128909 | AUH	ENST00000423719	ENSE00001710054	93925153	93927858	0.531684644	0.447916121	0.765246612
kgp11836631	11	43967090	LOC387763 | ACCSL	ENST00000501541	ENSE00001984255	43965337	43967226	-0.832530587	0.44883791	0.765246612
kgp5340797	18	4294533	LOC284215 | LOC642597	ENST00000565811	ENSE00002615565	4293160	4295405	0.435994643	0.450174576	0.765246612
kgp7420867	14	48235082	MDGA2 | RPS29	ENST00000555985	ENSE00002534172	48234157	48236960	-0.587423293	0.450421034	0.765246612
rs4853848	2	2899681	MYT1L | LOC729897	ENST00000457478	ENSE00001681664	2898820	2900452	-0.446789316	0.450561915	0.765246612
kgp7560779	4	185291020	LOC728175 | IRF2	ENST00000512674	ENSE00002061359	185289567	185291024	0.435708793	0.451263086	0.765246612
kgp9432107	5	159912418	PTTG1 | ATP10B	ENST00000517927	ENSE00002103277	159912306	159914433	-0.432895661	0.453076932	0.765246612
kgp5359576	3	172309051	TNFSF10 | AADACL1	ENST00000418839	ENSE00001731231	172308503	172311504	-0.439076177	0.454629874	0.765246612
kgp12261170	11	13901041	FAR1 | SPON1	ENST00000530492	ENSE00002181925	13900969	13901046	-0.750066453	0.460097252	0.765246612
kgp872375	1	246846313	C1orf71 | SCCPDH	ENST00000570141	ENSE00002627157	246845410	246848377	-0.429928795	0.464334275	0.765246612
kgp17229083	6	134825175	SGK1 | LOC645175	ENST00000431422	ENSE00001612330	134823926	134825719	0.450599246	0.467169895	0.765246612
kgp2270642	16	72462787	PMFBP1 | ZFHX3	ENST00000564508	ENSE00002602918	72459847	72463072	-0.418737963	0.469013355	0.765246612
kgp22549512	5	7151064	POLS | LOC442132	ENST00000512838	ENSE00002064798	7150917	7151141	-0.4473119	0.469065933	0.765246612
kgp6061903	2	28671677	FOSL2 | PLB1	ENST00000439700	ENSE00001803576	28671034	28671737	0.41758945	0.469127453	0.765246612
kgp10564577	7	149700010	ATP6V0E2 | LOC100286961	ENST00000565102	ENSE00002598017	149697841	149700167	-0.49565005	0.469190432	0.765246612
kgp3324324	5	6705819	SRD5A1 | POLS	ENST00000503989	ENSE00002071199	6705221	6707824	0.416175079	0.469987182	0.765246612
kgp8915015	12	126451196	TMEM132B | LOC10028870	ENST00000545784	ENSE00002228181	126450746	126451888	0.517124522	0.470317724	0.765246612
kgp7344269	3	172309226	TNFSF10 | AADACL1	ENST00000418839	ENSE00001731231	172308503	172311504	-0.426141694	0.470335973	0.765246612
rs1383740	11	38676519	LOC100129825 | LOC10028	ENST00000534756	ENSE00002166839	38676510	38676802	0.41741588	0.470645965	0.765246612
kgp4836667	5	180112934	FLT4 | OR2Y1	ENST00000513535	ENSE00002038277	180112649	180113546	0.414016897	0.470920992	0.765246612
kgp2145051	12	102354842	DRAM | CCDC53	ENST00000547004	ENSE00002369834	102354808	102354900	0.199224709	0.559651122	0.766119963
kgp16408216	16	80636184	DYNLRB2 | CDYL2	ENST00000570137	ENSE00002617613	80631803	80636416	-0.367121966	0.559832483	0.766119963
rs13129831	4	8357645	HTRA3 | ACOX3	ENST00000505448	ENSE00002069127	8357038	8359103	-0.17820768	0.560703016	0.766442288
kgp19481288	14	45233586	FSCB | C14orf28	ENST00000556405	ENSE00002460394	45232360	45234829	-0.485414576	0.56386248	0.769889155
kgp4517182	4	27282287	STIM2 | PCDH7	ENST00000512873	ENSE00002064550	27281892	27283847	0.430968207	0.476669741	0.77027196
kgp26687452	3	187167154	RTP4 | SST	ENST00000440726	ENSE00001709446	187167039	187167238	-0.656158503	0.477604525	0.77027196
kgp14095156	17	14324262	HS3ST3B1 | FLJ45831	ENST00000436469	ENSE00001711591	14323974	14324343	0.725887591	0.480155373	0.7714856
kgp63527	9	37088475	LOC100287249 | ZCCHC7	ENST00000429493	ENSE00001770690	37086665	37090398	-0.119403174	0.566040572	0.771989796
kgp2739883	11	130736113	C11orf44 | SNX19	ENST00000525716	ENSE00002194756	130735365	130737889	-0.192297783	0.568486254	0.773402723
kgp8320633	2	19658852	OSR1 | TTC32	ENST00000443897	ENSE00001763511	19657981	19659081	0.30842514	0.568938163	0.773402723
rs7122860	11	58273098	OR5B12 | OR5B21	ENST00000527054	ENSE00002152631	58272942	58273231	0.209218623	0.569497658	0.773402723
kgp3991266	15	95024760	MCTP2 | LOC440311	ENST00000565106	ENSE00002296294	95024040	95027181	0.114660995	0.56963962	0.773402723
kgp22943973	11	107184483	GUCY1A2 | CWF19L2	ENST00000561746	ENSE00002621788	107182858	107186997	-0.119390917	0.570375981	0.77353237
kgp17829041	3	156800955	LEKR1 | CCNL1	ENST00000471357	ENSE00001879700	156799456	156801064	0.347828238	0.571042721	0.773567412
kgp30026889	5	86401355	COX7C | RASA1	ENST00000503349	ENSE00002060653	86401232	86401355	-0.428768535	0.483726367	0.774323177
rs1054476	22	25609572	CRYBB3 | CRYBB2	ENST00000454253	ENSE00001601351	25609270	25609652	0.155853337	0.57376449	0.776363471
rs9506949	13	23426172	FGF9 | LOC646201	ENST00000445646	ENSE00001782287	23426135	23426331	0.16797147	0.575032894	0.776363471
rs12625213	20	48894919	CEBPB | PTPN1	ENST00000445003	ENSE00001632288	48894714	48894964	-0.273567918	0.575036407	0.776363471
kgp508554	11	68915045	TPCN2 | MYEOV	ENST00000562772	ENSE00002613001	68914696	68915208	0.406971411	0.490835665	0.777159827
kgp8667665	6	22222240	FLJ22536 | PRL	ENST00000567753	ENSE00002610158	22221010	22222624	0.525349728	0.490886403	0.777159827
rs11914753	3	172309040	TNFSF10 | AADACL1	ENST00000418839	ENSE00001731231	172308503	172311504	-0.40541109	0.492840039	0.777159827
kgp1327549	22	17152462	psiTPTE22 | XKR3	ENST00000457060	ENSE00002603498	17152408	17152478	0.497387433	0.494321085	0.777159827
rs941605	14	50410547	ARF6 | C14orf182	ENST00000556913	ENSE00002499815	50408654	50410610	-0.609090576	0.495054048	0.777159827
kgp734490	22	37749536	CYTH4 | ELFN2	ENST00000445088	ENSE00001716394	37749166	37750879	0.584239611	0.498229566	0.777159827
rs949633	14	51426327	PYGL | TRIM9	ENST00000553648	ENSE00002451667	51422977	51427333	0.516366994	0.498585934	0.777159827
kgp4587973	4	150075716	NR3C2 | LOC100287246	ENST00000503100	ENSE00002071198	150075447	150075771	0.483604661	0.499990937	0.777159827
kgp19567427	14	85996332	SEL1L | FLRT2	ENST00000380722	ENSE00001486021	85994961	85996332	-0.522320241	0.577412093	0.778283616
kgp9457348	2	43268621	HAAO | ZFP36L2	ENST00000438736	ENSE00001785368	43268332	43268782	0.385861684	0.579322377	0.778283616
kgp19141109	12	3153012	TEAD4 | TSPAN9	ENST00000513358	ENSE00002234190	3152644	3154116	-0.385114031	0.579549238	0.778283616
rs9951290	18	65152131	CDH19 | DSEL	ENST00000562669	ENSE00002612652	65149028	65152203	-0.291453991	0.579671337	0.778283616
kgp4576934	10	3027128	LOC727878 | PFKP	ENST00000446337	ENSE00001695564	3027098	3027331	-0.215881936	0.57968266	0.778283616
kgp7645717	16	67583154	FAM65A | CTCF	ENST00000565929	ENSE00002588122	67583122	67583636	-0.200530866	0.580567109	0.778605001
kgp1457537	8	116976293	TRPS1 | EIF3H	ENST00000505156	ENSE00002079147	116976018	116976383	-0.412295187	0.503088595	0.779043764
kgp11354686	4	133045142	LOC100128747 | PCDH10	ENST00000420721	ENSE00002061637	133045006	133045186	-0.38531943	0.504834886	0.779043764
kgp9146827	17	47447283	ZNF652 | PHB	ENST00000510360	ENSE00002063712	47447161	47447360	-0.207968124	0.581684738	0.779238045
kgp24695646	22	46451788	C22orf26 | LOC150381	ENST00000439423	ENSE00001755261	46451620	46452395	-0.431175917	0.584871747	0.782638801
rs2763529	14	103654939	TNFAIP2 | LOC441698	ENST00000514902	ENSE00002084072	103654929	103655365	-0.43349213	0.569209069	0.78266247
kgp4107162	21	46410547	C21orf70 | NCRNA00162	ENST00000439088	ENSE00001802548	46409779	46411747	0.380682749	0.585860454	0.783093652
kgp639868	3	134067137	RYK | AMOTL2	ENST00000568384	ENSE00002597828	134066130	134068075	-0.262013757	0.572755737	0.784271342
rs2802929	1	235095444	PP2672 | TOMM20	ENST00000451766	ENSE00001647592	235095413	235095663	-0.37969369	0.511613706	0.786184368
rs2033670	7	22929061	SNORD93 | FAM126A	ENST00000421730	ENSE00001632532	22928990	22929312	0.37884334	0.513127326	0.786184368
kgp6728477	14	101538059	SNORD114-31 | LOC10013C	ENST00000444846	ENSE00001687158	101537124	101539271	0.613621635	0.519043868	0.786300891
kgp27810841	12	31521753	FAM60A | DENND5B	ENST00000541749	ENSE00002274720	31521683	31522235	0.443137218	0.521579591	0.786300891
rs773280	9	93867445	SYK | LOC100128909	ENST00000423380	ENSE00001640521	93867239	93867664	-0.27822405	0.576697432	0.786405589
kgp3445621	16	66445707	CDH5 | BEAN	ENST00000499966	ENSE00001976529	66444183	66446038	-0.118852371	0.589160698	0.786633808
kgp11793372	4	24671505	DHX15 | SOD3	ENST00000569621	ENSE00002600133	24671185	24673191	-0.421303789	0.52475911	0.787138665
kgp5683150	2	231556354	SP100 | CAB39	ENST00000415174	ENSE00001768231	231555636	231556948	0.345880898	0.590700177	0.787817806
kgp9317226	12	114182800	LHX5 | RBM19	ENST00000547963	ENSE00002355822	114182382	114184400	-0.42467106	0.528141052	0.788270227
kgp1757663	3	72371563	PROK2 | RYBP	ENST00000570082	ENSE00002588390	72370202	72373178	0.367318652	0.518453442	0.788311395

kgp22763691	1	145382349	NBPF10 | HFE2	ENST00000433081	ENSE00001721985	145382056	145382397	-0.529255741	0.519509225	0.788311395
kgp509157	2	14541059	TRIB2 | FAM84A	ENST00000418420	ENSE00001736425	14540967	14541082	0.375953317	0.520028263	0.788311395
kgp2262827	7	56562396	LOC100132050 | DKFZp434	ENST00000566570	ENSE00002614277	56560817	56564978	0.116185332	0.59264374	0.788392131
kgp25776497	16	22623010	LOC653786 | HS3ST2	ENST00000567401	ENSE00002582095	22621852	22623517	0.25469775	0.592832367	0.788392131
kgp25782006	16	66787026	DYNC1LI2 | CCDC79	ENST00000501143	ENSE00001980181	66786899	66788643	-0.192096978	0.593556654	0.788392131
kgp19841568	15	98564578	ARRDC4 | FAM169B	ENST00000554798	ENSE00002533240	98564417	98565007	0.215704735	0.594093942	0.788392131
rs12241885	10	60086547	CISD1 | UBE2D1	ENST00000562575	ENSE00002589065	60085374	60086790	0.11255896	0.595248347	0.788392131
kgp9005396	16	80636066	DYNLRB2 | CDYL2	ENST00000570137	ENSE00002617613	80631803	80636416	-0.37822816	0.596543477	0.788392131
rs602524	7	42941854	GLI3 | C7orf25	ENST00000569883	ENSE00002599349	42940871	42942238	-0.191518074	0.5971274	0.788392131
kgp18388454	9	100000731	ZNF322B | KIAA1529	ENST00000366109	ENSE00001779242	100000705	100000960	-0.227425056	0.597526638	0.788392131
kgp1164261	5	9550333	SNORD123 | TAS2R1	ENST00000508179	ENSE00002021903	9549414	9550721	0.219679886	0.597710065	0.788392131
kgp20045802	15	95024271	MCTP2 | LOC440311	ENST00000565106	ENSE00002296294	95024040	95027181	0.126031442	0.598081416	0.788392131
kgp19872751	15	95024153	MCTP2 | LOC440311	ENST00000565106	ENSE00002296294	95024040	95027181	0.10826616	0.598315818	0.788392131
kgp4054081	5	104135199	NUDT12 | RAB9P1	ENST00000523745	ENSE00002101316	104135007	104135473	0.404993947	0.522566382	0.78936964
rs10105114	8	127889918	FAM84B | POU5F1B	ENST00000519319	ENSE00002095146	127889917	127890035	-0.13796406	0.60199848	0.791386458
rs1322771	1	173832806	DARS2 | GAS5	ENST00000416578	ENSE00001660333	173832364	173833079	-0.145818212	0.602000398	0.791386458
kgp9579269	16	80636297	DYNLRB2 | CDYL2	ENST00000570137	ENSE00002617613	80631803	80636416	-0.457882093	0.603090329	0.791386458
kgp12239174	14	57535254	LOC440180 | EXOC5	ENST00000554160	ENSE00002431637	57535091	57535260	0.114399545	0.603546915	0.791386458
kgp19340007	20	17868423	BANF2 | SNX5	ENST00000425405	ENSE00001740096	17868007	17868448	0.185621597	0.604066247	0.791386458
kgp5986893	1	181144914	IER5 | LOC100287948	ENST00000438428	ENSE00001755862	181144846	181144970	-0.312279829	0.604522216	0.791386458
kgp11957568	1	29197202	OPRD1 | EPB41	ENST00000443593	ENSE00001675297	29197025	29197779	-0.290984302	0.605742616	0.792070052
kgp7230170	2	47006748	SOCS5 | LOC388948	ENST00000568862	ENSE00002622745	47004922	47007384	-0.157908234	0.606356858	0.792070052
kgp12857509	11	29357543	METT5D1 | KCNA4	ENST00000528553	ENSE00002190104	29357445	29357629	0.157383029	0.607029739	0.792091779
kgp8671372	12	114182892	LHX5 | RBM19	ENST00000547963	ENSE00002355822	114182382	114184400	-0.275367276	0.583526851	0.792443872
kgp13142311	21	46410693	C21orf70 | NCRNA00162	ENST00000439088	ENSE00001802548	46409779	46411747	0.469930624	0.527325425	0.793763534
kgp8694881	1	2361799	PEX10 | PLCH2	ENST00000420406	ENSE00001621165	2361777	2362089	-0.473873872	0.534920965	0.793982644
rs6583249	3	195587257	MUC4 | TNK2	ENST00000429834	ENSE00001713552	195587144	195587275	0.417726414	0.53979266	0.793982644
rs12679136	8	38401296	C8orf86 | RNF5P1	ENST00000521623	ENSE00002130210	38401170	38401364	-0.409132385	0.539908198	0.793982644
rs229706	14	45234728	FSCB | C14orf28	ENST00000556405	ENSE00002460394	45232360	45234829	-0.441782759	0.611551349	0.796707084
kgp7801308	3	67356099	KBTBD8 | SUCLG2	ENST00000484222	ENSE00001882711	67354804	67356783	-0.214381284	0.611886882	0.796707084
kgp19487067	14	45234560	FSCB | C14orf28	ENST00000556405	ENSE00002460394	45232360	45234829	-0.466398334	0.613002088	0.797299052
kgp17392091	6	134749697	SGK1 | LOC645175	ENST00000417483	ENSE00001603855	134749378	134750353	-0.253246107	0.616562525	0.800140817
kgp4780429	7	6694930	ZNF316 | LOC100133111	ENST00000564837	ENSE00002606444	6694495	6696063	-0.279910635	0.617907076	0.800140817
kgp8642927	15	95026114	MCTP2 | LOC440311	ENST00000565106	ENSE00002296294	95024040	95027181	-0.114861416	0.617981756	0.800140817
kgp26228682	6	134750031	SGK1 | LOC645175	ENST00000417483	ENSE00001603855	134749378	134750353	0.220580985	0.618285466	0.800140817
rs3778928	7	156396540	C7orf4 | C7orf13	ENST00000439364	ENSE00002627938	156396312	156396605	-0.192346545	0.618501559	0.800140817
kgp2686827	2	9247144	MBOAT2 | ASAP2	ENST00000565044	ENSE00002602192	9246722	9249994	-0.212243834	0.619202399	0.800189824
kgp3203121	15	44827024	CTDSPL2 | LOC645212	ENST00000313807	ENSE00001427514	44825691	44827672	-0.116887948	0.620700847	0.801268366
kgp9675382	16	81421590	GAN | CMIP	ENST00000568107	ENSE00002626991	81416874	81424489	0.099173222	0.621691301	0.80168953
kgp7655117	1	56724232	LOC100288320 | PPAP2B	ENST00000569425	ENSE00002626501	56724041	56724243	0.120786198	0.623373129	0.802281483
kgp8844965	5	9549308	SNORD123 | TAS2R1	ENST00000509788	ENSE00002040542	9549102	9549702	-0.34645748	0.623479728	0.802281483
kgp17668999	3	142842321	CHST2 | LOC100289567	ENST00000483262	ENSE00001815386	142842204	142842418	0.141792311	0.625002425	0.803384374
kgp6392248	14	70920134	SYNJ2BP | ADAM21	ENST00000556646	ENSE00002446855	70920124	70920226	-0.376846584	0.549422093	0.804032331
kgp28315808	14	71075556	MED6 | TTC9	ENST00000500016	ENSE00001980392	71075515	71078067	-0.340969102	0.594962319	0.804662153
kgp1402525	3	195563659	MUC4 | TNK2	ENST00000413586	ENSE00001689436	195563625	195563916	-0.250687601	0.597772562	0.805163042
kgp8426413	18	77830707	C18orf22 | ADNP2	ENST00000568911	ENSE00002616791	77830238	77830758	0.219977701	0.602399687	0.805422118
rs12187328	5	81841967	ATP6AP1L | TMEM167A	ENST00000512952	ENSE00002025952	81841681	81842129	-0.403199128	0.60481265	0.805422118
kgp22836267		1517727	LOC100286886 | NCRNA00	ENST00000434938	ENSE00001676140	1517718	1517852	-0.330159485	0.605286925	0.805422118
kgp9835740	11	65211392	NCRNA00084 | MALAT1	ENST00000501122	ENSE00001961965	65190269	65213011	-0.136481307	0.627527649	0.805772205
rs2980185	4	4324233	ZNF509 | D4S234E	ENST00000509015	ENSE00002051780	4323689	4324272	0.183335698	0.628535636	0.806208833
kgp5146196	8	10338256	MSRA | LOC346702	ENST00000518098	ENSE00002137308	10338056	10338496	0.222277057	0.611680726	0.807200209
kgp16452718	16	50681634	NKD1 | SNX20	ENST00000563424	ENSE00002621697	50679720	50683060	0.3003133	0.611713806	0.807200209
kgp12217421	10	49872421	ARHGAP22 | WDFY4	ENST00000440750	ENSE00001731397	49872244	49872457	0.214160791	0.615149342	0.807200209
kgp8482816	4	142245202	ZNF330 | LOC100286983	ENST00000509161	ENSE00002080359	142244576	142247161	-0.365993665	0.616407432	0.807200209
rs11969660	6	14395373	CD83 | JARID2	ENST00000434947	ENSE00001591299	14395284	14395502	0.359610783	0.557537749	0.808897249
kgp4318693	21	35336115	FLJ46020 | MRPS6	ENST00000381181	ENSE00001487739	35334367	35336260	0.386653366	0.558139102	0.808897249
kgp5214431	15	93324046	LOC643797 | CHD2	ENST00000562894	ENSE00002583293	93322987	93324722	-0.35928622	0.561545014	0.809920693
kgp26215500	6	3594601	LOC643327 | LOC10012962	ENST00000443445	ENSE00001746929	3594481	3594769	-0.174541783	0.632349196	0.810239362
kgp10178160	1	112903084	LOC643355 | CTTNBP2NL	ENST00000427290	ENSE00001597844	112902984	112903150	-0.507405777	0.541898082	0.810921746
kgp10847517	12	114182632	LHX5 | RBM19	ENST00000547963	ENSE00002355822	114182382	114184400	-0.468849541	0.545228694	0.810921746
kgp28978087	8	79751192	IL7 | STMN2	ENST00000565297	ENSE00002583800	79749764	79752757	-0.408478426	0.549303055	0.810921746
rs7615701	3	147997618	FLJ30375 | AGTR1	ENST00000460324	ENSE00001847818	147997569	147997885	0.365540141	0.551813959	0.810921746
kgp4731771	2	42120360	SLC8A1 | LOC400950	ENST00000398796	ENSE00001534923	42119669	42121179	0.586610994	0.55282512	0.810921746
rs1487111	3	81296550	LOC728290 | GBE1	ENST00000464131	ENSE00001936785	81296244	81296693	0.334829363	0.553985127	0.810921746
kgp7860114	12	5426775	KCNA5 | NTF3	ENST00000544842	ENSE00002302652	5425127	5428513	0.425053308	0.554627144	0.810921746
kgp4747738	7	156377086	C7orf4 | C7orf13	ENST00000414767	ENSE00002627951	156376854	156377214	-0.331286645	0.558341523	0.810921746
kgp3116185	22	27960098	MIAT | MN1	ENST00000415655	ENSE00001737567	27959968	27960394	-0.331816683	0.558933969	0.810921746
kgp4338642	1	98433830	DPYD | FLJ35409	ENST00000561881	ENSE00002583625	98432561	98434370	-0.573863763	0.562941025	0.810921746
kgp2861290	8	71383400	NCOA2 | TRAM1	ENST00000520259	ENSE00002092378	71383369	71384080	-0.921827851	0.563081489	0.810921746
kgp14715812	2	235370622	LOC100287159 | ARL4C	ENST00000418025	ENSE00001670233	235370511	235370771	0.328020099	0.563146403	0.810921746
kgp17355693	6	148455134	SAMD5 | SASH1	ENST00000417838	ENSE00001659900	148454945	148455274	-0.551196208	0.563297623	0.810921746
kgp1177731	15	45740911	C15orf48 | SLC30A4	ENST00000559869	ENSE00002543658	45740625	45740999	-0.361829217	0.567933388	0.81148014
kgp9078369	14	97410013	VRK1 | C14orf64	ENST00000495064	ENSE00001912215	97409976	97410017	0.481040073	0.568036098	0.81148014
kgp19890743	15	95026217	MCTP2 | LOC440311	ENST00000565106	ENSE00002296294	95024040	95027181	-0.115257172	0.63493516	0.81202639
kgp8942322	16	89979851	TCF25 | MC1R	ENST00000539976	ENSE00002202946	89979640	89981576	-0.295819427	0.635089406	0.81202639
rs13395865	2	75159869	HK2 | POLE4	ENST00000435984	ENSE00001619573	75159788	75159921	0.177141757	0.635832852	0.81211667
kgp5214966	16	56643517	MT2A | MT1L	ENST00000569778	ENSE00002603822	56643413	56643796	0.379969958	0.636793399	0.812483756
kgp1146734	3	156800874	LEKR1 | CCNL1	ENST00000471357	ENSE00001879700	156799456	156801064	0.319076254	0.64143877	0.817546564
kgp18926644	12	5134599	KCNA1 | LOC390282	ENST00000538202	ENSE00002282958	5134260	5134758	0.555180335	0.575266261	0.817914115
kgp12922274	11	130735608	C11orf44 | SNX19	ENST00000525716	ENSE00002194756	130735365	130737889	-0.223689388	0.627314848	0.818059407
kgp7512966	4	125443322	LOC100289258 | ANKRD50	ENST00000563724	ENSE00002599704	125443262	125443359	-0.222784432	0.633717546	0.818059407
kgp19679411	14	69658700	WDR22 | EXD2	ENST00000557409	ENSE00002500040	69658613	69658809	-0.270861244	0.634042076	0.818059407
kgp8201025	6	28806629	LOC646160 | TRIM27	ENST00000457253	ENSE00001738519	28806527	28806783	0.251897211	0.634615783	0.818059407
kgp7205928	15	92036501	LOC100128403 | SLCO3A1	ENST00000554333	ENSE00002461538	92036423	92038080	-0.421854914	0.570743085	0.818892253
rs12592573	15	43035004	CDAN1 | TTBK2	ENST00000500850	ENSE00002588053	43034200	43035400	-0.15235483	0.644082268	0.820049892
kgp1479019	3	112863072	C3orf17 | BOC	ENST00000490139	ENSE00001823459	112861197	112863182	-0.151798309	0.64509759	0.820258461

kgp15434136	1	144594393	LOC728875 | C1orf152	ENST00000428365	ENSE00001765747	144594389	144594483	0.196148397	0.647024644	0.820258461
kgp4962530	6	3911054	LOC100289591 | PRPF4B	ENST00000566733	ENSE00002617674	3905144	3912213	0.183498716	0.64789731	0.820258461
kgp6118629	2	42120156	SLC8A1 | LOC400950	ENST00000442214	ENSE00001777568	42119669	42120186	-0.13211992	0.648361785	0.820258461
kgp7000606	2	9247365	MBOAT2 | ASAP2	ENST00000565044	ENSE00002602192	9246722	9249994	-0.161117982	0.648513961	0.820258461
rs28602975	15	40604862	PLCB2 | C15orf52	ENST00000559030	ENSE00002547681	40604816	40608835	0.096859564	0.648958038	0.820258461
rs2030558	1	175889627	TNR | RFWD2	ENST00000426575	ENSE00001619283	175889592	175889649	0.135248514	0.649634404	0.820258461
rs2293877	14	70935875	ADAM21 | ADAM20	ENST00000556964	ENSE00002518821	70935598	70938309	0.138181831	0.649682758	0.820258461
rs2271155	11	13943228	FAR1 | SPON1	ENST00000532065	ENSE00002196971	13942997	13943508	0.252683697	0.639993412	0.821781424
rs749985	16	50673651	NKD1 | SNX20	ENST00000565077	ENSE00002621561	50671504	50674771	0.203500189	0.651903807	0.821917385
kgp10819851	2	34902925	MYADML | LOC100288911	ENST00000423663	ENSE00001665890	34902646	34902994	-0.10085161	0.652358621	0.821917385
kgp9900138	1	48226939	LOC100287946 | LOC10028	ENST00000438589	ENSE00001628376	48226804	48226952	-0.236746411	0.655427602	0.824194134
rs7413116	1	182057506	ZNF648 | LOC100130996	ENST00000428646	ENSE00001766058	182057406	182057611	-0.100000582	0.656004529	0.824194134
kgp11923777	15	25281586	PAR5 | SNORD109A	ENST00000552334	ENSE00002349489	25277020	25281637	-0.123911777	0.656586857	0.824194134
kgp1892404	14	85995608	SEL1L | FLRT2	ENST00000380722	ENSE00001486021	85994961	85996332	0.105734849	0.656897065	0.824194134
kgp10121216	10	25940716	GPR158 | MYO3A	ENST00000450270	ENSE00001689485	25940704	25940728	0.393273134	0.582608636	0.824446183
rs7152848	14	70937121	ADAM21 | ADAM20	ENST00000556964	ENSE00002518821	70935598	70938309	0.106972851	0.659922123	0.827129806
rs3742406	14	101537857	SNORD114-31 | LOC10013	ENST00000444846	ENSE00001687158	101537124	101539271	0.706870402	0.580164503	0.829635239
kgp21897376	10	47098286	PPYR1 | LOC728643	ENST00000422732	ENSE00001719699	47096454	47098649	0.266778435	0.663174164	0.830038883
kgp7133701	2	208100648	KLF7 | CREB1	ENST00000448786	ENSE00001726953	208100497	208100790	0.212754728	0.663618494	0.830038883
kgp4644237	22	32556062	C22orf42 | RFPL2	ENST00000426354	ENSE00001617153	32555888	32556379	0.330680535	0.582622046	0.830073749
kgp7099851	16	33950151	LOC100287647 | TOP	ENST00000567668	ENSE00002585926	33950099	33950415	-0.406199051	0.584340961	0.830073749
kgp2979841	11	58902056	FAM111B | FAM111A	ENST00000501817	ENSE00001976137	58901697	58903061	-0.101154418	0.665166199	0.83111346
kgp21307700	4	129489637	PGRMC2 | PHF17	ENST00000514265	ENSE00002048854	129489127	129490442	-0.260635888	0.667007534	0.832552321
kgp6482818	7	95103202	PON2 | ASB4	ENST00000416593	ENSE00001607208	95103005	95103310	0.212993886	0.650932208	0.832587708
kgp6882297	5	147648287	SPINK6 | SPINK5L3	ENST00000501695	ENSE00001960784	147647870	147648567	-0.134426777	0.667971089	0.832868691
kgp11111788	17	13680460	HS3ST3A1 | CDRT15P	ENST00000423323	ENSE00001689622	13679951	13680666	-0.125254933	0.668641062	0.832868691
kgp18769827	12	54144466	CALCOCO1 | HOXC13	ENST00000570015	ENSE00002617645	54144231	54144749	-0.4446859	0.590311077	0.833681176
kgp2849736	6	10423406	TFAP2A | C6orf218	ENST00000420389	ENSE00001701886	10423373	10423736	-0.391927679	0.59076708	0.833681176
kgp22268821	5	81841912	ATP6AP1L | TMEM167A	ENST00000512952	ENSE00002025952	81841681	81842129	-0.520815516	0.592929226	0.833988977
rs6836534	4	188230701	FAT1 | ZFP42	ENST00000511385	ENSE00002081969	188230336	188230836	0.379136306	0.655083497	0.834662371
kgp2852749	21	46410070	C21orf70 | NCRNA00162	ENST00000439088	ENSE00001802548	46409779	46411747	0.431085466	0.598160357	0.835476197
kgp22149599	5	142084124	FGF1 | ARHGAP26	ENST00000566527	ENSE00002614243	142083347	142084986	-0.702277926	0.600945361	0.835476197
kgp25224607	1	222157999	LOC100129950 | LOC72861	ENST00000441160	ENSE00001600434	222157396	222158306	0.304005697	0.601409454	0.835476197
rs17505533	10	34206497	NRP1 | PARD3	ENST00000434552	ENSE00001645316	34206472	34206732	0.330263053	0.603148256	0.835476197
kgp19510955	14	97411597	VRK1 | C14orf64	ENST00000495064	ENSE00001951246	97411575	97411731	-0.291374309	0.60455039	0.835476197
kgp5895749	5	3419111	C5orf38 | IRX1	ENST00000505443	ENSE00002060518	3417266	3419430	0.295772434	0.6063647	0.835476197
kgp11784813	8	64346513	YTHDF3 | LOC100130155	ENST00000521061	ENSE00002110564	64346381	64346567	-0.452588048	0.608334099	0.835476197
kgp2993701	1	92865424	RPAP2 | GFI1	ENST00000564442	ENSE00002592577	92864531	92867613	-0.657115296	0.60956655	0.835476197
kgp3390669	6	3908438	LOC100289591 | PRPF4B	ENST00000566733	ENSE00002617674	3905144	3912213	-0.328854775	0.595591397	0.835643651
rs8032343	15	96946875	LOC728800 | SPATA8	ENST00000558382	ENSE00002573184	96946740	96946964	-0.329897846	0.596148289	0.835643651
kgp11737626	14	83262456	SEL1L | FLRT2	ENST00000554451	ENSE00002514857	83262417	83262565	-0.324450222	0.601701493	0.835643651
kgp2091	5	142137424	FGF1 | ARHGAP26	ENST00000432677	ENSE00001732056	142137392	142137499	-0.353108868	0.602165624	0.835643651
kgp8318067	12	5134684	KCNA1 | LOC390282	ENST00000538202	ENSE00002282958	5134260	5134758	0.442206264	0.604448907	0.835643651
kgp20555202	8	16989102	EFHA2 | ZDHHC2	ENST00000513892	ENSE00002077855	16988690	16990578	-0.350737887	0.6608859	0.836470796
kgp8173195	4	129489460	PGRMC2 | PHF17	ENST00000514265	ENSE00002048854	129489127	129490442	0.202571892	0.661572357	0.836470796
kgp11665722	1	4008029	LOC728716 | LOC284661	ENST00000425194	ENSE00001656760	4008017	4008655	0.311172671	0.612650219	0.838229244
kgp3448365	16	80864621	CDYL2 | C16orf61	ENST00000569356	ENSE00002618300	80864323	80864654	-0.319340044	0.612734443	0.838229244
rs4296932	6	3908474	LOC100289591 | PRPF4B	ENST00000566733	ENSE00002617674	3905144	3912213	-0.3125601	0.614701445	0.838229244
rs236212	10	120118598	C10orf84 | PRLHR	ENST00000445161	ENSE00001686734	120118509	120118715	0.288251924	0.667775009	0.839028396
kgp9461852	3	67355523	KBTBD8 | SUCLG2	ENST00000484222	ENSE00001882711	67354804	67356783	0.19233878	0.66957055	0.839028396
kgp25047105	1	234768889	IRF2BP2 | PP2672	ENST00000429269	ENSE00001771955	234768694	234770526	0.301793771	0.671222717	0.839028396
kgp14260887	2	64750374	HSPC159 | AFTPH	ENST00000561559	ENSE00002598792	64749321	64751227	0.107828901	0.674419897	0.83920084
kgp5117236	8	99383627	NIPAL2 | KCNS2	ENST00000518704	ENSE00002131207	99383583	99383906	-0.133632482	0.675398221	0.839325086
kgp5915630	2	20878994	GDF7 | C2orf43	ENST00000565841	ENSE00002602242	20877569	20879005	0.102015512	0.675910509	0.839325086
rs1346266	15	45694516	GATM | SPATA5L1	ENST00000458245	ENSE00001988574	45694097	45694525	0.141775605	0.676636409	0.839362945
rs12546910	8	10334723	MSRA | LOC346702	ENST00000524047	ENSE00002099410	10334633	10334804	0.11636975	0.678443046	0.840298233
kgp5201750	20	29641550	FRG1B | DEFB115	ENST00000446917	ENSE00001678897	29641421	29641792	0.144700701	0.67961224	0.840298233
kgp14230708	2	10179300	UNQ5830 | KLF11	ENST00000567540	ENSE00002582289	10179219	10180790	0.093117218	0.680090013	0.840298233
kgp3669476	11	82783814	RAB30 | PCF11	ENST00000534499	ENSE00002181888	82783665	82784236	0.084037063	0.680538732	0.840298233
rs7283594	21	35335100	ATP5O | FLJ46020	ENST00000381181	ENSE00001487739	35334367	35336260	0.179238192	0.680871311	0.840298233
rs328632	2	9247782	MBOAT2 | ASAP2	ENST00000565044	ENSE00002602192	9246722	9249994	0.304954719	0.620249652	0.840349167
kgp17442249	6	142580757	VTA1 | GPR126	ENST00000426166	ENSE00001602386	142580541	142580769	-0.34367725	0.62249508	0.840349167
kgp11054107	2	75159375	HK2 | POLE4	ENST00000418204	ENSE00001757721	75159326	75159416	0.379785856	0.624659547	0.840349167
kgp9409222	15	41201382	VPS18 | LOC100288995	ENST00000503052	ENSE00002073016	41201027	41201540	0.184891432	0.675448483	0.841124526
kgp11194237	3	176352224	NAALADL2 | TBL1XR1	ENST00000434969	ENSE00001632374	176352133	176352323	0.188473549	0.682352355	0.841265876
kgp5756558	17	13680506	HS3ST3A1 | CDRT15P	ENST00000423323	ENSE00001689622	13679951	13680666	-0.095939431	0.683486119	0.841624645
kgp7629225	7	65960365	LOC100289098 | LOC34632	ENST00000452565	ENSE00001780443	65960242	65960459	-0.110670726	0.684037926	0.841624645
rs2559619	17	27876561	TAOK1 | ABHD15	ENST00000562535	ENSE00002584590	27873905	27878921	-0.080991355	0.685396821	0.842437844
kgp17672377	3	112782125	C3orf17 | BOC	ENST00000462010	ENSE00001923793	112781954	112782290	-0.083218666	0.686397183	0.842520139
kgp11652270	2	217475831	RPL37A | IGFBP2	ENST00000441803	ENSE00001659572	217475467	217475925	-0.212428558	0.686859832	0.842520139
kgp8985009	22	27620674	MIAT | MN1	ENST00000444114	ENSE00001656532	27620484	27620688	0.319729099	0.617654735	0.843865864
kgp6574949	14	96108480	GLRX5 | TCL6	ENST00000555032	ENSE00002481784	96108355	96109622	0.423452831	0.622070779	0.844071789
kgp20790922	4	38455190	TBC1D1 | FLJ13197	ENST00000512517	ENSE00002022007	38454844	38455542	-0.514235021	0.623053594	0.844071789
kgp9981789	5	87730027	TMEM161B | LOC645323	ENST00000504034	ENSE00002031149	87729702	87730095	-0.351862232	0.623708058	0.844071789
rs2374377	2	42120306	SLC8A1 | LOC400950	ENST00000398796	ENSE00001534923	42119669	42121179	0.173070794	0.691239586	0.847002095
rs6111720	20	17868623	BANF2 | SNX5	ENST00000425405	ENSE00001676865	17868615	17868804	-0.12778364	0.691917204	0.847002095
kgp1272991	1	234807458	IRF2BP2 | PP2672	ENST00000442382	ENSE00001695948	234805269	234808736	-0.234850053	0.692980391	0.847444105
kgp10498417	11	46197992	PHF21A | CREB3L1	ENST00000533793	ENSE00002149414	46197900	46198656	0.088907659	0.696122066	0.849854907
rs17329485	5	9549386	SNORD123 | TAS2R1	ENST00000509788	ENSE00002040542	9549102	9549702	-0.231518817	0.696749704	0.849854907
rs17153381	11	58901763	FAM111B | FAM111A	ENST00000501817	ENSE00001976137	58901697	58903061	-0.111190304	0.697064091	0.849854907
kgp6065128	22	47762564	LOC100289420 | FAM19A5	ENST00000434707	ENSE00001737535	47762518	47762565	0.444916438	0.630020672	0.849933548
kgp17454019	6	142580634	VTA1 | GPR126	ENST00000426166	ENSE00001602386	142580541	142580769	-0.245681754	0.688776923	0.852439326
rs940894	3	154687338	GPR149 | MME	ENST00000486545	ENSE00001822288	154687072	154687471	0.175207077	0.692268549	0.852439326
kgp2905701	1	234807113	IRF2BP2 | PP2672	ENST00000442382	ENSE00001695948	234805269	234808736	-0.238449296	0.6942234	0.852439326
kgp20410111	8	10338190	MSRA | LOC346702	ENST00000518098	ENSE00002137308	10338056	10338496	0.175268722	0.695650411	0.852439326
kgp16917976	6	24751855	C6orf62 | GMNN	ENST00000453179	ENSE00001787704	24751793	24752149	0.247553156	0.697450358	0.852439326

kgp22743701	16	68607903	ZFP90 | CDH3	ENST00000569654	ENSE00002581868	68607685	68608126	-0.146181687	0.701056107	0.853859456
kgp4470127	11	82902169	PCF11 | ANKRD42	ENST00000530825	ENSE00002152969	82902105	82902210	-0.10510751	0.702092878	0.854260185
kgp28586516	8	102088235	YWHAZ | ZNF706	ENST00000514926	ENSE00002063679	102087397	102088479	0.30267129	0.638389289	0.854392561
kgp14396894	2	187219851	FLJ44048 | ZC3H15	ENST00000564407	ENSE00002576512	187219299	187221500	-0.301767629	0.642685167	0.854392561
kgp10242702	16	54400736	LOC728792 | LOC643911	ENST00000566649	ENSE00002604056	54399919	54401571	-0.321629225	0.643642396	0.854392561
kgp6983371	6	15079924	CD83 | JARID2	ENST00000437648	ENSE00001709379	15079865	15080062	0.088273788	0.704396921	0.856200487
rs1349151	9	22767236	DMRTA1 | LOC402360	ENST00000448570	ENSE00001738020	22767174	22767265	0.163359321	0.703584495	0.856763407
rs3749541	4	153143877	PET112L | FBXW7	ENST00000504785	ENSE00002033587	153143620	153143911	0.279194845	0.649351657	0.858173995
kgp10042721	16	66444938	CDH5 | BEAN	ENST00000499966	ENSE00001976529	66444183	66446038	-0.096948549	0.707263701	0.858820208
kgp10600170	2	59153017	LOC644456 | LOC730134	ENST00000452840	ENSE00001655364	59152746	59153044	-0.223504699	0.708992954	0.860175275
kgp22810112	22	50231187	BRD1 | ZBED4	ENST00000565177	ENSE00002605050	50230940	50231434	0.272128627	0.640818685	0.861790646
kgp19727208	14	62028050	PRKCH | HIF1A	ENST00000508827	ENSE00002070975	62027461	62031959	0.093993389	0.710877563	0.862340923
kgp7297825	7	121290341	FAM3C | PTPRZ1	ENST00000432702	ENSE00001656139	121290307	121290414	-0.267756174	0.644291269	0.862545151
kgp4600137	8	102088357	YWHAZ | ZNF706	ENST00000514926	ENSE00002063679	102087397	102088479	-0.343318542	0.647251662	0.862545151
kgp9552593	2	42104329	SLC8A1 | LOC400950	ENST00000418836	ENSE00001627859	42104214	42104441	0.682789499	0.647411512	0.862545151
kgp27368509	9	37086960	LOC100287249 | ZCCHC7	ENST00000429493	ENSE00001770690	37086665	37090398	0.079449237	0.711881317	0.862691515
kgp24205251	2	38054468	LOC344382 | FAM82A1	ENST00000413792	ENSE00001797876	38053390	38055021	-0.388263772	0.658228071	0.863829651
kgp10068841	2	154213815	ARL6IP6 | LOC642635	ENST00000429916	ENSE00001803289	154213770	154213936	0.274852256	0.659389967	0.863829651
rs9405967	6	6682321	LY86 | RREB1	ENST00000563225	ENSE00002602400	6680542	6683866	0.107315909	0.714905938	0.865371772
rs2625527	15	72115438	NR2E3 | MYO9A	ENST00000561834	ENSE00002626554	72115404	72115477	0.07959151	0.716497932	0.865371772
kgp4996440	14	50413234	ARF6 | C14orf182	ENST00000556130	ENSE00002433545	50412872	50413432	-0.080855822	0.717571442	0.865371772
kgp11772879	14	101415324	SNORD113-9 | SNORD114-	ENST00000556099	ENSE00002455129	101415315	101415401	0.173623043	0.718486296	0.865371772
kgp2309787	8	29387420	LOC100132051 | C8orf75	ENST00000521101	ENSE00002099631	29387308	29387840	-0.082769556	0.719027327	0.865371772
kgp18658665	9	37088182	LOC100287249 | ZCCHC7	ENST00000429493	ENSE00001770690	37086665	37090398	0.085900575	0.719424664	0.865371772
rs3872612	3	112782186	C3orf17 | BOC	ENST00000462010	ENSE00001923793	112781954	112782290	-0.079463098	0.719449843	0.865371772
rs6872005	5	133772648	CDKN2AIPNL | PHF15	ENST00000513329	ENSE00002067519	133772402	133772724	0.224282435	0.719828715	0.865371772
kgp19468992	14	50428488	ARF6 | C14orf182	ENST00000556913	ENSE00002460979	50428421	50428678	-0.15126576	0.71859755	0.866023718
rs10827311	10	34201760	NRP1 | PARD3	ENST00000434552	ENSE00001760715	34201696	34201761	0.166802821	0.722731937	0.866023718
kgp6327093	15	45689237	GATM | SPATA5L1	ENST00000527933	ENSE00002558080	45689120	45689544	0.15409879	0.723675452	0.866023718
kgp4408883	2	42120150	SLC8A1 | LOC400950	ENST00000442214	ENSE00001777568	42119669	42120186	-0.233928206	0.724310746	0.866023718
kgp11307707	15	25278763	PAR5 | SNORD109A	ENST00000552334	ENSE00002349489	25277020	25281637	0.071001417	0.721395916	0.866392906
kgp17313435	6	3911127	LOC100289591 | PRPF4B	ENST00000566733	ENSE00002617674	3905144	3912213	0.276192894	0.653773389	0.868324408
rs2946740	17	27875865	TAOK1 | ABHD15	ENST00000562535	ENSE00002584590	27873905	27878921	-0.073520818	0.725778428	0.870746973
kgp25988232	13	80144951	NDFIP2 | LOC729479	ENST00000450187	ENSE00001680800	80144698	80144983	0.098871794	0.726464128	0.870746973
kgp22820674	19	28223197	LOC100101266 | LOC14818	ENST00000561521	ENSE00002619204	28221181	28223355	-0.101274907	0.727721752	0.871389043
kgp8251318	15	30297714	LOC727808 | LOC10012843	ENST00000561392	ENSE00002564316	30297646	30297992	-0.218187391	0.728937516	0.871979764
kgp5370323	9	37087160	LOC100287249 | ZCCHC7	ENST00000429493	ENSE00001770690	37086665	37090398	-0.078333602	0.730269398	0.872141559
kgp160476	12	132671437	NOC4L | GALNT9	ENST00000538731	ENSE00002290912	132671287	132672246	-0.096472802	0.730517909	0.872141559
kgp8607176	16	80633731	DYNLRB2 | CDYL2	ENST00000570137	ENSE00002617613	80631803	80636416	0.183870291	0.732472144	0.872620244
kgp11431094	12	4915903	GALNT8 | KCNA6	ENST00000542988	ENSE00002284510	4915708	4916034	-0.371436689	0.659488655	0.87321183
kgp5211460	16	80635000	DYNLRB2 | CDYL2	ENST00000570137	ENSE00002617613	80631803	80636416	-0.218812934	0.733484685	0.873678392
kgp6416915	16	80636149	DYNLRB2 | CDYL2	ENST00000570137	ENSE00002617613	80631803	80636416	-0.201155833	0.733882041	0.873678392
kgp13326289	7	6694607	ZNF316 | LOC100133111	ENST00000564837	ENSE00002606444	6694495	6696063	-0.220498634	0.733976711	0.873678392
rs2834083	21	34436973	OLIG2 | OLIG1	ENST00000453716	ENSE00001625299	34436909	34437289	0.250086076	0.663673095	0.874423422
kgp15558273	1	99953344	LPPR4 | PALMD	ENST00000438829	ENSE00001628512	99953301	99953360	-0.370513286	0.665731832	0.874423422
kgp10436960	8	10335262	MSRA | LOC346702	ENST00000520494	ENSE00002093248	10335001	10335291	-0.262088244	0.666518552	0.874423422
kgp13066882	11	74953574	LOC441617 | ARRB1	ENST00000562197	ENSE00002609763	74953064	74954742	0.183792267	0.736811119	0.874631904
kgp7886332	5	117619966	LOC100287135 | DTWD2	ENST00000503877	ENSE00002021170	117619408	117620477	-0.406514364	0.670155027	0.87651374
kgp5290557	1	17523235	LOC400743 | PADI1	ENST00000539219	ENSE00002261175	17520556	17524112	-0.149136602	0.737360072	0.876840993
kgp4515723	9	37086996	LOC100287249 | ZCCHC7	ENST00000429493	ENSE00001770690	37086665	37090398	-0.071996775	0.74157193	0.878340772
rs6937846	6	134775632	SGK1 | LOC645175	ENST00000422736	ENSE00001717108	134775591	134775719	0.132077253	0.741996903	0.878340772
rs16880892	5	52409245	LOC257396 | FST	ENST00000499459	ENSE00001982677	52408070	52410956	-0.083872459	0.742100166	0.878340772
kgp10684241	11	65211505	NCRNA00084 | MALAT1	ENST00000501122	ENSE00001961965	65190269	65213011	-0.075776677	0.742742028	0.878340772
kgp13990243	17	53638398	MMD | TMEM100	ENST00000455347	ENSE00001598631	53638318	53638452	-0.15773117	0.742916631	0.878340772
kgp12554412	14	50409192	ARF6 | C14orf182	ENST00000556913	ENSE00002499815	50408654	50410610	-0.136484408	0.743151595	0.878340772
kgp12522920	21	40361023	FLJ45139 | LOC100289305	ENST00000417335	ENSE00001798412	40360633	40361352	0.105325401	0.744320769	0.878340772
kgp24501146	2	75158653	HK2 | POLE4	ENST00000435984	ENSE00001696016	75158545	75158663	0.159338876	0.744442925	0.878340772
kgp1030644	19	28223070	LOC100101266 | LOC14818	ENST00000561521	ENSE00002619204	28221181	28223355	-0.106054795	0.746637052	0.88006926
rs7252041	19	28248720	LOC100101266 | LOC14818	ENST00000562493	ENSE00002598618	28248092	28251757	0.108433342	0.747515444	0.880245016
kgp9019169	12	6504037	LTBR | SRP14P1	ENST00000541888	ENSE00002315622	6503673	6504235	0.072712235	0.749439383	0.881650424
rs35907069	2	69940780	AAK1 | ANXA4	ENST00000415342	ENSE00001593127	69940744	69940979	-0.289444685	0.676188356	0.881984812
rs6437416	3	194500372	FAM43A | C3orf21	ENST00000455796	ENSE00001647637	194500191	194500387	0.189704677	0.746733585	0.883233273
kgp29776887	10	47098318	PPYR1 | LOC728643	ENST00000422732	ENSE00001719699	47096454	47098649	0.188913212	0.751770459	0.883531591
kgp22798043	12	133609645	ZNF26 | ZNF84	ENST00000443154	ENSE00001973759	133609245	133609890	-0.270854081	0.68143072	0.884974961
kgp7173635	2	150714672	LOC642340 | LOC10028952	ENST00000295052	ENSE00001070095	150713965	150715117	-0.179751778	0.75160882	0.885824681
kgp8736704	16	80633775	DYNLRB2 | CDYL2	ENST00000570137	ENSE00002617613	80631803	80636416	0.120529537	0.754644801	0.88604696
kgp6327342	8	29535429	LOC100132051 | C8orf75	ENST00000518623	ENSE00002090317	29535426	29535520	-0.112224827	0.757968945	0.887810219
kgp6992674	9	37086756	LOC100287249 | ZCCHC7	ENST00000430809	ENSE00001609838	37086665	37086871	-0.066266158	0.758332594	0.887810219
rs2457400	8	80733475	HEY1 | MRPS28	ENST00000517365	ENSE00002138364	80733363	80733545	-0.132178488	0.75835322	0.887810219
kgp14249279	2	19167909	NT5C1B | OSR1	ENST00000424895	ENSE00001627691	19167729	19169146	-0.064981321	0.761045715	0.889300079
kgp7118498	5	1939989	IRX4 | IRX2	ENST00000513419	ENSE00002070223	1939886	1940055	0.074837864	0.761099405	0.889300079
kgp4601770	2	64750639	HSPC159 | AFTPH	ENST00000561559	ENSE00002598792	64749321	64751227	0.107935673	0.761956196	0.889440163
kgp17298325	6	8785472	HULC | OFCC1	ENST00000429060	ENSE00001620931	8785350	8785678	-0.082757662	0.764297545	0.88997554
kgp4406698	8	16990531	EFHA2 | ZDHHC2	ENST00000513892	ENSE00002077855	16988690	16990578	-0.122130839	0.76447535	0.88997554
kgp22737403		1518262	LOC100286886 | NCRNA001	ENST00000430235	ENSE00001640360	1518260	1518295	0.114367595	0.764997741	0.88997554
kgp6576301	14	96389594	TCL1A | C14orf132	ENST00000504119	ENSE00002072462	96389118	96391899	0.115107198	0.765364217	0.88997554
rs873319	1	17523806	LOC400743 | PADI1	ENST00000539219	ENSE00002261175	17520556	17524112	-0.140159117	0.767204762	0.891008707
kgp9426591	16	81422926	GAN | CMIP	ENST00000568107	ENSE00002626991	81416874	81424489	-0.061541425	0.767729126	0.891008707
kgp24491727	2	187221392	FLJ44048 | ZC3H15	ENST00000564407	ENSE00002576512	187219299	187221500	0.259933212	0.689675673	0.891821991
rs4754214	11	107186896	GUCY1A2 | CWF19L2	ENST00000561746	ENSE00002621788	107182858	107186997	-0.117009985	0.769272883	0.891942718
rs643958	9	22768289	DMRTA1 | LOC402360	ENST00000448570	ENSE00001671272	22767961	22768315	-0.066951425	0.771946475	0.89418368
rs16951296	16	79791574	MAF | DYNLRB2	ENST00000568389	ENSE00002607025	79791548	79791612	0.093855277	0.77284679	0.894368241
rs6436409	2	224363562	KCNE4 | SCG2	ENST00000422118	ENSE00001765766	224363491	224363574	0.2596608	0.689211825	0.895440264
kgp585573	2	4005884	LOC728597 | SOX11	ENST00000454455	ENSE00001657033	4005245	4006531	0.22494884	0.690806765	0.895440264
kgp74019	21	44255112	PDE9A | WDR4	ENST00000431150	ENSE00001683445	44255055	44255143	-0.309168998	0.690887477	0.895440264
rs12186736	5	9549755	SNORD123 | TAS2R1	ENST00000508179	ENSE00002021903	9549414	9550721	-0.080003108	0.778046598	0.898509492

kgp19813501	15	95026450	MCTP2 | LOC440311	ENST00000565106	ENSE00002296294	95024040	95027181	0.077014186	0.778278092	0.898509492
rs3088103	22	26920048	TFIP11 | TPST2	ENST00000564772	ENSE00002607234	26917949	26921002	-0.066236144	0.779197822	0.898509492
kgp6413874	1	234664365	TARBP1 | IRF2BP2	ENST00000435574	ENSE00001674386	234663637	234666088	0.11160214	0.779431953	0.898509492
kgp25687903	16	72460813	PMFBP1 | ZFHX3	ENST00000564508	ENSE00002602918	72459847	72463072	-0.170069071	0.78014743	0.898509492
kgp1823963	11	68640556	CPT1A | MRPL21	ENST00000512200	ENSE00002055373	68639011	68642010	-0.188092857	0.767164022	0.899069268
kgp758480	20	10845344	JAG1 | LOC728573	ENST00000421788	ENSE00001602101	10845303	10845376	0.126023878	0.768295556	0.899069268
kgp9819137	3	194553294	FAM43A | C3orf21	ENST00000427064	ENSE00001752865	194553191	194553321	0.220144126	0.695918091	0.899243557
kgp12189884	16	70611232	SF3B3 | IL34	ENST00000562874	ENSE00002580772	70611221	70611571	0.056930365	0.783717627	0.900427032
kgp20066789	8	23316492	ENTPD4 | SLC25A37	ENST00000521021	ENSE00002110503	23316114	23316604	0.066189518	0.78474672	0.900427032
kgp21002885	4	8357970	HTRA3 | ACOX3	ENST00000505448	ENSE00002069127	8357038	8359103	-0.07662536	0.784883032	0.900427032
kgp9119901	14	76669866	C14orf118 | ESRRB	ENST00000555426	ENSE00002532654	76669600	76669870	0.054737733	0.785282981	0.900427032
kgp11268048	9	37089150	LOC100287249 | ZCCHC7	ENST00000429493	ENSE00001770690	37086665	37090398	0.056729703	0.78554239	0.900427032
kgp18226433	3	149694878	PFN2 | TMEM183B	ENST00000471176	ENSE00001834458	149694542	149694931	0.267148548	0.699824615	0.901061736
kgp24507670	2	19658404	OSR1 | TTC32	ENST00000443897	ENSE00001763511	19657981	19659081	0.211547866	0.787018833	0.901263502
rs6414814	5	68263602	PIK3R1 | SLC30A5	ENST00000513197	ENSE00002054308	68263494	68263672	-0.251432219	0.70453862	0.903254642
kgp9142835	2	139370544	SPOPL | NXPH2	ENST00000453636	ENSE00001804638	139370108	139370737	0.094216398	0.789823164	0.903268651
kgp4508681	9	37088167	LOC100287249 | ZCCHC7	ENST00000429493	ENSE00001770690	37086665	37090398	-0.058589875	0.790266525	0.903268651
kgp5369267	14	70936985	ADAM21 | ADAM20	ENST00000556964	ENSE00002518821	70935598	70938309	0.080844014	0.792222873	0.904318115
rs16912133	9	98177956	FANCC | PTCH1	ENST00000433644	ENSE00001650490	98177781	98178446	0.088184862	0.792683153	0.904318115
kgp2172007	13	33909145	STARD13 | RFC3	ENST00000443576	ENSE00001780719	33909142	33909403	-0.26469615	0.703433804	0.905077553
kgp6878653	14	86595036	FLRT2 | LOC730121	ENST00000553668	ENSE00002492185	86595009	86595117	-0.228358066	0.704652454	0.905077553
kgp12115169	5	7151032	POLS | LOC442132	ENST00000512838	ENSE00002064798	7150917	7151141	-0.210247832	0.707016675	0.905403444
kgp8807289	4	165722265	ANP32C | LOC100131276	ENST00000515485	ENSE00002027020	165722243	165722606	-0.373893143	0.716268668	0.907181822
kgp11175861	6	151518403	MTHFD1L | AKAP12	ENST00000431947	ENSE00001774294	151517816	151518499	0.205567018	0.716458722	0.907181822
kgp5050910	12	93501119	LOC100287580 | NUDT4	ENST00000551928	ENSE00002372876	93501084	93501376	0.234296863	0.716624235	0.907181822
kgp5269667	9	132243226	C9orf106 | LOC100128077	ENST00000565882	ENSE00002629303	132242884	132244817	-0.463749619	0.716863957	0.907181822
kgp11855544	12	101802215	ARL1 | SPIC	ENST00000547360	ENSE00002350103	101802150	101802309	0.058896779	0.796680397	0.908020056
kgp1234301	5	6706577	SRD5A1 | POLS	ENST00000503989	ENSE00002071199	6705221	6707824	-0.071054208	0.797508815	0.908106736
kgp19447913	14	50428561	ARF6 | C14orf182	ENST00000556913	ENSE00002460979	50428421	50428678	-0.202308493	0.722406878	0.911507502
kgp25286918	1	223318208	TLR5 | SUSD4	ENST00000435108	ENSE00001645603	223317879	223318296	0.099320599	0.801716369	0.912037377
kgp6434193	3	177470032	TBL1XR1 | KCNMB2	ENST00000439009	ENSE00001751027	177469699	177470093	0.144822541	0.802495451	0.912064039
kgp14408723	2	19168858	NT5C1B | OSR1	ENST00000424895	ENSE00001627691	19167729	19169146	0.117198202	0.782369568	0.912303737
kgp19296983	20	61665585	BHLHE23 | HAR1B	ENST00000414668	ENSE00001642533	61665569	61666020	0.237206346	0.71578358	0.912892433
kgp2214213	11	94373795	PIWIL4 | AMOTL1	ENST00000438416	ENSE00002263259	94373749	94373999	0.239695919	0.719449063	0.912892433
kgp1222348	15	92148148	LOC100128403 | SLCO3A1	ENST00000555947	ENSE00002464925	92148107	92148441	0.228338008	0.721185022	0.912892433
kgp3930169	3	112770212	C3orf17 | BOC	ENST00000462010	ENSE00001882700	112769759	112770552	-0.083361143	0.804804897	0.913828327
kgp22767126		21915499	MBTPS2 | SMS	ENST00000449605	ENSE00001619106	21913079	21920026	-0.227446262	0.729920698	0.914194231
kgp14285831	2	38685971	ATL2 | LOC100288550	ENST00000437979	ENSE00001770651	38685779	38686017	0.222015573	0.730025147	0.914194231
kgp3745231	20	62668845	PRPF6 | PRR17	ENST00000444463	ENSE00001722043	62667490	62669555	-0.251757829	0.731355385	0.914194231
kgp2293821	2	214020749	IKZF2 | SPAG16	ENST00000426870	ENSE00001739685	214020530	214020926	-0.211980363	0.734716355	0.914538427
kgp3384238	4	38454939	TBC1D1 | FLJ13197	ENST00000512517	ENSE00002022007	38454844	38455542	-0.253068902	0.737727665	0.914538427
kgp3534534	3	176353305	NAALADL2 | TBL1XR1	ENST00000434969	ENSE00001802272	176353229	176353320	0.145791548	0.787985633	0.914917326
rs2271154	11	13943261	FAR1 | SPON1	ENST00000532065	ENSE00002196971	13942997	13943508	-0.137597438	0.791920118	0.914917326
kgp19691834	14	55729819	DLGAP5 | FBXO34	ENST00000556183	ENSE00002476405	55729626	55729874	-0.15740564	0.792928349	0.914917326
kgp11366240	11	130736271	C11orf44 | SNX19	ENST00000525716	ENSE00002194756	130735365	130737889	-0.053234461	0.809674018	0.918492988
rs1026378	8	2662135	LOC100286951 | CSMD1	ENST00000520024	ENSE00002138262	2662104	2662227	0.081812132	0.811651914	0.919416933
kgp10531694	14	64827903	LOC441687 | MTHFD1	ENST00000556556	ENSE00002468564	64827828	64827969	-0.05805537	0.812011972	0.919416933
kgp18871889	12	59106010	XRCC6BP1 | LRIG3	ENST00000552201	ENSE00002417202	59105966	59106134	-0.278193801	0.736035509	0.921819962
kgp14867557	2	189030529	LOC344328 | LOC729141	ENST00000434418	ENSE00001743340	189030481	189030635	0.337737988	0.736816115	0.921819962
kgp1116785	3	194553230	FAM43A | C3orf21	ENST00000427064	ENSE00001752865	194553191	194553321	0.208771034	0.739431285	0.921819962
kgp10348263	14	101537956	SNORD114-31 | LOC10013(	ENST00000444846	ENSE00001687158	101537124	101539271	0.343007286	0.739930473	0.921819962
rs10139825	14	39982933	FBXO33 | LRFN5	ENST00000555555	ENSE00002534029	39982821	39982983	-0.183644213	0.741668639	0.921819962
kgp13199666	21	40249911	ETS2 | FLJ45139	ENST00000544859	ENSE00002214012	40249215	40252276	-0.307951241	0.74347251	0.921819962
kgp187846	4	34123006	PCDH7 | ARAP2	ENST00000513843	ENSE00002074574	34122883	34123077	0.419225306	0.748746007	0.92212123
kgp553573	21	46411077	C21orf70 | NCRNA00162	ENST00000439088	ENSE00001802548	46409779	46411747	0.284819427	0.75029881	0.92212123
rs1868240	3	127209361	C3orf56 | GPR175	ENST00000479610	ENSE00001821750	127209201	127209630	0.178624625	0.751485246	0.92212123
kgp3815498	5	10197299	LOC285692 | FAM173B	ENST00000566945	ENSE00002600148	10195233	10197740	-0.233800174	0.752949977	0.92212123
kgp1062162	20	10847812	JAG1 | LOC728573	ENST00000421788	ENSE00001594655	10847661	10847830	-0.22119941	0.754462824	0.92212123
kgp4165907	5	28782903	CDH9 | LOC729862	ENST00000504398	ENSE00002048763	28782785	28782942	-0.106170795	0.802734307	0.923004604
kgp14671042	2	217082974	XRCC5 | MARCH4	ENST00000562038	ENSE00002618694	217081768	217084915	-0.07595035	0.816748333	0.923320243
kgp3134537	16	81424117	GAN | CMIP	ENST00000568107	ENSE00002626991	81416874	81424489	0.045385244	0.816989246	0.923320243
GA030593	15	72114747	NR2E3 | MYO9A	ENST00000563041	ENSE00002601016	72114376	72114796	0.048707863	0.817905292	0.923490821
kgp7414971	14	55729755	DLGAP5 | FBXO34	ENST00000556183	ENSE00002476405	55729626	55729874	-0.153899244	0.807122096	0.924827402
kgp22122115	5	65867719	FLJ46010 | MAST4	ENST00000535074	ENSE00002320902	65866144	65867801	-0.217941696	0.760390641	0.924838265
kgp14328302	2	20878050	GDF7 | C2orf43	ENST00000565841	ENSE00002602242	20877569	20879005	-0.185049248	0.760997453	0.924838265
rs207898	2	217084199	XRCC5 | MARCH4	ENST00000562038	ENSE00002618694	217081768	217084915	0.047651688	0.820901706	0.926007812
kgp23098910	11	74954523	LOC441617 | ARRB1	ENST00000562197	ENSE00002609763	74953064	74954742	0.095326527	0.823641345	0.928230722
kgp12001477	5	6702784	SRD5A1 | POLS	ENST00000503989	ENSE00002041186	6702671	6702822	0.100470733	0.813312401	0.928695821
rs785003	8	128032251	FAM84B | POU5F1B	ENST00000561978	ENSE00002619091	128031889	128033259	-0.050953776	0.82596531	0.929981464
kgp2400433	5	114540351	TRIM36 | PGGT1B	ENST00000507241	ENSE00002051389	114539713	114541943	0.045526666	0.827935607	0.931331107
kgp10446625	9	107753342	ABCA1 | SLC44A1	ENST00000457720	ENSE00001727188	107753077	107753584	0.076312129	0.829414534	0.931340553
rs6443513	3	177614691	TBL1XR1 | KCNMB2	ENST00000436078	ENSE00001606478	177614352	177617012	-0.065065805	0.830123864	0.931340553
rs10870544	12	133491098	CHFR | LOC100289635	ENST00000503695	ENSE00002082163	133489682	133491318	-0.090150187	0.830258852	0.931340553
kgp8405671	12	10550007	KLRK1 | KLRC4	ENST00000500682	ENSE00001969973	10548778	10551105	-0.120762892	0.821649774	0.931452666
rs633727	11	64659424	LOC100127953 | ATG2A	ENST00000413053	ENSE00001657856	64658898	64660639	0.096411814	0.823181095	0.931452666
kgp6753356	8	9051487	PPP1R3B | TNKS	ENST00000523747	ENSE00002111128	9051468	9051542	-0.096429982	0.825369965	0.931452666
kgp10377951	12	114182452	LHX5 | RBM19	ENST00000547963	ENSE00002355822	114182382	114184400	0.095035129	0.827017064	0.931452666
rs4666376	2	19244727	NT5C1B | OSR1	ENST00000449124	ENSE00001621793	19244622	19244767	0.061685971	0.833712866	0.93269282
kgp18193555	3	152557506	P2RY1 | LOC100287133	ENST00000460407	ENSE00001822402	152557179	152559228	-0.054749444	0.83378181	0.93269282
kgp22995070	11	130737194	C11orf44 | SNX19	ENST00000525716	ENSE00002194756	130735365	130737889	-0.041504535	0.833782563	0.93269282
rs7200991	16	81417366	GAN | CMIP	ENST00000568107	ENSE00002626991	81416874	81424489	-0.049012645	0.835869462	0.934161519
kgp129026	6	3908378	LOC100289591 | PRPF4B	ENST00000566733	ENSE00002617674	3905144	3912213	-0.201995327	0.756971894	0.934378171
rs791623	5	173138434	BOD1 | CPEB4	ENST00000521128	ENSE00002126658	173137443	173138951	0.195320085	0.762803144	0.934378171
kgp18928766	12	52218080	FIGNL2 | LOC728503	ENST00000567167	ENSE00002607709	52217894	52218295	-0.197107343	0.764836936	0.934378171
kgp10780578	14	101537377	SNORD114-31 | LOC10013(	ENST00000444846	ENSE00001687158	101537124	101539271	0.232003554	0.7661901	0.934378171
rs957795	21	46758719	C21orf93 | COL18A1	ENST00000416722	ENSE00001708983	46758505	46758793	0.201154974	0.772327706	0.934662919

rs1437768	15	96948362	LOC728800 | SPATA8	ENST00000561097	ENSE00002560198	96948340	96948412	-0.201150839	0.773439012	0.934662919
GA026048	9	132104153	C9orf106 | LOC100128077	ENST00000444125	ENSE00001744622	132104015	132104322	-0.041315415	0.837132374	0.93470747
kgp19685549	14	44948036	LRFN5 | FSCB	ENST00000556472	ENSE00002489125	44947932	44948054	-0.196823307	0.769709712	0.934870095
kgp922696	5	31742410	C5orf22 | PDZD2	ENST00000506865	ENSE00002067179	31741940	31742434	0.175948947	0.772957177	0.935028843
kgp19913624	15	95027139	MCTP2 | LOC440311	ENST00000565106	ENSE00002296294	95024040	95027181	-0.043342818	0.838779443	0.93568095
rs11182232	12	44115989	ADAMTS20 | PUS7L	ENST00000553202	ENSE00002345647	44112796	44117803	0.047299686	0.841743431	0.936959639
rs7536392	1	180877409	XPR1 | KIAA1614	ENST00000434447	ENSE00001732933	180877339	180877427	0.077190509	0.842045033	0.936959639
kgp24092585	2	10179633	UNQ5830 | KLF11	ENST00000567540	ENSE00002582289	10179219	10180790	-0.044491579	0.842369896	0.936959639
kgp18400594	9	37087561	LOC100287249 | ZCCHC7	ENST00000429493	ENSE00001770690	37086665	37090398	-0.039646224	0.843381717	0.936959639
kgp19978790	15	77336133	PSTPIP1 | TSPAN3	ENST00000560446	ENSE00002551850	77336022	77336179	0.054423844	0.843962307	0.936959639
kgp7523274	5	173138306	BOD1 | CPEB4	ENST00000521128	ENSE00002126658	173137443	173138951	-0.100401307	0.844583337	0.936959639
kgp17494280	6	84697823	LOC100132652 | MRAP2	ENST00000420766	ENSE00001660777	84697811	84697915	-0.143713332	0.835348876	0.937636494
rs2244382	5	10137363	LOC285692 | FAM173B	ENST00000506299	ENSE00002044261	10137250	10137378	-0.182951384	0.785519134	0.937677416
kgp7640060	1	41730196	SCMH1 | FOXO6	ENST00000425554	ENSE00001621807	41730073	41730230	0.174667489	0.786391818	0.937677416
kgp3724109	15	93324280	LOC643797 | CHD2	ENST00000562894	ENSE00002583293	93322987	93324722	-0.168814866	0.78777666	0.937677416
rs1013375	5	127277217	LOC728586 | FLJ33630	ENST00000514853	ENSE00002045046	127276874	127277326	-0.17228908	0.790428902	0.937677416
rs11728462	4	55822867	KIT | KDR	ENST00000509711	ENSE00002033685	55822808	55822985	0.191922306	0.796193231	0.937677416
kgp9139368	16	72258743	PMFBP1 | ZFHX3	ENST00000563347	ENSE00002609413	72258464	72258758	-0.182316825	0.798541259	0.937677416
kgp1837741	4	185818635	ACSL1 | HELT	ENST00000515864	ENSE00002063897	185818502	185818721	0.228402012	0.80286222	0.937677416
kgp11877637	7	95103233	PON2 | ASB4	ENST00000416593	ENSE00001607208	95103005	95103310	0.16525509	0.806260366	0.937677416
kgp6878336	21	40251714	FLJ45139 | LOC100289305	ENST00000544859	ENSE00002214012	40249215	40252276	0.150629845	0.808498139	0.937677416
kgp5285967	7	95101273	PON2 | ASB4	ENST00000416593	ENSE00001716343	95101147	95101374	0.143747246	0.814504459	0.937677416
kgp24157622	2	3129103	MYT1L | LOC729897	ENST00000457478	ENSE00001720874	3129067	3129210	-0.144061699	0.816046127	0.937677416
kgp1864651	17	13694933	HS3ST3A1 | CDRT15P	ENST00000423323	ENSE00001666236	13693586	13695867	0.141374625	0.816297341	0.937677416
rs16864862	2	224368870	KCNE4 | SCG2	ENST00000422118	ENSE00001754732	224368700	224369329	-0.143456803	0.818709615	0.937677416
kgp4636970	11	87831173	TMEM135 | RAB38	ENST00000531454	ENSE00002150113	87831080	87831315	0.147507631	0.818904943	0.937677416
kgp20555593	8	2532314	LOC100286951 | CSMD1	ENST00000523971	ENSE00002098887	2532238	2532353	0.052904399	0.846122795	0.937805522
kgp28068341	14	63589781	KCNH5 | RHOJ	ENST00000554921	ENSE00002440324	63589751	63590478	0.040713658	0.847654146	0.937852449
kgp30384952	5	37939581	GDNF | EGFLAM	ENST00000510938	ENSE00002069111	37939467	37939771	-0.072386666	0.847719156	0.937852449
kgp758094	12	104565620	NFYB | TXNRD1	ENST00000547554	ENSE00002363303	104565551	104565814	-0.102417617	0.840200324	0.938027895
kgp6612401	6	6682828	LY86 | RREB1	ENST00000563225	ENSE00002602400	6680542	6683866	-0.084202228	0.841562252	0.938027895
kgp12345206	14	36344344	BRMS1L | LOC100289323	ENST00000553046	ENSE00002366044	36343986	36344509	0.098372092	0.844225106	0.938027895
kgp7147966	9	98178345	FANCC | PTCH1	ENST00000433644	ENSE00001650490	98177781	98178446	0.059221812	0.849111595	0.938223185
kgp9150544	12	10516417	KLRD1 | KLRK1	ENST00000500682	ENSE00001975845	10516368	10516632	-0.059530227	0.849608899	0.938223185
rs561582	11	87831251	TMEM135 | RAB38	ENST00000531454	ENSE00002150113	87831080	87831315	-0.095887087	0.85042622	0.939051563
kgp9614989	3	177470070	TBL1XR1 | KCNMB2	ENST00000439009	ENSE00001751027	177469699	177470093	-0.117709624	0.855276142	0.939051563
rs6702559	1	234806995	IRF2BP2 | PP2672	ENST00000442382	ENSE00001695948	234805269	234808736	0.168364671	0.855756272	0.939051563
kgp6025741	6	42060747	TAF8 | C6orf132	ENST00000562471	ENSE00002625332	42059976	42061997	0.07518831	0.856832823	0.939051563
kgp23015929	11	19322720	E2F8 | NAV2	ENST00000529082	ENSE00002200326	19322708	19322791	0.132570003	0.85937446	0.939051563
kgp10255779	16	79550067	LOC729251 | MAF	ENST00000569938	ENSE00002606211	79549878	79550190	0.183016296	0.780191144	0.94017416
kgp14714046	2	179267007	OSBPL6 | PRKRA	ENST00000565104	ENSE00002619842	179265561	179267618	-0.057254152	0.852603114	0.940669066
kgp6808369	5	114540766	TRIM36 | PGGT1B	ENST00000507241	ENSE00002051389	114539713	114541943	-0.047876766	0.853618994	0.940929795
kgp9886595	19	36199020	UPK1A | ZBTB32	ENST00000450758	ENSE00001658541	36198868	36199158	-0.073388246	0.864463061	0.941494423
kgp18021509	3	177614588	TBL1XR1 | KCNMB2	ENST00000436078	ENSE00001606478	177614352	177617012	-0.090554508	0.855165344	0.941774243
kgp28067162	14	105562065	GPR132 | JAG2	ENST00000548361	ENSE00002354972	105561877	105562104	-0.046654422	0.857527732	0.943515016
kgp5938366	9	120417972	ASTN2 | TLR4	ENST00000450938	ENSE00001776391	120417886	120418065	-0.078343062	0.85969644	0.945039712
rs2839734	15	95024344	MCTP2 | LOC440311	ENST00000565106	ENSE00002296294	95024040	95027181	0.037403518	0.861166772	0.945747328
kgp26195235	6	6681773	LY86 | RREB1	ENST00000563225	ENSE00002602400	6680542	6683866	0.037268946	0.861981468	0.945747328
kgp7716855	2	19168617	NT5C1B | OSR1	ENST00000424895	ENSE00001627691	19167729	19169146	-0.038156742	0.86269081	0.945747328
kgp9204144	12	9408168	PZP | LOC642846	ENST00000538219	ENSE00002299624	9408155	9408288	0.090792324	0.872130725	0.946136879
kgp2246886	18	77825981	C18orf22 | ADNP2	ENST00000568911	ENSE00002622433	77822752	77827308	0.068610248	0.874459843	0.946136879
kgp12058690	3	153094652	RAP2B | LOC152118	ENST00000462300	ENSE00001948898	153094620	153094684	0.174722263	0.793412531	0.946757444
rs1429723	5	117916235	LOC100287135 | DTWD2	ENST00000513366	ENSE00002047319	117916227	117916343	0.171867161	0.795492488	0.946757444
kgp13112957	21	46410140	C21orf70 | NCRNA00162	ENST00000439088	ENSE00001802548	46409779	46411747	0.251134125	0.800130169	0.946757444
kgp6858664	5	3419110	C5orf38 | IRX1	ENST00000505443	ENSE00002060518	3417266	3419430	-0.156507445	0.801623302	0.946757444
kgp9668849	12	9399110	PZP | LOC642846	ENST00000538219	ENSE00002252887	9399093	9399144	-0.227773257	0.802495828	0.946757444
kgp8599363	12	78721104	NAV3 | SYT1	ENST00000548512	ENSE00002346101	78720460	78721156	0.142665514	0.807482927	0.946757444
kgp13992262	17	69095028	LOC100133226 | LOC12468	ENST00000569074	ENSE00002589961	69093916	69095759	-0.153961153	0.808257762	0.946757444
kgp16509094	16	50680977	NKD1 | SNX20	ENST00000563424	ENSE00002621697	50679720	50683060	-0.264415533	0.81176804	0.946757444
rs9275653	6	32685865	HLA-DQB1 | HLA-DQA2	ENST00000455328	ENSE00001625505	32685807	32685924	0.134977217	0.814371704	0.946757444
kgp13181796	21	46410780	C21orf70 | NCRNA00162	ENST00000439088	ENSE00001802548	46409779	46411747	0.194314333	0.814663362	0.946757444
kgp2733937	5	123828568	CSNK1G3 | ZNF608	ENST00000503145	ENSE00002049208	123828468	123828878	0.144894283	0.815224446	0.946757444
kgp4630755	5	91378474	LOC100129716 | FLJ42709	ENST00000507217	ENSE00002052373	91378414	91378637	0.129659296	0.816098342	0.946757444
kgp9275340	8	25544679	CDCA2 | EBF2	ENST00000517964	ENSE00002099862	25544025	25545040	0.148526221	0.816540656	0.946757444
kgp22765694		1516045	LOC100286886 | NCRNA00	ENST00000430235	ENSE00001690734	1515918	1516117	0.174496209	0.818924329	0.946757444
kgp17064837	6	3906504	LOC100289591 | PRPF4B	ENST00000566733	ENSE00002617674	3905144	3912213	0.126692059	0.818989967	0.946757444
kgp6730019	1	41729724	SCMH1 | FOXO6	ENST00000422305	ENSE00001640799	41729513	41729780	-0.157522362	0.822234969	0.946757444
kgp10837439	6	140388978	CITED2 | LOC729076	ENST00000456896	ENSE00001623106	140388572	140389017	0.3091064	0.823171391	0.946757444
rs10770444	12	8449814	LOC653113 | LOC389634	ENST00000509919	ENSE00002028173	8449260	8450140	-0.047414894	0.864664385	0.947050738
kgp10850012	15	25281398	PAR5 | SNORD109A	ENST00000552334	ENSE00002349489	25277020	25281637	0.041523975	0.86642143	0.947515105
kgp17171838	6	6682145	LY86 | RREB1	ENST00000563225	ENSE00002602400	6680542	6683866	0.045910487	0.866733841	0.947515105
kgp2600843	5	127302466	LOC728586 | FLJ33630	ENST00000514573	ENSE00002041626	127302365	127302620	-0.034456611	0.867443406	0.947515105
kgp4353526	11	107184585	GUCY1A2 | CWF19L2	ENST00000561746	ENSE00002621788	107182858	107186997	0.034042867	0.868797305	0.948135937
rs3131050	6	30760025	IER3 | DDR1	ENST00000439406	ENSE00001648072	30759203	30760027	0.138209267	0.831983931	0.9490311
kgp698100	2	47007072	SOCS5 | LOC388948	ENST00000568862	ENSE00002622745	47004922	47007384	0.051305536	0.871172731	0.949173234
kgp13023911	11	65192169	FRMD8 | NCRNA00084	ENST00000501122	ENSE00001961965	65190269	65213011	0.03977496	0.871623839	0.949173234
kgp15482941	1	17521994	LOC400743 | PADI1	ENST00000539219	ENSE00002261175	17520556	17524112	0.066198341	0.8721261	0.949173234
kgp26256365	6	42060772	TAF8 | C6orf132	ENST00000562471	ENSE00002625332	42059976	42061997	0.032491444	0.872893363	0.949173234
kgp15748684	1	234807026	IRF2BP2 | PP2672	ENST00000442382	ENSE00001695948	234805269	234808736	-0.092831551	0.880388099	0.949438146
kgp18720861	12	40536506	SLC2A13 | LRRK2	ENST00000563933	ENSE00002589573	40534728	40536678	0.18772328	0.828625064	0.950481691
rs2374379	2	42120443	SLC8A1 | LOC400950	ENST00000398796	ENSE00001534923	42119669	42121179	0.176431619	0.839787901	0.951494104
kgp13151100	21	46410032	C21orf70 | NCRNA00162	ENST00000439088	ENSE00001802548	46409779	46411747	0.150908288	0.840486458	0.951494104
rs12917633	16	25097713	LOC554206 | LCMT1	ENST00000563962	ENSE00002602082	25097654	25098044	0.087930349	0.876298997	0.951607491
kgp20032201	15	95024928	MCTP2 | LOC440311	ENST00000565106	ENSE00002296294	95024040	95027181	0.034551866	0.876708807	0.951607491
kgp21382202	19	28248862	LOC100101266 | LOC14818	ENST00000562493	ENSE00002598618	28248092	28251757	0.057667025	0.878286628	0.952463576
rs766425	21	35334566	ATP5O | FLJ46020	ENST00000381181	ENSE00001487739	35334367	35336260	-0.094767184	0.880143917	0.953620922

rs17715914	5	172246510	DUSP1 | ERGIC1	ENST00000518260	ENSE00002100670	172246485	172246961	-0.123983459	0.834673388	0.954866356
kgp1282874	3	156807868	LEKR1 | CCNL1	ENST00000474477	ENSE00001873948	156807670	156807911	-0.082233136	0.888836807	0.955392858
kgp18927929	12	126470518	TMEM132B | LOC10028870	ENST00000534849	ENSE00002270684	126470392	126470780	0.071544643	0.891700001	0.955392858
kgp12394293	9	93063510	LOC100129066 | LOC34051	ENST00000425666	ENSE00001744495	93063184	93063935	-0.119649497	0.837977557	0.956096733
kgp16796617	13	48504329	HTR2A | SUCLA2	ENST00000566385	ENSE00002595049	48504290	48506757	-0.04660274	0.884129375	0.957079961
kgp6846049	3	156800914	LEKR1 | CCNL1	ENST00000471357	ENSE00001879700	156799456	156801064	0.112761633	0.88528108	0.957467979
rs8033649	15	80136288	KIAA1024 | MTHFS	ENST00000567415	ENSE00002606026	80135889	80136646	-0.04965804	0.887022129	0.95843879
kgp1600402	12	47651257	FAM113B | LOC728148	ENST00000552063	ENSE00002366145	47651245	47651322	0.060580266	0.887766833	0.95843879
kgp5769273	22	26920312	TFIP11 | TPST2	ENST00000564772	ENSE00002607234	26917949	26921002	0.033479123	0.889303869	0.959240187
rs7954843	12	44113010	ADAMTS20 | PUS7L	ENST00000553202	ENSE00002345647	44112796	44117803	-0.033707934	0.890945472	0.960152844
kgp4517195	5	151066598	SPARC | ATOX1	ENST00000510576	ENSE00002018969	151065943	151067471	0.058429905	0.901996092	0.960211925
kgp25189873	1	1363981	TMEM88B | LOC100288271	ENST00000428781	ENSE00001792190	1363502	1364430	0.096479979	0.902855197	0.960211925
kgp551038	3	134039862	RYK | AMOTL2	ENST00000482019	ENSE00001894974	134039762	134040013	0.057334914	0.906057684	0.960211925
kgp7700055	7	149742082	ATP6V0E2 | LOC100286961	ENST00000493400	ENSE00002448784	149741839	149742181	-0.050503789	0.9100138	0.960211925
kgp17669764	3	156840724	LEKR1 | CCNL1	ENST00000471357	ENSE00001898671	156840665	156840793	0.07752774	0.910958071	0.960211925
kgp9336747	7	1778741	LOC401296 | ELFN1	ENST00000453348	ENSE00001767074	1778266	1778838	-0.062221182	0.913656195	0.960211925
kgp3830550	15	76052282	DNM1P35 | LOC441728	ENST00000561777	ENSE00002618385	76051842	76054746	0.135329593	0.844969674	0.960793056
kgp12226706	12	126451452	TMEM132B | LOC10028870	ENST00000545784	ENSE00002228181	126450746	126451888	0.11657684	0.848506824	0.960793056
kgp829396	14	101542967	LOC100130814 | DIO3OS	ENST00000556136	ENSE00002481242	101542927	101543043	-0.127905605	0.849074532	0.960793056
kgp11818751	2	65131288	SERTAD2 | SLC1A4	ENST00000449259	ENSE00001774306	65128974	65131754	0.139593389	0.851052124	0.960793056
kgp3882918	18	77807228	C18orf22 | ADNP2	ENST00000562391	ENSE00002576217	77806900	77807425	-0.062766812	0.892905502	0.960889325
rs11151367	18	65151728	CDH19 | DSEL	ENST00000562669	ENSE00002612652	65149028	65152203	-0.071309518	0.893221063	0.960889325
kgp16928694	6	107144226	QRSL1 | LOC553137	ENST00000424162	ENSE00001693072	107144150	107144275	0.185968727	0.853956143	0.961541169
kgp22794170	11	68640823	CPT1A | MRPL21	ENST00000512200	ENSE00002055373	68639011	68642010	0.067346572	0.921158787	0.961698296
kgp6575876	6	10425031	TFAP2A | C6orf218	ENST00000420389	ENSE00001634310	10424991	10425085	0.058327297	0.922479971	0.961698296
kgp19151441	12	43556803	PRICKLE1 | ADAMTS20	ENST00000553211	ENSE00002370238	43556771	43556913	-0.060460888	0.923813212	0.961698296
rs16866900	2	9247025	MBOAT2 | ASAP2	ENST00000565044	ENSE00002602192	9246722	9249994	0.078233786	0.895090688	0.962043153
kgp19207527	20	29641645	FRG1B | DEFB115	ENST00000446917	ENSE00001678897	29641421	29641792	0.057698306	0.89691258	0.962508997
kgp16511915	16	81421639	GAN | CMIP	ENST00000568107	ENSE00002626991	81416874	81424489	-0.028511204	0.89711899	0.962508997
kgp4122747	10	33370602	ITGB1 | NRP1	ENST00000450890	ENSE00001755916	33370270	33371030	-0.101101717	0.898958258	0.963625771
kgp6488126	11	68640859	CPT1A | MRPL21	ENST00000512200	ENSE00002055373	68639011	68642010	0.068326867	0.931204469	0.964050488
kgp56902	14	42074011	FBXO33 | LRFN5	ENST00000557067	ENSE00002496796	42073957	42074059	0.067792555	0.931915472	0.964050488
kgp969777	2	158582500	ACVR1C | ACVR1	ENST00000447019	ENSE00001797204	158582220	158582702	-0.034367693	0.900314231	0.96422296
rs6080455	20	16844694	OTOR | PCSK2	ENST00000422574	ENSE00001619897	16844627	16844793	0.094137799	0.866345026	0.964301407
rs2402218	14	93372370	GOLGA5 | CHGA	ENST00000555299	ENSE00002497085	93372042	93373011	0.102800569	0.866379376	0.964301407
rs3742404	14	101537047	SNORD114-31 | LOC10013	ENST00000444846	ENSE00001755005	101536824	101537078	-0.295945	0.867898481	0.964301407
kgp11048014	2	42120540	SLC8A1 | LOC400950	ENST00000398796	ENSE00001534923	42119669	42121179	-0.253116172	0.873307907	0.964301407
kgp21243578	4	139019632	PCDH18 | SLC7A11	ENST00000514600	ENSE00002044680	139019607	139019667	-0.088694903	0.874065208	0.964301407
kgp14863322	2	42119944	SLC8A1 | LOC400950	ENST00000442214	ENSE00001777568	42119669	42120186	0.210171164	0.875744322	0.964301407
kgp5133633	1	9228328	GPR157 | LOC727721	ENST00000437157	ENSE00001717885	9228327	9228424	-0.1803493	0.87902807	0.964301407
kgp1180661	2	9910336	YWHAQ | TAF1B	ENST00000474667	ENSE00001815018	9910328	9910470	-0.190314453	0.879605457	0.964301407
kgp8204948	15	98674720	ARRDC4 | FAM169B	ENST00000559643	ENSE00002539033	98674663	98674836	-0.093358757	0.879853712	0.964301407
rs275430	5	6870971	POLS | LOC442132	ENST00000510622	ENSE00002051524	6870856	6870971	0.117191105	0.880083132	0.964301407
rs6997885	8	24031875	LOC100132107 | ADAM28	ENST00000521681	ENSE00002124136	24031611	24031881	-0.130606562	0.881133221	0.964301407
rs565629	21	44785510	FLJ41733 | SIK1	ENST00000435702	ENSE00001758406	44783212	44785567	0.034434294	0.901516255	0.964408816
rs17057121	13	38628067	TRPC4 | UFM1	ENST00000454060	ENSE00001783231	38627835	38628306	0.026892455	0.902245648	0.964408816
kgp22780035		46185738	LOC392452 | ZNF673	ENST00000446884	ENSE00001797372	46185359	46185889	-0.036514638	0.903422035	0.964408816
kgp10870909	5	114540463	TRIM36 | PGGT1B	ENST00000507241	ENSE00002051389	114539713	114541943	-0.02748187	0.903822658	0.964408816
kgp12074187	2	217083565	XRCC5 | MARCH4	ENST00000562038	ENSE00002618694	217081768	217084915	0.02406901	0.904482833	0.964408816
kgp1702371	15	48944056	FBN1 | LOC645405	ENST00000558061	ENSE00002563600	48943151	48944213	0.128543472	0.856833032	0.96461252
kgp19958556	15	76053553	DNM1P35 | LOC441728	ENST00000561777	ENSE00002618385	76051842	76054746	0.10860764	0.861163615	0.96461252
rs938646	8	129418532	PVT1 | LOC100287906	ENST00000520206	ENSE00002117776	129417515	129418879	0.10788592	0.861720517	0.96461252
rs1057890	22	26920270	TFIP11 | TPST2	ENST00000564772	ENSE00002607234	26917949	26921002	0.025100654	0.907525775	0.964723277
rs12476467	2	19168314	NT5C1B | OSR1	ENST00000424895	ENSE00001627691	19167729	19169146	-0.024444331	0.909147158	0.964723277
rs4853082	2	75169699	HK2 | POLE4	ENST00000453951	ENSE00001643565	75169014	75169797	-0.05940059	0.910038244	0.964723277
rs5921704		100262172	ARL13A | TRMT2B	ENST00000456301	ENSE00001736399	100261608	100262962	0.059061794	0.91022619	0.964723277
rs3824106	8	29387385	LOC100132051 | C8orf75	ENST00000521101	ENSE00002099631	29387308	29387840	0.022933312	0.910716158	0.964723277
kgp22405865	5	139486090	NRG2 | PURA	ENST00000499203	ENSE00001984051	139485881	139487228	-0.032654247	0.911620481	0.964723277
kgp3089318	8	74332283	LOC100127988 | LOC10012	ENST00000522703	ENSE00002092678	74332239	74332474	0.042812627	0.912064541	0.964723277
kgp9378796	3	156801001	LEKR1 | CCNL1	ENST00000471357	ENSE00001879700	156799456	156801064	0.090035935	0.912106854	0.964723277
kgp18384854	9	37089499	LOC100287249 | ZCCHC7	ENST00000429493	ENSE00001770690	37086665	37090398	0.027097183	0.912837631	0.964723277
kgp1182658	16	81421457	GAN | CMIP	ENST00000568107	ENSE00002626991	81416874	81424489	0.028932074	0.91313345	0.964723277
kgp21021677	4	47843116	CORIN | NFXL1	ENST00000563286	ENSE00002580194	47842139	47846356	-0.023880765	0.914311036	0.964723277
kgp407144	11	82817623	RAB30 | PCF11	ENST00000527627	ENSE00002161892	82817521	82817761	0.041992829	0.914369038	0.964723277
rs1034285	14	62027847	PRKCH | HIF1A	ENST00000508827	ENSE00002070975	62027461	62031959	0.105094954	0.867021791	0.966056545
kgp338202	11	28538833	METT5D1 | KCNA4	ENST00000524707	ENSE00002171684	28538379	28538862	0.117473273	0.869644155	0.966056545
kgp24548590	2	224368767	KCNE4 | SCG2	ENST00000422118	ENSE00001754732	224368700	224369329	0.100291454	0.872671079	0.966056545
kgp8332324	22	32672837	SLC5A4 | RFPL3	ENST00000452181	ENSE00001763735	32672672	32673173	-0.045086635	0.939381971	0.966397103
rs12163987	5	87572026	TMEM161B | LOC645323	ENST00000496733	ENSE00002085384	87571728	87572039	-0.043321094	0.94183225	0.966397103
rs13153995	5	169624772	FOXI1 | LOC133874	ENST00000520275	ENSE00002089995	169624756	169624833	0.031291721	0.942969295	0.966397103
kgp13055840	11	82817635	RAB30 | PCF11	ENST00000527627	ENSE00002161892	82817521	82817761	0.055393423	0.918360891	0.967033465
kgp18032149	3	156800768	LEKR1 | CCNL1	ENST00000471357	ENSE00001879700	156799456	156801064	-0.05047391	0.918520989	0.967033465
kgp11440937	16	80635032	DYNLRB2 | CDYL2	ENST00000570137	ENSE00002617613	80631803	80636416	-0.051777475	0.919310903	0.967033465
kgp8001281	9	37090151	LOC100287249 | ZCCHC7	ENST00000429493	ENSE00001770690	37086665	37090398	-0.020562918	0.920019248	0.967033465
kgp9470939	20	48884195	CEBPB | PTPN1	ENST00000445003	ENSE00001708071	48884036	48884200	0.11619913	0.920616865	0.967033465
kgp22791774	20	29641597	FRG1B | DEFB115	ENST00000446917	ENSE00001678897	29641421	29641792	-0.032922007	0.922755745	0.967033465
kgp7332261	3	128579208	ERV3 | LOC653712	ENST00000567253	ENSE00002589601	128578559	128579369	0.024527112	0.922851054	0.967033465
rs10186377	2	201691502	BZW1 | CLK1	ENST00000568571	ENSE00002589934	201689396	201692061	-0.020268194	0.923016341	0.967033465
kgp19793041	15	44826812	CTDSPL2 | LOC645212	ENST00000313807	ENSE00001427514	44825691	44827672	0.021161338	0.924431477	0.967033465
kgp20118429	8	79750404	IL7 | STMN2	ENST00000565297	ENSE00002583800	79749764	79752757	-0.045274298	0.926197297	0.967033465
rs1062858	4	47843628	CORIN | NFXL1	ENST00000563286	ENSE00002580194	47842139	47846356	0.019407315	0.926452059	0.967033465
kgp11988439	16	66924245	PDP2 | CDH16	ENST00000563086	ENSE00002616204	66923094	66924996	-0.022222676	0.927095638	0.967033465
kgp7305099	8	10334778	MSRA | LOC346702	ENST00000524047	ENSE00002099410	10334633	10334804	0.0227812	0.927898009	0.967033465
rs217660	14	62331662	SNAPC1 | SYT16	ENST00000554436	ENSE00002508325	62331598	62331976	0.032458298	0.928014961	0.967033465
kgp25165482	1	223318269	TLR5 | SUSD4	ENST00000435108	ENSE00001645603	223317879	223318296	-0.029918533	0.928576459	0.967033465
kgp14423932	2	42119914	SLC8A1 | LOC400950	ENST00000442214	ENSE00001777568	42119669	42120186	-0.169055382	0.887937365	0.967159324

kgp287541	21	36509034	RUNX1 | SETD4	ENST00000455028	ENSE00001623290	36508935	36509146	-0.079378632	0.888253552	0.967159324
rs6915085	6	160008204	FNDC1 | SOD2	ENST00000430078	ENSE00001683375	160007987	160008987	-0.031701911	0.930103116	0.967788328
rs9285574	14	46175550	LOC644589 | LOC10028958	ENST00000557602	ENSE00002486553	46175453	46175705	-0.122860052	0.883175568	0.968631719
kgp4991630	12	126470637	TMEM132B | LOC10028870	ENST00000534849	ENSE00002270684	126470392	126470780	-0.116247495	0.884677747	0.968631719
rs7534854	1	180535187	ACBD6 | XPR1	ENST00000442621	ENSE00001599647	180534387	180535654	0.105328522	0.886588294	0.968631719
rs6902553	6	14977206	CD83 | JARID2	ENST00000437648	ENSE00001647747	14977019	14977549	0.084480349	0.889493543	0.968631719
kgp3242992	3	163021072	OTOL1 | LOC730129	ENST00000494897	ENSE00001813150	163020984	163021076	0.088715732	0.89260395	0.968631719
kgp12335915	2	179266973	OSBPL6 | PRKRA	ENST00000565104	ENSE00002619842	179265561	179267618	0.08203813	0.895886428	0.968631719
kgp3312317	1	25295402	RUNX3 | SYF2	ENST00000568143	ENSE00002580573	25294914	25297356	-0.07553901	0.906482211	0.968631719
kgp9086514	14	42074040	FBXO33 | LRFN5	ENST00000557067	ENSE00002496796	42073957	42074059	0.106065891	0.906590383	0.968631719
kgp5223481	8	102087616	YWHAZ | ZNF706	ENST00000514926	ENSE00002063679	102087397	102088479	0.08171061	0.907100509	0.968631719
rs9923437	16	79710028	MAF | DYNLRB2	ENST00000563360	ENSE00002587690	79710005	79710105	-0.071739132	0.907285044	0.968631719
kgp23240877	21	46410328	C21orf70 | NCRNA00162	ENST00000439088	ENSE00001802548	46409779	46411747	-0.185495281	0.894112133	0.968675208
kgp2952197	2	849731	LOC100128185 | LOC39134	ENST00000414556	ENSE00001751314	849658	849896	-0.155205418	0.894933867	0.968675208
kgp14709527	2	42120567	SLC8A1 | LOC400950	ENST00000398796	ENSE00001534923	42119669	42121179	-0.251577616	0.89853452	0.968675208
kgp26873029	3	182457509	SOX2OT | ATP11B	ENST00000565024	ENSE00002612508	182457457	182458636	0.182299507	0.898677699	0.968675208
kgp8930928	5	81842023	ATP6AP1L | TMEM167A	ENST00000512952	ENSE00002025952	81841681	81842129	0.079726759	0.911940537	0.968806892
kgp25675517	16	17919604	XYLT1 | LOC100288502	ENST00000564852	ENSE00002589454	17919109	17920763	0.080070217	0.913907835	0.968806892
kgp4402157	14	97976603	VRK1 | C14orf64	ENST00000554862	ENSE00002441632	97976592	97976702	-0.060887904	0.920479947	0.970296236
rs1912132	2	132396225	ACTBL3 | C2orf27A	ENST00000431979	ENSE00001608232	132394598	132396751	-0.05889416	0.92621246	0.970296236
rs3811628	2	181941282	UBE2E3 | ITGA4	ENST00000456895	ENSE00001700483	181941265	181941351	-0.062692997	0.926718836	0.970296236
kgp7261358	4	24671556	DHX15 | SOD3	ENST00000569621	ENSE00002600133	24671185	24673191	-0.059700467	0.928250066	0.970296236
rs2906173	7	2486810	LOC100288594 | LFNG	ENST00000313156	ENSE00001270623	2485577	2487485	-0.040104274	0.951231222	0.97184614
kgp19578333	14	62036334	PRKCH | HIF1A	ENST00000508827	ENSE00002064690	62036262	62036395	-0.035566593	0.954260351	0.971931839
kgp7574419	6	3905475	LOC100289591 | PRPF4B	ENST00000566733	ENSE00002617674	3905144	3912213	-0.081368672	0.908922688	0.97277274
kgp9066301	1	181144537	IER5 | LOC100287948	ENST00000438428	ENSE00001743313	181144473	181144569	-0.168015694	0.91277603	0.97277274
kgp9602228	5	77638715	AP3B1 | LOC728769	ENST00000513755	ENSE00002060483	77638693	77638769	0.079131635	0.914837306	0.97277274
kgp1828913	5	86093524	COX7C | RASA1	ENST00000507653	ENSE00002083508	86093502	86093655	-0.072926354	0.914894025	0.97277274
rs2610128	8	137827853	KHDRBS3 | FLJ45872	ENST00000520068	ENSE00002130161	137827374	137827931	-0.074267561	0.91517346	0.97277274
kgp25577295	16	50672433	NKD1 | SNX20	ENST00000565077	ENSE00002621561	50671504	50674771	0.128641557	0.916700713	0.97277274
kgp6530086	15	40664096	DISP2 | C15orf23	ENST00000561261	ENSE00002571029	40664026	40664323	-0.062669206	0.918351887	0.97277274
rs2776153	21	29491481	C21orf94 | NCRNA00161	ENST00000453420	ENSE00001640835	29488413	29492193	0.073481857	0.925471864	0.972927918
kgp10947938	7	125558060	POT1 | GRM8	ENST00000411856	ENSE00001767248	125557925	125558172	-0.05275184	0.926374051	0.972927918
kgp8091302	12	93501232	LOC100287580 | NUDT4	ENST00000551928	ENSE00002372876	93501084	93501376	-0.05047761	0.93554447	0.972927918
kgp9737412	17	27342481	SEZ6 | LOC100130794	ENST00000426489	ENSE00001798773	27342360	27342688	-0.045540674	0.935993804	0.972927918
kgp1282571	12	127809323	LOC121296 | LOC10028841	ENST00000545739	ENSE00002265105	127808700	127809350	-0.075198724	0.936705812	0.972927918
kgp9202170	14	101536982	SNORD114-31 | LOC10013(	ENST00000444846	ENSE00001755005	101536824	101537078	-0.18806934	0.937748533	0.972927918
rs812086	11	19322717	E2F8 | NAV2	ENST00000529082	ENSE00002200326	19322708	19322791	0.051689793	0.938540109	0.972927918
kgp31134295		119148854	NKAP | RHOXF2B	ENST00000454625	ENSE00001750982	119148738	119149071	-0.056242168	0.93975012	0.972927918
kgp15960509	18	4294960	LOC284215 | LOC642597	ENST00000565811	ENSE00002615565	4293160	4295405	0.053044905	0.94096797	0.972927918
kgp597555	2	221498699	SLC4A3 | EPHA4	ENST00000414512	ENSE00001649993	221498653	221498849	-0.074682324	0.941177357	0.972927918
kgp3487849	13	114630504	FLJ44054 | RASA3	ENST00000562710	ENSE00002578109	114629487	114631817	-0.033153163	0.936225085	0.973319274
kgp16357663	16	31060889	STX4 | ZNF668	ENST00000570266	ENSE00002577122	31060834	31061201	0.019688861	0.938306675	0.973442321
kgp12468925	4	53609129	KIAA0114 | LOC100288413	ENST00000443173	ENSE00001782809	53608719	53612110	0.022870722	0.939038794	0.973442321
rs4916897	5	87581588	TMEM161B | LOC645323	ENST00000507736	ENSE00002025247	87581558	87581668	0.017556207	0.93908163	0.973442321
kgp19796872	15	95025840	MCTP2 | LOC440311	ENST00000565106	ENSE00002296294	95024040	95027181	0.019110785	0.939569432	0.973442321
kgp18931960	12	64901032	TBK1 | RASSF3	ENST00000541885	ENSE00002239048	64900946	64901255	0.048491031	0.936921561	0.973742342
kgp10678565	16	63092572	CDH8 | CDH11	ENST00000568741	ENSE00002616828	63091122	63093812	-0.058304687	0.938038456	0.973742342
kgp16045172	18	65151640	CDH19 | DSEL	ENST00000562669	ENSE00002612652	65149028	65152203	-0.031613115	0.94371486	0.976395976
kgp3534175	2	217475535	RPL37A | IGFBP2	ENST00000441803	ENSE00001659572	217475467	217475925	-0.021941323	0.94411799	0.976395976
kgp9221932	9	37087622	LOC100287249 | ZCCHC7	ENST00000429493	ENSE00001770690	37086665	37090398	-0.014598709	0.944847142	0.976395976
rs10865224	2	47565795	BCYRN1 | EPCAM	ENST00000418539	ENSE00001641977	47558199	47571656	0.044244268	0.945924747	0.97655668
kgp29212380	4	185286498	LOC728175 | IRF2	ENST00000512674	ENSE00002040634	185286341	185286799	0.039534721	0.952114663	0.97655668
kgp11048091	7	90964874	FZD1 | MTERF	ENST00000449361	ENSE00001649664	90964870	90965056	-0.03544369	0.956594481	0.97655668
kgp6525225	14	62028237	PRKCH | HIF1A	ENST00000508827	ENSE00002070975	62027461	62031959	-0.031616386	0.959480739	0.97655668
rs356439	5	139064994	CXXC5 | PSD2	ENST00000515296	ENSE00002052233	139064913	139065017	0.030861624	0.959690535	0.97655668
kgp777342	4	27209562	STIM2 | PCDH7	ENST00000382007	ENSE00001490576	27209127	27211372	-0.030768514	0.960280736	0.97655668
rs13101237	3	99219235	DCBLD2 | COL8A1	ENST00000471993	ENSE00001874478	99219191	99219385	0.071716727	0.949559958	0.97788744
kgp1595401	15	40664092	DISP2 | C15orf23	ENST00000561261	ENSE00002571029	40664026	40664323	-0.042085748	0.950781972	0.97788744
kgp3381231	6	29475039	MAS1L | UBD	ENST00000436804	ENSE00001780769	29474946	29475445	-0.034425131	0.952813403	0.97788744
kgp18302739	9	37090020	LOC100287249 | ZCCHC7	ENST00000429493	ENSE00001770690	37086665	37090398	0.014214935	0.947183844	0.977939826
kgp4173397	5	114540060	TRIM36 | PGGT1B	ENST00000507241	ENSE00002051389	114539713	114541943	0.013863952	0.948369295	0.977939826
kgp2789344	2	89130884	LOC100132330 | LOC10028	ENST00000418209	ENSE00001769310	89130700	89130914	0.02200147	0.948771778	0.977939826
kgp6628616	15	80125261	KIAA1024 | MTHFS	ENST00000560760	ENSE00002565064	80124808	80125317	-0.042867991	0.950090247	0.97846325
rs10079949	5	117846644	LOC100287135 | DTWD2	ENST00000506769	ENSE00002076127	117846583	117846759	-0.027865403	0.965538978	0.978586802
rs7705011	5	173002143	STC2 | LOC285593	ENST00000520300	ENSE00002119188	173001941	173002257	-0.048704034	0.95678451	0.979619463
kgp22782025	14	70937595	ADAM21 | ADAM20	ENST00000556964	ENSE00002518821	70935598	70938309	-0.025817162	0.952907429	0.980527934
kgp11391473	7	76602298	POMZP3 | LOC100288878	ENST00000434531	ENSE00002505131	76601996	76602357	-0.027644388	0.953721872	0.980530068
kgp11663907	8	2559353	LOC100286951 | CSMD1	ENST00000520570	ENSE00002133497	2558974	2559395	0.025985716	0.963696862	0.981849874
kgp6630219	12	2039741	LOC100288635 | DCP1B	ENST00000418006	ENSE00001804023	2038368	2040596	0.029860631	0.964414018	0.981849874
kgp8078157	3	181252640	DNAJC19 | SOX2OT	ENST00000482559	ENSE00001855729	181252403	181252835	0.034763258	0.965829014	0.981849874
kgp5806670	5	2737167	IRX4 | IRX2	ENST00000560688	ENSE00002540557	2736776	2737435	0.021748453	0.972186309	0.982006373
rs4633961	2	42120198	SLC8A1 | LOC400950	ENST00000398796	ENSE00001534923	42119669	42121179	-0.052186712	0.968924843	0.982668458
kgp57981	8	2532317	LOC100286951 | CSMD1	ENST00000523971	ENSE00002098887	2532238	2532353	-0.015518043	0.95670312	0.982758013
rs4756926	11	12559691	PARVA | TEAD1	ENST00000526112	ENSE00002172414	12559630	12560091	0.017285434	0.958266674	0.983051325
kgp20844288	4	78320784	CCNG2 | CXCL13	ENST00000513871	ENSE00002040651	78320747	78320948	-0.015894133	0.958816869	0.983051325
kgp2160627	4	8357835	HTRA3 | ACOX3	ENST00000505448	ENSE00002069127	8357038	8359103	-0.010328578	0.959918509	0.983051325
kgp28126700	14	85995929	SEL1L | FLRT2	ENST00000380722	ENSE00001486021	85994961	85996332	0.013791155	0.960246489	0.983051325
rs766424	21	35334666	ATP5O | FLJ46020	ENST00000381181	ENSE00001487739	35334367	35336260	0.028850178	0.970182443	0.985108327
kgp10378776	1	98433312	DPYD | FLJ35409	ENST00000561881	ENSE00002583625	98432561	98434370	-0.018593368	0.97929713	0.985869593
kgp17779510	3	193920863	HES1 | LOC100131551	ENST00000415869	ENSE00001780985	193920805	193921468	-0.013096314	0.964564879	0.985870001
kgp22782732	15	25279942	PAR5 | SNORD109A	ENST00000552334	ENSE00002349489	25277020	25281637	0.009336625	0.964633365	0.985870001
kgp12183668	6	38131442	ZFAND3 | BTBD9	ENST00000448738	ENSE00001662516	38131289	38131580	-0.018351681	0.982609206	0.985895524
kgp6768246	22	42670740	LOC388906 | NFAM1	ENST00000446578	ENSE00001617224	42670662	42670827	0.019604632	0.967259242	0.987644148
kgp27380241	9	37089224	LOC100287249 | ZCCHC7	ENST00000429493	ENSE00001770690	37086665	37090398	-0.008475447	0.968005822	0.987644148
kgp2276069	12	3152486	TEAD4 | TSPAN9	ENST00000513358	ENSE00002302855	3150603	3152614	0.026882825	0.976211624	0.988189681

kgp7326035	16	81773267	CMIP | PLCG2	ENST00000569731	ENSE00002594889	81773231	81773339	0.016787304	0.977285654	0.988810249
rs9471759	6	42060835	TAF8 | C6orf132	ENST00000562471	ENSE00002625332	42059976	42061997	-0.0075461	0.970729094	0.98883137
kgp15037176	22	42670612	LOC388906 | NFAM1	ENST00000332965	ENSE00001324190	42669900	42670868	-0.014810799	0.971502166	0.98883137
kgp30946689		152690573	ZFP92 | TREX2	ENST00000569962	ENSE00002593900	152689210	152691934	0.016537677	0.973571581	0.98883137
kgp10109346	17	13680505	HS3ST3A1 | CDRT15P	ENST00000423323	ENSE00001689622	13679951	13680666	0.006609958	0.973895589	0.98883137
kgp5778966	12	44113667	ADAMTS20 | PUS7L	ENST00000553202	ENSE00002345647	44112796	44117803	-0.007562374	0.973987823	0.98883137
rs7148896	14	54316607	DDHD1 | BMP4	ENST00000418927	ENSE00001646248	54316440	54316688	0.014947214	0.97408492	0.98883137
kgp1134123	12	115151772	TBX3 | MED13L	ENST00000551875	ENSE00002365231	115151616	115151804	0.008590965	0.989192975	0.989192975
kgp14746834	2	113348386	CHCHD5 | LOC100131455	ENST00000436885	ENSE00001631650	113348373	113348457	0.008964225	0.976211792	0.990157675
kgp19991988	15	95026115	MCTP2 | LOC440311	ENST00000565106	ENSE00002296294	95024040	95027181	-0.005926119	0.977351574	0.990481402
rs3099300	6	166526499	LOC729681 | T	ENST00000456477	ENSE00001617535	166526175	166526761	-0.012843626	0.982854534	0.99210493
kgp11812305	19	35304910	ZNF599 | LOC401913	ENST00000561778	ENSE00002616079	35302738	35305249	-0.011771929	0.985627276	0.992568313
kgp1678172	4	189923138	TRIML1 | FRG1	ENST00000508799	ENSE00002037518	189922924	189923229	-0.008426287	0.991792771	0.992729965
kgp2298600	4	148515501	EDNRA | TMEM184C	ENST00000509370	ENSE00002049794	148515417	148515739	0.006325238	0.992329284	0.992729965
kgp20769550	4	76007055	DKFZP564O0823 | RCHY1	ENST00000561705	ENSE00002576268	76006912	76007567	-0.005813095	0.992729965	0.992729965
rs2296048	6	5870350	FARS2 | LOC100287622	ENST00000454882	ENSE00001708041	5870274	5870453	0.007208016	0.984205541	0.995774091
kgp15840615	1	229228872	RHOU | RAB4A	ENST00000433734	ENSE00001664536	229228562	229229035	0.008160246	0.985306205	0.995774091
kgp12135327	6	6682662	LY86 | RREB1	ENST00000563225	ENSE00002602400	6680542	6683866	-0.004156651	0.985504235	0.995774091
kgp11236681	3	114033960	TIGIT | ZBTB20	ENST00000570269	ENSE00002576360	114033348	114035026	0.003244981	0.987085298	0.995774091
rs12082567	1	247351237	ZNF124 | LOC729806	ENST00000566446	ENSE00002602464	247350583	247351828	-0.004079215	0.987628273	0.995774091
kgp5410646	2	231556613	SP100 | CAB39	ENST00000415174	ENSE00001768231	231555636	231556948	-0.007247158	0.98861138	0.995774091
kgp2161255	16	81422611	GAN | CMIP	ENST00000568107	ENSE00002626991	81416874	81424489	-0.003186748	0.989043858	0.995774091
rs798794	14	62027910	PRKCH | HIF1A	ENST00000508827	ENSE00002070975	62027461	62031959	0.005292267	0.989174097	0.995774091
kgp9961999	21	23305674	NCAM2 | NCRNA00158	ENST00000452500	ENSE00001616092	23305635	23305919	-0.005524309	0.992271916	0.996411659
kgp3830095	8	9117795	PPP1R3B | TNKS	ENST00000518589	ENSE00002093747	9117538	9117805	-0.005220241	0.992704556	0.996411659
kgp6115364	12	126470564	TMEM132B | LOC10028870	ENST00000534849	ENSE00002270684	126470392	126470780	0.004144824	0.994235183	0.996411659
kgp8279234	4	4323980	ZNF509 | D4S234E	ENST00000509015	ENSE00002051780	4323689	4324272	-0.002226518	0.996411659	0.996411659
kgp16727363	13	30681811	UBL3 | KATNAL1	ENST00000432770	ENSE00001729921	30681736	30682108	0.004241438	0.991934982	0.996473619
kgp9137347	2	139359465	SPOPL | NXPH2	ENST00000562796	ENSE00002608781	139357233	139359996	0.00377467	0.99228803	0.996473619
kgp7137558	1	17524010	LOC400743 | PADI1	ENST00000539219	ENSE00002261175	17520556	17524112	0.00436675	0.992345725	0.996473619
rs2168188	15	101710986	LRRK1 | CHSY1	ENST00000558515	ENSE00002566308	101710755	101711026	0.001582378	0.996202914	0.998345484
rs12153829	6	8784491	HULC | OFCC1	ENST00000429060	ENSE00001761237	8784411	8784570	0.001047473	0.996803701	0.998345484
kgp11498263	9	37089873	LOC100287249 | ZCCHC7	ENST00000429493	ENSE00001770690	37086665	37090398	-0.000905683	0.997007402	0.998345484
kgp3097370	6	169558416	SMOC2 | THBS2	ENST00000449466	ENSE00001768361	169558356	169558551	-0.000748766	0.998133772	0.998345484
kgp20035716	15	95027130	MCTP2 | LOC440311	ENST00000565106	ENSE00002296294	95024040	95027181	0.000477333	0.998345484	0.998345484

Table S3A. Top gene hits from *LincIN* knockdown by shRNA1 (*P*<0.001)

Gene Symbol	FC(shRNA1/Vec)	P-Value	FDR
**SLAMF7***	20.25	2.30E-06	0.00583
**SLCO4C1**	17.32	2.90E-06	0.00583
**TMEM156**	14.36	5.70E-06	0.00747
**OLR1**	13.34	6.50E-06	0.00747
**PTPRQ**	25.4	7.60E-06	0.00764
**LOC729974**	11.06	9.00E-06	0.00805
**ARHGDIB**	8.33	2.13E-05	0.0153
**OR2M3**	8.32	2.84E-05	0.0153
**LOC730755**	7.36	3.13E-05	0.0153
INSIG1	5.61	3.59E-05	0.0153
**PLXDC2**	6.54	3.74E-05	0.0153
**TLR4**	5.85	3.75E-05	0.0153
**MYCT1**	11.06	3.78E-05	0.0153
**HMGCS1**	7.3	4.06E-05	0.0153
**DSC2**	5.97	4.51E-05	0.0153
**F2RL2**	6.45	4.87E-05	0.0153
**MERTK**	5.19	5.26E-05	0.0153
IL18	6.78	5.66E-05	0.0153
**CXCR4**	11.43	6.10E-05	0.0153
**PCDH7**	4.4	6.45E-05	0.0153
DUSP5	4.7	6.70E-05	0.0153
CTGF	5.15	6.76E-05	0.0153
**UGT8**	10.7	7.47E-05	0.0153
**NAALAD2**	5.18	7.79E-05	0.0153
**SLC16A7**	4.26	7.93E-05	0.0153
**NR4A2**	4.18	8.07E-05	0.0153
**LOC152742**	0.22	8.09E-05	0.0153
**APCDD1L**	0.19	8.31E-05	0.0153
**MAL2**	5.92	8.43E-05	0.0153
**LOC149773**	0.17	8.46E-05	0.0153
**NTN4**	5.84	8.54E-05	0.0153
**ARAP2**	4.87	8.55E-05	0.0153
**B4GALT6**	6.09	8.66E-05	0.0153
EDN1	4.33	8.73E-05	0.0153
**C10orf116**	0.25	8.99E-05	0.0154
**RHOJ**	4.86	9.85E-05	0.0165
**ID1**	0.2	0.000101	0.0166
**DOCK10**	4.13	0.0001074	0.0168
**DUSP1**	6.64	0.0001078	0.0168
**MGC4294**	0.25	0.0001149	0.0168
**IL8**	3.85	0.0001169	0.0168
**TNFSF15**	4.04	0.0001169	0.0168
**CHRDL1**	4.55	0.000117	0.0168
**VCAM1**	0.26	0.0001271	0.0171
**GCNT4**	3.7	0.0001358	0.0171
**FAM49A**	4.57	0.0001397	0.0171
**KIF16B**	4.42	0.0001415	0.0171
**SCN9A**	5.21	0.0001511	0.0171
**TMEM27**	3.72	0.0001526	0.0171
**KRTAP21-3**	0.24	0.0001618	0.0171
**CPA4**	0.3	0.0001714	0.0178
**NOV**	3.51	0.0001747	0.0178

Table S3B. Top gene hits from *LincIN* knockdown by shRNA2 (*P*<0.001)

Symbol	FC (shRNA2/Vec)	P-Value	FDR
SNORA36C	0.054	9.00E-07	0.00268
**SLCO4C1**	20.11	1.00E-06	0.00268
**SLAMF7**	12.84	2.40E-06	0.00312
**TMEM156**	7.92	6.10E-06	0.00368
**VCAM1**	0.1	6.80E-06	0.00368
**DSC2**	6.99	6.90E-06	0.00368
**PTPRQ**	19.38	8.90E-06	0.00368
**OR2M3**	8.32	1.48E-05	0.00479
**MYCT1**	12.51	1.55E-05	0.00479
**NTN4**	6.16	1.65E-05	0.00479
**ANXA10**	0.22	2.03E-05	0.00479
**OLR1**	15	2.07E-05	0.00479
**LOC730755**	6.75	2.08E-05	0.00479
TNFSF18	0.16	2.17E-05	0.00479
SNORA36C	0.19	2.24E-05	0.00479
**ARHGDIB**	6.94	2.26E-05	0.00479
**GCNT4**	4.45	2.26E-05	0.00479
**ESAM**	7.8	2.70E-05	0.00517
**ROBO1**	4.24	2.91E-05	0.00532
SOST	0.12	3.15E-05	0.00535
MIR539	4.12	3.24E-05	0.00535
**PLXDC2**	7.78	3.26E-05	0.00535
**ARAP2**	5.12	3.52E-05	0.00555
**NAALAD2**	5.16	3.69E-05	0.00556
**CDK15**	0.11	3.72E-05	0.00556
**TLR4**	4.33	3.99E-05	0.00556
**UGT8**	9.88	4.02E-05	0.00556
**DYSF**	4.4	4.53E-05	0.00566
IGLJ2	4.51	4.62E-05	0.00566
**B4GALT6**	6.15	4.69E-05	0.00566
FPR1	0.22	4.87E-05	0.00566
**PCDH7**	5.95	5.04E-05	0.00566
**FST**	3.82	5.04E-05	0.00566
**DOCK10**	4.3	5.16E-05	0.00569
**RHOJ**	4.5	5.31E-05	0.00576
**ACSS3**	5.52	5.59E-05	0.00576
SUSD3	0.28	5.62E-05	0.00576
**MAL2**	6.1	5.65E-05	0.00576
**C10orf116**	0.25	6.21E-05	0.006
**LOC286467**	0.25	6.41E-05	0.006
**ZNF804A**	8.31	6.51E-05	0.006
ST6GALNAC2	0.2	6.56E-05	0.006
AKR1C3	0.29	6.57E-05	0.006
**CHRDL1**	4.21	6.71E-05	0.006
PRAMEF3	0.28	6.92E-05	0.006
**MGC4294**	0.28	7.15E-05	0.006
**FBXL13**	0.29	7.25E-05	0.006
**NOV**	3.44	7.38E-05	0.006
CNGA1	0.21	7.99E-05	0.00607
**LPHN2**	3.19	8.04E-05	0.00607
RNU6ATAC3P	0.29	8.09E-05	0.00607
**CELF2**	3.81	8.10E-05	0.00607

**CD24**	4.13	0.0001748	0.0178
**SHC3**	3.6	0.0001817	0.0178
**LINC00261**	7.49	0.0001834	0.0178
**ZNF804A**	6.96	0.0001837	0.0178
**KHDRBS3**	3.47	0.0001889	0.018
**TTC9**	5.57	0.000192	0.018
**MMP1**	27.21	0.0001973	0.0182
**CDK15**	0.18	0.0002009	0.0184
**LPHN2**	3.23	0.0002054	0.0186
**LOC100128054**	6.35	0.0002104	0.0188
**GABRE**	0.3	0.0002136	0.0188
**SYNPR-AS1**	4.32	0.0002168	0.0188
**FGF5**	11.85	0.0002241	0.0192
ARG2	4.27	0.0002387	0.0198
**ACSS3**	4.37	0.0002513	0.0204
ITFG1	3.56	0.0002605	0.0208
**CLDN1**	6.15	0.000261	0.0208
**MYO5C**	3.25	0.0002725	0.0215
**ROBO1**	3.85	0.0002934	0.0229
STARD4	3.14	0.0002977	0.023
MITF	3.21	0.00031	0.0234
**PTPRQ**	3.06	0.0003139	0.0234
**PDE7B**	0.24	0.0003142	0.0234
**LOC286467**	0.3	0.0003169	0.0234
GRIK2	0.24	0.0003323	0.0237
**SPDEF**	3.15	0.0003413	0.024
**CELF2**	4.02	0.0003455	0.024
**PXDN**	3.32	0.0003547	0.0243
**DHCR7**	3.51	0.0003674	0.0243
**MIR4500**	3.12	0.0003719	0.0243
SPRY1	3.02	0.0003751	0.0243
**PBX1**	0.19	0.0003758	0.0243
ANKRD1	2.91	0.0003772	0.0243
HSD17B7P2	3.26	0.0003788	0.0243
**GABRE**	0.25	0.0003827	0.0243
**GRAMD1B**	0.24	0.0003842	0.0243
**RNF43**	0.29	0.0003914	0.0244
AK5	3.08	0.0003967	0.0244
**SORCS2**	0.34	0.0004022	0.0245
TGFBI	0.33	0.0004061	0.0245
PKP2	3.24	0.0004092	0.0245
**CFH**	6.68	0.0004105	0.0245
**C3**	0.29	0.0004156	0.0246
**MAOA**	3.79	0.0004193	0.0246
**FBXL13**	0.37	0.0004261	0.0248
LIPH	4.33	0.0004285	0.0248
**HMGCR**	3.21	0.0004354	0.0249
**TNFRSF10D**	2.86	0.0004366	0.0249
SCD	2.96	0.0004456	0.0251
MBNL3	3.13	0.0004485	0.0251
**DYSF**	4.56	0.0004548	0.0252
**DOCK3**	0.33	0.0004618	0.0255
SYTL2	2.72	0.000485	0.0256
**ZNF521**	4.17	0.0004897	0.0256
EMB	4.66	0.0004924	0.0256
LINC00478	3.3	0.0004928	0.0256
CTGF	2.75	0.0004937	0.0256
**RELN**	2.97	0.0005075	0.026
LIPG	2.89	0.0005297	0.0262

**FAM49A**	4.22	8.31E-05	0.00607
**CPA4**	0.31	8.32E-05	0.00607
**DOCK3**	0.29	8.33E-05	0.00607
**HMGCS1**	3.64	8.37E-05	0.00607
**KRTAP21-3**	0.27	8.53E-05	0.00613
**CD24**	3.95	8.67E-05	0.00617
SNORA38B	0.22	8.98E-05	0.00623
**DUSP1**	5.15	9.13E-05	0.00628
**NR4A2**	3.57	9.36E-05	0.00636
**CFH**	6.2	9.44E-05	0.00636
**ID1**	0.25	9.53E-05	0.00636
**LRRTM3**	3.81	0.0001001	0.00636
VTRNA1-2	0.31	0.0001027	0.00636
COX7B2	0.32	0.0001034	0.00636
**LOC152742**	0.28	0.0001036	0.00636
**C3**	0.29	0.0001045	0.00636
**SPDEF**	3.15	0.0001067	0.00636
PHGDH	0.32	0.0001085	0.00637
MIR548I2	0.28	0.0001093	0.00637
**SORCS2**	0.32	0.0001105	0.0064
TAF1D	0.28	0.0001115	0.00641
MNS1	0.34	0.000117	0.00658
RNY1P5	0.33	0.0001185	0.00662
LOC100506610	0.25	0.0001224	0.00675
**PBX1**	0.25	0.0001262	0.00675
**ATP10A**	2.92	0.0001271	0.00675
LOC100507009	4.07	0.000131	0.00679
**SLC16A7**	4.25	0.0001335	0.00682
**PXDN**	3.54	0.0001343	0.00682
ANGPT1	0.3	0.0001357	0.00682
**GRAMD1B**	0.26	0.0001369	0.00684
**TNFSF15**	2.91	0.0001421	0.00706
**GNAO1**	0.29	0.0001516	0.00735
**MMP8**	7.13	0.0001591	0.00749
**KHDRBS3**	2.75	0.0001599	0.00749
**SLC14A1**	2.8	0.0001635	0.00752
**F2RL2**	2.96	0.0001635	0.00752
**RELN**	3.39	0.0001679	0.00753
LAPTM5	0.34	0.0001684	0.00753
**LOC100128054**	3.53	0.0001697	0.00753
FAM20C	0.27	0.0001719	0.00753
**DAPK1**	2.84	0.0001722	0.00753
**LOC149773**	0.27	0.000177	0.00761
ALS2CR11	0.29	0.0001777	0.00761
CST7	0.34	0.000186	0.00778
**KIF16B**	4.22	0.0001861	0.00778
MKX	0.33	0.0001868	0.00778
ATP8B2	2.68	0.0001881	0.00778
**CLDN1**	5.65	0.0001887	0.00778
MIR550A1	3.43	0.0001892	0.00778
**UST**	3.6	0.0001975	0.00803
**MMP1**	25.15	0.0001988	0.00803
SEMA3A	3.45	0.0001995	0.00803
**TNFRSF10D**	3.07	0.0002007	0.00803
**DTNA**	3.84	0.0002027	0.00803
**CXCR4**	5.7	0.0002065	0.00803
MIR3118-5 // M	0.38	0.000207	0.00803
POTEC	0.38	0.000207	0.00803
**GABRE**	0.29	0.0002108	0.00803

**NRK**	6.85	0.0005382	0.0263
**ADAMTS1**	4.6	0.0005411	0.0263
**LOC283299**	3.2	0.0005421	0.0263
RASGRP3	3.69	0.000548	0.0264
**ATP10A**	3	0.0005623	0.0267
**SERPINI1**	2.6	0.0005666	0.0267
**PPM1K**	2.62	0.0005718	0.0267
FDFT1	2.98	0.0005784	0.0267
SH3BGRL2	2.95	0.0005796	0.0267
HMSD	2.62	0.0005862	0.0267
DSG2	2.61	0.0005959	0.0267
LPIN1	2.68	0.0005963	0.0267
**ESAM**	6.72	0.0006075	0.0267
**UST**	3.56	0.000613	0.0267
**SEL1L3**	3.06	0.0006211	0.0267
SEMA3E	2.87	0.000624	0.0267
**ZG16B**	2.53	0.0006245	0.0267
**SLC22A15**	3.74	0.0006254	0.0267
IL24	2.65	0.0006294	0.0267
FZD7	0.38	0.0006328	0.0267
**ZNF704**	3.25	0.0006435	0.0269
**DTNA**	3.95	0.000646	0.0269
**MMP8**	8.49	0.0006499	0.027
CDH1	2.68	0.0006616	0.0272
SLCO4A1	2.96	0.0006651	0.0272
SLC38A4	3.19	0.0006654	0.0272
DPY19L2P2	0.37	0.0006762	0.0275
**FST**	2.7	0.0007079	0.0285
ID3	0.32	0.0007176	0.0286
**LOC201651**	3.2	0.0007233	0.0287
**LRIG1**	0.38	0.0007284	0.0287
**PTGS2**	3.41	0.0007433	0.029
TCP11L2	3.36	0.0007444	0.029
**LRRTM3**	3.25	0.0007452	0.029
MXRA8	2.74	0.0007528	0.0291
KYNU	3.28	0.0007596	0.0292
**BMPR1B**	2.46	0.0007617	0.0292
SCG5	0.28	0.0007706	0.0294
ST3GAL6-AS1	2.44	0.0007822	0.0296
**GNAO1**	0.33	0.0007895	0.0296
**CST4**	3.12	0.0007942	0.0296
**DAPK1**	2.71	0.0008086	0.03
**ANXA10**	0.3	0.0008126	0.03
ADAM17	2.43	0.0008247	0.0303
**RAB27B**	3.49	0.000843	0.0308
SREBF2	2.48	0.0008458	0.0308
**BANK1**	2.57	0.0008581	0.0311
PHLPP1	2.38	0.0008741	0.0315
**SLC14A1**	2.47	0.0008862	0.0315
**NMNAT2**	2.61	0.0008916	0.0315
**KRTAP4-8**	3.36	0.0008921	0.0315
**PDE3B**	2.6	0.0008928	0.0315
FRAS1	2.96	0.0009054	0.0318
**LOC100507127**	2.41	0.000911	0.0319
HMGN2	0.41	0.0009226	0.032
**HAS2**	3.53	0.000924	0.032
LOC643486	3	0.0009339	0.0323
CROT	0.41	0.0009594	0.033
**SQLE**	3.16	0.000967	0.0331

**LRIG1**	0.36	0.0002157	0.00803
SAMD12-AS1	2.75	0.0002159	0.00803
**SHC3**	4.02	0.000217	0.00803
**SCN9A**	4.05	0.0002171	0.00803
MIR328	0.22	0.0002189	0.00803
MIR328	0.22	0.0002189	0.00803
PFKFB2	3.97	0.0002211	0.00803
MPV17L2	2.6	0.0002229	0.00804
JAG1	2.78	0.0002291	0.00819
SNORD114-27	3.08	0.0002374	0.00834
LGALS12	0.23	0.0002454	0.00837
NUPR1	0.36	0.0002457	0.00837
DDX39B	2.73	0.0002479	0.00837
DDX39B	2.73	0.0002479	0.00837
DDX39B	2.73	0.0002479	0.00837
MIR568	0.33	0.0002491	0.00837
**LOC201651**	3.38	0.0002498	0.00837
SCARNA4	0.24	0.0002517	0.00837
**ADAMTS1**	4.43	0.0002518	0.00837
**APCDD1L**	0.32	0.0002546	0.00843
MIR3179-3 // M	2.9	0.0002618	0.00849
COL1A2	0.4	0.0002618	0.00849
MIR5047	3.4	0.0002628	0.00849
**MAOA**	3.13	0.0002657	0.00855
**MIR4500**	2.77	0.0002682	0.00856
**SLC2A13**	2.76	0.0002703	0.00856
**TMEM27**	2.72	0.0002709	0.00856
**MERTK**	2.64	0.000275	0.00861
RASA4	0.37	0.0002772	0.00861
**LOC729974**	8.23	0.0002837	0.00863
**PDE7B**	0.31	0.0002839	0.00863
TMEM173	2.94	0.0002846	0.00863
**DHCR7**	2.69	0.0002876	0.00863
SNORA14A	0.16	0.0002927	0.00872
FOXA2	3.55	0.0002952	0.00877
LOC100133123	0.36	0.0002974	0.00877
**MYO5C**	2.49	0.0003017	0.00885
**ZNF704**	2.97	0.0003034	0.00885
MAN1A1	3.88	0.0003137	0.00907
**PTPRQ**	2.49	0.0003143	0.00907
CRLF2	0.39	0.0003185	0.00907
ABCA1	0.31	0.0003194	0.00907
MMP16	0.38	0.000321	0.00907
KRTAP2-2	0.35	0.0003216	0.00907
CDON	0.39	0.0003264	0.00909
ZNF724P	0.39	0.0003278	0.0091
CPVL	2.69	0.0003398	0.00922
NFE4	0.34	0.0003403	0.00922
PEAR1	2.58	0.000342	0.00922
BAI3	2.43	0.0003425	0.00922
ASNS	0.25	0.0003436	0.00922
**LOC100507127**	2.53	0.0003471	0.00928
**LINC00261**	5.02	0.0003512	0.00936
PLEKHS1	0.35	0.0003538	0.0094
GPR98	2.61	0.0003675	0.0096
ITGB4	0.39	0.0003714	0.0096
**FGF5**	8.26	0.0003722	0.0096
GRIK1-AS2 // B	2.38	0.0003751	0.00962
**RNF43**	0.39	0.0003822	0.0097

PRKG1	2.41	0.0009696	0.0331
**SLC2A13**	2.61	0.0009784	0.0332
**RCAN1**	2.76	0.0009936	0.0336

*: Common hits (n=122) from shRNA1 and shRNA2 treatments are shown in bold.

SNORA26	0.39	0.000384	0.0097
MIR9-1	2.42	0.0003845	0.0097
MORC4	0.42	0.0003889	0.00975
SAMD5	2.67	0.000389	0.00975
PDK4	0.43	0.0003974	0.00987
**SEL1L3**	2.84	0.0003977	0.00987
BCL2	2.45	0.000398	0.00987
SORBS2	0.38	0.0004049	0.00999
LRRC8C	2.55	0.0004123	0.0101
OR5P3	3.53	0.000426	0.0102
**PDE3B**	3.24	0.0004267	0.0102
DBF4	0.44	0.0004277	0.0102
PPYR1	0.39	0.0004297	0.0102
DET1	0.41	0.0004307	0.0102
LONRF2	0.41	0.0004322	0.0102
RN5S322	0.38	0.0004386	0.0102
AADAC	2.3	0.0004413	0.0102
MGAT4A	0.41	0.0004457	0.0102
SSX7	0.4	0.0004459	0.0102
VTRNA2-1	0.18	0.0004486	0.0102
**LOC283299**	2.88	0.0004495	0.0102
SCARA3	0.44	0.0004521	0.0102
MIR27B	0.43	0.0004533	0.0102
MIR378D2	0.44	0.0004564	0.0103
TSPAN2	0.4	0.0004625	0.0104
RNU6-81	0.36	0.0004718	0.0104
MIR4684	2.26	0.0004723	0.0104
SNORD113-3	2.74	0.0004732	0.0104
**GABRE**	0.32	0.0004749	0.0104
SUSD5	2.51	0.0004758	0.0104
LPL	0.42	0.0004778	0.0104
MGLL	2.3	0.0004789	0.0104
**NMNAT2**	2.72	0.0004807	0.0104
**SYNPR-AS1**	3.33	0.0004839	0.0104
IL11	2.43	0.0004839	0.0104
HERC2P9	2.46	0.0004846	0.0104
**HMGCR**	2.27	0.0004859	0.0104
TNFSF10	0.4	0.0004869	0.0104
**RAB27B**	3.04	0.0004893	0.0104
4-Mar	2.87	0.000491	0.0104
CRTAM	0.33	0.000492	0.0104
ADAM22	0.35	0.0004925	0.0104
KGFLP2	2.26	0.0004925	0.0104
BAIAP2L1	0.44	0.000495	0.0104
KDSR	2.48	0.0005013	0.0104
**SLC22A15**	3.52	0.0005064	0.0105
LOXL2	2.31	0.0005079	0.0105
AKT3-IT1	2.27	0.0005099	0.0105
**HAS2**	4.56	0.0005247	0.0107
NR3C2	3.01	0.0005268	0.0107
LOC146880	2.83	0.0005395	0.0109
SNORA70G	3.64	0.0005514	0.011
PELI2	2.46	0.0005672	0.0112
**SERPINI1**	2.45	0.0005706	0.0112
GPX7	0.43	0.0005723	0.0112
TNFRSF11A	2.46	0.000575	0.0112
PKIA	2.31	0.0005784	0.0112
FXC1	2.25	0.0005795	0.0112
VNN1	0.43	0.0005824	0.0112

**IL8**	2.47	0.0005896	0.0113
SNORD13P3	0.33	0.0005958	0.0113
DHRS3	0.33	0.0006059	0.0114
SLC5A6	2.17	0.0006081	0.0115
**BMPR1B**	2.42	0.0006118	0.0115
STEAP2	0.41	0.0006301	0.0117
LOC100190986	2.34	0.0006308	0.0117
SLC4A11	2.15	0.0006327	0.0117
**KRTAP4-8**	4.46	0.0006359	0.0117
FPR2	0.44	0.0006462	0.0117
TAP2	2.17	0.0006626	0.0119
TAP2	2.17	0.0006626	0.0119
RNU4ATAC2P	0.38	0.0006826	0.0121
HLTF	0.47	0.0006898	0.0121
VWDE	2.32	0.0007022	0.0122
RN5S111	3.43	0.000716	0.0122
PGM5-AS1	3.14	0.000716	0.0122
**ZG16B**	2.26	0.0007171	0.0122
MIR373	2.67	0.0007269	0.0122
**ZNF521**	4.19	0.0007293	0.0122
MIR3690	2.2	0.0007321	0.0122
MIR3690	2.2	0.0007321	0.0122
LOC100132111	0.45	0.0007322	0.0122
**NRK**	8.58	0.0007385	0.0122
SNORA51	0.47	0.0007405	0.0122
LSS	2.23	0.0007413	0.0122
**SQLE**	2.1	0.000743	0.0122
BAK1	2.29	0.0007437	0.0122
**TTC9**	3.47	0.0007461	0.0122
**RCAN1**	2.62	0.0007471	0.0122
CPE	0.36	0.0007502	0.0122
SDR16C5	2.32	0.0007524	0.0122
LURAP1L	2.35	0.0007528	0.0122
CLCN6	2.4	0.0007582	0.0122
ATP8B1	2.1	0.0007583	0.0122
AIM2	0.39	0.0007603	0.0122
TNFAIP3	2.12	0.0007608	0.0122
MST4	0.46	0.0007624	0.0122
FAM104B	0.45	0.0007741	0.0123
SNORD113-9	2.79	0.0007778	0.0123
GPRC5B	2.2	0.0007787	0.0123
DEFB131	0.48	0.0007811	0.0123
NCF2	2.58	0.0007832	0.0123
PCDHB16	0.43	0.0007951	0.0124
MIR29A	0.34	0.0007992	0.0125
RP1-177G6.2	2.9	0.0008011	0.0125
TMEM104	2.36	0.0008085	0.0126
**PPM1K**	2.3	0.0008126	0.0126
ATF7IP2	2.1	0.0008171	0.0126
ITGA5	2.1	0.0008213	0.0126
LTB4R	2.22	0.0008253	0.0126
NAP1L3	2.13	0.0008329	0.0126
SNORD116-17	0.46	0.0008478	0.0127
SNORD116-17	0.46	0.0008478	0.0127
FKBP9L	2.51	0.0008485	0.0127
HLA-DRB3	2.44	0.0008501	0.0127
IRF2BP2	0.43	0.0008541	0.0127
CATSPERG	2.16	0.0008545	0.0127
EPAS1	2.2	0.0008719	0.0128

LOC100506486	2.41	0.0008756	0.0128
LOC100506486	2.41	0.0008756	0.0128
LRRC20	2.48	0.0008835	0.0128
RN5S201	0.22	0.0008837	0.0128
PPP1R15A	2.48	0.000887	0.0128
LOC100131826	2.07	0.0008931	0.0128
SNORD77 // SN	0.45	0.0008995	0.0129
ABL2	2.36	0.0009017	0.0129
PON2	0.47	0.0009019	0.0129
ASNA1	2.07	0.0009034	0.0129
HLA-DRB3	2.54	0.0009081	0.0129
LXN	0.44	0.0009119	0.0129
TAF12	0.31	0.0009179	0.013
LOC441666	2.28	0.000928	0.013
TSPAN8	2.31	0.0009304	0.013
LINC00328	0.31	0.0009309	0.013
**CST4**	2.61	0.0009405	0.013
**BANK1**	2.14	0.0009432	0.013
ZYX	2.1	0.0009476	0.013
LOC100288637	2.35	0.0009479	0.013
UAP1L1	2.03	0.0009511	0.013
LGMN	2.02	0.0009513	0.013
MIR376A2	2.36	0.0009534	0.013
SHFM1	2.09	0.0009568	0.013
ABCG2	0.47	0.0009612	0.013
IFIT3	2.06	0.0009624	0.013
MIR552	2.37	0.0009633	0.013
ZC3H6	0.47	0.0009698	0.0131
SNORA60	0.36	0.0009701	0.0131
RGS2	2.06	0.0009894	0.0132
**PTGS2**	2.74	0.0009934	0.0132
RASSF9	2.51	0.0009942	0.0132
ROCK1P1	0.22	0.0009959	0.0132

*: Common hits (n=122) from shRNA1 and shRNA2 treatments are shown in bold.

Table S4. Ingenuity Pathway Analysis (IPA) of top biological functions targeted by two independent shRNA knockdowns of LincIN

Category (A-Z)	AnalysisName	p-value
Behavior	Common	6.5E-04-1.91E-03
Cancer	Common	1.27E-06-5.44E-03
Carbohydrate Metabolism	Common	9.95E-04-4.13E-03
Cardiovascular Disease	Common	4.3E-07-5.11E-03
Cardiovascular System Development and Function	Common	4.7E-08-4.52E-03
**Cell Cycle**	**Common**	**7.81E-04-7.81E-04**
**Cell Death and Survival**	**Common**	**2.27E-05-5.44E-03**
**Cell Morphology**	**Common**	**3.24E-08-3.63E-03**
**Cell Signaling**	**Common**	**1.85E-03-3.53E-03**
**Cell-mediated Immune Response**	**Common**	**4.67E-03-4.76E-03**
**Cell-To-Cell Signaling and Interaction**	**Common**	**6.68E-06-5.27E-03**
**Cellular Assembly and Organization**	**Common**	**1.27E-03-1.27E-03**
**Cellular Compromise**	**Common**	**9.18E-05-4.13E-03**
**Cellular Development**	**Common**	**4.7E-08-4.98E-03**
**Cellular Function and Maintenance**	**Common**	**3.61E-06-4.76E-03**
**Cellular Growth and Proliferation**	**Common**	**3.61E-06-5E-03**
**Cellular Movement**	**Common**	**1.05E-06-5.27E-03**
Connective Tissue Development and Function	Common	2.23E-05-4.67E-03
Connective Tissue Disorders	Common	1.63E-07-5.14E-03
Dermatological Diseases and Conditions	Common	1.22E-06-5.27E-03
Developmental Disorder	Common	1.46E-04-2.72E-03
Digestive System Development and Function	Common	4E-04-3.86E-03
Drug Metabolism	Common	4.07E-04-8.6E-04
Embryonic Development	Common	1.27E-03-4.67E-03
Endocrine System Disorders	Common	1.79E-06-3.16E-03
Free Radical Scavenging	Common	8.82E-05-1.16E-03
Gastrointestinal Disease	Common	1.79E-06-5.36E-03
Hair and Skin Development and Function	Common	5.7E-04-3.86E-03
Hematological Disease	Common	1.24E-05-5E-03
Hematological System Development and Function	Common	7.12E-06-5.27E-03
Hematopoiesis	Common	1.13E-03-4.76E-03
Hepatic System Development and Function	Common	4E-04-7.81E-04
Hepatic System Disease	Common	4E-04-3.68E-03
Hereditary Disorder	Common	3.85E-04-3.85E-04
Humoral Immune Response	Common	2.72E-03-5.23E-03
Hypersensitivity Response	Common	1.23E-03-4.08E-03
Immune Cell Trafficking	Common	1.44E-05-5.27E-03
Immunological Disease	Common	1.19E-06-5E-03
Infectious Diseases	Common	4.59E-04-4.67E-03
Inflammatory Disease	Common	1.63E-07-5.27E-03
Inflammatory Response	Common	1.44E-05-5.27E-03
Lipid Metabolism	Common	9.95E-04-4.13E-03
Lymphoid Tissue Structure and Development	Common	6.48E-04-4.76E-03
Metabolic Disease	Common	3.24E-07-2.72E-03
Molecular Transport	Common	2.59E-04-3.87E-03
Nervous System Development and Function	Common	8.11E-04-3.28E-03
Neurological Disease	Common	2.11E-08-5.26E-03
Nucleic Acid Metabolism	Common	1.85E-03-1.85E-03
Nutritional Disease	Common	7.49E-06-7.49E-06
Ophthalmic Disease	Common	3.2E-04-4.51E-03
Organ Development	Common	8.99E-05-4.67E-03
Organ Morphology	Common	2.17E-04-4.67E-03
Organismal Development	Common	4.7E-08-4.67E-03
Organismal Functions	Common	3.17E-05-1.27E-03
Organismal Injury and Abnormalities	Common	5.62E-07-5.44E-03
Organismal Survival	Common	4.14E-04-3.07E-03
Post-Translational Modification	Common	3.6E-04-3.6E-04
Protein Degradation	Common	3.6E-04-3.6E-04
Protein Synthesis	Common	3.6E-04-4.02E-03
Psychological Disorders	Common	2.11E-08-4.16E-03
Renal and Urological Disease	Common	1.23E-06-2.72E-03
Renal and Urological System Development and Function	Common	3.59E-04-4.67E-03
Reproductive System Development and Function	Common	2.17E-04-4.09E-03
Reproductive System Disease	Common	5.62E-07-5.39E-03
Respiratory Disease	Common	2.64E-06-3.56E-03
Respiratory System Development and Function	Common	1.27E-03-1.27E-03
Skeletal and Muscular Disorders	Common	1.63E-07-5.14E-03

Skeletal and Muscular System Development and Function	Common	2.23E-05-4.67E-03
Small Molecule Biochemistry	Common	2.59E-04-4.13E-03
Tissue Development	Common	4.7E-08-4.76E-03
Tissue Morphology	Common	7.12E-06-4.52E-03
Tumor Morphology	Common	1.97E-04-5.44E-03
Visual System Development and Function	Common	7.29E-04-2.89E-03
Vitamin and Mineral Metabolism	Common	3.53E-03-4.08E-03
**Antimicrobial Response**	Unique For shRNA1	8.55E-04-8.55E-04
**Endocrine System Development and Function**	Unique For shRNA2	3.24E-04-3.24E-04
**Gene Expression**	Unique For shRNA1	3.23E-04-1.36E-03

Table S5. Associations between p21 scores among individual lung metastasis colonies

**p21 IHC Score**	**Vector**[a]	**shRNA1 (*p*-value**[b]**)**	**shRNA2 (*p*-value)**
H<50 (negative or low)	16	6 *(0.04)*	4 *(0.05)*
H≥50 (moderate or high)	5	9	7

[a] Reference category
[b] Fisher's exact test.

Nuclear staining was considered as positive for p21. H scores for p21 were generated by multiplying the staining intensity of nuclei (0, 1, 2, 3) by the percentage of positive cells (0 – 100%). H-scores that were below 50 were considered low for p21, while H-scores above 50 were considered high.
