# Supplementary material for: LincIN, a novel NF90-binding long non-coding RNA, is overexpressed in advanced breast tumors and involved in metastasis
Source: Breast Cancer Res. 2017 May 30;19:62. doi: 10.1186/s13058-017-0853-2 (PMC5450112; doi:10.1186/s13058-017-0853-2)
Supplement: Additional file 1: — Supplementary figures and tables. (ZIP 4.69 mb) [file 13058_2017_853_MOESM1_ESM.zip › LincIN_Manuscript_Suppl_0928.pdf]

Supplemental Data-Tables

Table S1, related to Methods. Screening shRNA sequences for knockdown efficiency in transient transfection

Table S2, related to Figure 1. High density array-based differential expression analysis of intergenic transcripts in breast tumors (T) *vs.* adjacent normal (N)

Table S3, related to Figure 3. Microarray analysis: Top gene hits from *LincIN* knockdown by shRNA1 (A) and shRNA2 (B)

Table S4, related to Figure 3. Ingenuity Pathway Analysis (IPA) of top biological functions targeted by two independent shRNA knockdowns of *LincIN*

Table S5. Related to Figure 3 and 5. Associations between p21 scores among individual lung metastasis colonies

Supplemental Figure Legend

Figure S1, related to Figure 1. High throughput profiling of intergenic lncRNA transcriptome using high density SNP array. (A) Representative Western blot images for primary HMECs. Protein lysates isolated from paired normal-tumor HMEC cells were tested for the expression of an epithelial marker, pan-keratin, and a fibroblast marker, vimentin. β-actin was used as a loading control. MCF-10F (an immortalized HMEC line) and 293FT (a human fibroblastic cell line) served as controls for keratin and vimentin, respectively. These results indicate that the HMEC cell lines that we have established are *bona fide* epithelial cells. (B) Schematic workflow of analyzing lncRNA transcripts using Illumina HumanOmni5 BeadChips. Start and end coordinates of annotated intergenic lncRNA exons were retrieved from the online genomic database Ensembl release 68, which is built on the Genome Reference Consortium release GRCh37 using the BioMart data management system. To obtain the array probes that fall within the lncRNA exons, a subset of intergenic probes (~2.4 millions) was overlapped with Ensembl annotated lncRNA exons in the intergenic region, resulting in lncRNA-specific probes, which were used for subsequent differential expression analysis. (C) A representative raw signal plot (sum XY) showed a high correlation between paired normal and tumor samples. (D) Distribution of HumanOmni5 probes in intergenic and protein coding regions indicated a high coverage of intergenic lncRNAs. (E) A Circos plot demonstrated the whole genome-wide view of lncRNA transcript signals in 5 normal-tumor paired samples analyzed in the present study. Log transformed values for normal (outer layer, red) and for tumor (inner layer, blue) samples were illustrated.

Figure S2, related to Figure 1. RT-PCR results of *LincIN* in different cellular fractions and poly(A) enriched RNAs. U2 snRNA and ribosome protein S14 were used as nuclear and cytoplasmic RNA controls, respectively.

Figure S3, related to Figure 1. Coding potentials of *LincIN* and other known coding and noncoding RNAs. The evolutionary protein-coding potential was determined by smoothed PhyloCSF score.

PhyloCSF examines evolutionary signatures characteristic of alignments of conserved coding regions. PhyloCSF provides more information than conservation of the amino acid sequence, because it distinguishes the different codons that code for the same amino acid. Smoothed PhyloCSF scores for sense sequences in three reading frames were generated using the UCSC Genome Browser by copying the URL “http://www.broadinstitute.org/compbio1/PhyloCSFtracks/trackHub/hub.txt” into the “My Hubs” tab under “track hubs”.

Figure S4, related to Figure 3. Expression of *LincIN* in breast cell lines. Evaluation of *LincIN* expression in two benign breast cell lines (MCF-10A and MCF-10F) and ten breast cancer cell lines by RT-qPCR (**: P< 0.01, ***: P<0.001 vs. MCF-10A, n=3).

Figure S5, related to Figure 3. (A) Representative image (left) and quantitative analysis (middle) of wound closure assay using MDA-MB-231 cells expressing *LincIN* shRNAs or vector control at 0 and 24 hr. Quantitative analysis of knockdown using *LincIN* shRNAs vs. empty vector by RT-qPCR (right). Data was analyzed using one-way ANOVA and Dunnett’s test to account for multiple post-hoc comparisons (***: P<0.005, **: P<0.01, and *: P<0.05; n=3). (B) Representative image (left) and quantitative analysis (middle) of wound closure assay using MCF10DCIS cells expressing *LincIN* or empty vector at 0 and 24 hr. Quantitative analysis of *LincIN* overexpression (right) was performed using empty vector or the sample vector carrying *LincIN* by RT-qPCR. Data was analyzed using Student *t*-test (*: P<0.05; repeated experiments and imaging taken at 100×). (C) Effects of *LincIN* on cell proliferation. After counting with a hemocytometer, 5000 cells/well were seeded in 96-well plates for each MCF-10A vector control or *LincIN*-overexpression, MCF10ADCIS vector control or *LincIN*-overexpression, and MDA-MB-231-luc shRNA control or *LincIN* knockdown lines. After 48 h, 10 ul/well of WST-1 was added to each well and absorbance was then measured at 450 nm versus a 650 nm reference by using a plate reader. Data was analyzed using Student *t*-test (*: P<0.05).

Figure S6. Related to Figure 3. Comparison of the effects of *LincIN* knockdown on the transcriptome by two independent shRNAs. MDA-MB-231 cells stably expressing vector, shRNA1 or shRNA2 were harvested at about 70% confluences. Total RNA was isolated using TRIzol reagent and the quality of total RNA was assessed by an Agilent 2100 Bioanalyzer. 250 ng total RNA samples were labeled and hybridized to the Affymetrix Human Gene 2.0 ST Array according to the manufacturer's instructions. For each condition, two biological replicate samples were used for microarray experiments. After hybridization, the slides were scanned and images were analyzed using the Affymetrix Gene Expression Console with the RMA (Robust Multi-array Average) normalization algorithm. (A) 173 and 321 differentially expressed genes were identified for shRNA1 and shRNA2 in comparison to the vector control, respectively ($P<0.001$). Statistical Analyses were performed using BRB-ArrayTools developed by Dr. Richard Simon and BRB-ArrayTools Development Team. (B) Correlation analysis of Log_2 (FC) in overlapping hits (n=122) between two shRNA groups (Speaman's Rho test). (C) Biological process networks of top "hits" (76 and 75 for shRNA1 and shRNA2, respectively) were generated using IPA (Ingenuity® Systems) ($P<0.01$).

Figure S7. Related to Figure 3 and 5. Knockdown *LincIN* increase p21 expression in MDA-MB231 cells in culture cells and lung metastasis colonies. (A) Western blot of p21, NF90 or beta-actin, in SC and *LincIN* knockdown groups of MDA-MB-231 cells, repeated experiment. (B) Examples of the expression of p21 and cytokeratin 18 (CK18, the luminal epithelial markers) , which was analyzed by immunohistochemistry (IHC) in lung metastasis colonies from shRNA vector and LincIN knockdown group. Antibody for p21 (OP64) and CK18 (DC10) were purchased from Calbiochem and Dako, respectively.
