# Supplementary material for: LincIN, a novel NF90-binding long non-coding RNA, is overexpressed in advanced breast tumors and involved in metastasis
Source: Breast Cancer Res. 2017 May 30;19:62. doi: 10.1186/s13058-017-0853-2 (PMC5450112; doi:10.1186/s13058-017-0853-2)

Figure S1. Genome-wide profiling of intergenic lncRNA transcriptome using high-density SNP arrays in paired primary normal and malignant HMECs

A

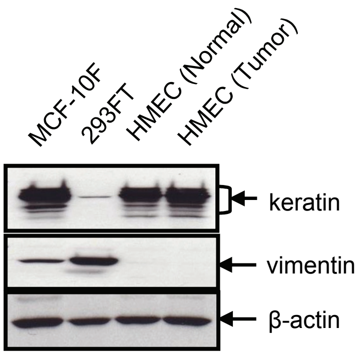

B

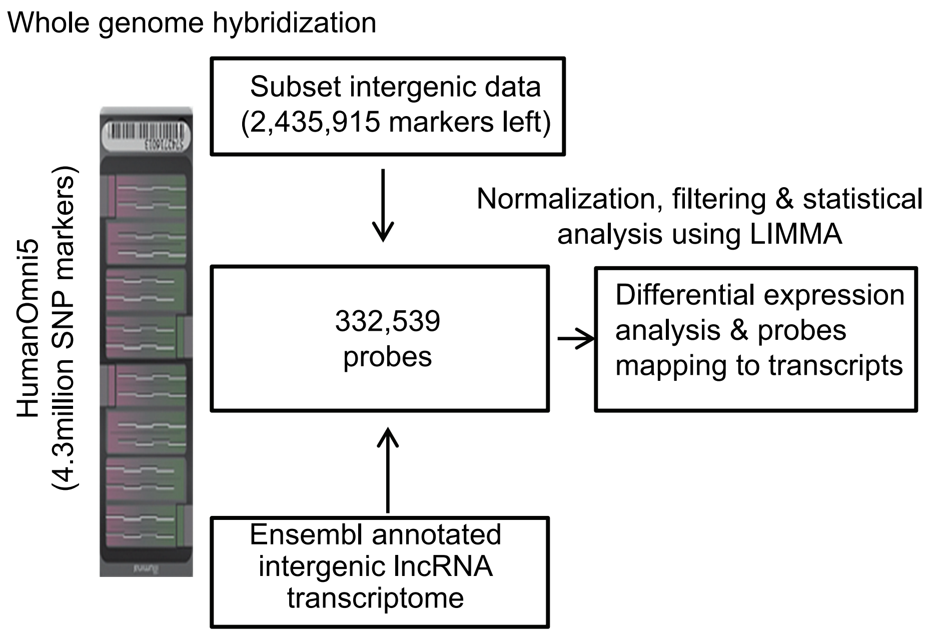

C

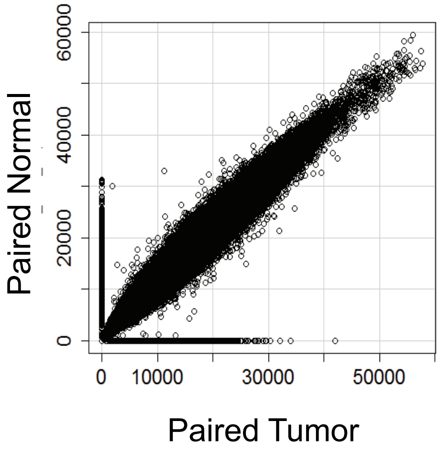

D

Type	Intergenic lncRNA	Protein coding
# probes targeting each region	2,435,915	1,812,712
# Genes in the database[1]	6,631	22,665
# probes targeting annotated genes	332,539	257,366
Mean space per gene	50	11
# Exons in each region	8,659	618,319
# probes targeting exons	332,539	257,366
Mean space per exon	38.4	0.4

[1] Ensembl Builder 70

E

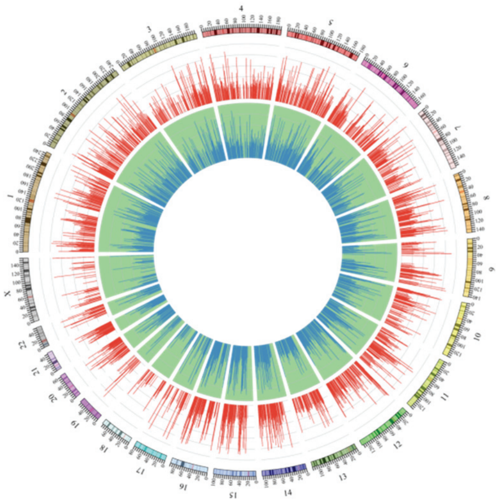

Figure S2. RT-PCR results of *LincIN* in different cellular fractions and poly(A) enriched RNAs. U2 snRNA and *ribosome protein S14* were used as nuclear and cytoplasmic RNA controls, respectively.

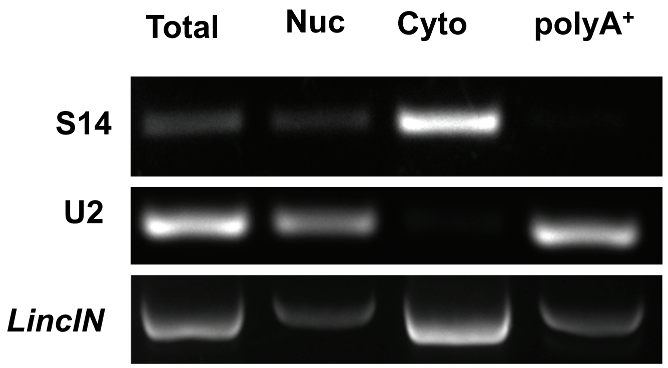

Figure S3. Coding potentials of *LincIN* as well as other known coding and noncoding RNAs.

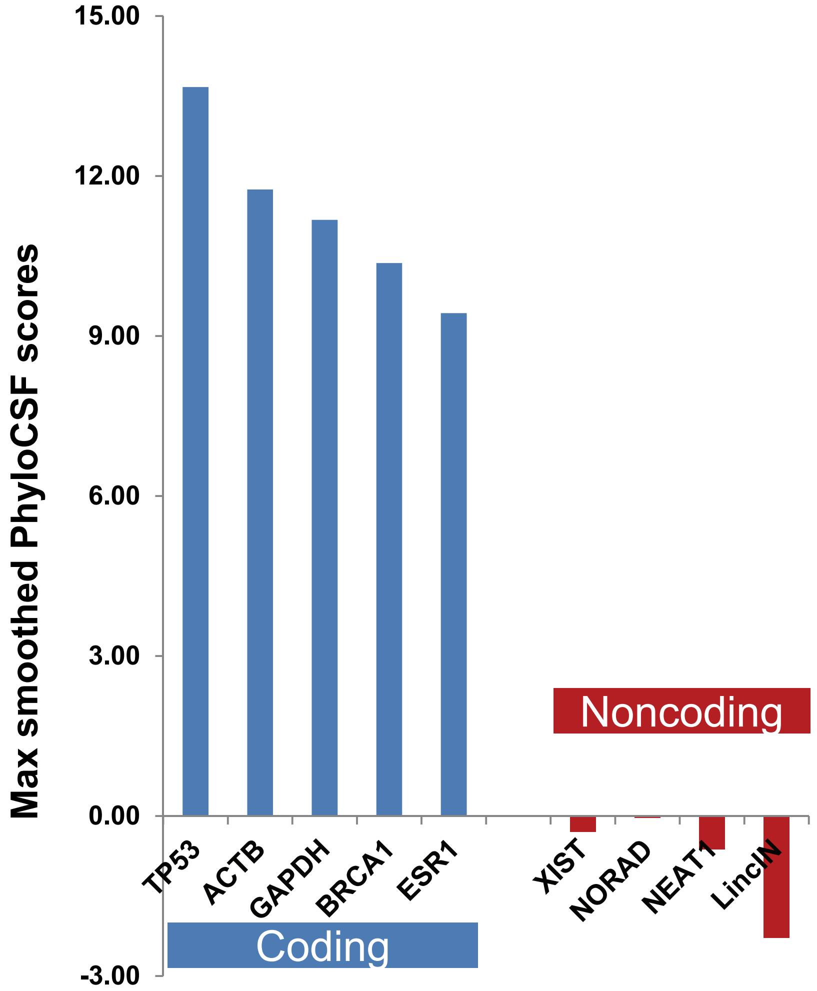

Figure S4. *LincIN* expression in breast cell lines

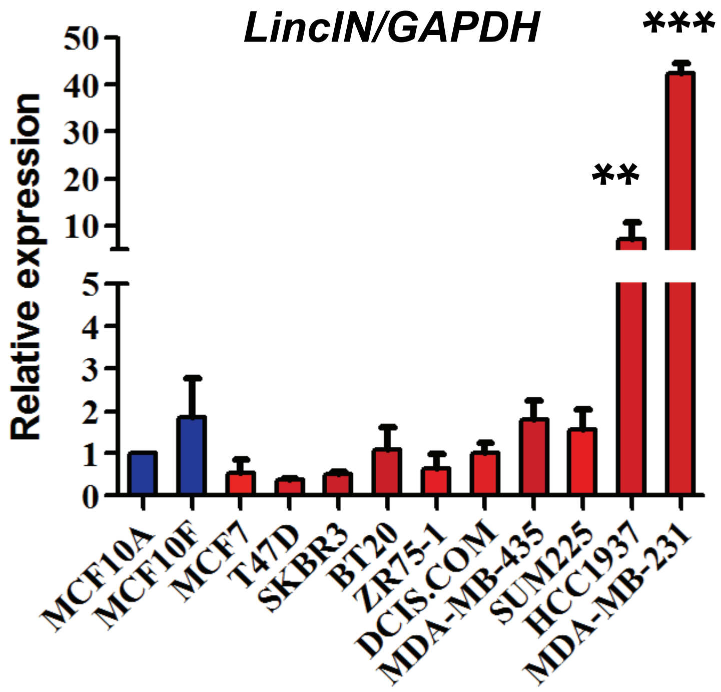

Figure S5A. *LincIN* mediates MDA-MB-231 cells migration *in vitro*

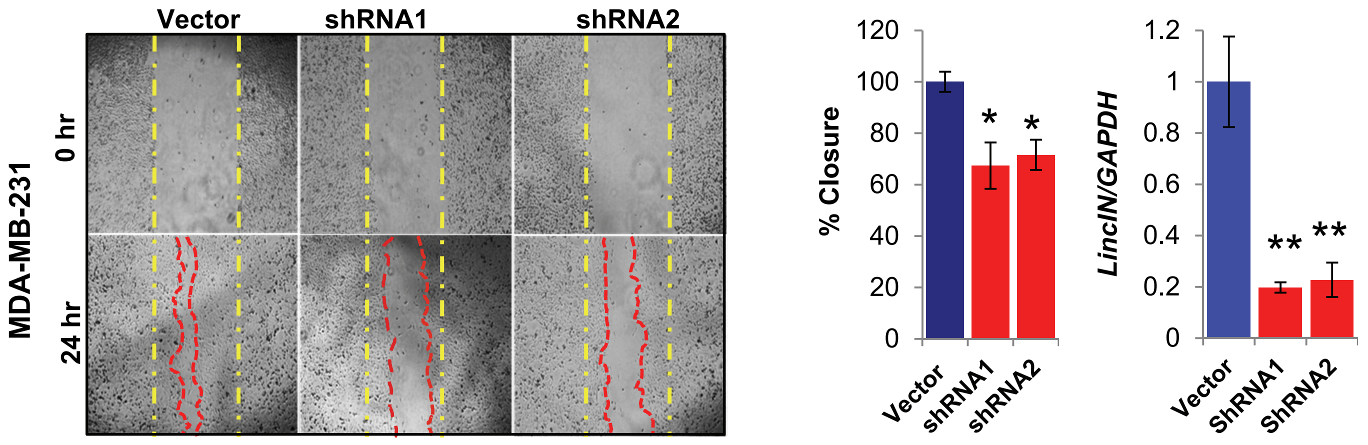

Figure S5B. *LincIN* mediates MCF10ADCIS cells migration *in vitro*

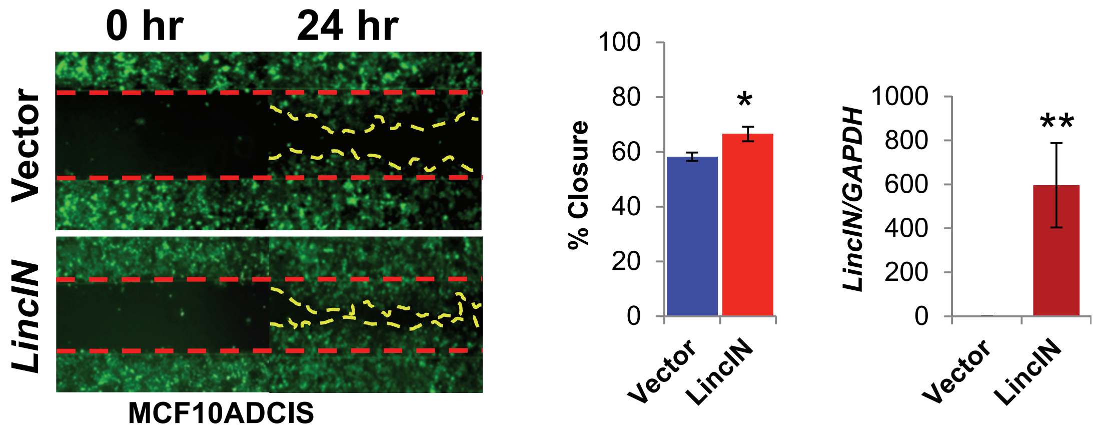

Figure S5C. Effects of *LincIN* on cell proliferation

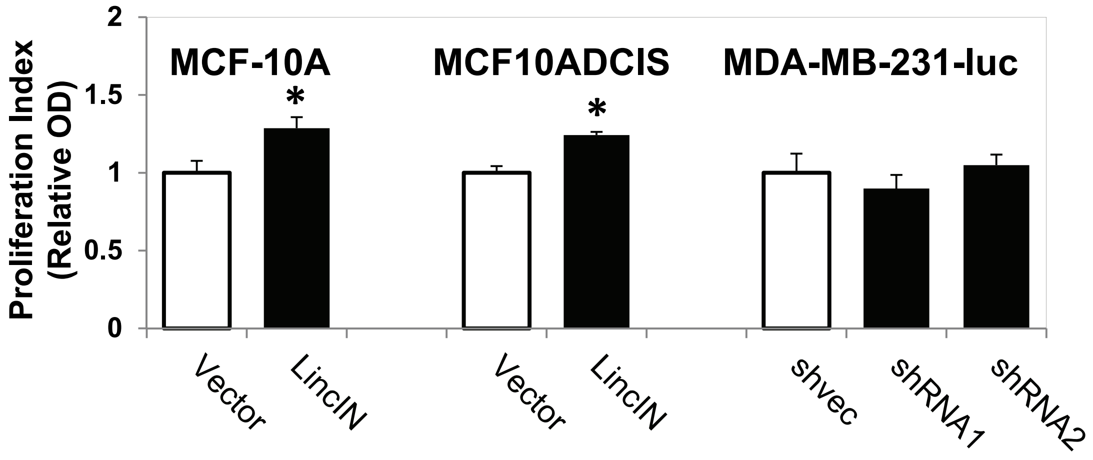

Figure S6. Comparison of the effects of *LincIN* knockdown on transcriptome by two independent RNAi.

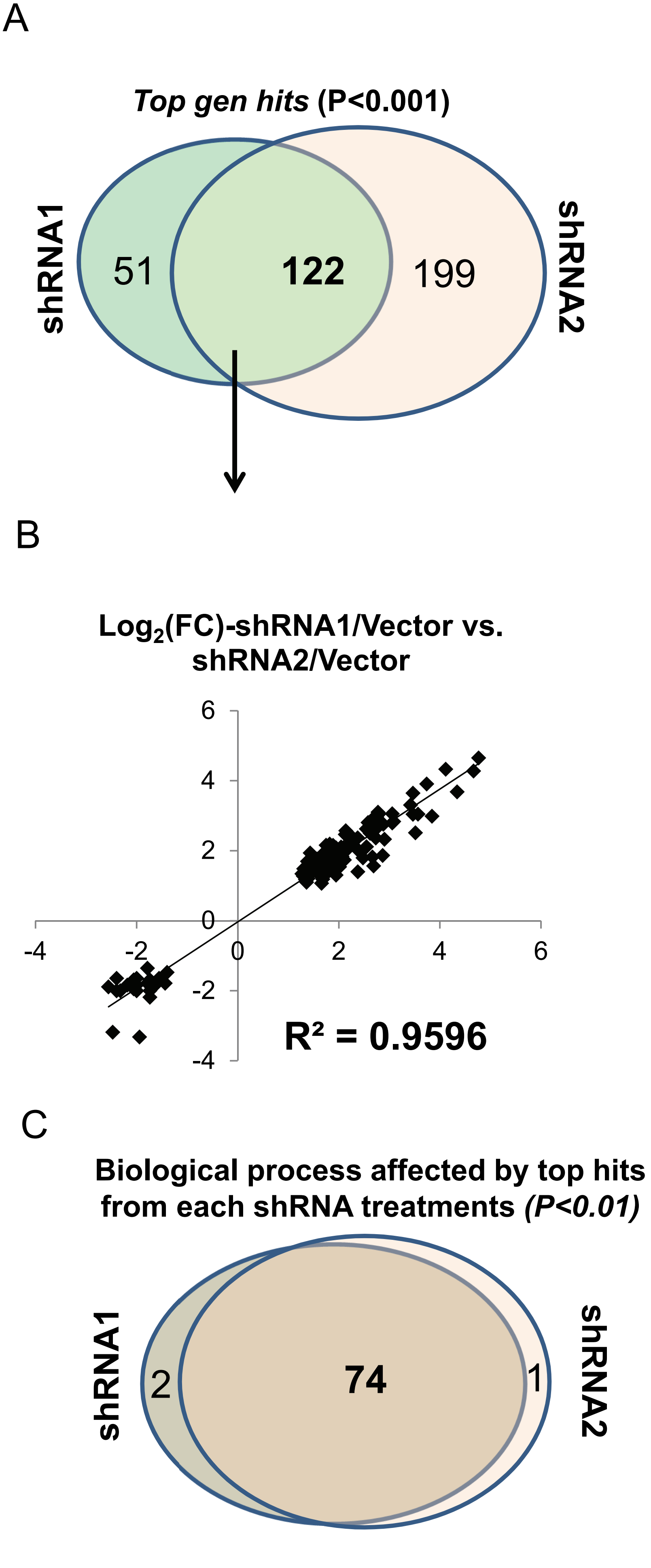

Figure S7. Knockdown *LincIN* increase p21 expression in MDA-MB231 cells in culture cells (A) and metastasis colonies (B).

A.

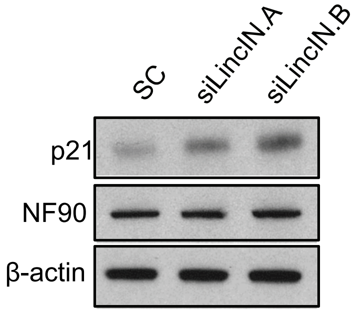

Figure S7 (cont.) Knockdown LincIN increase p21 expression in MDA-MB231 cells in culture cells (A) and metastasis colonies (B).

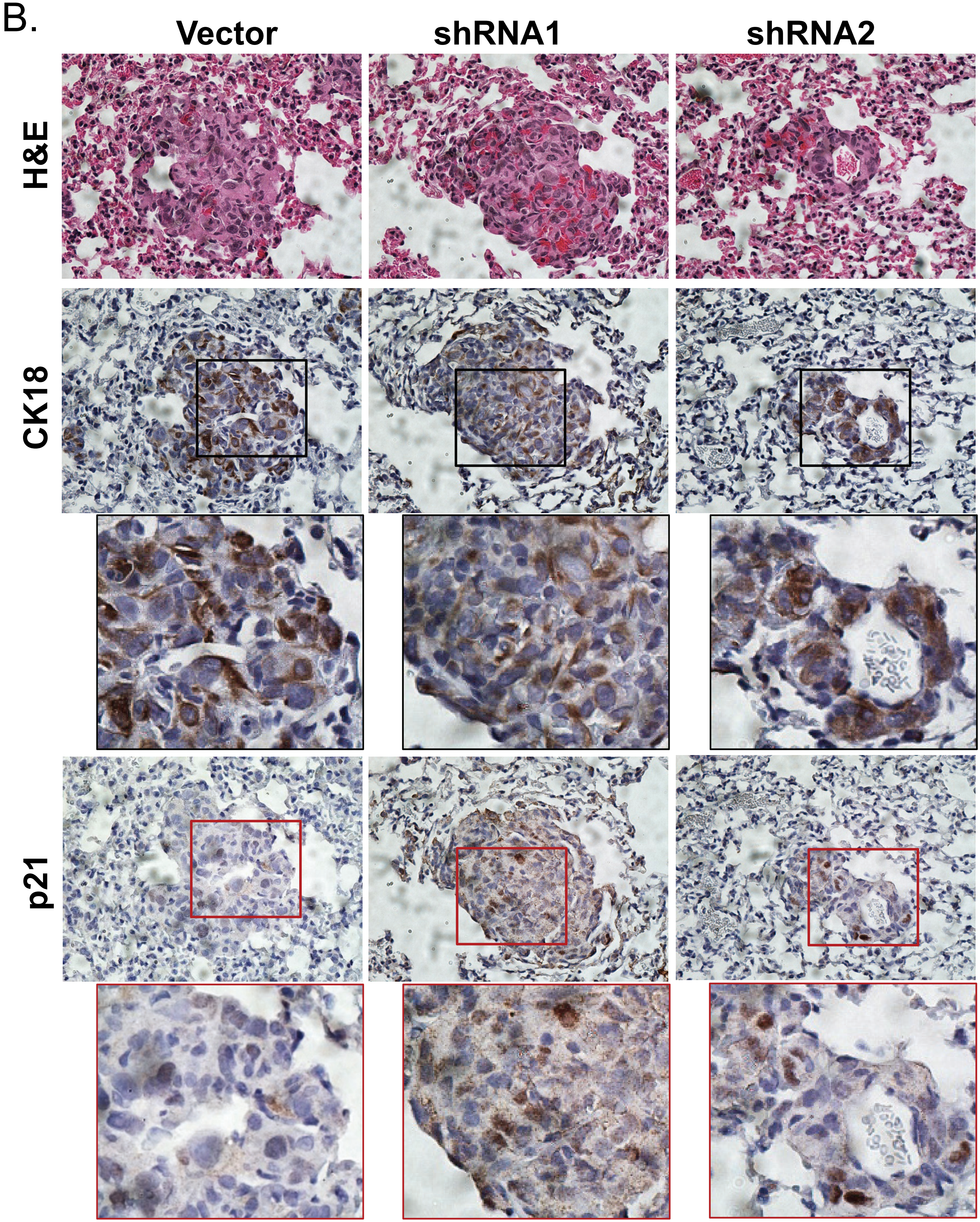

Supplement: Additional file 1: — Supplementary figures and tables. (ZIP 4.69 mb) [file 13058_2017_853_MOESM1_ESM.zip › Supplement Figures_0111.pdf]
